# Contaminating plasmid sequences and disrupted vector genomes in the liver following adeno-associated virus gene therapy

**DOI:** 10.1038/s41591-025-04073-z

**Published:** 2026-01-16

**Authors:** Sarah Buddle, Li-An K. Brown, Sofia Morfopoulou, Oscar Enrique Torres Montaguth, Mariacristina Scoto, Vanessa Herder, Anil Dhawan, Julianne R. Brown, Laura Atkinson, Angelika Kopec, Dee Davis, Nathaniel Storey, Luis Campos, Neil Sebire, Hannah Macpherson, Jasmaine Lee, Richard Orton, Giovanni Baranello, Patawee Asamaphan, Georgios Ilia, Rajvinder Karda, Holly Belfield, ISARIC4C Investigators, Malcolm Gracie Semple, J. Kenneth Baillie, John Counsell, Simon N. Waddington, Emma C. Thomson, Francesco Muntoni, Judith Breuer

**Affiliations:** 1 Infection, Immunity and Inflammation Department, Great Ormond Street Institute of Child Health, University College London, London, UK; 2 Dubowitz Neuromuscular Centre, University College London Great Ormond Street Institute of Child Health and Great Ormond Street Hospital, London, UK; 3 National Institute for Health Research Great Ormond Street Hospital Biomedical Research Centre, London, UK; 4 Medical Research Council–University of Glasgow Centre for Virus Research, Glasgow, UK; 5 Paediatric Liver GI and Nutrition Centre, King’s College Hospital, London, UK; 6 Mowatlabs, King’s College Hospital, London, UK; 7 Department of Microbiology, Virology and Infection Control, Great Ormond Street Hospital for Children NHS Foundation Trust, London, UK; 8 Histopathology Department, Great Ormond Street Hospital for Children NHS Foundation Trust, London, UK; 9 Long Read Sequencing Facility, Department of Neurodegenerative Disease, Queen Square Institute of Neurology, University College London, London, UK; 10 Department of Genetics and Genomic Medicine, Great Ormond Street Institute of Child Health, University College London, London, UK; 11 EGA Institute for Women’s Health, University College London, London, UK; 12 Critical Care Research Team, Great Ormond Street Hospital NHS Foundation Trust, London, UK; 13 Research Department of Targeted Intervention, Division of Surgery and Interventional Science, University College London, London, UK; 14 Pandemic Institute, University of Liverpool, Liverpool, UK; 15 Respiratory Medicine, Alder Hey Children’s Hospital NHS Foundation Trust, Liverpool, UK; 16 Baillie Gifford Pandemic Science Hub, Centre for Inflammation Research, University of Edinburgh, Edinburgh, UK; 17 Roslin Institute, University of Edinburgh, Edinburgh, UK; 18 Intensive Care Unit, Royal Infirmary of Edinburgh, Edinburgh, UK

**Keywords:** Next-generation sequencing, Metagenomics

## Abstract

Adeno-associated viruses (AAVs) are common vectors in gene therapy but can frequently cause liver complications in patients. The mechanisms underlying AAV-related liver toxicity remain poorly understood, posing challenges for effective prevention and intervention. Here we conducted a case study of a child with spinal muscular atrophy type 1 experiencing substantial hepatitis after receiving onasemnogene abeparvovec, undertaking long- and short-read metagenomic sequencing of liver tissue. We identified manufacturing plasmid sequences with complex structures and recombination. Vector genomes had extensive disruption and concatemerization as well as numerous vector–human fusion junctions. We also identified human betaherpesvirus 6B in the liver. Further work and investigation of more patients is needed to establish whether the presence of manufacturing plasmid sequences or helper viruses contribute to the formation of these complex concatemeric DNA structures in the liver, and whether these are a factor in the development of liver toxicity after AAV gene therapy.

## Abstract (web summary)

Analyses of liver biopsies from a child with spinal muscular atrophy treated with adeno-associated virus gene therapy who developed hepatitis reveal contaminating manufacturing plasmids and disrupted vector genomes, possibly resulting from recombination events.

## Main

Adeno-associated virus (AAV) gene therapies show promise for treating a variety of serious genetic conditions, including hemophilia^1–3^, muscular dystrophies^4^ and spinal muscular atrophy (SMA)^5^. As of 2025, there were seven AAV gene therapies approved by the US Food and Drug Administration^6^, with many more in clinical trials. The most common adverse effect of intravenously administered AAV gene therapies is hepatotoxicity, routinely treated with high dose steroids. Occasionally, liver toxicity is severe, and some patients have experienced fulminant liver failure^7–11^. Hepatotoxicity tends to be more severe in older patients with a higher body weight, who receive higher vector doses^12,13^.

The mechanisms underlying hepatotoxicity are incompletely understood, and it has been postulated to be caused by innate, humoral and cellular immune responses to the vector capsid, genome or transgene product^14–16^, by impurities within the vector preparation^17,18^ or from a direct toxic effect^19,20^. Acute sinusoidal endothelial injury resembling capillary leak syndrome has also been well documented in nonhuman primates using both empty capsids and therapeutic transgenes^21^.

**Fig. 1:**
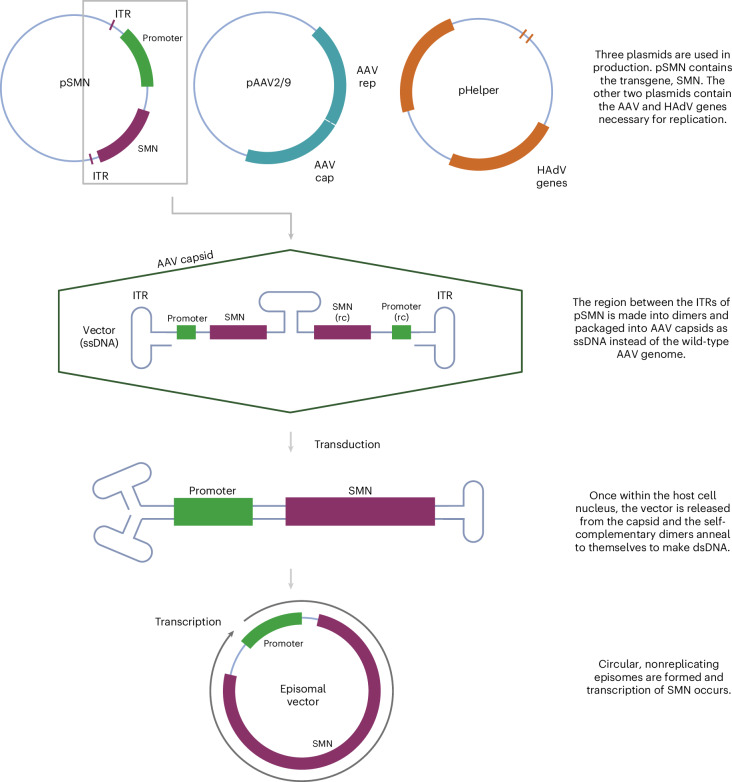
Schematic of plasmids used to manufacture OA and its mechanism of action. OA is produced by transfection of HEK293 cells with a vector plasmid (pSMN), containing *SMN* between AAV ITRs, an AAV plasmid containing AAV2 *rep* and AAV9 *cap* genes (pAAV2/9), and a helper plasmid containing HAdV genes such as *E2A*, *E4* and *VA* RNA genes (pHelper)^23,24^. SMN, survival motor neuron; HAdV, human adenovirus; ssDNA, single-stranded DNA; dsDNA, double-stranded DNA. Created with BioRender.com.

Onasemnogene abeparvovec (OA) is an AAV-vectored gene therapy for SMA, a neurodegenerative disease caused by deleterious variants in the survival motor neuron 1 (*SMN1*) gene^22^. OA is manufactured using three plasmids (Fig. 1): a vector plasmid (pSMN), which contains *SMN* and elements necessary for its expression; a packaging plasmid (pAAV2/9), which contains AAV2 replication (*rep*) and AAV9 capsid (*cap*) genes; and a helper plasmid (pHelper), which contains adenovirus (HAdV) genes necessary for AAV replication^23,24^. The resultant vector preparation contains therapeutic recombinant AAV (rAAV) particles that have an outer AAV9 capsid, containing a vector genome encoding human *SMN*. Manufacturing process-related impurities, including empty capsids, reverse-packaged plasmids, genome fragments and recombined products, are also present in rAAV preparations, even after good manufacturing practice procedures^25–27^. These manufacturing issues are complex to study and resolve, and the US Food and Drug Administration has released guidance on reporting and validating the steps in the manufacturing process^28^.

We investigated a 7-year-old female patient treated with OA for SMA type 1 (homozygous deletion of exon 7 of *SMN1*, two copies of *SMN2*), whose clinical course has been reported previously (case 2, Finnegan et al.^12^). The patient weighed >20 kg at the time of infusion, and therefore required a high total vector dose of 2.2 × 10^15^ vector genomes. The patient experienced symptomatic hepatitis, with vomiting, jaundice, abdominal pain and dark urine. Serum hepatic markers, indicating liver injury, peaked 7 weeks after infusion (Extended Data Table 1). Liver injury was managed using steroids and tacrolimus. Tacrolimus was successfully withdrawn 7 months after infusion, and steroid treatment continued for 19 months.

**Fig. 2:**
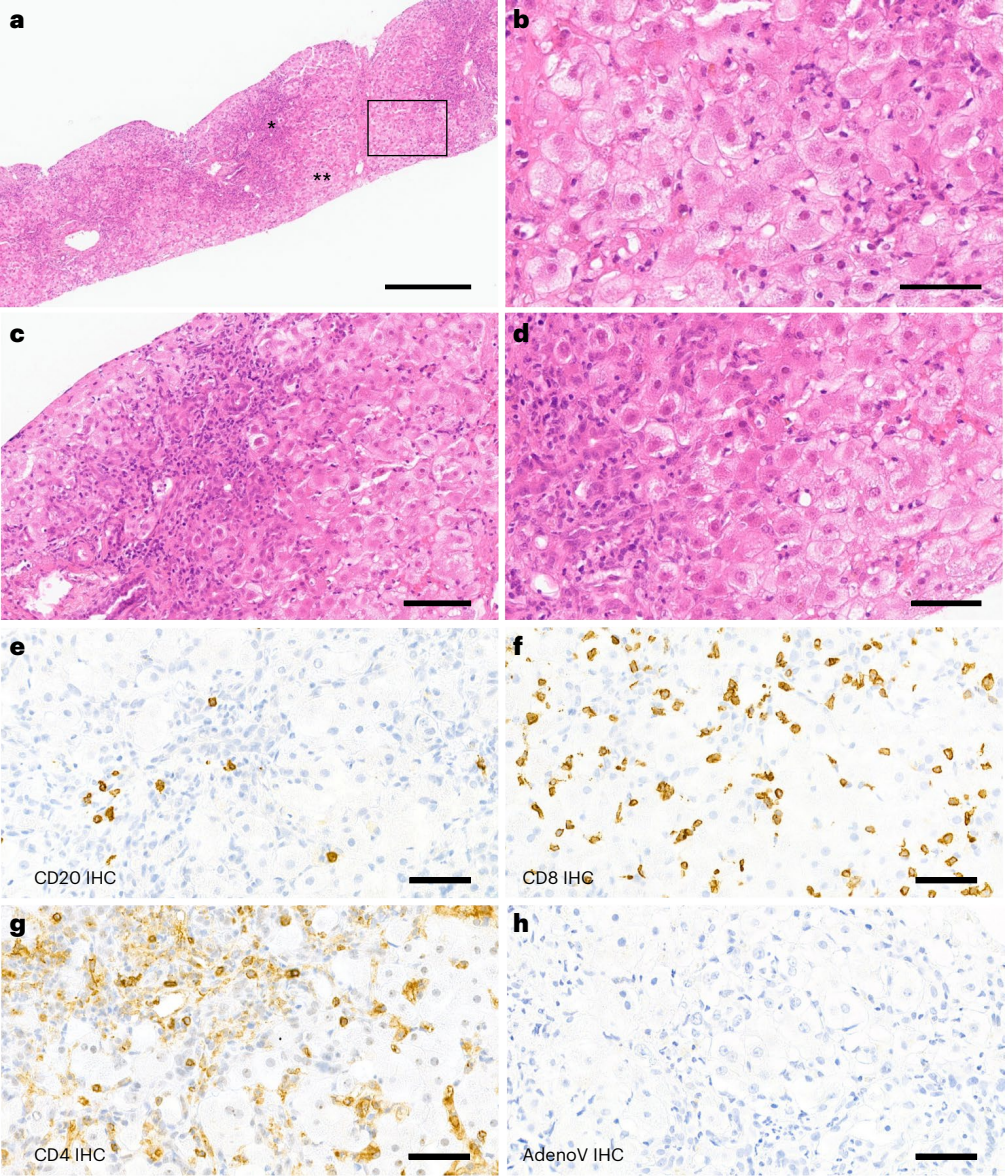
Liver biopsy findings. **a**, Liver biopsy of the patient shows marked periportal and lobular inflammation as well as interface inflammation (*); numerous hepatocytes with ballooning degeneration are present (**). **b**, High magnification of the box in **a**, with ballooning hepatocytes highlighting the swollen cytoplasm. **c**, Magnification of the ‘*’ region from **a**. **d**, Magnification of the ‘**’ region from **a**. **e**–**g**, Inflammation in the liver is shown by immunohistochemistry (IHC) detecting CD20 (**e**), CD8 (**f**) and CD4 (**g**). There was no noteworthy steatosis or spotty necrosis, and special stains did not show periportal diastase periodic acid-schiff (DPAS)-positive globules or iron deposition. **h**, Adenovirus immunostaining was negative. Scale bars, 400 µm (**a**), 60 µm (**b** and **d**), 100 µm (**c**) and 50 µm (**e**–**h**). All available tissue was stained, and representative images have been captured to illustrate the signal in the sample.

A needle core liver biopsy, taken 7 weeks after infusion, showed mild perivenular and portal fibrosis and a single focus of porto-central necrosis. There was mild portal tract expansion, including a portal ductular reaction and periductal and intraepithelial neutrophils. There was a moderate portal inflammatory infiltrate composed predominantly of CD4- and CD8-positive T lymphocytes and occasional plasma cells, with mild interface inflammation and moderate lobular inflammation with foci of hepatocellular cholestasis (Fig. 2). Few CD20-positive B lymphocytes were detected. These histological features are consistent with those previously reported in children with hepatitis associated with wild-type AAV2 infection^29,30^ and in ‘indeterminate’ pediatric acute liver failure^31^. Adenovirus immunostaining was negative (Fig. 2h).

**Fig. 3:**
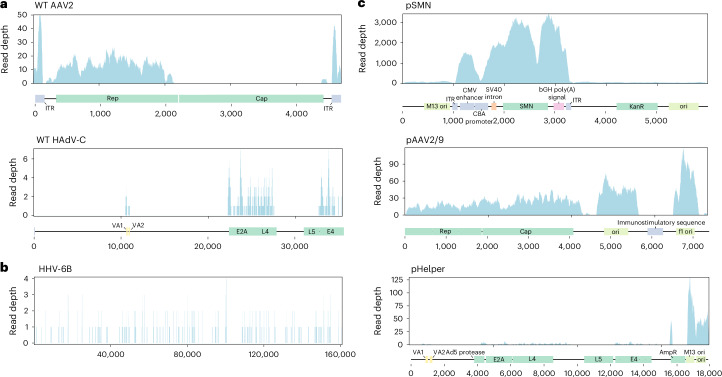
Metagenomic sequence alignment. **a**, Genome coverage of wild-type (WT) AAV2 and HAdV-C from Illumina sequencing reads. Approximate locations of the genes present in the manufacturing plasmids are marked along the *x* axis. AAV2 alignment uses more stringent mapping parameters to more clearly differentiate between any AAV2- and AAV9-derived sequences (Methods). **b**, Alignment of Illumina sequencing reads to the HHV-6B genome shows reads cover the breadth of the genome. **c**, Alignment of Illumina sequencing reads to approximate manufacturing plasmid sequences shows the presence of plasmid sequences. CMV enhancer, cytomegalovirus enhancer; SV40 intron, simian virus 40 small intron; bGH poly(A), bovine growth hormone poly(A) signal. In the negative control, ten reads aligned to the pSMN sequence, while no reads aligned to the pAAV2/9 or pHelper sequences.

We conducted untargeted short-read metagenomic sequencing of DNA and RNA from the residual patient liver sample. In the DNA sequencing analysis, the initial assignment of nonhuman reads to the most likely microbial species identified multiple serotypes of AAV, primarily AAV2, as well as human mastadenovirus C (HAdV-C) and human betaherpesvirus 6B (HHV-6B) (Extended Data Table 2). Reads assigned to HHV-6B covered the breadth of the genome (Fig. 3b) and a specific polymerase chain reaction (PCR) for HHV-6B was positive (cycle threshold (CT) 26.2), indicating natural HHV-6B infection.

The incomplete genome coverage of AAV2 and HAdV suggested that the results did not derive from a wild-type infection (Fig. 3a). To investigate this further, we aligned the reads to the manufacturing plasmid sequences used in OA production. We found good coverage of the OA vector genome as expected, but also of the pSMN plasmid backbone and of pAAV2/9, and some reads mapping to pHelper (Fig. 3c). The reads originally classified as AAV2 or HAdV-C aligned only to sections of the viral genomes that are part of the OA manufacturing plasmids (AAV2 *rep*, HAdV *E4*, *E2A*, *L4* and *VA* regions), suggesting the presence of plasmid sequences in the liver tissue rather than wild-type virus infection (Fig. 3a). A specific PCR for HAdV, targeting a region of the genome that is not present in the pHelper plasmid, was negative.

**Extended Data Fig. 1:**
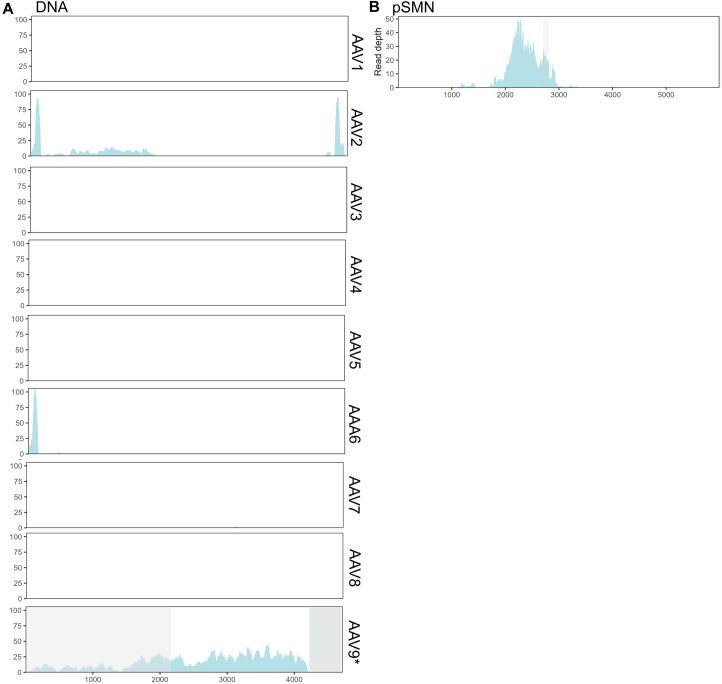
Additional alignments of metagenomics data. Alignment of short-read metagenomics data to **A**: AAV1-9 (DNA reads) and **B**: pSMN plasmid (RNA reads). Exon 7 is highlighted in grey. A full reference genome is not available for AAV9, so the sequence used is the closest full-genome BLAST match to the AAV9 *cap* sequence in the pAAV2/9 plasmid. The AAV9 *rep* sequence (shown in grey) is not verified and should be treated with caution, although in this sequence it closely resembles AAV2 *rep*. AAV accession numbers can be found in Supplementary Table 1.

The presence of the pAAV2/9 plasmid sequences also potentially explains why multiple AAV serotypes, other than AAV2, including AAV4 and AAV8, were found in our initial classification. As there is not currently a RefSeq reference sequence for AAV9, it is not included in the metagenomics database. Therefore, reads from the AAV9-derived region in pAAV2/9 (AAV9 *cap* gene) were probably misclassified as other AAV serotypes in the initial analysis. We performed an alignment of reads from the liver to AAV1–9 genomes, finding the best alignment to the *rep* gene of AAV2 and the *cap* gene of AAV9 (Extended Data Fig. 1a and Supplementary Table 1), in keeping with the chimeric structure of pAAV2/9 (AAV2 *rep* gene and AAV9 *cap* gene). Some short regions of the pAAV2/9 plasmid sequence had no aligning reads (Fig. 3c), suggesting that the sequence we used was not fully identical to the plasmid sequence used in OA manufacture, which is proprietary. Analysis of long-read metagenomic DNA data yielded similar results: initial classification identified AAVs, HAdV and HHV-6B, but subsequent alignment revealed sequences corresponding to all three manufacturing plasmids (Extended Data Table 2).

Classification and alignment of the nonhuman RNA sequencing (RNA-seq) metagenomics data detected two reads corresponding to the AAV2 *rep* gene. Four further reads showed BLAST similarity to the AAV inverted terminal repeat (ITR) region but did not align. No RNA reads corresponding to pHelper, HAdV or HHV-6B were found. Previous published work has shown that our RNA-seq metagenomics protocol is as sensitive as targeted real-time PCR^32^. The low-level AAV RNA could result from transcription of the AAV2 *rep* gene; however, this signal is below the typical reporting cutoff of the metagenomics protocol and would require further validation. RNA-seq sequence alignment confirmed the presence of RNA transcripts corresponding to the OA vector genome, including *SMN1* exon 7 (Extended Data Fig. 1b), suggesting successful expression of the therapeutic transgene.

**Fig. 4:**
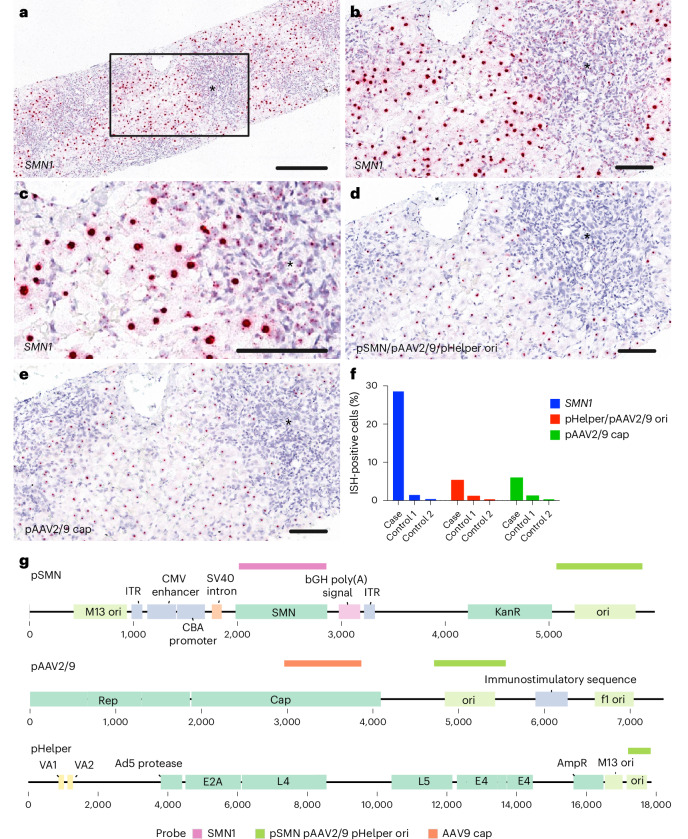
In situ hybridization. **a**–**c**, In situ hybridization (ISH) for the detection of *SMN1* in formalin-fixed paraffin-embedded (FFPE) liver tissue. **a**, A strong positive red signal was detected in the nucleus of ballooning hepatocytes separated by areas with severe immune cell infiltration (*). The box in **a** is magnified in **b**. **b**, Higher magnification of *SMN1*-positive hepatocytes next to the immune cell infiltrate (*). **c**, Dense nuclear signal for *SMN1* and a mild-to-moderate, punctuated signal within the cytoplasm of hepatocytes and immune cells (*). **d**,**e**, ISH for the detection of manufacturing plasmid sequences in FFPE liver tissue: pSMN/pAAV2/9/pHelper ori (**d**) and pAAV2/9 cap (**e**). **f**, The percentage of positive cells versus two control liver tissues (control 1, explant liver tissue from a child affected by severe hepatitis in AAV2 outbreak; control 2, healthy adult liver). **g**, Schematic showing probe binding sites on manufacturing plasmid sequences. See Extended Data Fig. 2 for controls. Scale bars, 300 µm (**a**) and 100 µm (**b**–**e**). All available tissue was stained, and representative images have been captured to illustrate the signal in the sample.

**Extended Data Fig. 2:**
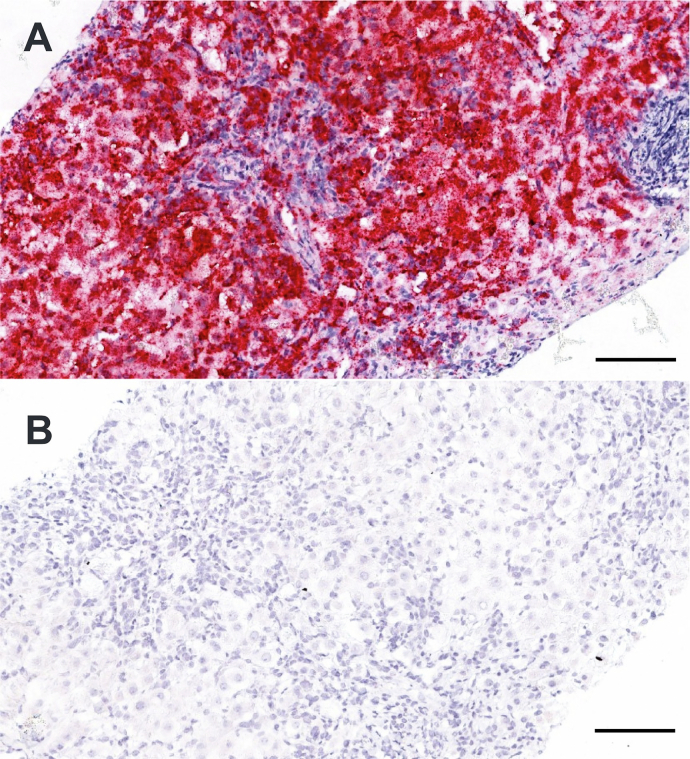
In situ hybridization controls. **A**) Abundant ubiquitin (RNA integrity, reference gene) positive signal detected. **B**) DapB (negative control, bacterial gene) no signal detected. Scale bars = 100 µm.

Next, we performed in situ hybridization to confirm the presence and location of nucleic acid sequences derived from OA. A probe for human *SMN* confirmed successful vector transduction in the patient’s liver, with 28.5% of cells in the biopsy tissue showing a positive signal (control patients showed 0.4–1.5% positive cells; Fig. 4 and Extended Data Fig. 2). We observed both nuclear and cytoplasmic positive signals. To detect plasmid sequences, we designed probes complementary to regions of the manufacturing plasmids that are absent from both the therapeutic OA vector genome and the human genome. Analysis confirmed the presence of the bacterial origin of replication in pSMN, pHelper and pAAV2/9 plasmids in 5.1% of cells (probe vector-pHelper-C1, 0.2–1.1% positive in controls), as well as a sequence from the AAV9 *cap* gene present in pAAV2/9 in 5.8% of cells (probe AAV-HeB-T1-VP1-O1-C1, 0.2–1.1% positive in controls) (Fig. 4). The contaminant plasmid-specific sequences were found at lower levels than *SMN*, in agreement with the metagenomic sequencing.

**Fig. 5:**
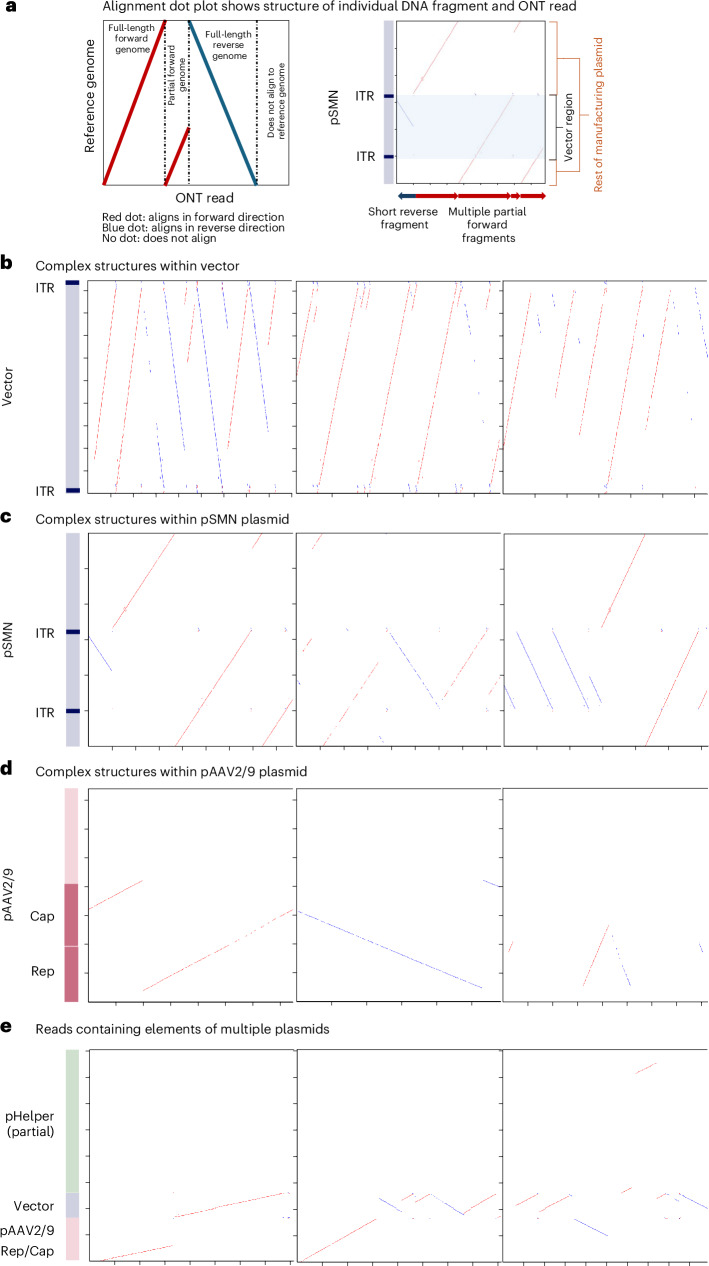
Alignment dot plots. Alignment dot plots showing individual nanopore reads (*x* axis) aligning to representative sequences of the OA manufacturing plasmids (*y* axis). Red dots indicate alignment to the forward strand, and blue dots indicate alignment to the reverse strand. **a**, Explanation of dot plot format. **b**, Alignment against the vector region of the pSMN plasmid. **c**, Alignment against the entire pSMN plasmid. **d**, Alignment to the pAAV2/9 plasmid. **e**, Alignment to regions of all three plasmids—the vector region of pSMN, AAV *rep* and *cap* within pAAV2/9 and the HAdV gene region within pHelper. Representative images were selected; the number of reads in each category can be found in Extended Data Table 3, and diagrams for all reads are provided in the Supplementary Information. See the Supplementary Information and Methods for description of similar dot plots generated for human reads.

We undertook detailed sequence analysis of individual reads from the long-read sequencing to determine the vector genome structures present in the liver. This showed high levels of vector genome concatemerization and complex genome structures with rearrangements (Fig. 5a–d and Extended Data Table 3). The concatemeric patterns observed, including head-to-head, head-to-tail and alternating repeats, showed similarities to those seen in replicating AAVs using rolling hairpin and rolling circle amplification^33^. Plasmid reads tended to not represent full-length plasmids but rather fragments of plasmid sequences in combination with the vector genome. The majority of pAAV2/9 reads also contained regions of the other manufacturing plasmids, indicating recombination between plasmids (Fig. 5 and Extended Data Table 3). Most of the complex structures and recombination events involved the vector genome, the *rep–cap* region of pAAV2/9 and the region of pHelper containing the HAdV-derived genes (Fig. 5 and Extended Data Table 3).

Rearranged sequences may derive from recombined plasmid contaminants outside vector particles, mispackaged recombined DNA from manufacture and/or recombination events after infusion. Many of the structures we observed were longer than the maximum packaging length of an AAV vector (up to 15 kb, while the packaging limit is approximately 5 kb (refs. ^34,35^)). Purification steps during manufacture are designed to remove nonpackaged DNA, and efficiency of uptake of any remaining DNA is likely to be low, suggesting that some recombination may have occurred in vivo, as previously described in nonhuman primate liver^36^.

**Extended Data Fig. 3:**
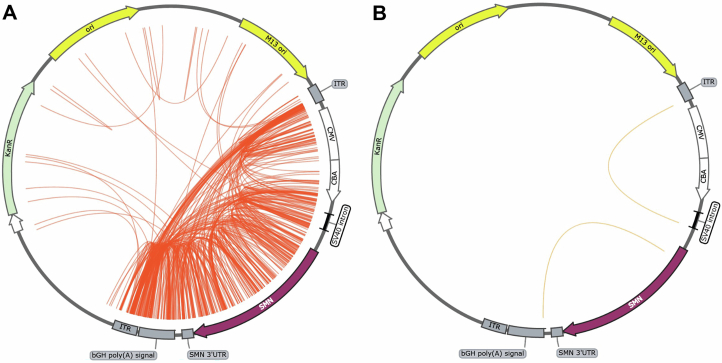
Internal vector rearrangements within pSMN plasmid. Circular representation of internal vector rearrangements for **A**) DNA and **B**) RNA reads. Genomic tracks were drawn using pyCirclize software. The vector reference map was created using SnapGene software.

We also identified numerous internal vector rearrangements at the DNA level from the short-read metagenomics. First, chimeric reads were identified, signifying read-through transcripts and noncanonical splice fusions at both DNA and RNA levels (Extended Data Fig. 3a). Mapping the reads to the vector plasmids revealed that most occurred between the AAV2 ITRs, with further junction points identified between the plasmid backbone and *SMN* transgene (Extended Data Fig. 3a). Without direct sequencing of the vector batch, we could not determine whether these rearrangements occurred during vector manufacture or within target cells, as investigated in previous studies^36^. Analysis of corresponding RNA reads showed substantially fewer chimeric transcripts, suggesting these rearranged DNA sequences generally did not produce stable transcripts (Extended Data Fig. 3).

**Extended Data Fig. 4:**
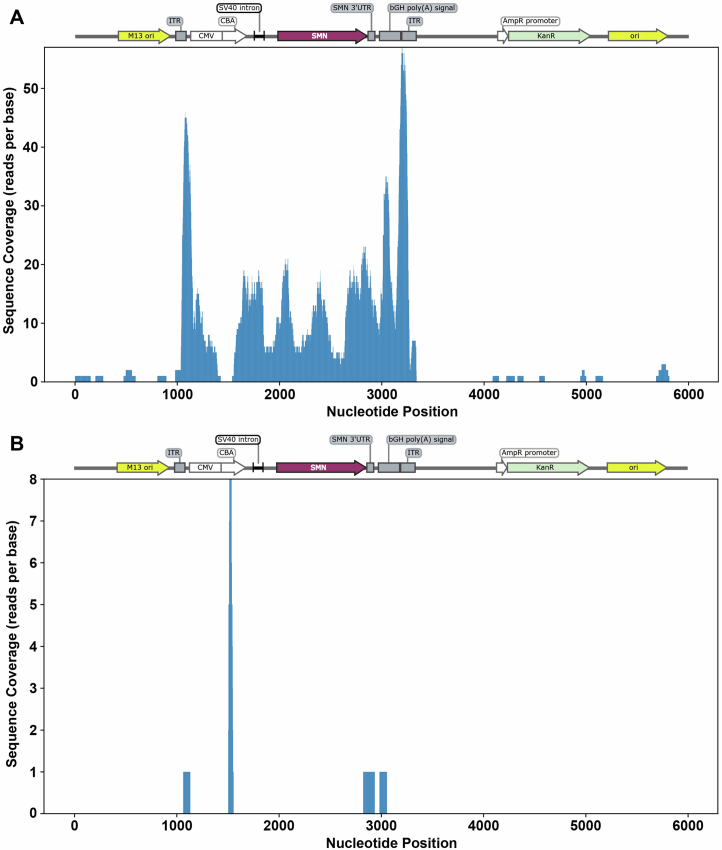
Mapping of human-AAV chimeric reads against the vector plasmid. **A**) DNA and **B**) RNA chimeric read alignment showing sequence coverage plotted as the frequency each nucleotide position appears in chimeric reads along the vector reference plasmid (x-axis). The plasmid map above each graph illustrates the corresponding functional elements. Key components include: M13 ori (bacteriophage origin of replication), ITR (inverted terminal repeat), CMV (cytomegalovirus enhancer), CBA (chicken beta-actin core promoter), SV40 intron (simian virus 40 small intron), SMN (spinal motor neuron coding sequence), SMN 3’UTR (noncoding region from endogenous SMN locus), bGH polyA (bovine growth hormone polyA signal), AmpR promoter (bacterial promoter for ampicillin resistance), KanR (kanamycin resistance marker), and ori (bacterial origin of replication).

Our study also revealed potential integration of AAV into the host genome. Analysis of chimeric DNA reads mapped to the pSMN plasmid revealed numerous vector–human junctions throughout the vector genome, including a small number of junctions in the plasmid backbone (Extended Data Fig. 4)*.* However, only a subset of these junctions appeared in chimeric RNA reads (Extended Data Fig. 4b). Notably, we detected several chimeric RNA reads in the hybrid cytomegalovirus enhancer/chicken β-actin (CBA) promoter region. Analysis of the human portions of chimeric reads mapped to the human reference genome revealed no specific fusion hotspots at either DNA or RNA levels. Chimeric DNA reads predominantly localized within gene bodies, as determined by their positions relative to annotated gene loci (Supplementary Table 3). Chimeric RNA reads were detected at lower frequencies, also primarily within transcribed gene bodies without any discernible hotspots (Supplementary Table 4).

Random, low-frequency integration of rAAV vectors in patient tissue is now well recognized^37–40^, and AAV integrants in complex concatemers containing mixtures of rearranged and truncated vector genomes have been demonstrated in the liver tissue of nonhuman primates after intravenous administration of rAAV8 vectors^36^. Chimeric reads containing plasmid sequences and non-*SMN* human DNA were also identified by the long-read sequencing, but due to the use of a ligation library preparation kit, we were unable to verify that these were not sequencing artifacts. AAV vectors are expected to persist episomally in postmitotic cells, and therefore it is plausible that vectors and associated contaminating sequences are maintained even without integration.

In conclusion, our metagenomic sequencing approaches, together with in situ hybridization, provide evidence that sequences from all three manufacturing plasmids were present in the liver of a patient with severe hepatitis after treatment with OA, 7 weeks after infusion. Long-read sequencing also revealed extensive disruption and concatemerization of vector genomes and manufacturing plasmids, with evidence of recombination events. Complex structural rearrangements and concatemers of AAV vector genomes have previously been demonstrated in macaque liver after treatment with rAAVs^36,41^ and in human hepatocytes in a humanized mouse model^42^. Similar complex concatemeric structures have also been noted in liver samples from children with hepatitis associated with wild-type AAV2 infection^29^. It will be important to ascertain whether these genomic structures are also present in rAAV-treated patients without hepatitis.

The relevance of our finding of HHV-6B in the liver is unclear in this single case description. Although it is noteworthy that HHV-6 can act as a helper virus in wild-type AAV2 replication, we detected no HHV-6 RNA, suggesting no active viral replication at the time of biopsy. HHV-6 has also been found in liver tissue in a proportion of children with hepatitis associated with wild-type AAV2 infection, although also sometimes in controls^29,30^, and has been found in children with acute liver failure of unknown cause^43,44^.

The mechanism by which complex rAAV-derived genome structures are produced, and whether they arise solely during manufacture or within transduced liver cells, remains unclear. Unfortunately, we have been unable to the access the OA batch used to infuse this patient, and there is no obligation for it to be retained by the regulators. We postulate that presence of certain manufacturing plasmid sequences (such as AAV *rep* gene and HAdV helper regions) and/or helper viruses (such as HHV-6) could enable amplification of the vector genome within cells if expressed, giving rise to the complex concatemeric structures we observed. Formation of replication-competent rAAV particles due to nonhomologous recombination in the course of vector production has been described^45^. Alternatively, these large DNA concatemers may arise purely from ITR-driven intermolecular recombination of transduced rearranged vector genomes^46,47^.

Future work is needed to determine the frequency and pathological consequences of complex DNA structures in patient liver cells after rAAV gene therapy, whether they are episomal or integrated into the host genome, the putative role of contaminating plasmid sequences and their potential toxicity and/or immunogenicity, and how together these factors may relate to the hepatotoxicity of rAAV gene therapies. This may inform both the management of patients receiving gene therapies and the manufacture of rAAV vectors.

## Methods

### Ethics

The liver biopsy procedure was performed for diagnostic purposes. Liver biopsy was obtained under general anesthesia by the percutaneous route using a liver biopsy gun under ultrasound guidance. The biopsy was nontargeted from the right lobe and contained a 3–4-cm-long core of liver tissue. Written informed consent was obtained from the child’s parent for residual biopsy material to be analyzed in this study, with additional consent for research conducted under the International Severe Acute Respiratory and Emerging Infection Consortium (ISARIC) World Health Organization (WHO) Clinical Characterization Protocol UK (CCP-UK) (ISRCTN 66726260). Ethical approval for the ISARIC CCP-UK study was given by the South Central–Oxford Research Ethics Committee in England (13/SC/0149), the Scotland A Research Ethics Committee (20/SS/0028) and the WHO Ethics Review Committee (RPC571 and RPC572).

### Short-read metagenomic sequencing

Untargeted Illumina metagenomic sequencing of the liver biopsy was carried out by the clinical metagenomics service at Great Ormond Street Hospital, according to the protocol previously described^29,32^. This is a clinical diagnostic virology laboratory and does not routinely work with plasmids, reducing the probability of contamination. A total of 44.1 million paired-end reads were obtained for DNA and 42.4 million for RNA. A negative control sample consisting of human DNA and RNA spiked with positive controls (cowpox DNA, and feline calicivirus and *Escherichia* phage MS2 RNA) was run in parallel, producing 44.3 and 46.3 million reads for DNA and RNA, respectively. Viruses were identified from the metagenomics data using Kraken2^48^ and Bracken^49^ run through nf-core’s nextflow pipeline Taxprofiler^50^ with short-read quality control and host removal using hg38 enabled, as well as metaMix^51^ with the preprocessing pipeline previously described in ref. ^29^. A custom database based on all the complete bacterial, viral, fungal and protozoa genomes in RefSeq as of June 2023^52^ was used for analysis.

Human-filtered reads from the metaMix pipeline (other than for alignment to pSMN, where raw reads were used) were aligned using Bowtie2^53^ in very sensitive mode (apart from wild-type AAV2, where the parameters -score-min L,0,-0.1 -N 0 -L 22--mp 6,2--rdg 5,3--rfg 5,3 were used to provide more stringent mapping and help distinguish between the AAV2 and AAV9 *cap* sequences) to genome sequences of AAV2 (NC_001401), HHV-6B (NC_000898) and HAdV-C (NC_001405) obtained from RefSeq, as well as representative sequences of the plasmids used in OA manufacture (pSMN^54^, pAAV2/9^55^, pHelper (pHGTI-Adeno1)^56^. A multi-fasta reference sequence consisting of AAV1–9 was also used (Supplementary Table 1). The sequence of the AMR gene region in the pSMN plasmid from the patent sequence did not match what was observed in the patient. This region was reconstructed using the long-read sequencing data, and it displayed over 99% similarity to publicly available KanR sequences (such as the KanR region of MH450172.1), suggesting that the AMR gene used in OA manufacture differs to the one in the relevant patent. The modified pSMN sequence was used in all alignments. PCR duplicates were removed from the resulting alignments using samtools markdup^57^, and alignments were plotted using a custom R script using tidyverse functions.

### Long-read metagenomic sequencing

DNA from approximately 3 mg of liver was purified using the Qiagen DNeasy Blood & Tissue kit as per the manufacturer’s instructions. DNA was fragmented to an average size of 10 kb using a Megaruptor 3 (Diagenode) to reach an optimal molar concentration for library preparation. Quality control was performed using a Femto Pulse System (Agilent Technologies) and a Qubit fluorometer (Invitrogen). Samples were prepared for nanopore sequencing using the ligation sequencing kit SQK-LSK110. DNA was sequenced on a PromethION using R9.4.1 flowcells (Oxford Nanopore Technologies, ONT). Samples were run for 72 h, resulting in 14.1 million reads and 82.5 Gb with an N50 of 9,624 bp and a mean read quality score of 13.5. All library preparation and sequencing were performed by the UCL Long Read Sequencing facility.

Reads were trimmed using porechop^58^ with an adaptor threshold of 85 and were mapped to the human genome (ensemble GRCh38 v107) using minimap2^59^ in map-ont mode. Unaligned reads were then aligned to the regions of the plasmids shown in the figures using minimap2, and the aligned reads were extracted using samtools^57^. A custom R script was used to filter reads that were over 1,000 bp in length, had a total alignment length of at least 80% of the total read length across all alignments and had a continuous stretch of matches/mismatches with no insertions or deletions of at least 100 bp. Alignment dot plots for these reads were created using redotable^60^ with a window size of 20. Representative examples are shown in the figures. Viruses were identified from the metagenomics data using Kraken2 and Bracken run through nf-core Taxprofiler^50^, with host removal with hg38 enabled.

### Validation of alignment dot plots

To confirm that the concatemeric structures identified were not sequencing artifacts, we repeated the analysis using alignment to human genes other than *SMN1*. All the ONT reads were aligned to the whole human genome, and reads aligning to the GTF2H2 and ACTB genes were extracted. GTF2H2 was chosen because it is located close to endogenous *SMN1* in the 5q13 region, and ACTB was chosen as a housekeeping gene on a different chromosome (Chr7). No evidence of complex concatemeric structures was found for these reads (Supplementary Fig. 2). Some duplex reads were identified, perhaps reflecting an ONT artifact where the complementary strand is sometimes sequenced directly after its pair. However, such duplex reads were excluded from the complex reads category in Extended Data Table 3 because they could result from the self-complementary vector (Supplementary Fig. 2 and Supplementary Table 2). There were also some reads that did not align completely to the targeted genes and surrounding regions, but instead partially aligned to another region of the human genome, usually on a different chromosome (Supplementary Fig. 2 and Supplementary Table 2). These could represent random ligation artifacts. However, both the frequency of these reads and the degree of concatemerization were much lower than those observed in the vector reads. Furthermore, in datasets that primarily consist of human reads, the probability of a ligation artifact arising between two human reads is likely to be much higher than the same between two vector or manufacturing plasmid reads, meaning that the human–human concatemers are more likely to have occurred by chance.

### Chimeric read analysis of short-read metagenomics data

#### Processing of reads

Raw paired-end sequencing data were processed using fastp v0.23.2^61^ for quality control and adapter removal. Read pairs were trimmed with a quality threshold of 20 (Phred score) and minimum length requirement of 50 bases. Adapter sequences and poly-G artifacts were automatically detected and removed using the paired-end detection algorithm. Overlapping paired-end reads were merged using PEAR v0.9.11^62^.

### Mapping to custom reference genome

Chimeric reads were identified using STAR aligner^63^. A custom reference genome was prepared by adding the vector plasmid sequence (pSMN) as an additional chromosome to the human reference genome (hg38). This approach allowed simultaneous mapping to both the human genome and the vector sequence, facilitating the identification of vector–genome junctions. The STAR aligner index was generated using this modified reference with default parameters and four processing threads. The alignment was performed against the custom reference genome with minimum chimeric segment length of 12 nucleotides, minimum overhang for a chimeric junction of 12 nucleotides, and output of chimeric junctions and separate SAM files. Chimeric alignments were filtered with a minimum alignment score of 1, maximum score drop of 30 and score separation of 1. A maximum gap of 3 bases was allowed in chimeric segments. For spliced alignments, we specified a minimum overhang of 10 bases for splice junctions, and both mate gap and intron size were limited to 1,000,000 bases. The alignment was executed using four processing threads, and the output was generated as coordinate-sorted BAM files.

### Analysis of chimeric vector reads

Chimeric junction data from STAR aligner output were parsed into a dataframe, filtering for fusion events involving the vector of interest (pSMN) by identifying chimeric reads where one fusion partner mapped to the vector sequence and the other to a genomic location. Chimeric junction data were processed to identify their proximity to endogenous genes using a custom Python script. Genomic coordinates from chimeric junctions were matched against gene annotations from GENCODE v38^64^. For each integration site, we identified the nearest gene and calculated the distance to its boundaries using a nearest-neighbor algorithm implemented in PyRanges^65^.

### Vector coverage analysis

To evaluate read distribution and coverage patterns across the vector genome, sorted BAM files from STAR alignment were filtered using samtools (v1.15) with a BED file defining the vector regions of interest. For each sample, we generated position-specific coverage depth using the samtools depth command with the -a flag to report coverage at all positions, including those with zero coverage. Coverage profiles were visualized using a custom Python script with matplotlib. Visualizations were generated for chimeric reads that span vector–genome junctions to profile which positions in the vector genome were commonly implicated in fusions.

### Mapping of internal vector rearrangements

To visualize the internal recombination events within the vector sequence, we developed a method to generate Circos plots using the pyCirclize^66^ Python package. Chimeric junction data were filtered to isolate vector-to-vector interactions (self-links), where both ends of a chimeric read mapped to different regions of the pSMN vector. A custom BED file was used to define the vector sequence boundaries. For each sample, vector self-interactions were represented as arcs connecting the respective start and end positions within the circular vector map. The positions were aligned against a circular representation of the parental pSMN map.

### Specific pathogen PCRs

Human adenovirus (HAdV) and HHV-6 real-time PCRs were performed by the diagnostic Microbiology and Virology laboratory at Great Ormond Street Hospital, and are accredited by the UK Accreditation Service to ISO15189:2022 standards. The HAdV assay targets a 132-bp region of the HAdV hexon gene gene (forward primer: GCC ACS GTG GGG TTT CTA AAC TT, reverse primer: GCC CCA GTG GKC TTA CAT GCA CAT C, probe: TGC ACC AGA CCC GGR CTC AGG TAC TCC GA)^67^ and the HHV-6 assay targets a 74-bp region of the HHV-6 DNA polymerase gene (forward primer: GAA GCA GCA ATC GCA ACA CA, reverse primer: ACA ACA TGT AAC TCG GTG TAC GGT, probe: AAC CCG TGC GCC GCT CCC)^68^. Each target was multiplexed with an internal positive control targeting mouse (mus) DNA spiked into each sample during DNA purification, as described previously^69^ with detection of a noncoding sequence (forward primer: GGA CAC TAT GCC CCT CCT TAG A, reverse primer: AGC TCC AAA CTC CGT CTC TGT AA, probe: TTG GGA ACA AAA CAC CCA TGG AAG GA).

In brief, each 25-μl reaction consisted of 0.6 μM (HAdV) or 0.5 μM (HHV-6) of each primer with 0.12 μM of each mus primer, 0.4 μM (HAdV) or 0.3 μM (HHV-6) probe with 0.08 μM mus probe, and 12.5 μl Qiagen Quantifast Fast mastermix with 10 μl template DNA. PCR cycling was performed on an ABI 7500 Fast thermocycler (95 °C for 5 min followed by 45 cycles of 95 °C for 30 s and 60 °C for 30 s). Each PCR run included a no template control and a DNA-positive control for each target.

### RNAscope in situ hybridization

Formalin-fixed paraffin-embedded liver sections were cut at 2–3 µm thickness and mounted on glass slides. According to the manufacturer’s instructions, RNAscope was performed with protease treatment and simmering in target solution (product codes 322360 and 322331, ACDBio) to detect the *SMN* gene (product code 553631, ACDBio, RNAscope Probe - Hs-SMN1-CDS - *Homo sapiens* survival of motor neuron 1 telomeric (*SMN1*) transcript variant d mRNA); the plasmid bacterial origin of replication in pSMN, pAAV2/9 and pHelper (product code 1261151-C1, ACDBio, RNAscope Probe - vector-pHelper-C1); and the AAV9 *cap* gene present in pAAV2/9 (product code 1261131-C1, ACDBio, RNAscope Probe - AAV-HeB-T1-VP1-O1-C1). As a positive control, a probe detecting ubiquitin (RNAscope Positive Control Probe - Hs-UBC, product code 310041, ACDBio) was used, and as a negative control, a probe for DapB (RNAscope Negative Control Probe - DapB, product code 310043, ACDBio) was used. Hematoxylin was used as a counterstain, and slides were digitized using the Leica Aperio 8 slide scanner.

To quantify the positive cells in the liver sections, the red signal of the ISH was detected using the deconvolution, cell segmentation and FISH module of the HALO-software (version 3.6, Indicalabs). A cell was considered positive if a red signal was detected in the cytoplasm and/or nucleus. Data were visualized using GraphPad Prism software (version 10).

### Immunohistochemistry

Immunohistochemistry was performed on formalin-fixed paraffin-embedded tissue cut at a thickness of 3 µm, using the Ventana Benchmark ULTRA staining platform and Optiview DAB Detection kit, with a hematoxylin counterstain.

For CD4, CD8 and CD20, the positive control was tonsil. The following antibodies were used after heat-induced epitope removal (HIER) pretreatment: anti-CD4 (clone SP35, Roche, 790-4423), anti-CD8 (clone SP239, Roche, 790-7176) and anti-CD20 (clone L26, Dako (Agilent), M0755).

For adenovirus, the positive control was a known HAdV-positive gastrointestinal surgical case. A proteolytic-induced epitope removal (PIER) pretreatment with protease 1 for 4 min was used. Antibody incubation was carried out for 32 min (AdV clone 2/6 and 20/11, Roche, 760-4870, prediluted).

### Statistics and reproducibility

This was a single case study, so no statistical analysis was performed.

### Reporting summary

Further information on research design is available in the Nature Portfolio Reporting Summary linked to this article.

## Online content

Any methods, additional references, Nature Portfolio reporting summaries, source data, extended data, supplementary information, acknowledgements, peer review information; details of author contributions and competing interests; and statements of data and code availability are available at 10.1038/s41591-025-04073-z.

## Supplementary information

Supplementary Information Supplementary Tables 1–4 and Figs. 1 and 2.

Reporting Summary

## Figures and Tables

**Extended Data Table 1:**
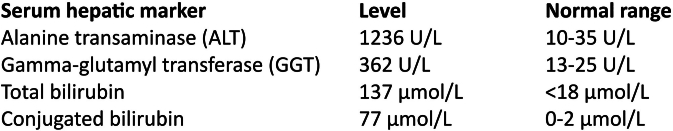
Levels of serum hepatic markers at seven weeks post infusion

**Extended Data Table 2:**
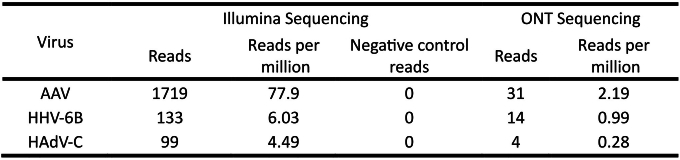
Metagenomic sequencing results (DNA only)

**Extended Data Table 3:**
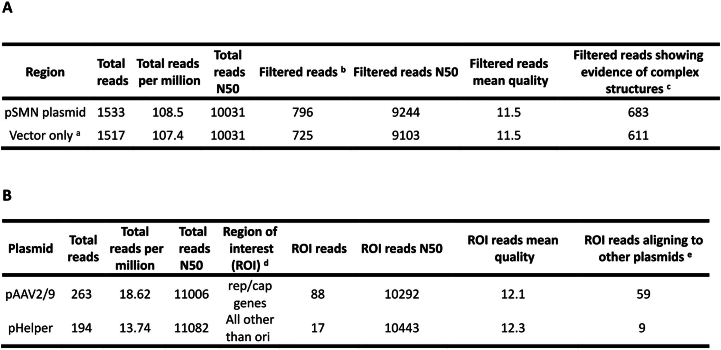
Gene therapy manufacturing plasmids in nanopore sequencing

^a^Vector only: gene therapy vector - region between the ITRs in the pSMN plasmid.

^b^Reads are filtered based the following requirements: at least 1000 bp in length, 80% of the read aligns to the plasmid across all distinct alignments, and has a continuous stretch of matches/mismatches without insertions or deletions of at least 100 bp. This filtering helps remove reads that do not align convincingly to the plasmid sequence but allows for complexity in alignments due to concatemeric structures.

^c^Complex structures were identified using alignment dot plots. Monomeric structures and duplexes which might have arisen due to self-complementary vectors were excluded.

^d^Regions of interest contain the most relevant genes for AAV replication – AAV *rep* and *cap* in the case of pAAV2/9 and the adenovirus genes in the case of pHelper. These regions also exclude regions such as the origin of replication, which may be shared between plasmids.

^e^As determined by manual inspection of alignment dot plots.

## Data Availability

The full sequencing datasets cannot be shared because of their human genetic content, which could allow the patient to be identified. Human-filtered datasets are available from the corresponding author within 30 days of request.

## Code availability

Code used for analysis in this study is available via GitHub at https://github.com/sarah-buddle/aav-gene-therapy.
