# Supplementary material for: Contaminating plasmid sequences and disrupted vector genomes in the liver following adeno-associated virus gene therapy
Source: Nat Med. 2026 Jan 16;32(2):472–80. doi: 10.1038/s41591-025-04073-z (PMC12920116; doi:10.1038/s41591-025-04073-z)

nature medicine

Supplementary information https://doi.org/10.1038/s41591-025-04073-z

Contaminating plasmid sequences and disrupted vector genomes in the liver following adeno-associated virus gene therapy

In the format provided by the authors and unedited

ISARIC4C Investigators - Consortium List

J. Kenneth Baillie, Malcolm G. Semple, Gail Carson, Peter J. M. Openshaw, Jake Dunning, Laura Merson, Clark D. Russell, David Dorward, Maria Zambon, Meera Chand, Richard S. Tedder, Saye Khoo, Lance C. W. Turtle, Tom Solomon, Samreen Ijaz, Tom Fletcher, Massimo Palmarini, Antonia Ho, Emma C. Thomson, Nicholas Price, Judith Breuer, Thushan de Silva, Chloe Donohue, Hayley Hardwick, Wilna Oosthuyzen, Miranda Odam, Primrose Chikowore, Lauren Obosi, Sara Clohisey, Andrew Law, Lucy Norris, Sarah Tait, Murray Wham, Richard Clark, Audrey Coutts, Lorna Donnelly, Angie Fawkes, Tammy Gilchrist, Katarzyna Hafezi, Louise MacGillivray, Alan Maclean, Sarah McCafferty, Kirstie Morrice, Lee Murphy, Nicola Wrobel, Sarah E. McDonald, Victoria Shaw, Katie A. Ahmed, Jane A. Armstrong, Lauren Lett, Paul Henderson, Louisa Pollock, Shyla Kishore, Helen Brotherton, Lawrence Armstrong, Andrew Mitra, Anna Dall, Kristyna Bohmova, Sheena Logan, Louise Gannon, Ken Agwuh, Srikanth Chukkambotla, Ingrid DuRand, Duncan Fullerton, Sanjeev Gar, Clive Graham, Tassos Grammatikopoulos, Stuart Hartshorn, Luke Hodgson, Paul Jennings, George Koshy, Tamas Leiner, James Limb, Jeff Little, Sheena Logan, Elijah Matovu, Fiona McGill, Craig Morris, John Morrice, David Price, Henrik Reschreiter, Tim Reynolds, Paul Whittaker, Thomas Jordan, Rachel Tayler, Clare Irving, Katherine Jack, Maxine Ramsay, Margaret Millar, Barry Milligan, Naomi Hickey, Maggie Connon, Catriona Ward, Laura Beveridge, Susan MacFarlane, Karen Leitch, Claire Bell, Lauren Finlayson, Joy Dawson, Janie Candlish, Laura McGenily, Tara Roome, Cynthia Diaba, Jasmine Player, Natassia Powell, Ruth Howman, Sara Burling, Sharon Floyd, Sarah Farmer, Susie Ferguson, Susan Hope, Lucy Rubick, Rachel Swingler, Emma Collins, Collette Spencer, Amaryl Jones, Barbara Wilson, Diane Armstrong, Mark Birt, Holly Dickinson, Rosemary Harper, Darran Martin, Amy Roff, Sarah Mills, Quen Mok, Shannon McCarty, Kate Plant, Emily Beech & Lauran O’Neill

Supplementary Tables

Supplementary Table 1: AAV RefSeq/GenBank IDs

AAV	RefSeq/GenBank ID	Notes
AAV1	NC_002077	
AAV2	NC_001401	
AAV3	NC_001729	
AAV4	NC_001829	
AAV5	NC_006152	
AAV6	AF028704	
AAV7	NC_006260	
AAV8	NC_006261	
AAV9*	OM451154	Not labelled as AAV9 – metagenomic assembled genome with AAV9 cap

Supplementary Table 2: Human gene alignment

Gene	Total reads	Total reads per million	Total reads N50	Monomeric reads	Duplexes/ incomplete alignments	Poor alignments
GTF2H2	173	1.96	9733	145	12	7
ACTB	183	2.07	10386	167	16	0

Supplementary Table 3: Genomic loci implicated in chimeric DNA reads. The nearest gene was determined based on gene annotations from GENCODE v38.

Chromosome	Position	Strand	Nearest Gene	Distance
chr1	94956248	-	CNN3-DT	0
chr1	167112576	-	GPA33	0
chr1	186873438	+	PLA2G4A	0
chr10	69413508	-	TACR2	0
chr10	125801283	+	UROS	0
chr11	17091371	+	PIK3C2A	0
chr11	36026827	+	LDLRAD3	0
chr12	117380911	+	NOS1	0
chr13	51798165	-	RP11-147H23.3	0
chr14	72130594	+	RGS6	0
chr15	82193334	-	EFL1	0
chr16	29024066	+	RP11-231C14.10	0
chr17	78123954	-	TMC6	0
chr19	19245161	+	NCAN	0
chr4	21279759	+	KCNIP4	0
chr4	138142466	-	SLC7A11-AS1	0
chr5	61764787	-	C5orf64	0
chr5	129997874	+	CHSY3	0
chr5	147386184	+	STK32A	0
chr6	10133040	+	OFCC1	0
chr6	22304121	+	CASC15	0
chr7	29391963	+	RP5-1189D6.2	0
chr17	79705513	-	MIR4739	1663
chr13	109267576	-	RP11-54H7.4	2058
chr11	47612519	+	MTCH2	4796
chr9	43252684	-	ABBA01045074.1	7698
chr5	112699178	-	APC	8320
chr18	49514224	+	LINC02837	12365
chr6	83752879	-	RP3-393K13.1	16355
chrX	35891671	-	RP11-307A17.2	22041
chr5	35255246	+	PRLR	24760
chr5	75782409	-	SLC25A5P9	28931
chr5	3316203	+	LINC01019	41061
chr6	4653855	-	KU-MEL-3	41937
chrX	39182809	+	RP11-265P11.1	43294
chr7	128139704	-	SND1	47096
chr4	58206444	-	RP11-25L5.2	88375
chr1	80743764	-	LINC01781	96974
chr18	40483912	-	RP11-10C23.1	236546
chr6	95251541	+	CYCSP17	252641
chr15	10470498	-	RP11-79C23.1	9408057

Supplementary Table 4: Genomic loci implicated in chimeric RNA reads. The nearest gene was determined based on gene annotations from GENCODE v38

Chromosome	Position	Strand	Nearest Gene	Distance
chr3	54146129	+	CACNA2D3	0
chr4	117622593	-	LINC01378	0
chr5	112284524	-	EPB41L4A	0
chr6	23839408	+	RP11-440J4.2	14731
chr6	23839408	+	RP11-440J4.2	14731
chr7	16348649	+	CRPPA	0
chr9	106547017	+	LINC01505	0
chr10	63302241	+	JMJD1C	0
chr11	94102273	-	HEPHL1	0
chr13	38080477	-	LINC00571	0

Supplementary Figures

Supplementary Figure 1: Alignment dot plots for OA manufacturing plasmids

See end of document

Supplementary Figure 2: Alignment dot plots for selected human genes

See end of document

Figure S1

pSMN plasmid (y axis)
Complex reads (unfiltered)

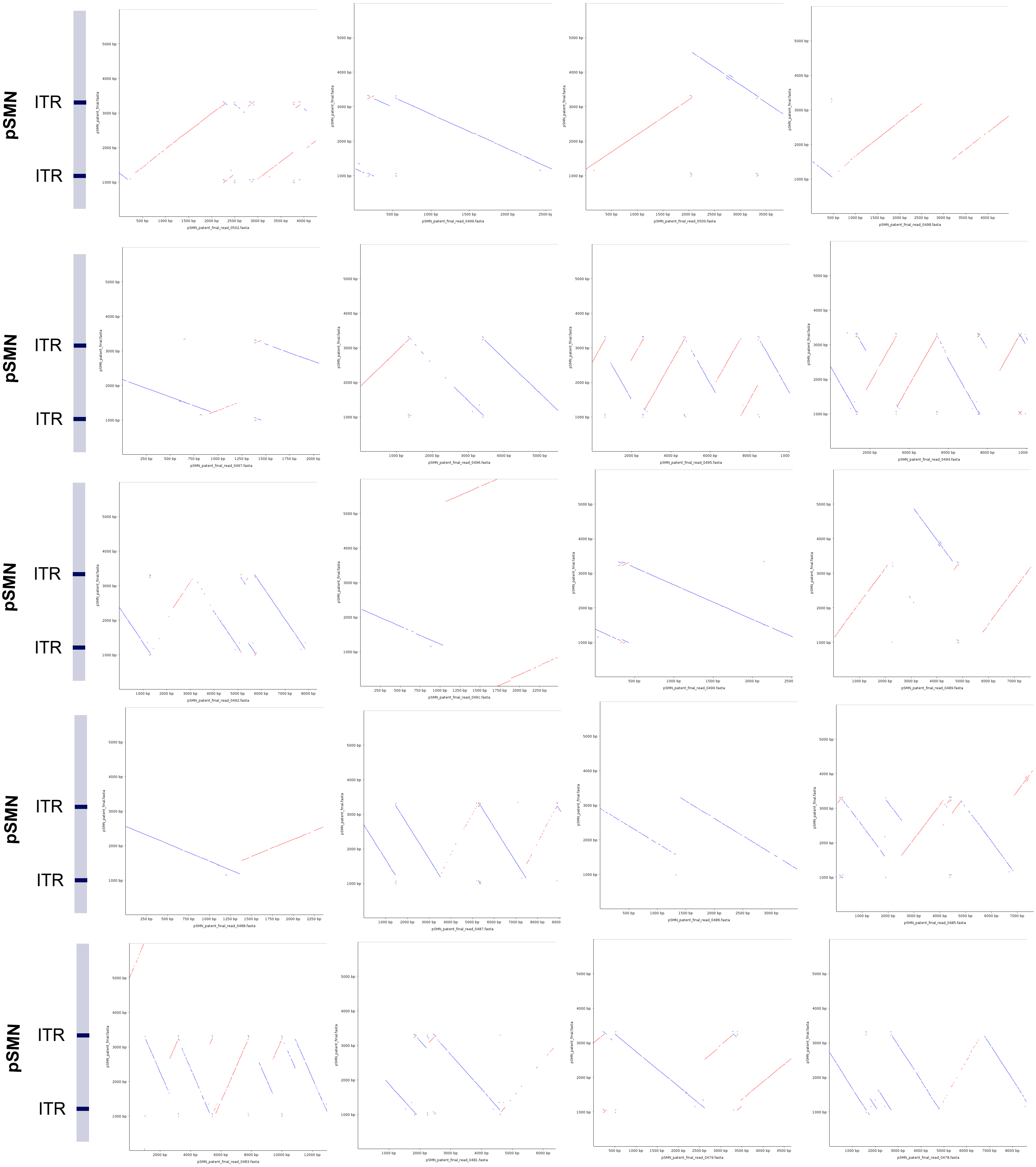

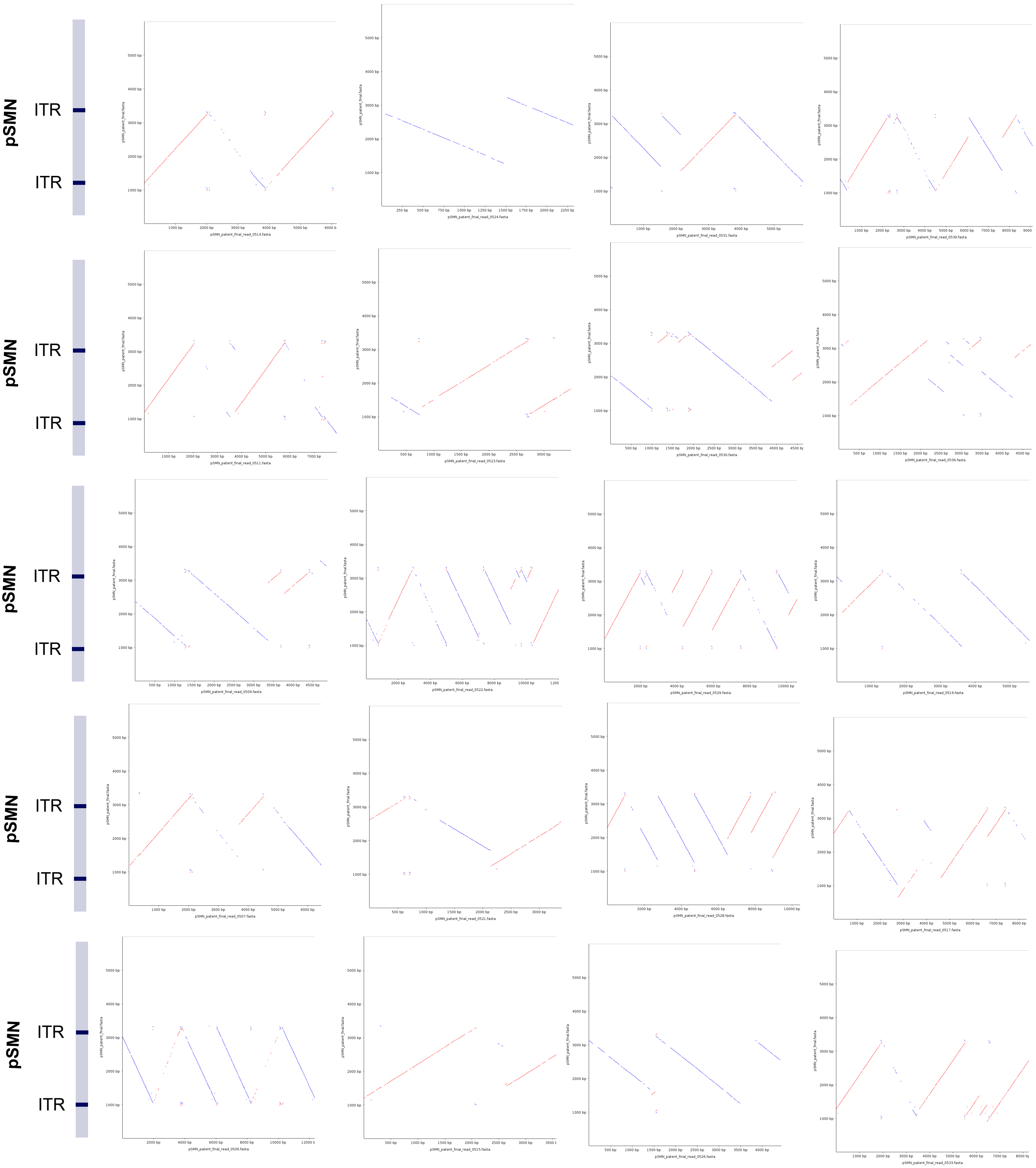
pSMN
ITR
ITR
pSMN
ITR
ITR
pSMN
ITR
ITR
pSMN
ITR
ITR
pSMN
ITR
ITR

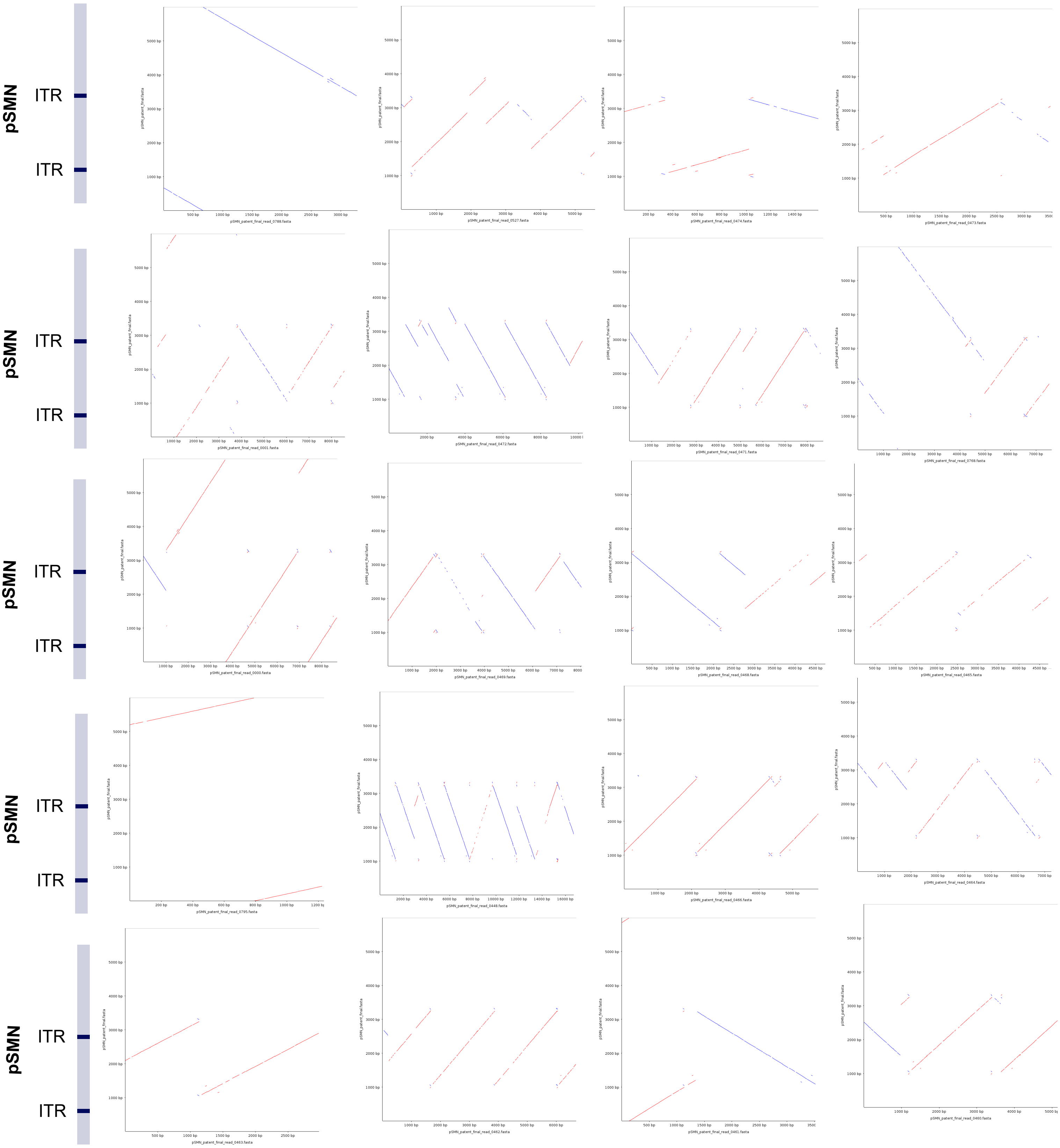
pSMN
ITR
ITR
pSMN_patent_final_read_0788.fasta
pSMN_patent_final_read_0527.fasta
pSMN_patent_final_read_0474.fasta
pSMN_patent_final_read_0473.fasta
pSMN
ITR
ITR
pSMN_patent_final_read_0001.fasta
pSMN_patent_final_read_0472.fasta
pSMN_patent_final_read_0471.fasta
pSMN_patent_final_read_0768.fasta
pSMN
ITR
ITR
pSMN_patent_final_read_0000.fasta
pSMN_patent_final_read_0469.fasta
pSMN_patent_final_read_0468.fasta
pSMN_patent_final_read_0465.fasta
pSMN
ITR
ITR
pSMN_patent_final_read_0795.fasta
pSMN_patent_final_read_0448.fasta
pSMN_patent_final_read_0466.fasta
pSMN_patent_final_read_0464.fasta
pSMN
ITR
ITR
pSMN_patent_final_read_0463.fasta
pSMN_patent_final_read_0462.fasta
pSMN_patent_final_read_0461.fasta
pSMN_patent_final_read_0460.fasta

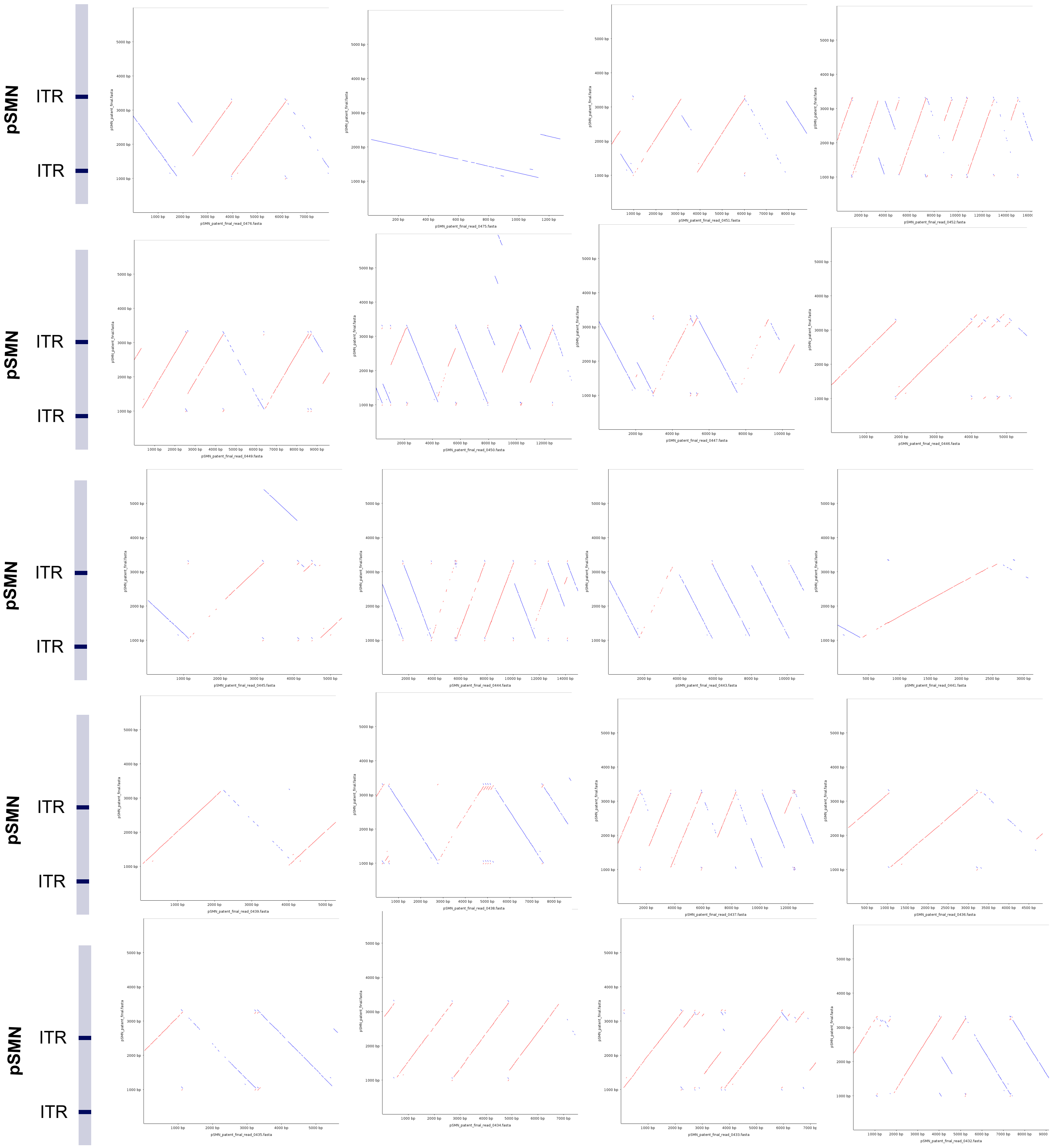

pSMN
ITR
ITR
pSMN_patent_final_read_0476.fasta
pSMN_patent_final_read_0475.fasta
pSMN_patent_final_read_0451.fasta
pSMN_patent_final_read_0452.fasta
pSMN
ITR
ITR
pSMN_patent_final_read_0449.fasta
pSMN_patent_final_read_0450.fasta
pSMN_patent_final_read_0447.fasta
pSMN_patent_final_read_0446.fasta
pSMN
ITR
ITR
pSMN_patent_final_read_0445.fasta
pSMN_patent_final_read_0444.fasta
pSMN_patent_final_read_0443.fasta
pSMN_patent_final_read_0441.fasta
pSMN
ITR
ITR
pSMN_patent_final_read_0439.fasta
pSMN_patent_final_read_0438.fasta
pSMN_patent_final_read_0437.fasta
pSMN_patent_final_read_0436.fasta
pSMN
ITR
ITR
pSMN_patent_final_read_0435.fasta
pSMN_patent_final_read_0434.fasta
pSMN_patent_final_read_0433.fasta
pSMN_patent_final_read_0432.fasta

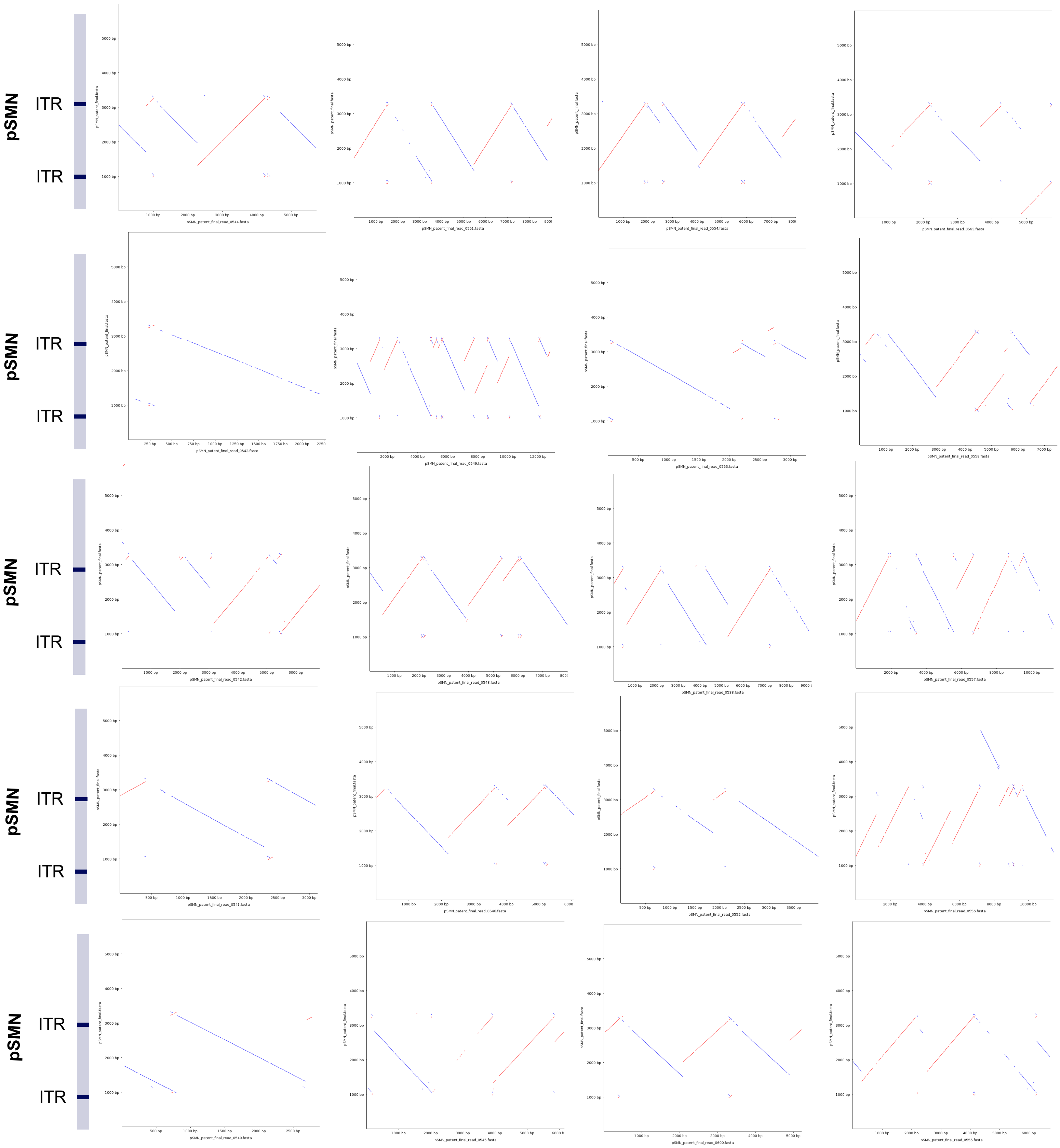
pSMN
ITR
ITR
pSMN_patent_final_read_0544.fasta
pSMN_patent_final_read_0551.fasta
pSMN_patent_final_read_0554.fasta
pSMN_patent_final_read_0563.fasta
pSMN
ITR
ITR
pSMN_patent_final_read_0543.fasta
pSMN_patent_final_read_0549.fasta
pSMN_patent_final_read_0553.fasta
pSMN_patent_final_read_0558.fasta
pSMN
ITR
ITR
pSMN_patent_final_read_0542.fasta
pSMN_patent_final_read_0548.fasta
pSMN_patent_final_read_0538.fasta
pSMN_patent_final_read_0557.fasta
pSMN
ITR
ITR
pSMN_patent_final_read_0541.fasta
pSMN_patent_final_read_0546.fasta
pSMN_patent_final_read_0552.fasta
pSMN_patent_final_read_0556.fasta
pSMN
ITR
ITR
pSMN_patent_final_read_0540.fasta
pSMN_patent_final_read_0545.fasta
pSMN_patent_final_read_0600.fasta
pSMN_patent_final_read_0555.fasta

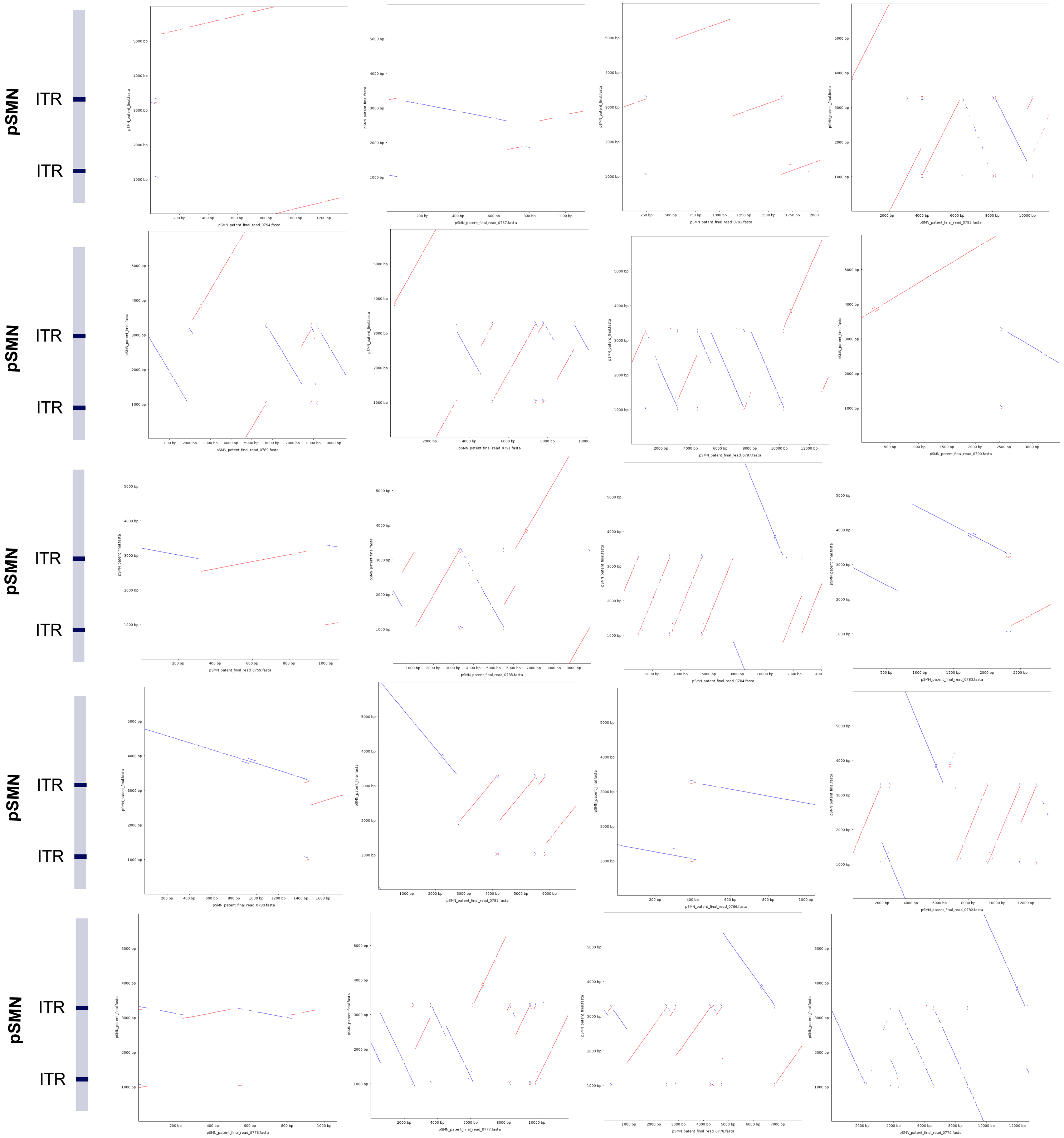
pSMN
ITR
ITR
pSMN
ITR
ITR
pSMN
ITR
ITR
pSMN
ITR
ITR
pSMN
ITR
ITR

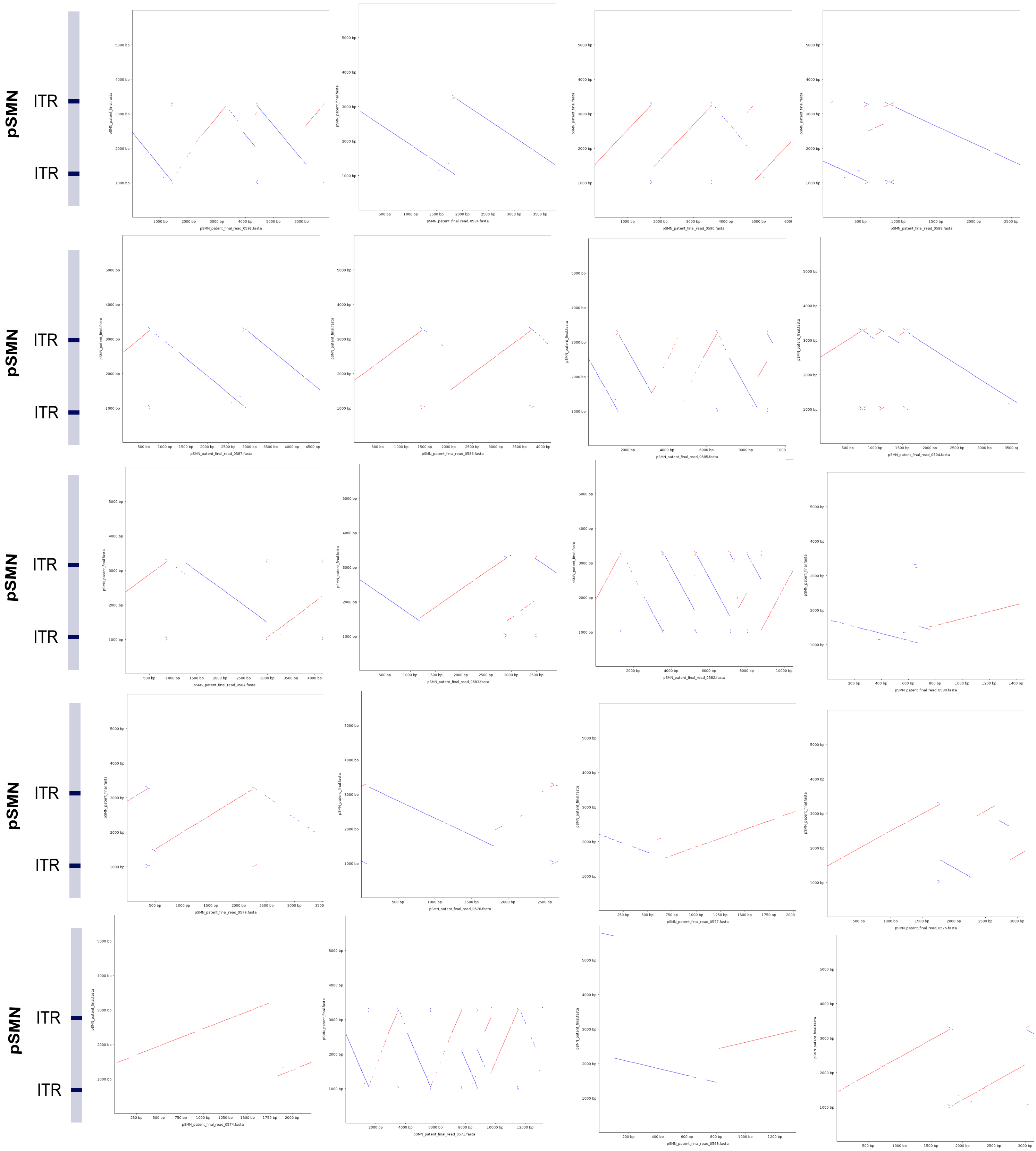

pSMN
ITR
ITR
pSMN_patent_final_read_0591.fasta
pSMN_patent_final_read_0534.fasta
pSMN_patent_final_read_0590.fasta
pSMN_patent_final_read_0588.fasta
pSMN_patent_final_read_0587.fasta
pSMN_patent_final_read_0586.fasta
pSMN_patent_final_read_0585.fasta
pSMN_patent_final_read_0504.fasta
pSMN_patent_final_read_0584.fasta
pSMN_patent_final_read_0583.fasta
pSMN_patent_final_read_0582.fasta
pSMN_patent_final_read_0580.fasta
pSMN_patent_final_read_0579.fasta
pSMN_patent_final_read_0578.fasta
pSMN_patent_final_read_0577.fasta
pSMN_patent_final_read_0575.fasta
pSMN_patent_final_read_0574.fasta
pSMN_patent_final_read_0571.fasta
pSMN_patent_final_read_0568.fasta
pSMN_patent_final_read_0567.fasta

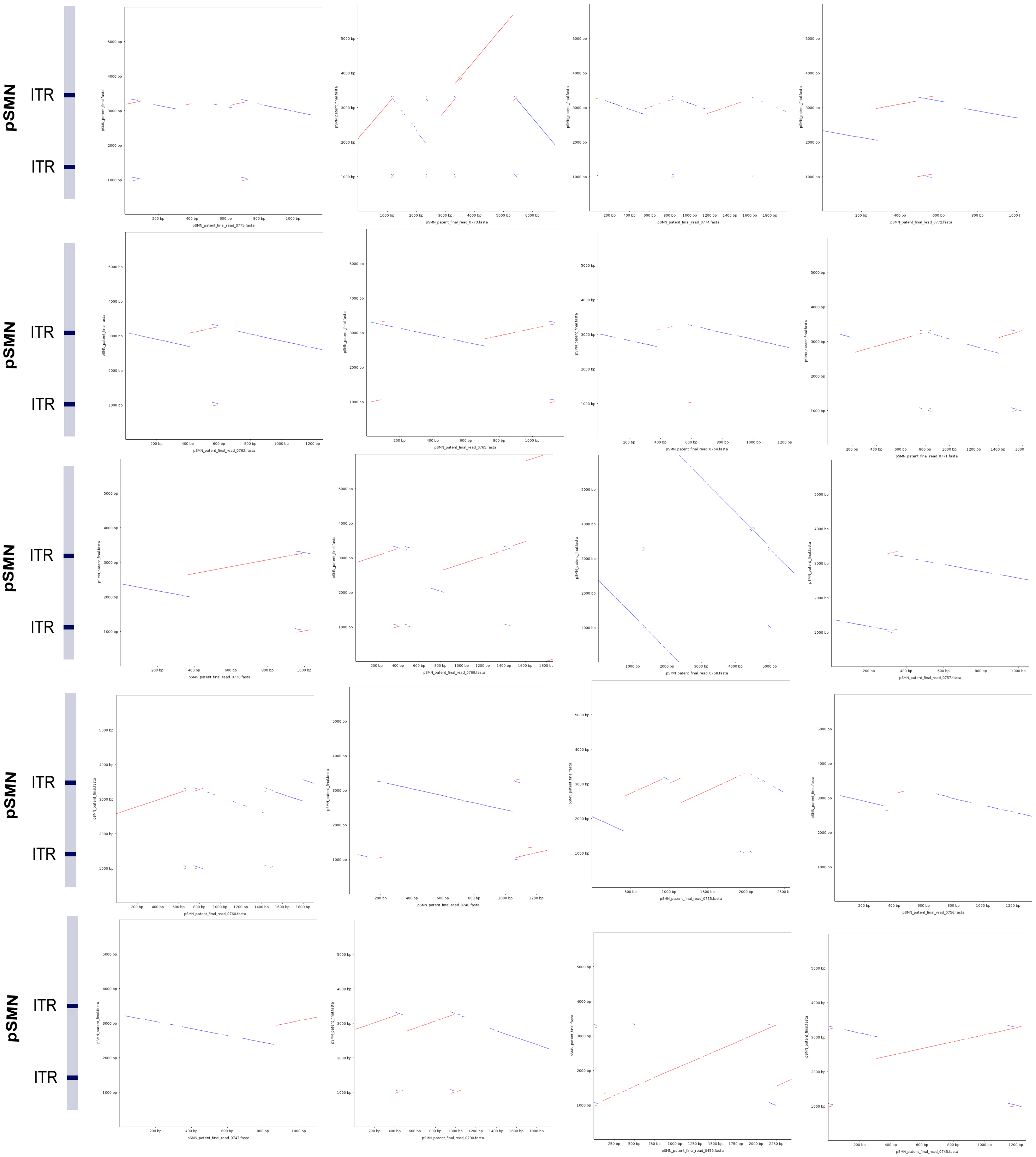
pSMN
ITR
ITR
pSMN_patent_final_read_0775.fasta
pSMN_patent_final_read_0773.fasta
pSMN_patent_final_read_0774.fasta
pSMN_patent_final_read_0772.fasta
pSMN
ITR
ITR
pSMN_patent_final_read_0762.fasta
pSMN_patent_final_read_0765.fasta
pSMN_patent_final_read_0764.fasta
pSMN_patent_final_read_0771.fasta
pSMN
ITR
ITR
pSMN_patent_final_read_0770.fasta
pSMN_patent_final_read_0769.fasta
pSMN_patent_final_read_0758.fasta
pSMN_patent_final_read_0757.fasta
pSMN
ITR
ITR
pSMN_patent_final_read_0760.fasta
pSMN_patent_final_read_0748.fasta
pSMN_patent_final_read_0755.fasta
pSMN_patent_final_read_0756.fasta
pSMN
ITR
ITR
pSMN_patent_final_read_0747.fasta
pSMN_patent_final_read_0730.fasta
pSMN_patent_final_read_0459.fasta
pSMN_patent_final_read_0745.fasta

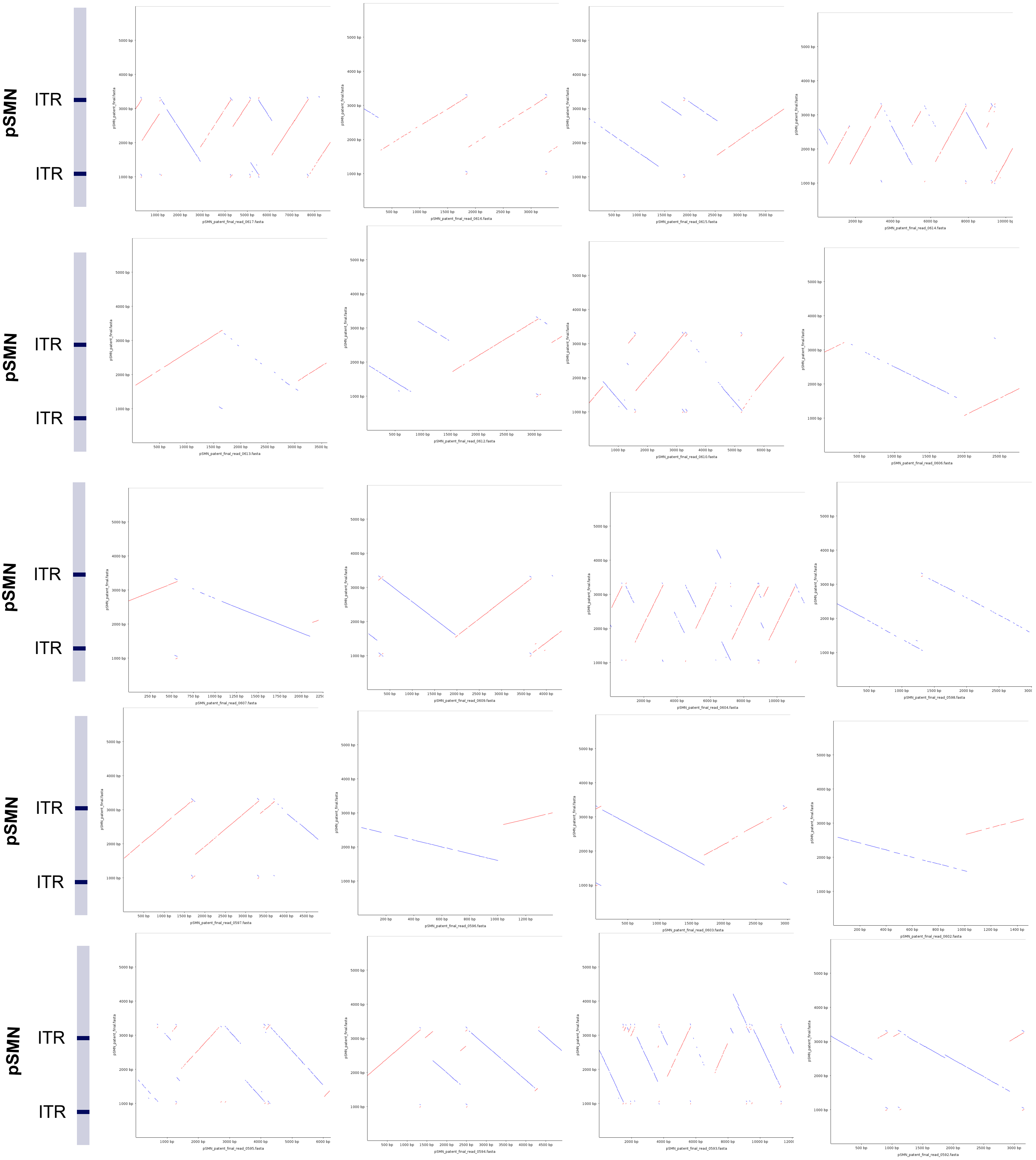
pSMN
ITR
ITR
pSMN
ITR
ITR
pSMN
ITR
ITR
pSMN
ITR
ITR
pSMN
ITR
ITR

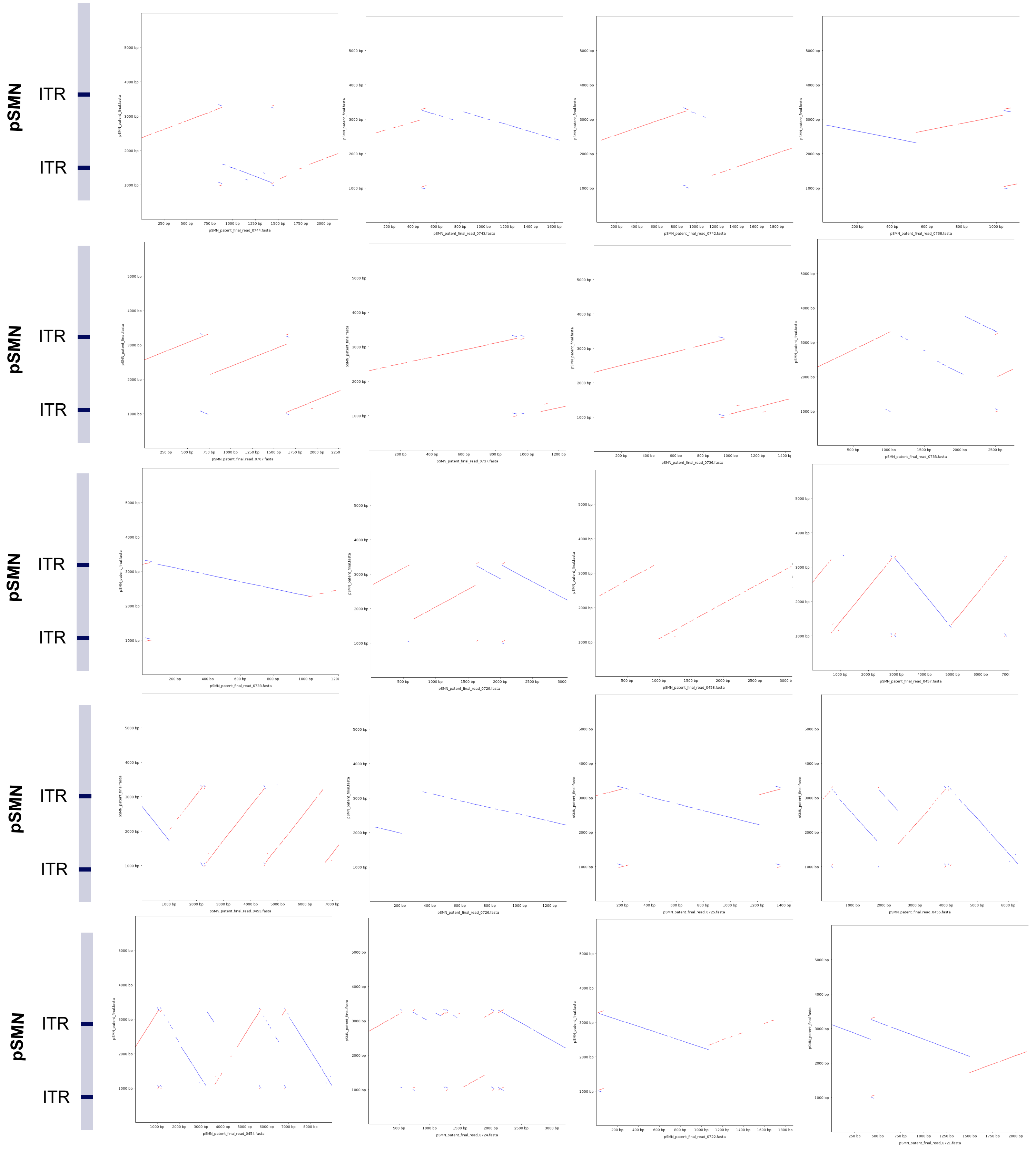
pSMN
ITR
ITR
pSMN
ITR
ITR
pSMN
ITR
ITR
pSMN
ITR
ITR
pSMN
ITR
ITR

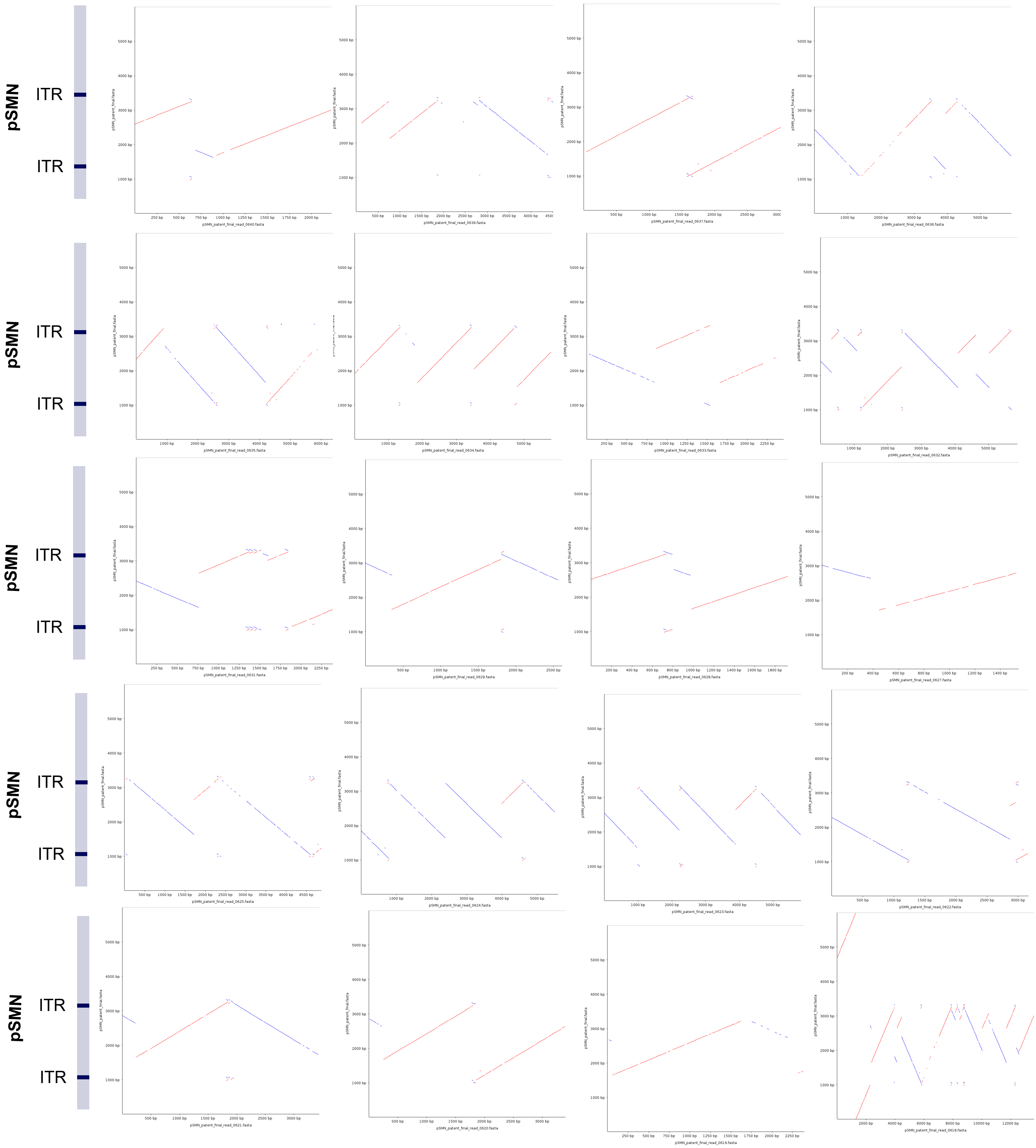
pSMN
ITR
ITR
pSMN_patent_final_read_0640.fasta
pSMN_patent_final_read_0639.fasta
pSMN_patent_final_read_0637.fasta
pSMN_patent_final_read_0636.fasta
pSMN
ITR
ITR
pSMN_patent_final_read_0635.fasta
pSMN_patent_final_read_0634.fasta
pSMN_patent_final_read_0633.fasta
pSMN_patent_final_read_0632.fasta
pSMN
ITR
ITR
pSMN_patent_final_read_0631.fasta
pSMN_patent_final_read_0629.fasta
pSMN_patent_final_read_0628.fasta
pSMN_patent_final_read_0627.fasta
pSMN
ITR
ITR
pSMN_patent_final_read_0625.fasta
pSMN_patent_final_read_0624.fasta
pSMN_patent_final_read_0623.fasta
pSMN_patent_final_read_0622.fasta
pSMN
ITR
ITR
pSMN_patent_final_read_0621.fasta
pSMN_patent_final_read_0620.fasta
pSMN_patent_final_read_0619.fasta
pSMN_patent_final_read_0618.fasta

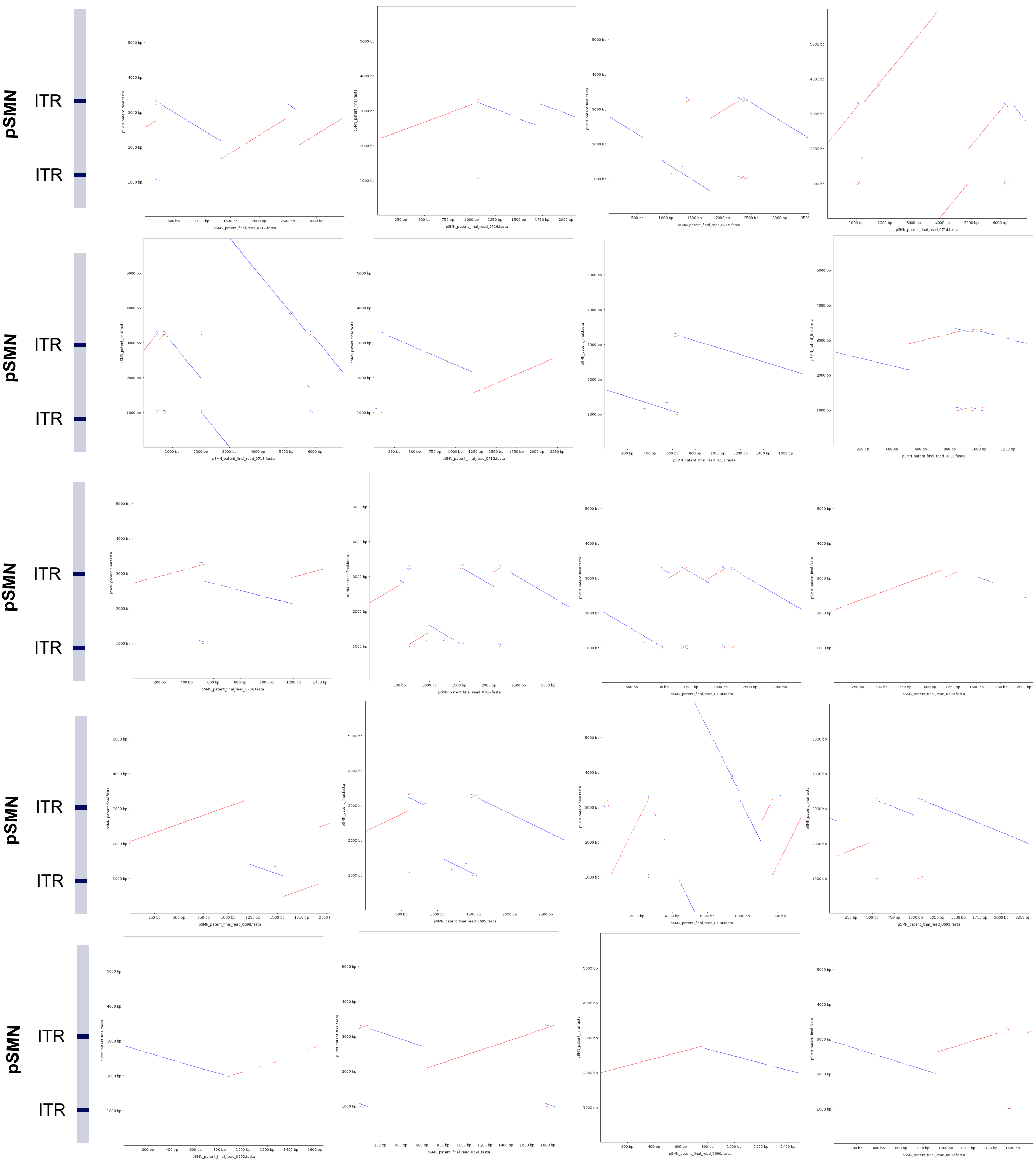
pSMN
ITR
ITR
pSMN_patent_final.fasta
pSMN_patent_final_read_0717.fasta
pSMN_patent_final_read_0716.fasta
pSMN_patent_final_read_0715.fasta
pSMN_patent_final_read_0714.fasta
pSMN
ITR
ITR
pSMN_patent_final_read_0713.fasta
pSMN_patent_final_read_0712.fasta
pSMN_patent_final_read_0711.fasta
pSMN_patent_final_read_0710.fasta
pSMN
ITR
ITR
pSMN_patent_final_read_0706.fasta
pSMN_patent_final_read_0705.fasta
pSMN_patent_final_read_0704.fasta
pSMN_patent_final_read_0700.fasta
pSMN
ITR
ITR
pSMN_patent_final_read_0698.fasta
pSMN_patent_final_read_0695.fasta
pSMN_patent_final_read_0694.fasta
pSMN_patent_final_read_0693.fasta
pSMN
ITR
ITR
pSMN_patent_final_read_0692.fasta
pSMN_patent_final_read_0691.fasta
pSMN_patent_final_read_0690.fasta
pSMN_patent_final_read_0689.fasta

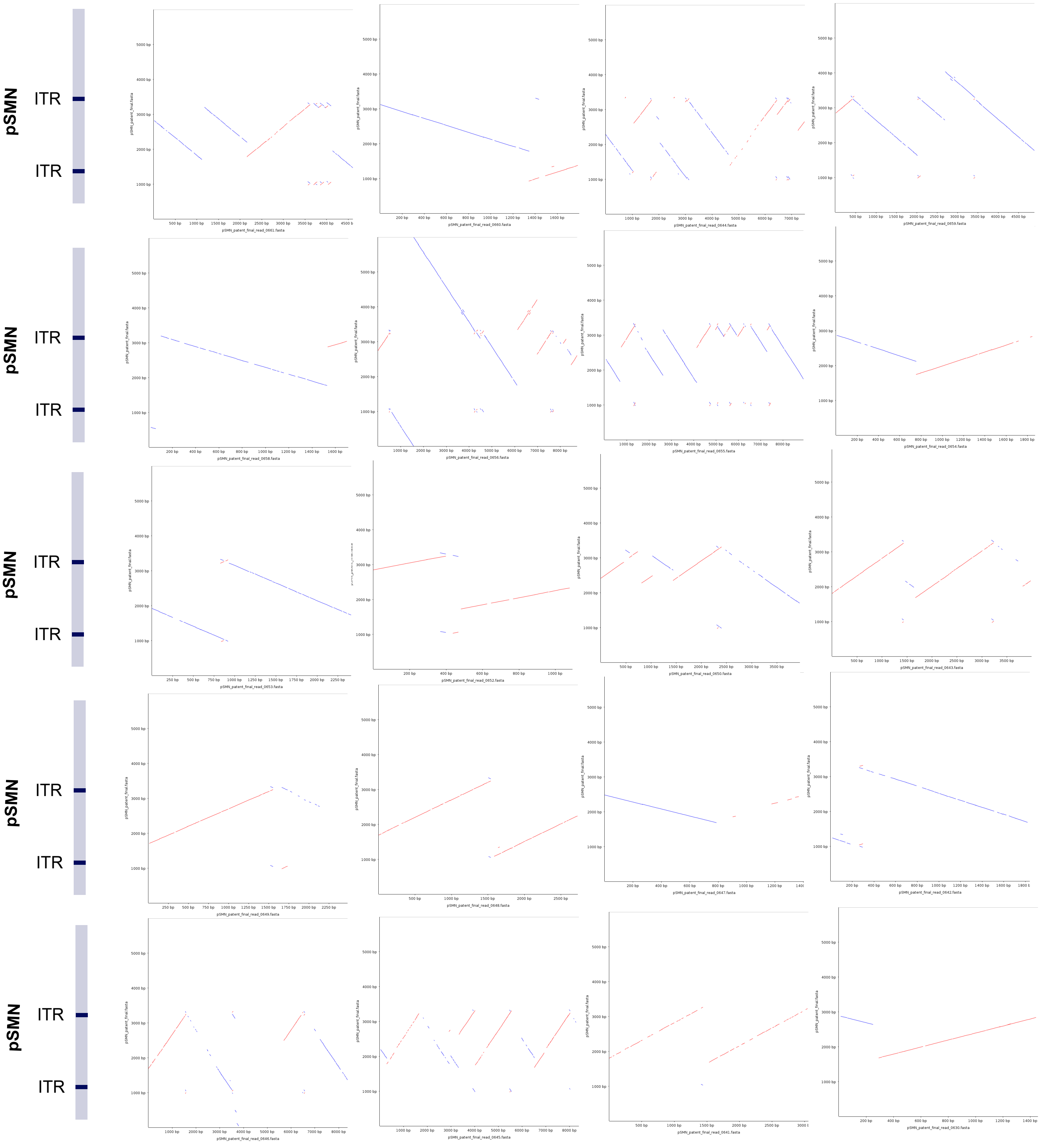
pSMN
ITR
ITR
pSMN_patent_final_read_0661.fasta
pSMN_patent_final_read_0660.fasta
pSMN_patent_final_read_0644.fasta
pSMN_patent_final_read_0659.fasta
pSMN
ITR
ITR
pSMN_patent_final_read_0658.fasta
pSMN_patent_final_read_0656.fasta
pSMN_patent_final_read_0655.fasta
pSMN_patent_final_read_0654.fasta
pSMN
ITR
ITR
pSMN_patent_final_read_0653.fasta
pSMN_patent_final_read_0652.fasta
pSMN_patent_final_read_0650.fasta
pSMN_patent_final_read_0643.fasta
pSMN
ITR
ITR
pSMN_patent_final_read_0649.fasta
pSMN_patent_final_read_0648.fasta
pSMN_patent_final_read_0647.fasta
pSMN_patent_final_read_0642.fasta
pSMN
ITR
ITR
pSMN_patent_final_read_0646.fasta
pSMN_patent_final_read_0645.fasta
pSMN_patent_final_read_0641.fasta
pSMN_patent_final_read_0630.fasta

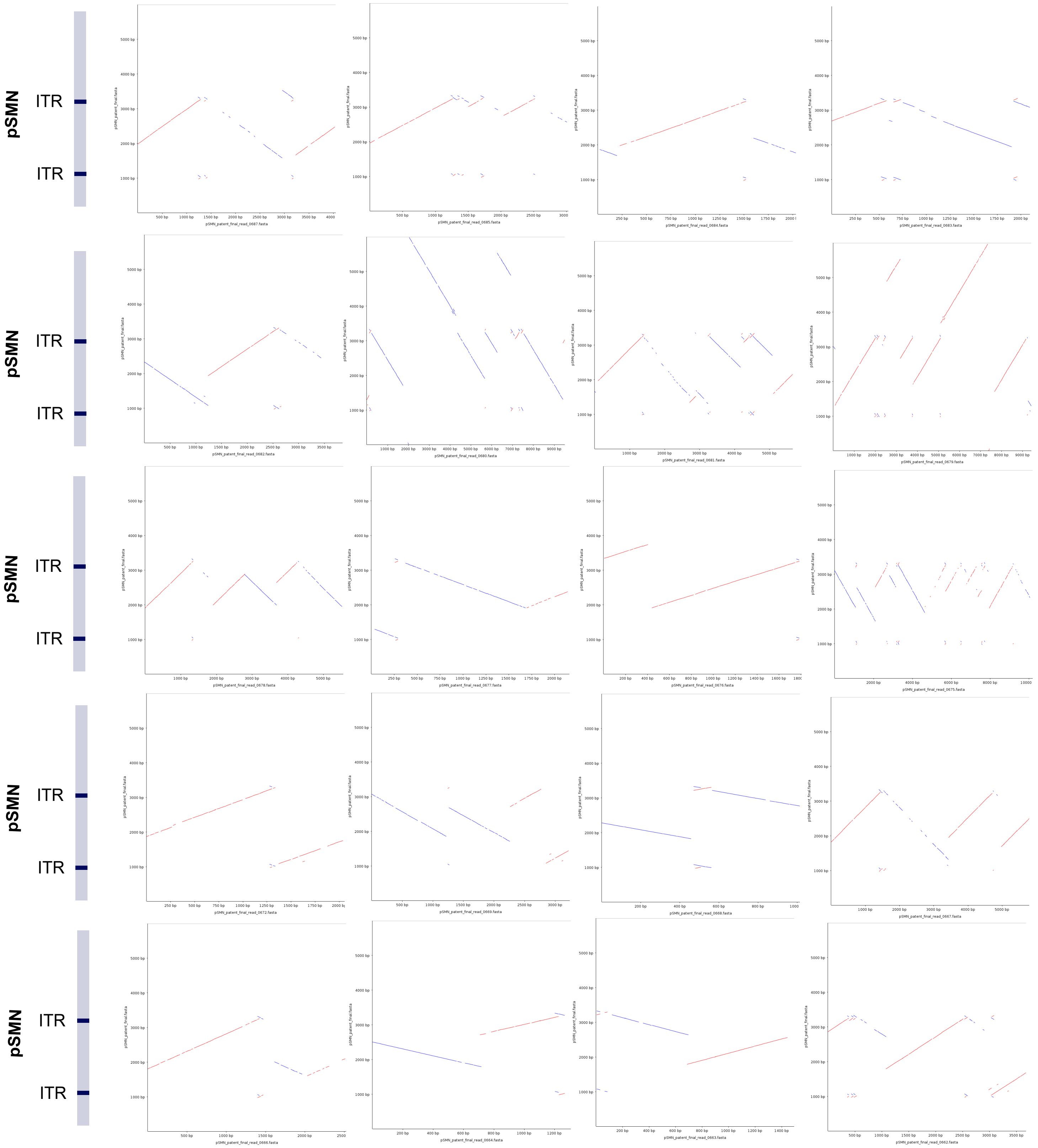

pSMN
ITR
ITR
pSMN
ITR
ITR
pSMN
ITR
ITR
pSMN
ITR
ITR
pSMN
ITR
ITR

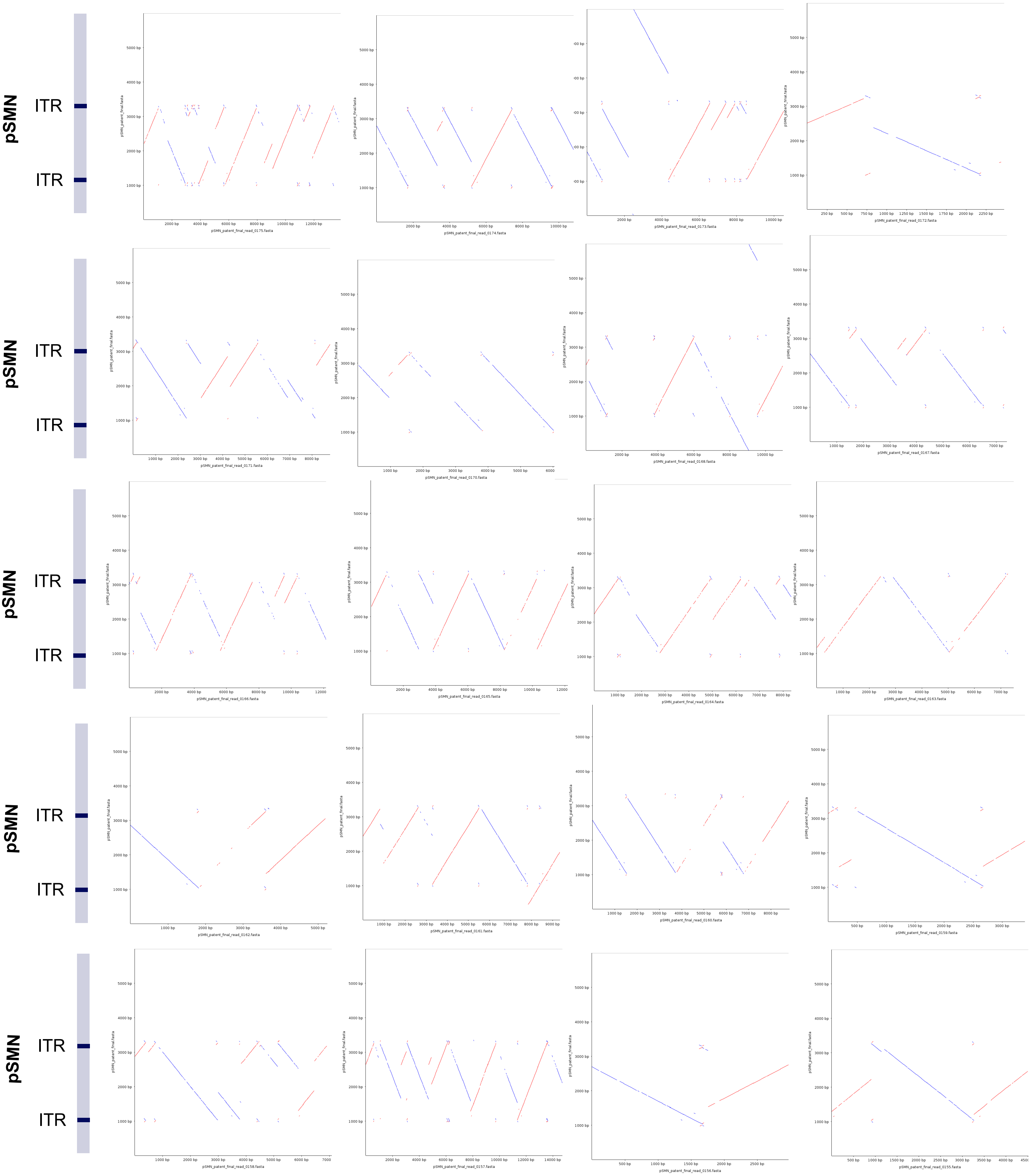

pSMN
ITR
ITR
pSMN_patent_final_read_0175.fasta
pSMN_patent_final_read_0174.fasta
pSMN_patent_final_read_0173.fasta
pSMN_patent_final_read_0172.fasta
pSMN
ITR
ITR
pSMN_patent_final_read_0171.fasta
pSMN_patent_final_read_0170.fasta
pSMN_patent_final_read_0168.fasta
pSMN_patent_final_read_0167.fasta
pSMN
ITR
ITR
pSMN_patent_final_read_0166.fasta
pSMN_patent_final_read_0165.fasta
pSMN_patent_final_read_0164.fasta
pSMN_patent_final_read_0163.fasta
pSMN
ITR
ITR
pSMN_patent_final_read_0162.fasta
pSMN_patent_final_read_0161.fasta
pSMN_patent_final_read_0160.fasta
pSMN_patent_final_read_0159.fasta
pSMN
ITR
ITR
pSMN_patent_final_read_0158.fasta
pSMN_patent_final_read_0157.fasta
pSMN_patent_final_read_0156.fasta
pSMN_patent_final_read_0155.fasta

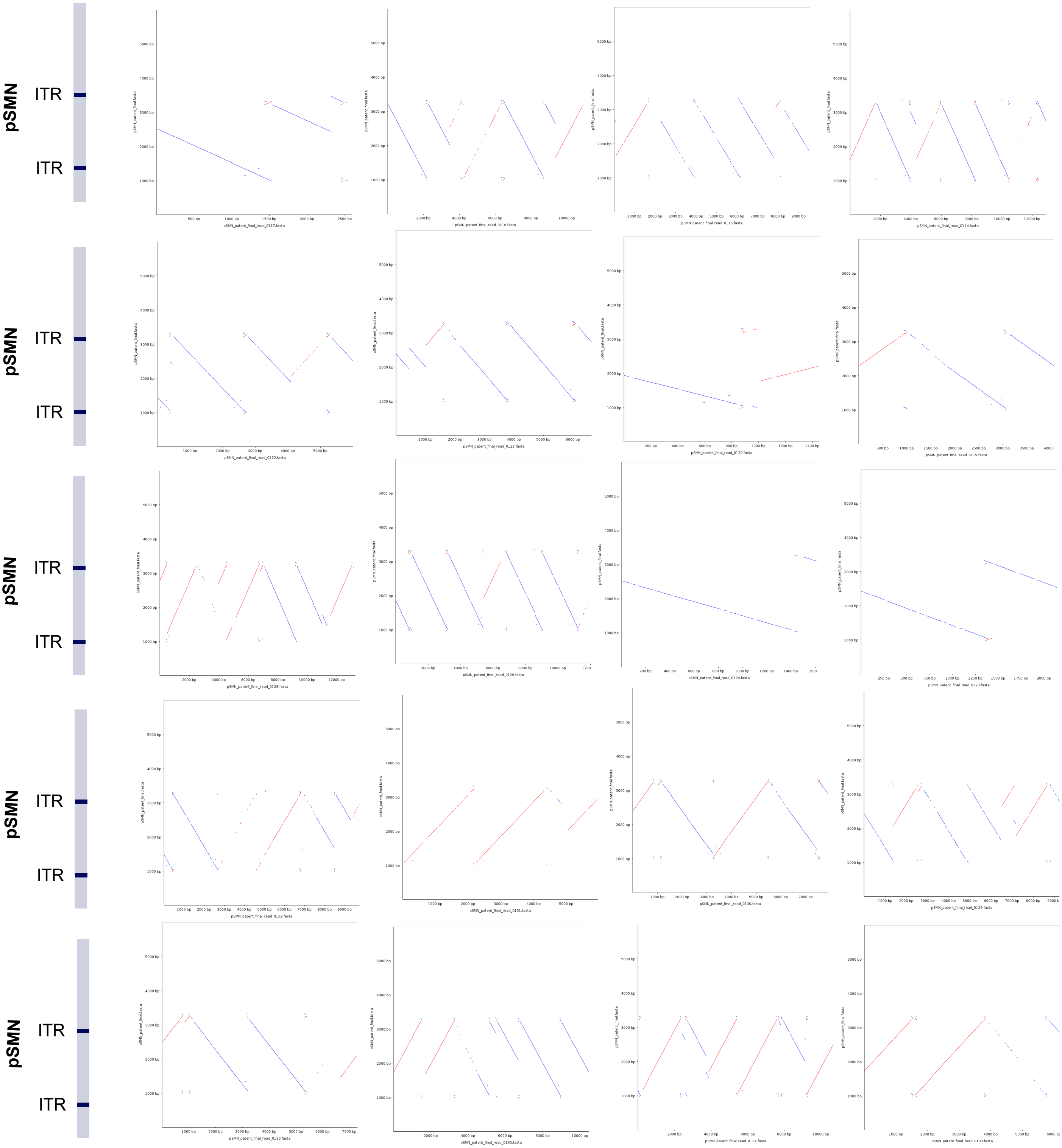
pSMN
ITR
ITR
pSMN_patent_final_read_0117.fasta
pSMN_patent_final_read_0116.fasta
pSMN_patent_final_read_0115.fasta
pSMN_patent_final_read_0114.fasta
pSMN
ITR
ITR
pSMN_patent_final_read_0122.fasta
pSMN_patent_final_read_0121.fasta
pSMN_patent_final_read_0120.fasta
pSMN_patent_final_read_0119.fasta
pSMN
ITR
ITR
pSMN_patent_final_read_0128.fasta
pSMN_patent_final_read_0126.fasta
pSMN_patent_final_read_0124.fasta
pSMN_patent_final_read_0123.fasta
pSMN
ITR
ITR
pSMN_patent_final_read_0132.fasta
pSMN_patent_final_read_0131.fasta
pSMN_patent_final_read_0130.fasta
pSMN_patent_final_read_0129.fasta
pSMN
ITR
ITR
pSMN_patent_final_read_0136.fasta
pSMN_patent_final_read_0135.fasta
pSMN_patent_final_read_0134.fasta
pSMN_patent_final_read_0133.fasta

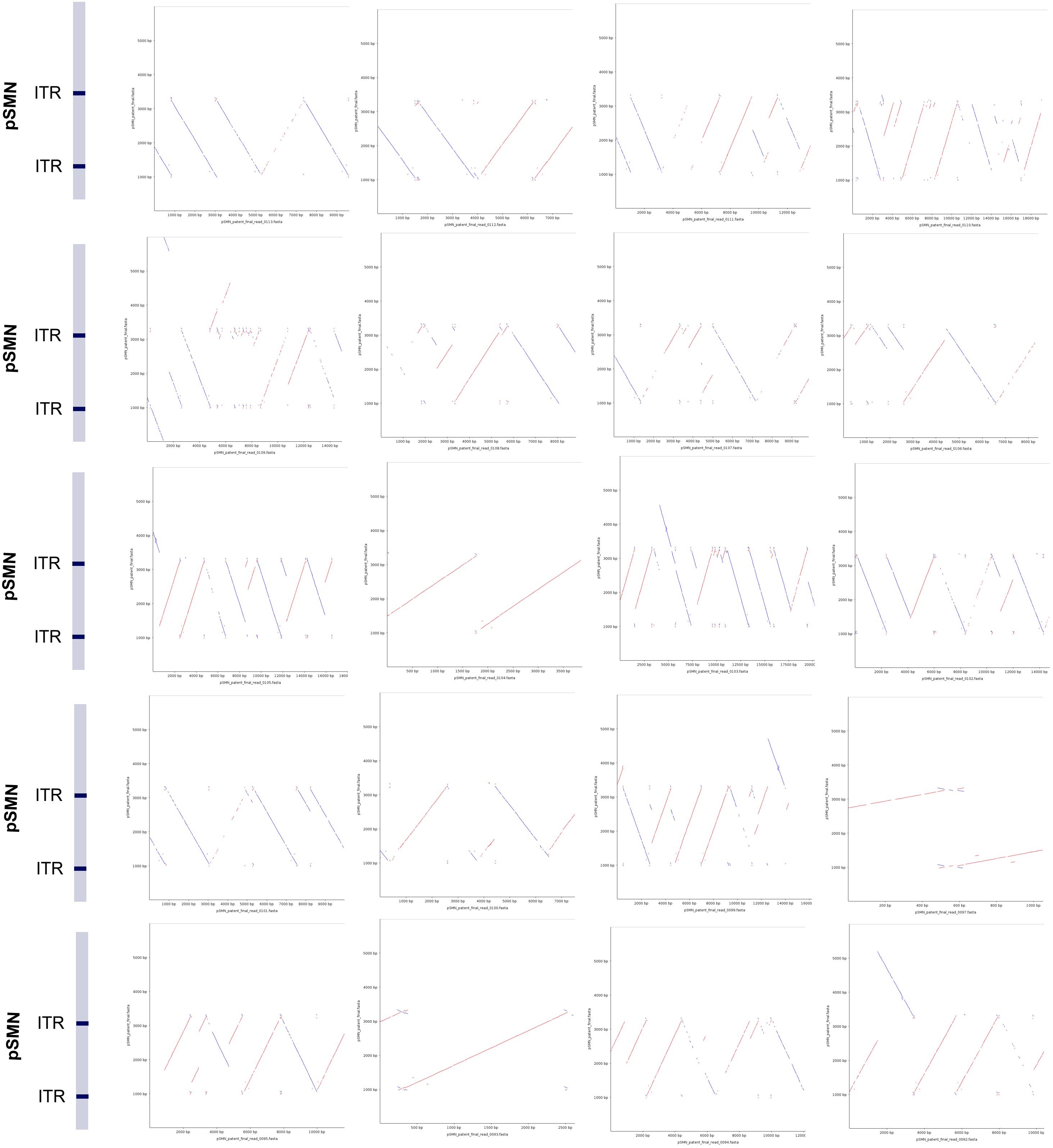

pSMN
ITR
ITR
pSMN
ITR
ITR
pSMN
ITR
ITR
pSMN
ITR
ITR
pSMN
ITR
ITR

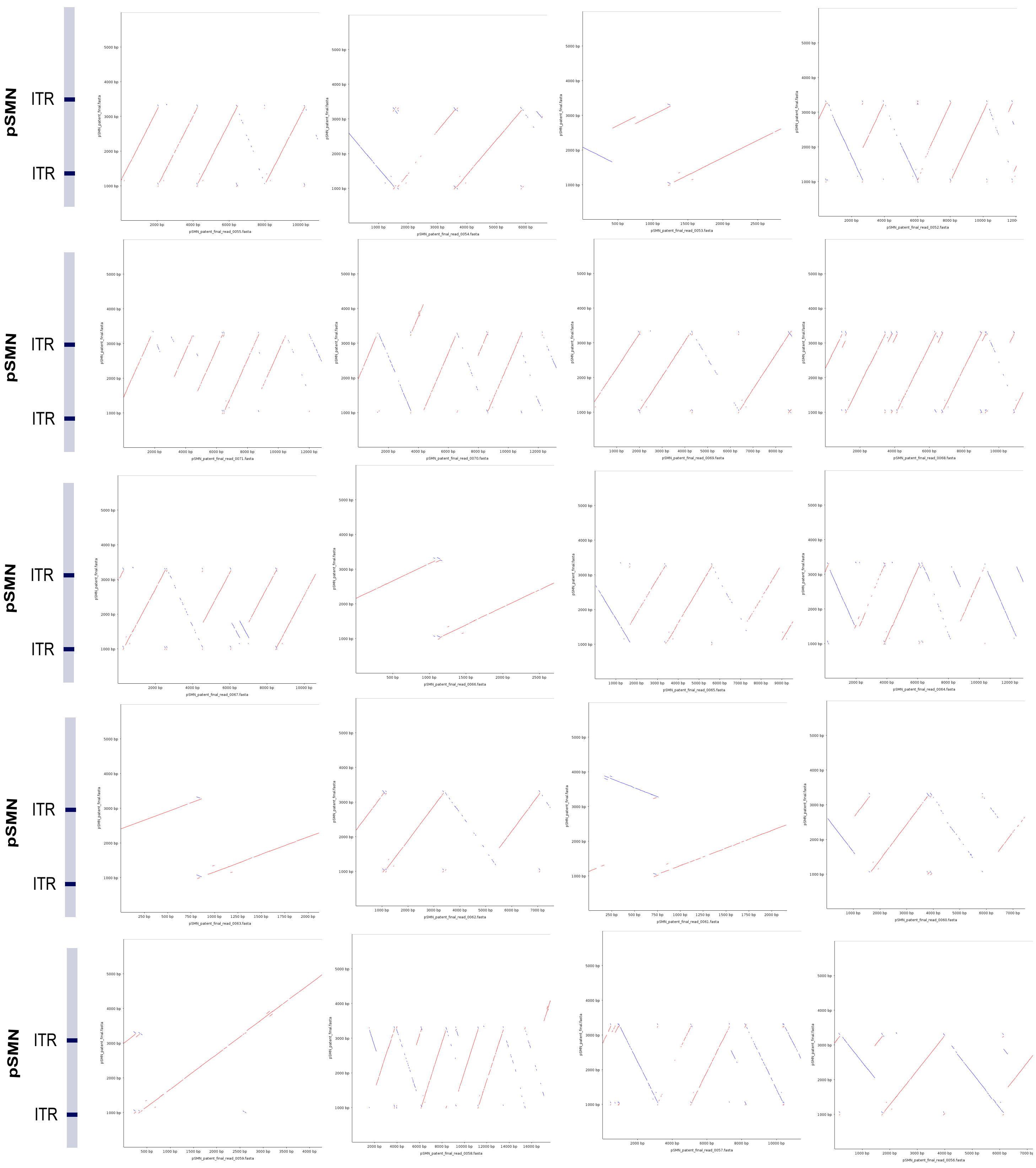
pSMN
ITR
ITR
pSMN
ITR
ITR
pSMN
ITR
ITR
pSMN
ITR
ITR
pSMN
ITR
ITR

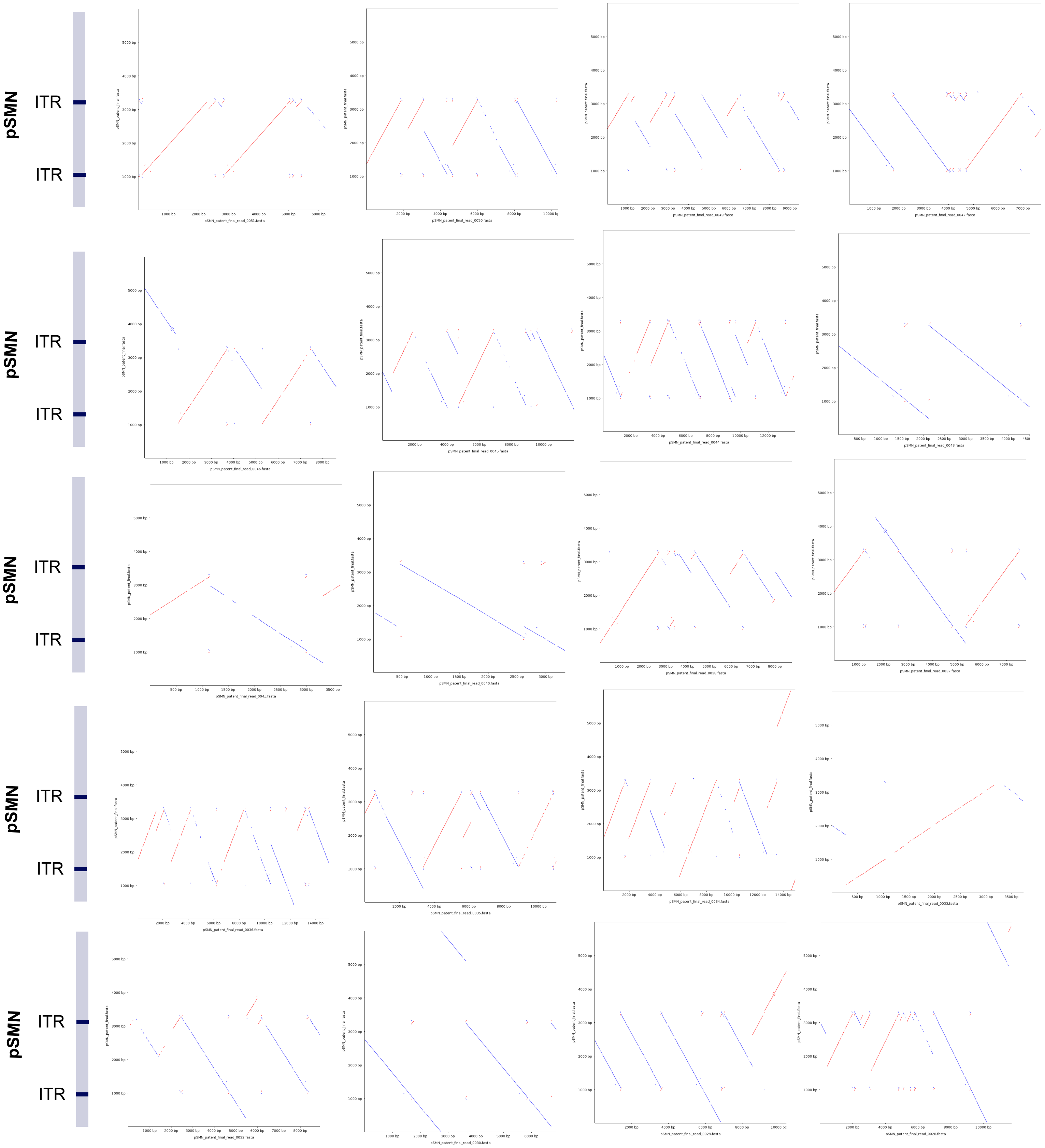
pSMN
ITR
ITR
pSMN
ITR
ITR
pSMN
ITR
ITR
pSMN
ITR
ITR
pSMN
ITR
ITR

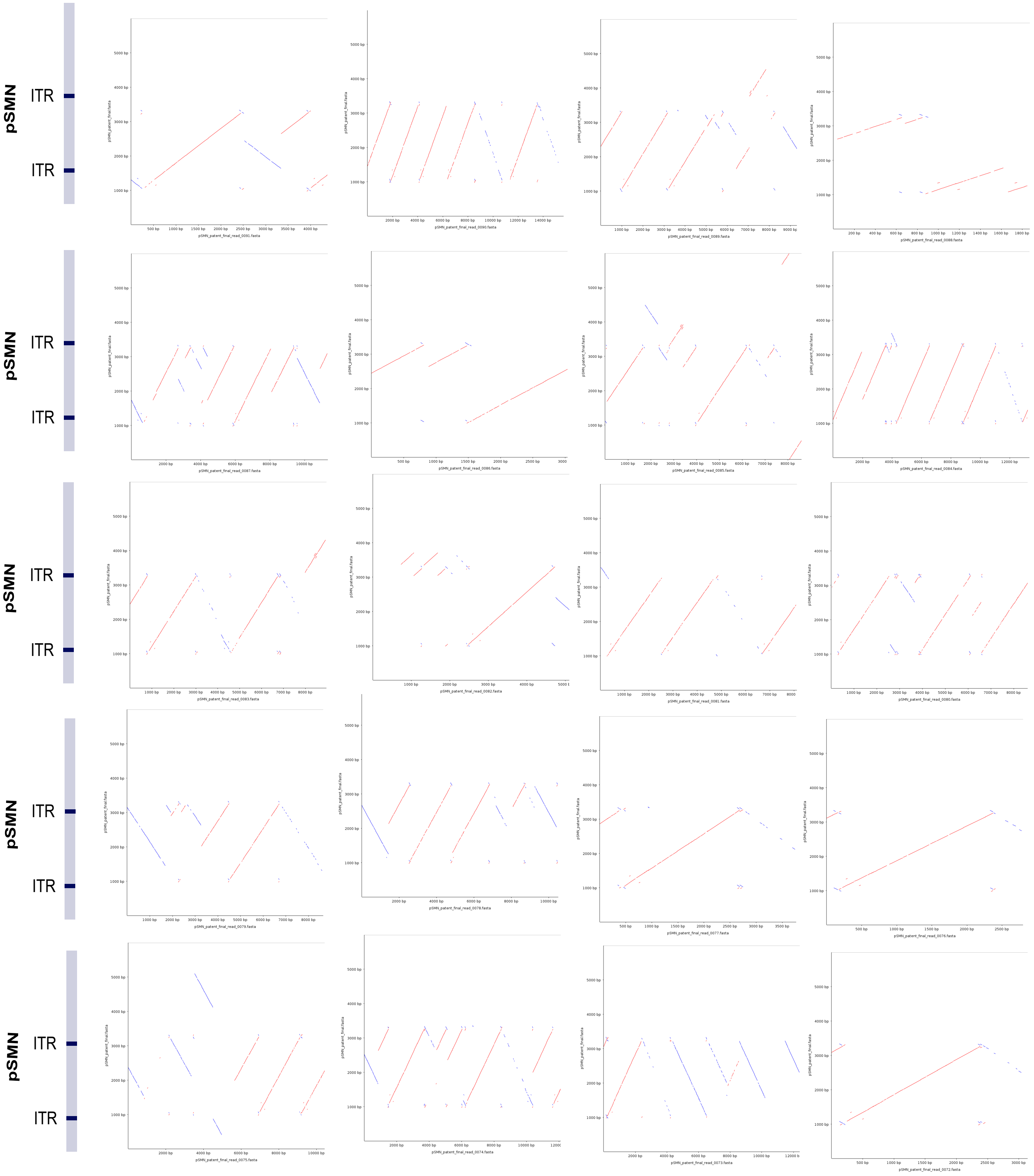
pSMN
ITR
ITR
pSMN
ITR
ITR
pSMN
ITR
ITR
pSMN
ITR
ITR
pSMN
ITR
ITR

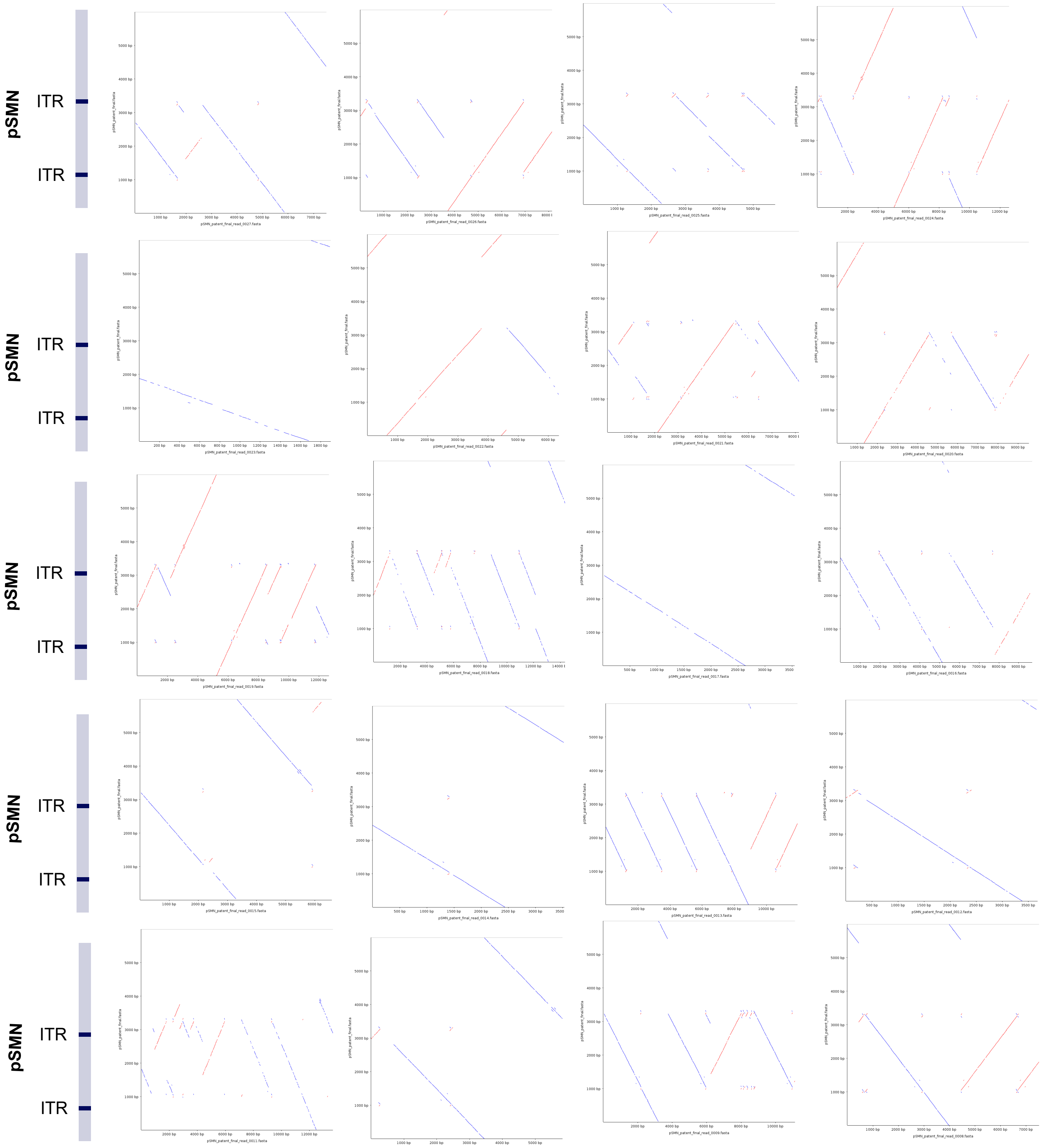
pSMN
ITR
ITR
pSMN_patent_final_read_0027.fasta
pSMN_patent_final_read_0026.fasta
pSMN_patent_final_read_0025.fasta
pSMN_patent_final_read_0024.fasta
pSMN
ITR
ITR
pSMN_patent_final_read_0023.fasta
pSMN_patent_final_read_0022.fasta
pSMN_patent_final_read_0021.fasta
pSMN_patent_final_read_0020.fasta
pSMN
ITR
ITR
pSMN_patent_final_read_0019.fasta
pSMN_patent_final_read_0018.fasta
pSMN_patent_final_read_0017.fasta
pSMN_patent_final_read_0016.fasta
pSMN
ITR
ITR
pSMN_patent_final_read_0015.fasta
pSMN_patent_final_read_0014.fasta
pSMN_patent_final_read_0013.fasta
pSMN_patent_final_read_0012.fasta
pSMN
ITR
ITR
pSMN_patent_final_read_0011.fasta
pSMN_patent_final_read_0010.fasta
pSMN_patent_final_read_0009.fasta
pSMN_patent_final_read_0008.fasta

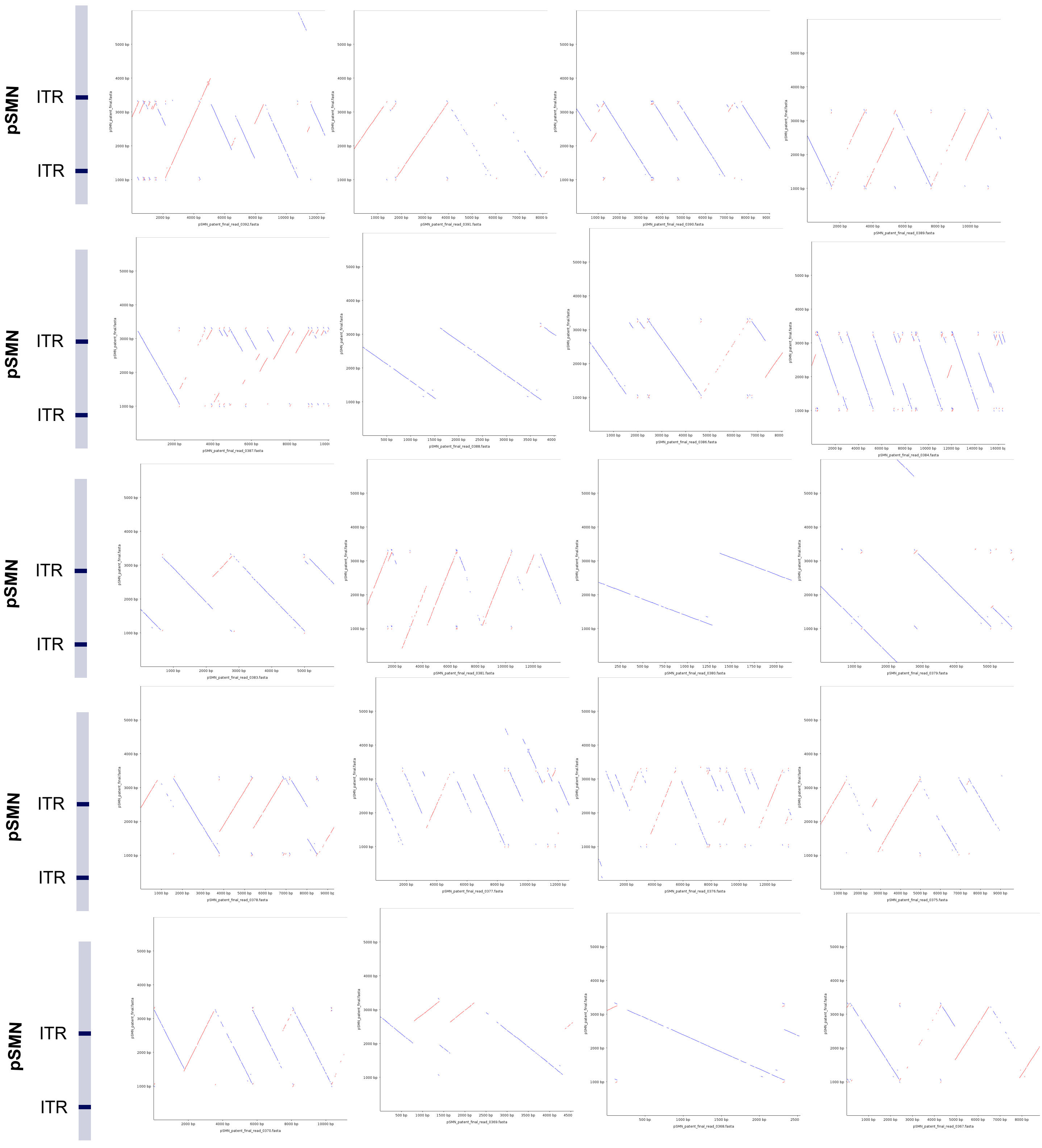
pSMN
ITR
ITR

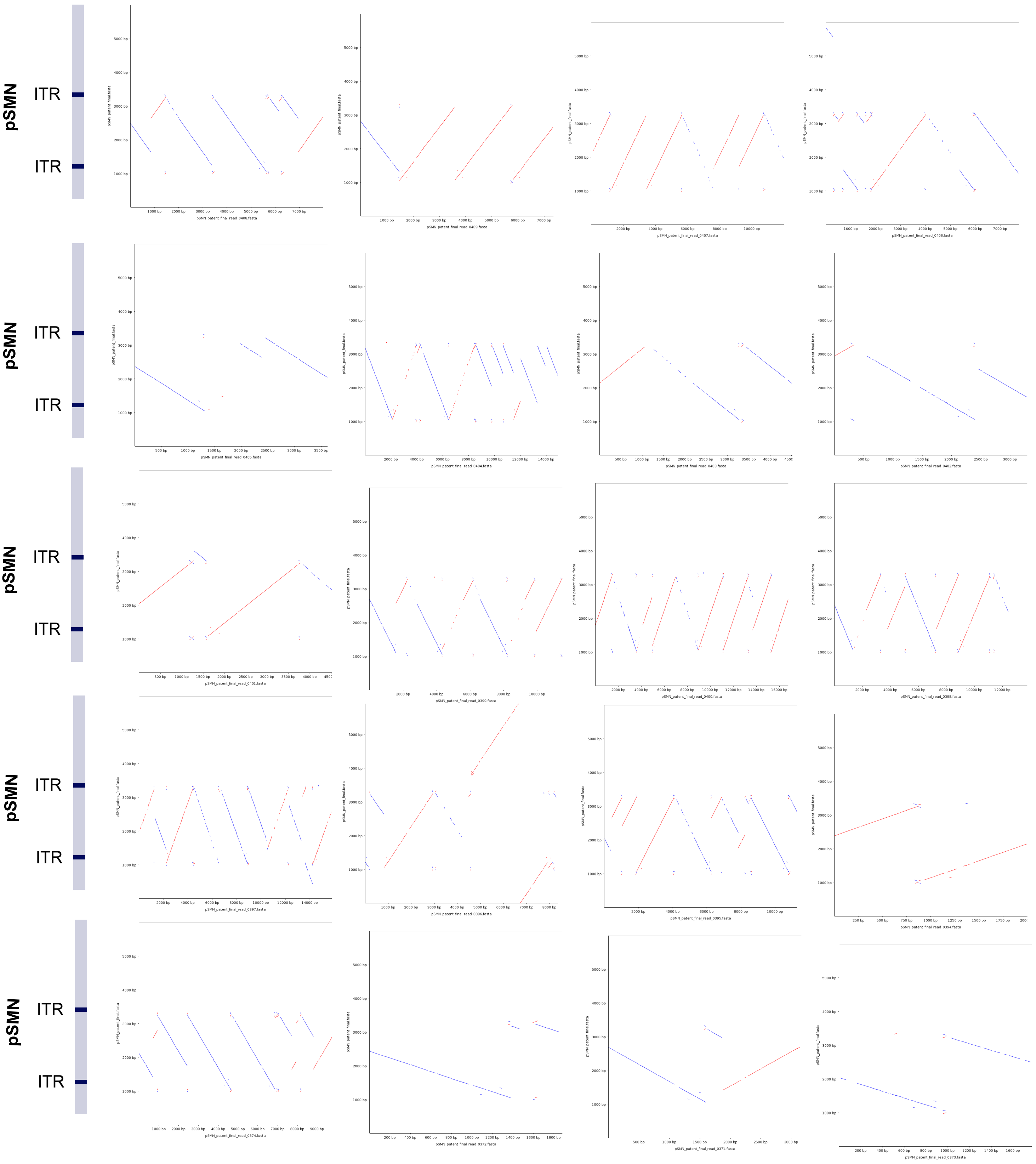
pSMN
ITR
ITR
pSMN
ITR
ITR
pSMN
ITR
ITR
pSMN
ITR
ITR
pSMN
ITR
ITR

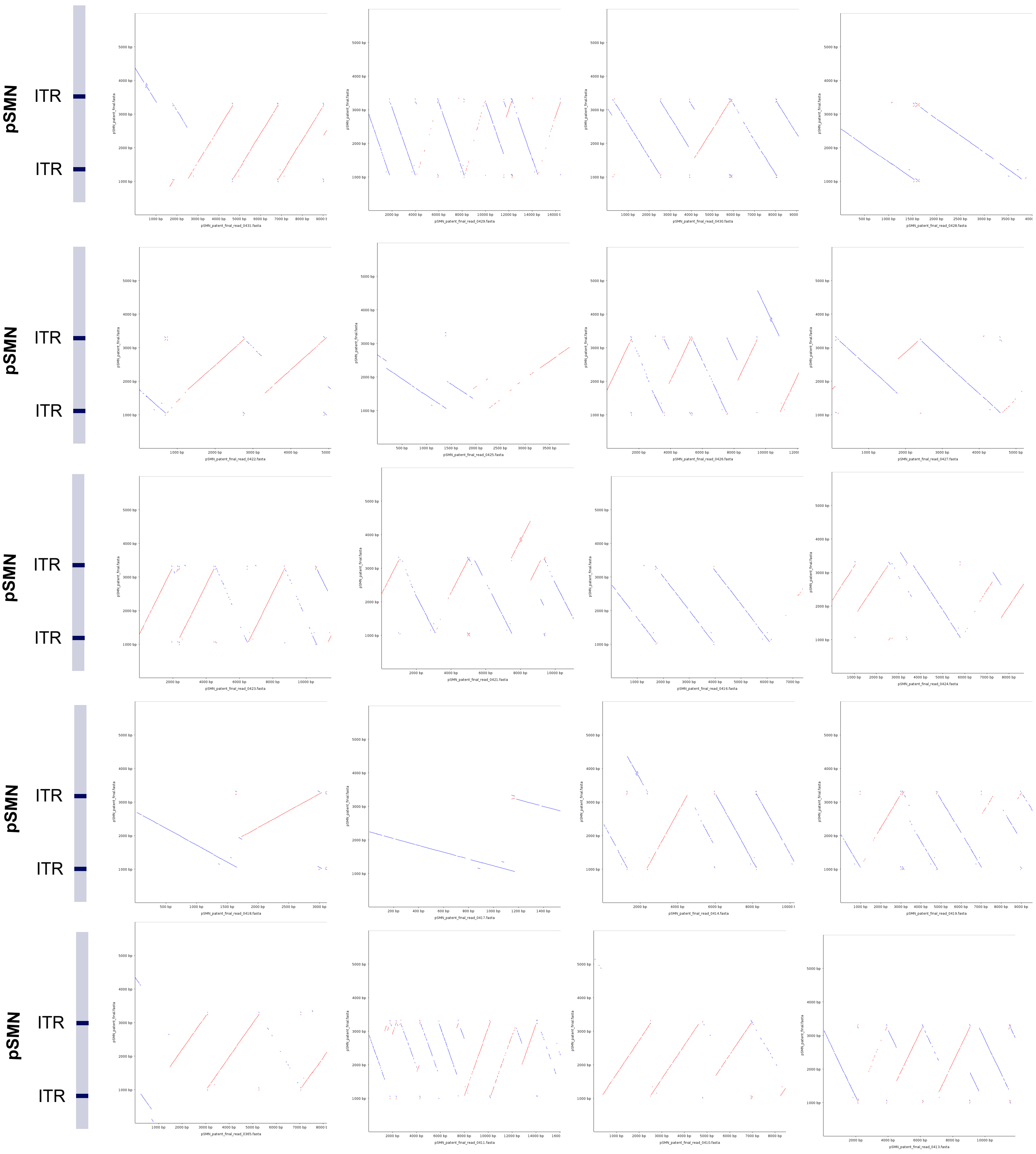
pSMN
ITR
ITR
pSMN
ITR
ITR
pSMN
ITR
ITR
pSMN
ITR
ITR
pSMN
ITR
ITR

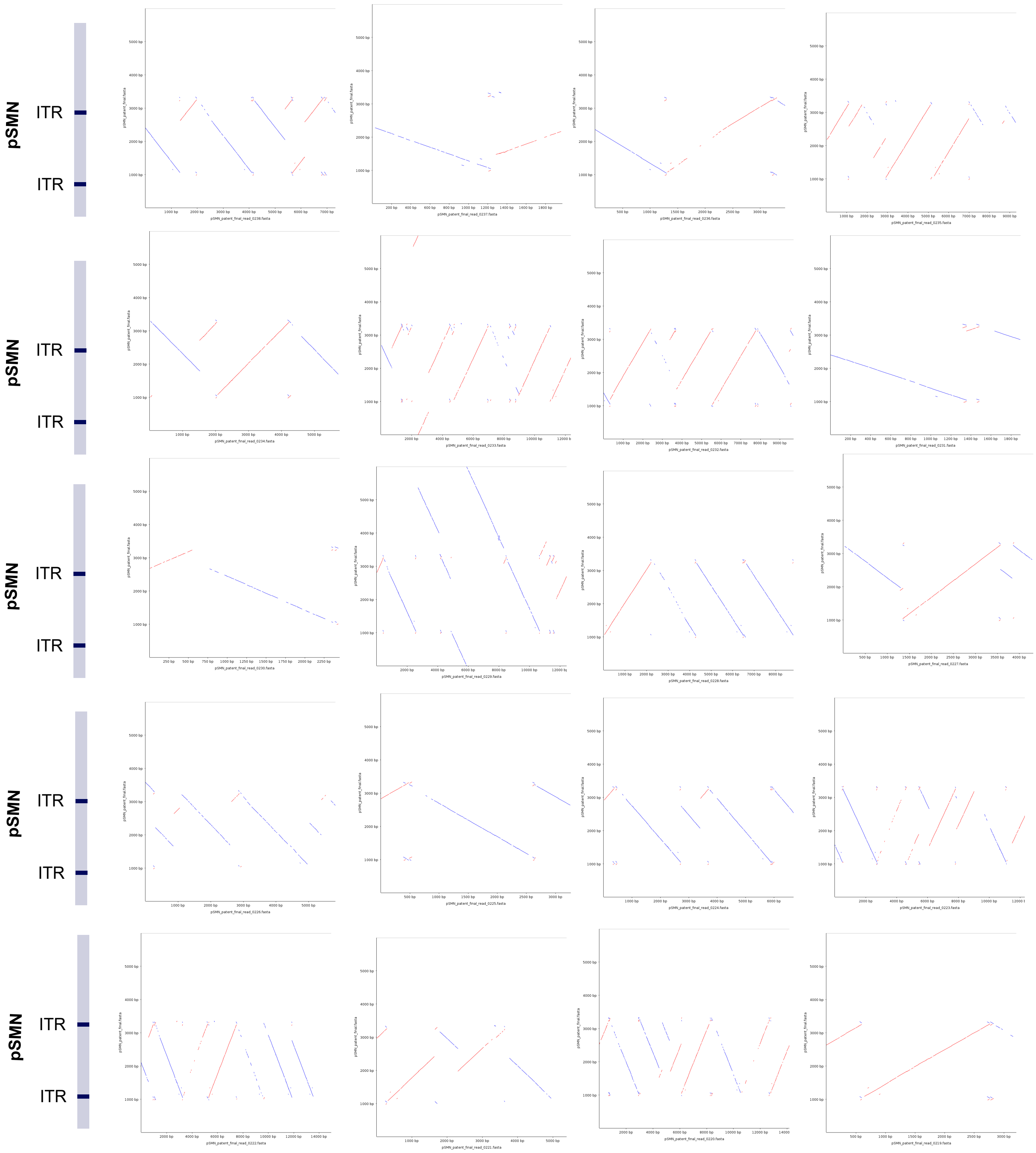
pSMN
ITR
ITR
pSMN
ITR
ITR
pSMN
ITR
ITR
pSMN
ITR
ITR
pSMN
ITR
ITR

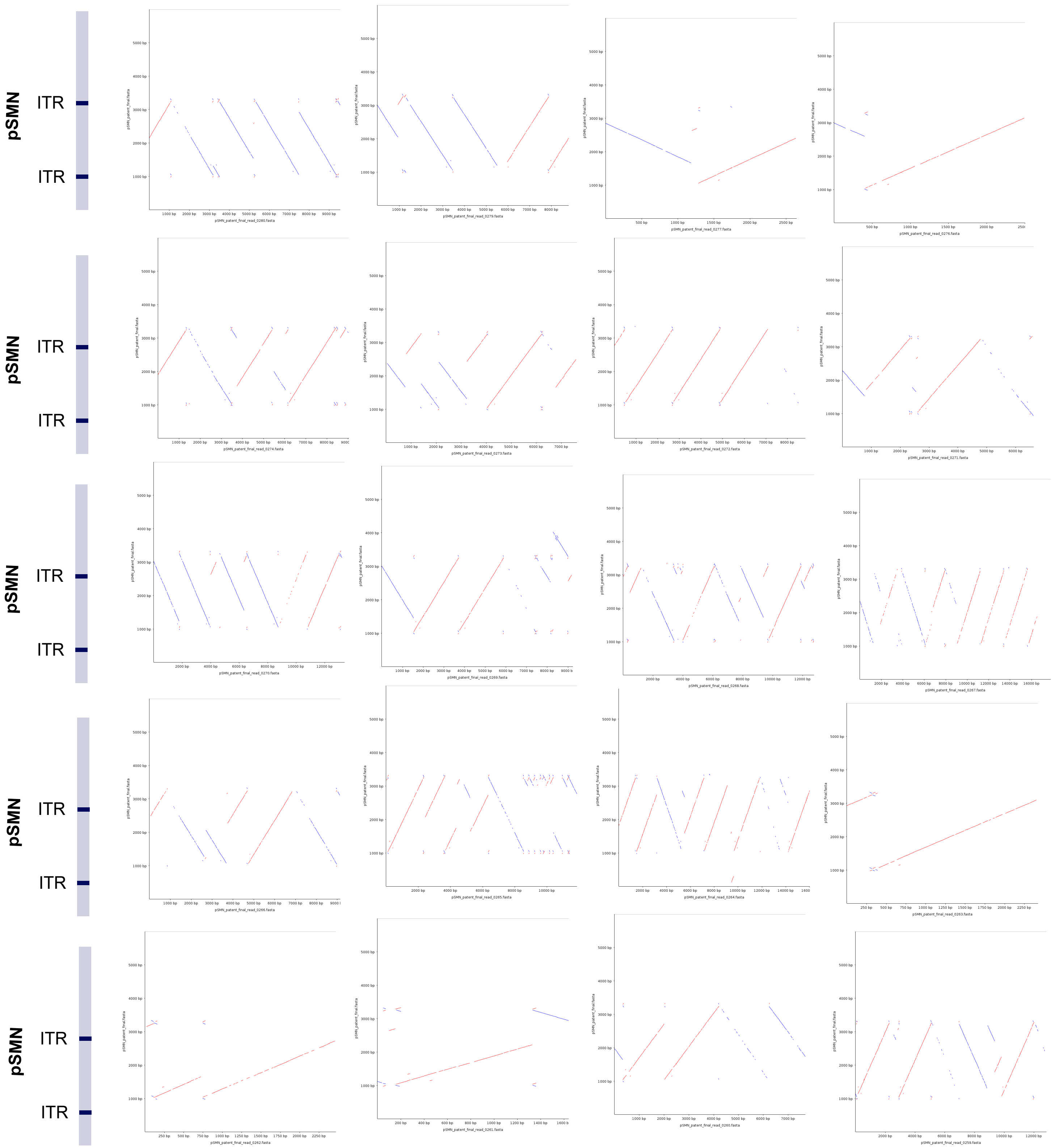
pSMN
ITR
ITR
pSMN_patent_final_read_0280.fasta
pSMN_patent_final_read_0279.fasta
pSMN_patent_final_read_0277.fasta
pSMN_patent_final_read_0276.fasta
pSMN
ITR
ITR
pSMN_patent_final_read_0274.fasta
pSMN_patent_final_read_0273.fasta
pSMN_patent_final_read_0272.fasta
pSMN_patent_final_read_0271.fasta
pSMN
ITR
ITR
pSMN_patent_final_read_0270.fasta
pSMN_patent_final_read_0269.fasta
pSMN_patent_final_read_0268.fasta
pSMN_patent_final_read_0267.fasta
pSMN
ITR
ITR
pSMN_patent_final_read_0266.fasta
pSMN_patent_final_read_0265.fasta
pSMN_patent_final_read_0264.fasta
pSMN_patent_final_read_0263.fasta
pSMN
ITR
ITR
pSMN_patent_final_read_0262.fasta
pSMN_patent_final_read_0261.fasta
pSMN_patent_final_read_0260.fasta
pSMN_patent_final_read_0259.fasta

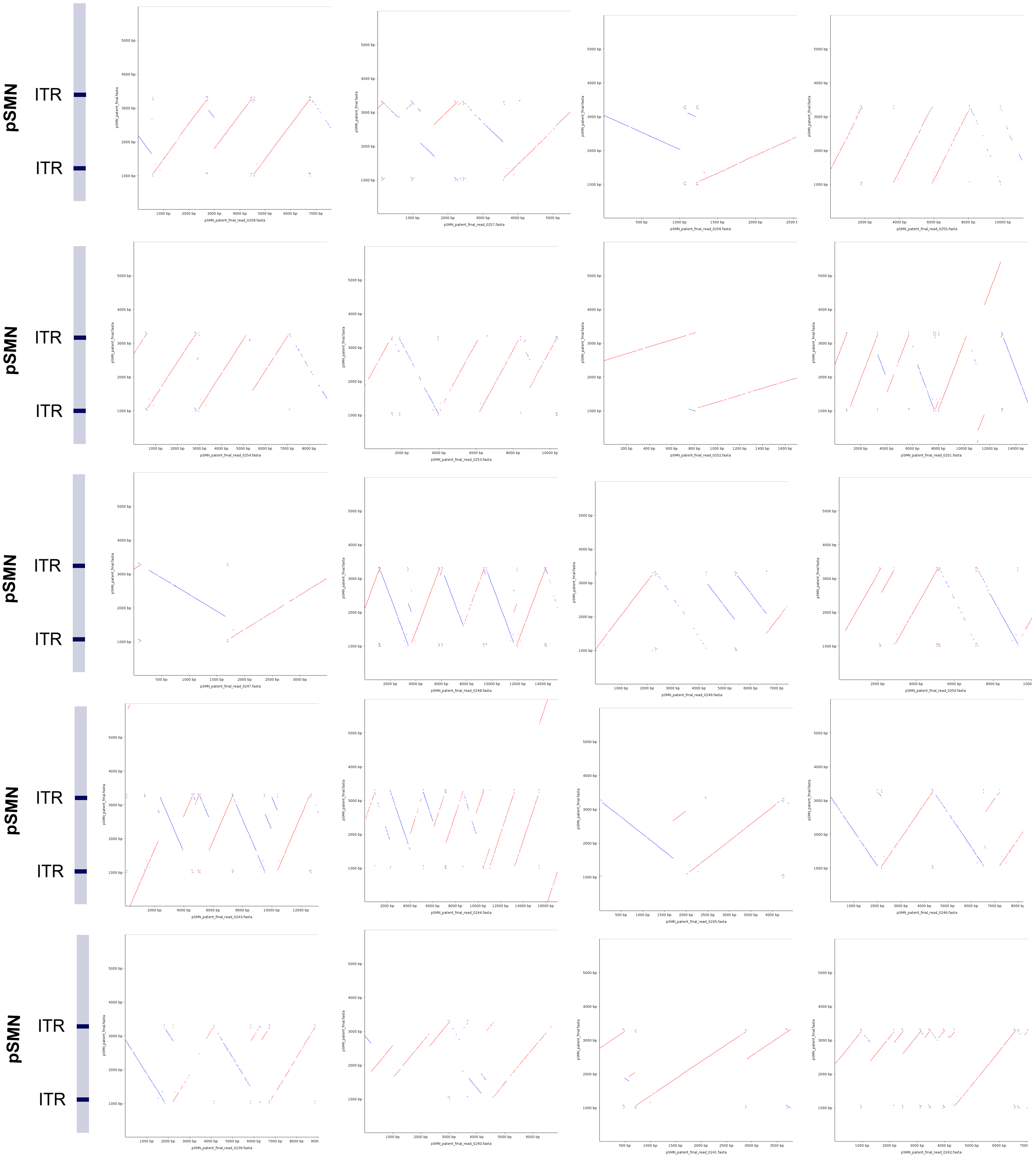
pSMN
ITR
ITR
pSMN
ITR
ITR
pSMN
ITR
ITR
pSMN
ITR
ITR
pSMN
ITR
ITR

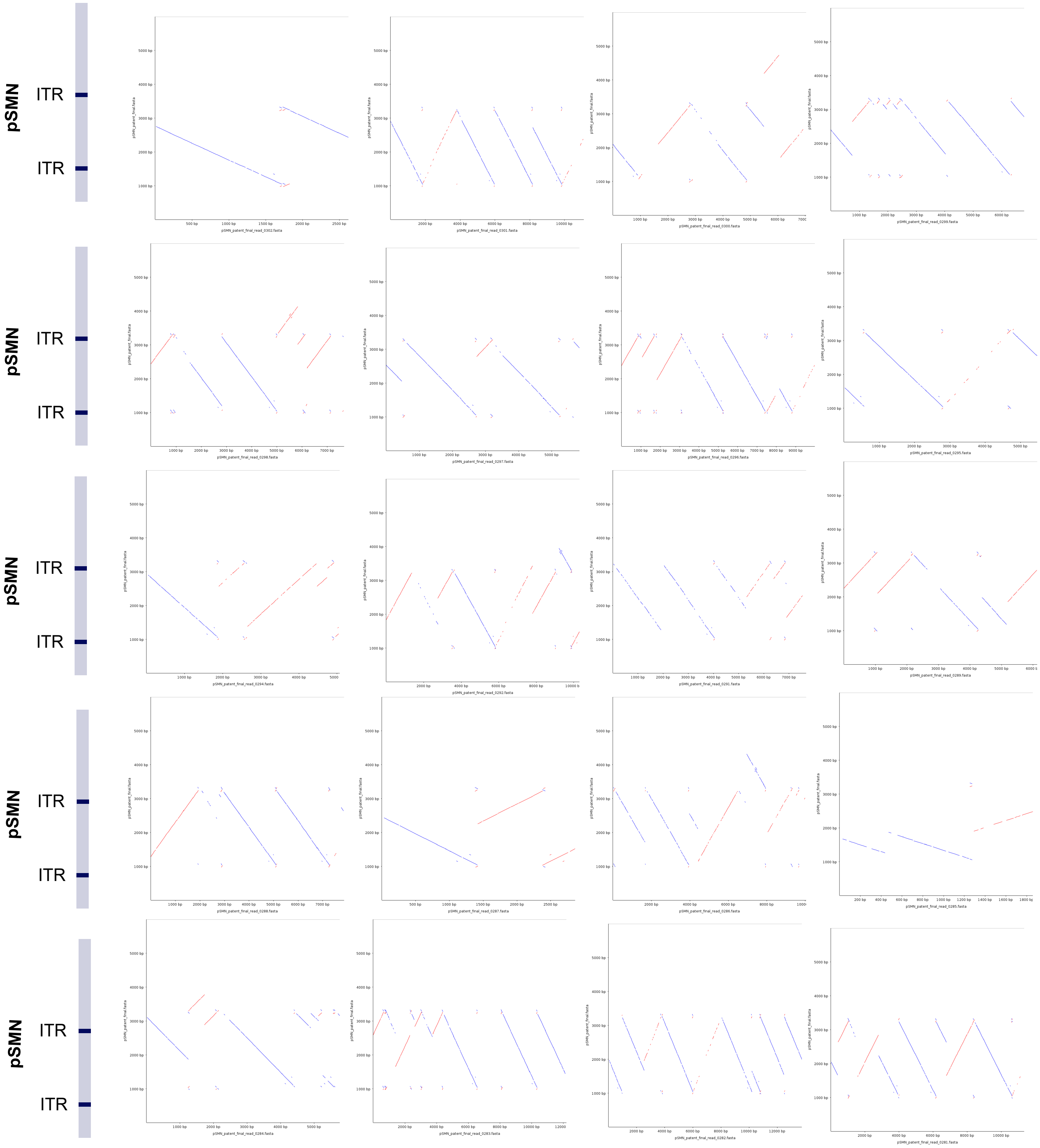
pSMN
ITR
ITR
pSMN
ITR
ITR
pSMN
ITR
ITR
pSMN
ITR
ITR
pSMN
ITR
ITR

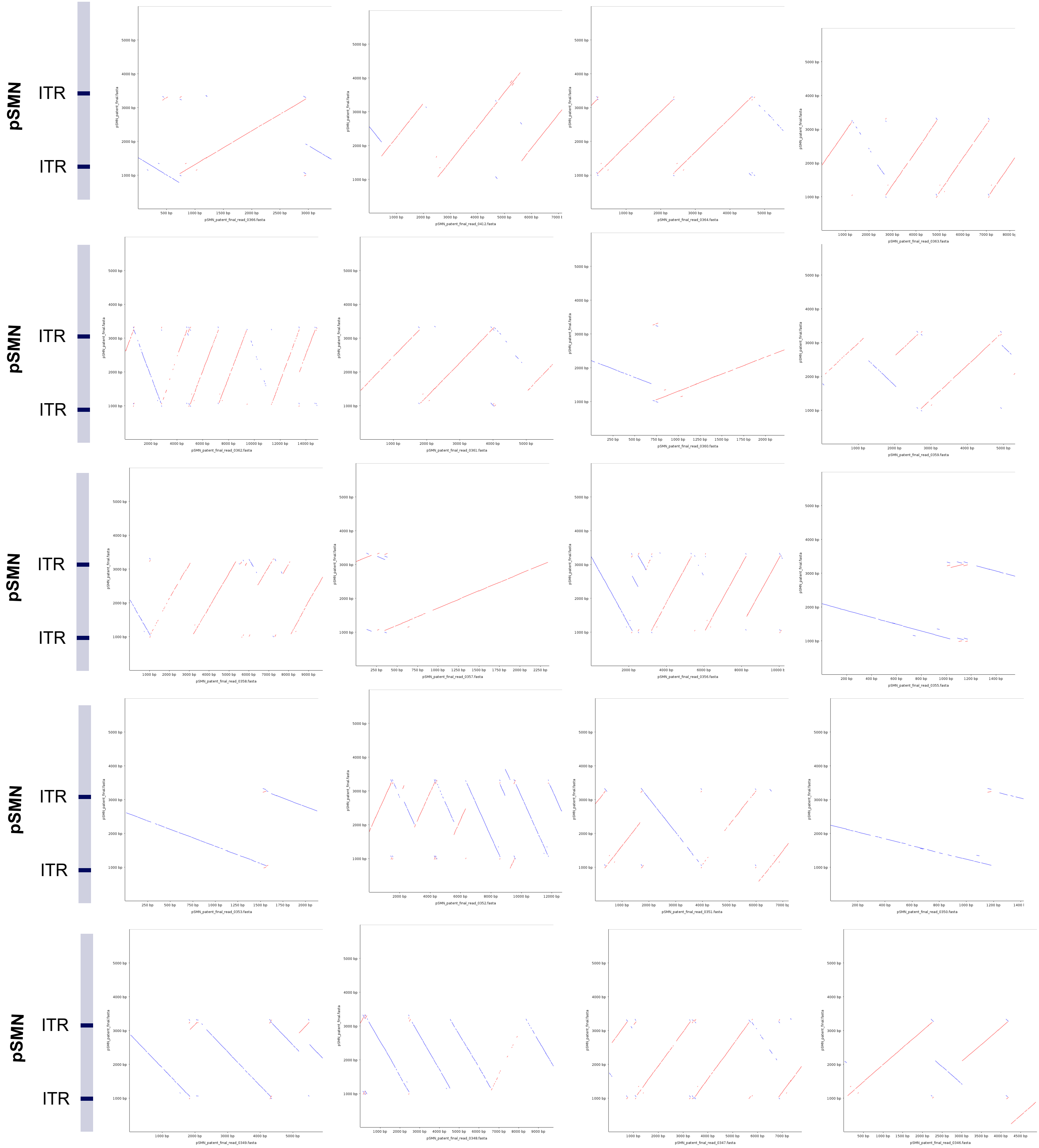
pSMN
ITR
ITR
pSMN_patent_final_read_0366.fasta
pSMN_patent_final_read_0412.fasta
pSMN_patent_final_read_0364.fasta
pSMN_patent_final_read_0363.fasta
pSMN
ITR
ITR
pSMN_patent_final_read_0362.fasta
pSMN_patent_final_read_0361.fasta
pSMN_patent_final_read_0360.fasta
pSMN_patent_final_read_0359.fasta
pSMN
ITR
ITR
pSMN_patent_final_read_0358.fasta
pSMN_patent_final_read_0357.fasta
pSMN_patent_final_read_0356.fasta
pSMN_patent_final_read_0355.fasta
pSMN
ITR
ITR
pSMN_patent_final_read_0353.fasta
pSMN_patent_final_read_0352.fasta
pSMN_patent_final_read_0351.fasta
pSMN_patent_final_read_0350.fasta
pSMN
ITR
ITR
pSMN_patent_final_read_0349.fasta
pSMN_patent_final_read_0348.fasta
pSMN_patent_final_read_0347.fasta
pSMN_patent_final_read_0346.fasta

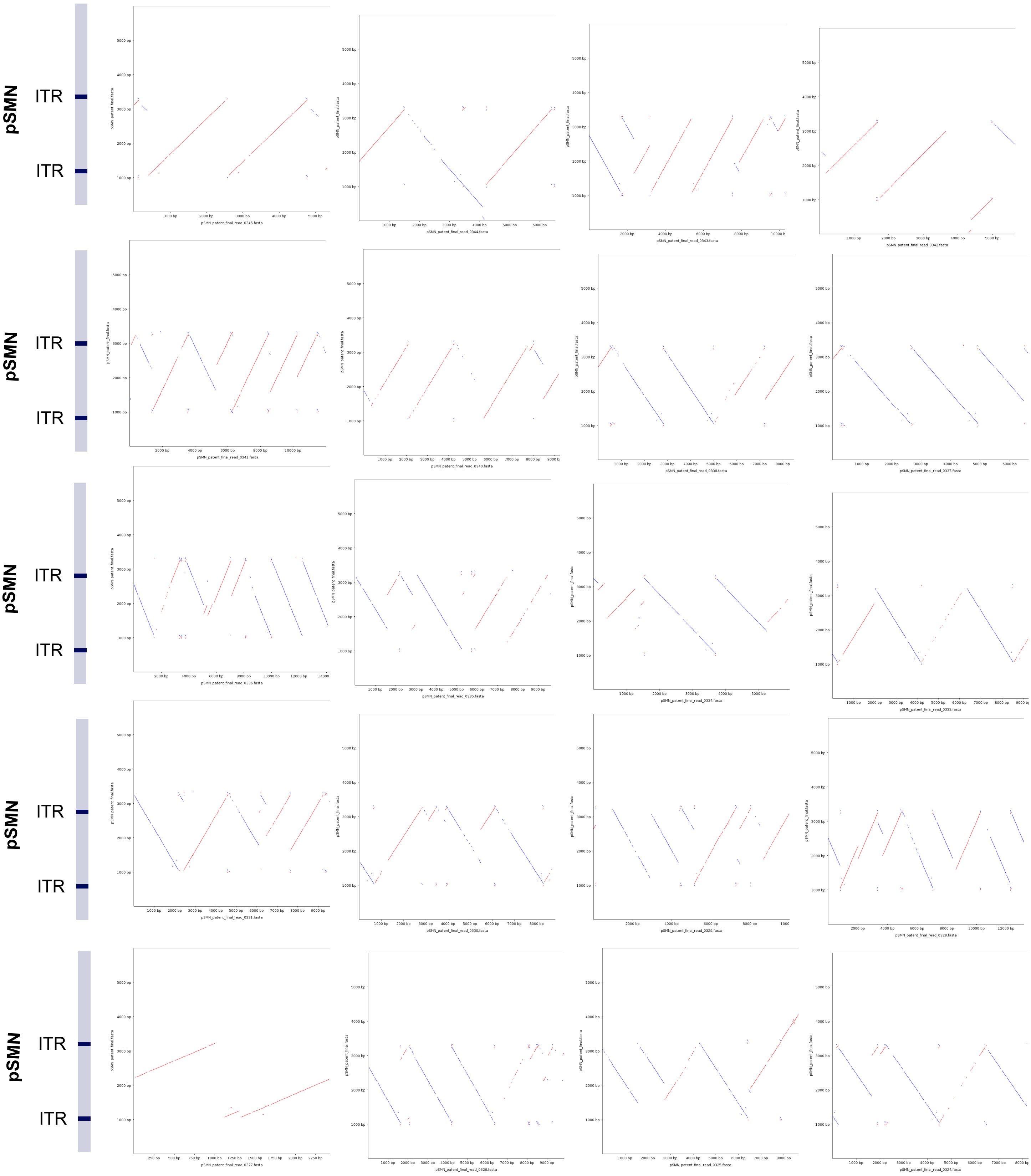
pSMN
ITR
ITR
pSMN_patent_final_read_0345.fasta
pSMN_patent_final_read_0344.fasta
pSMN_patent_final_read_0343.fasta
pSMN_patent_final_read_0342.fasta
pSMN
ITR
ITR
pSMN_patent_final_read_0341.fasta
pSMN_patent_final_read_0340.fasta
pSMN_patent_final_read_0338.fasta
pSMN_patent_final_read_0337.fasta
pSMN
ITR
ITR
pSMN_patent_final_read_0336.fasta
pSMN_patent_final_read_0335.fasta
pSMN_patent_final_read_0334.fasta
pSMN_patent_final_read_0333.fasta
pSMN
ITR
ITR
pSMN_patent_final_read_0331.fasta
pSMN_patent_final_read_0330.fasta
pSMN_patent_final_read_0329.fasta
pSMN_patent_final_read_0328.fasta
pSMN
ITR
ITR
pSMN_patent_final_read_0327.fasta
pSMN_patent_final_read_0326.fasta
pSMN_patent_final_read_0325.fasta
pSMN_patent_final_read_0324.fasta

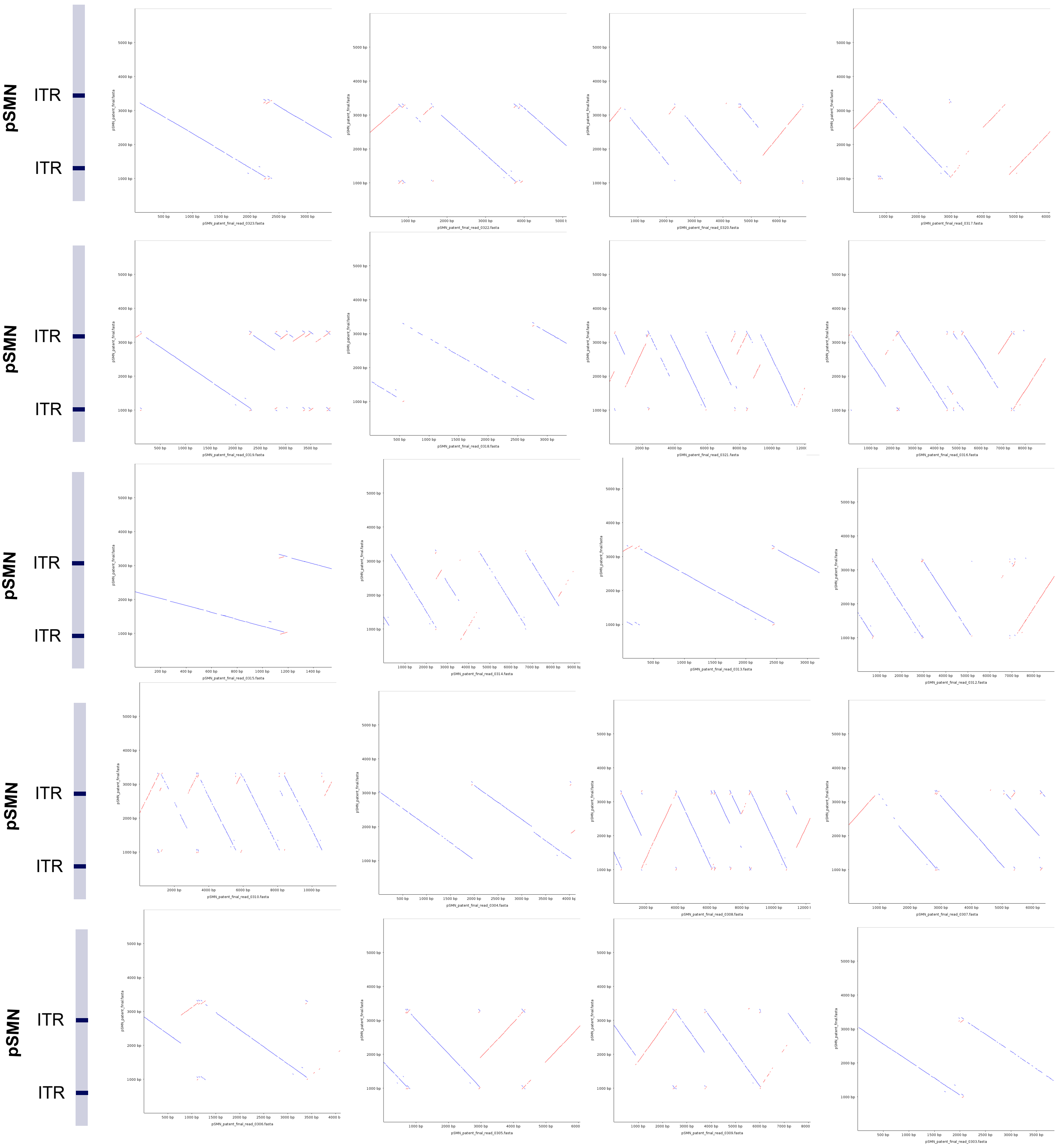
pSMN
ITR
ITR
pSMN
ITR
ITR
pSMN
ITR
ITR
pSMN
ITR
ITR
pSMN
ITR
ITR

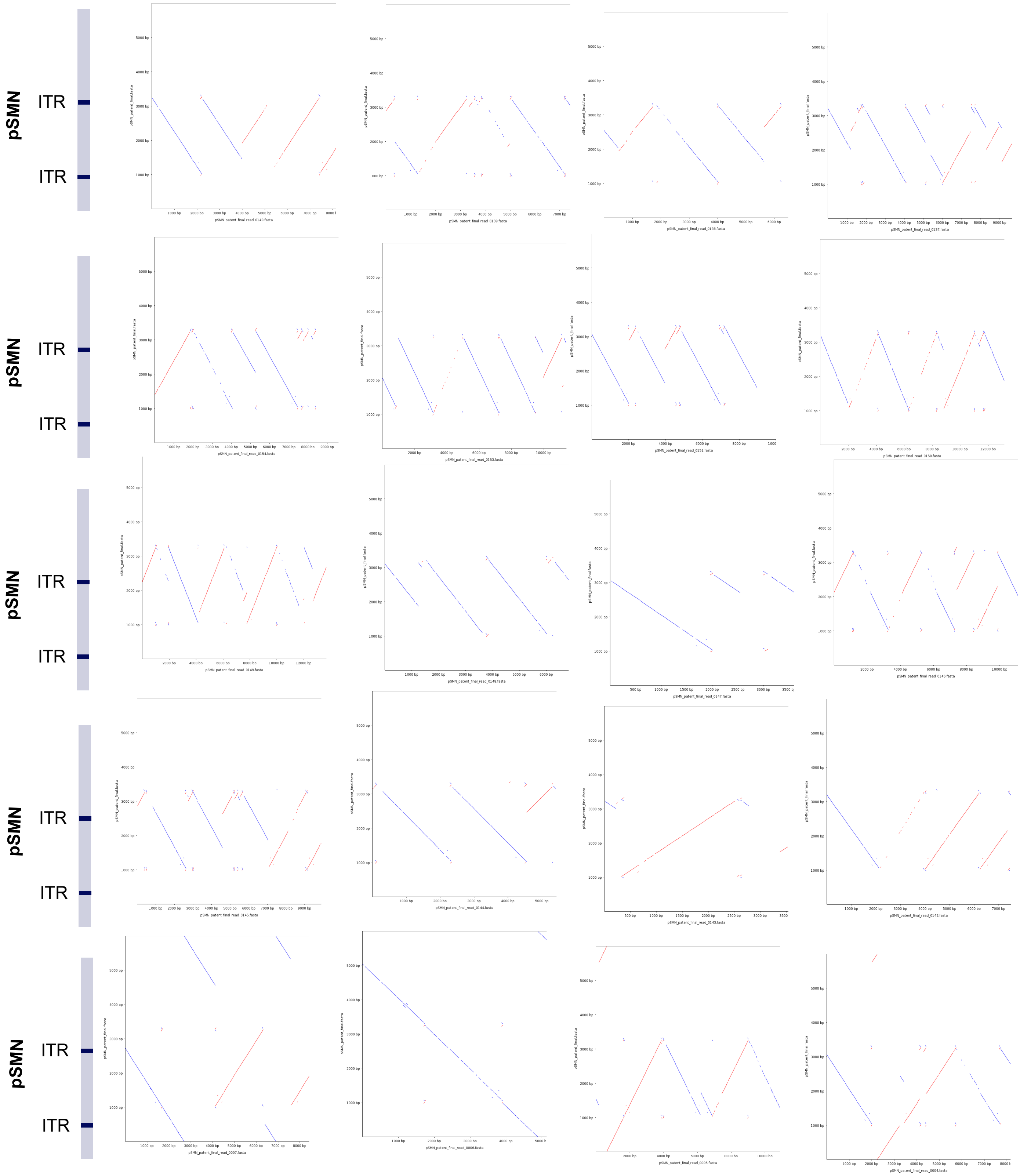
pSMN
ITR
ITR
pSMN
ITR
ITR
pSMN
ITR
ITR
pSMN
ITR
ITR
pSMN
ITR
ITR

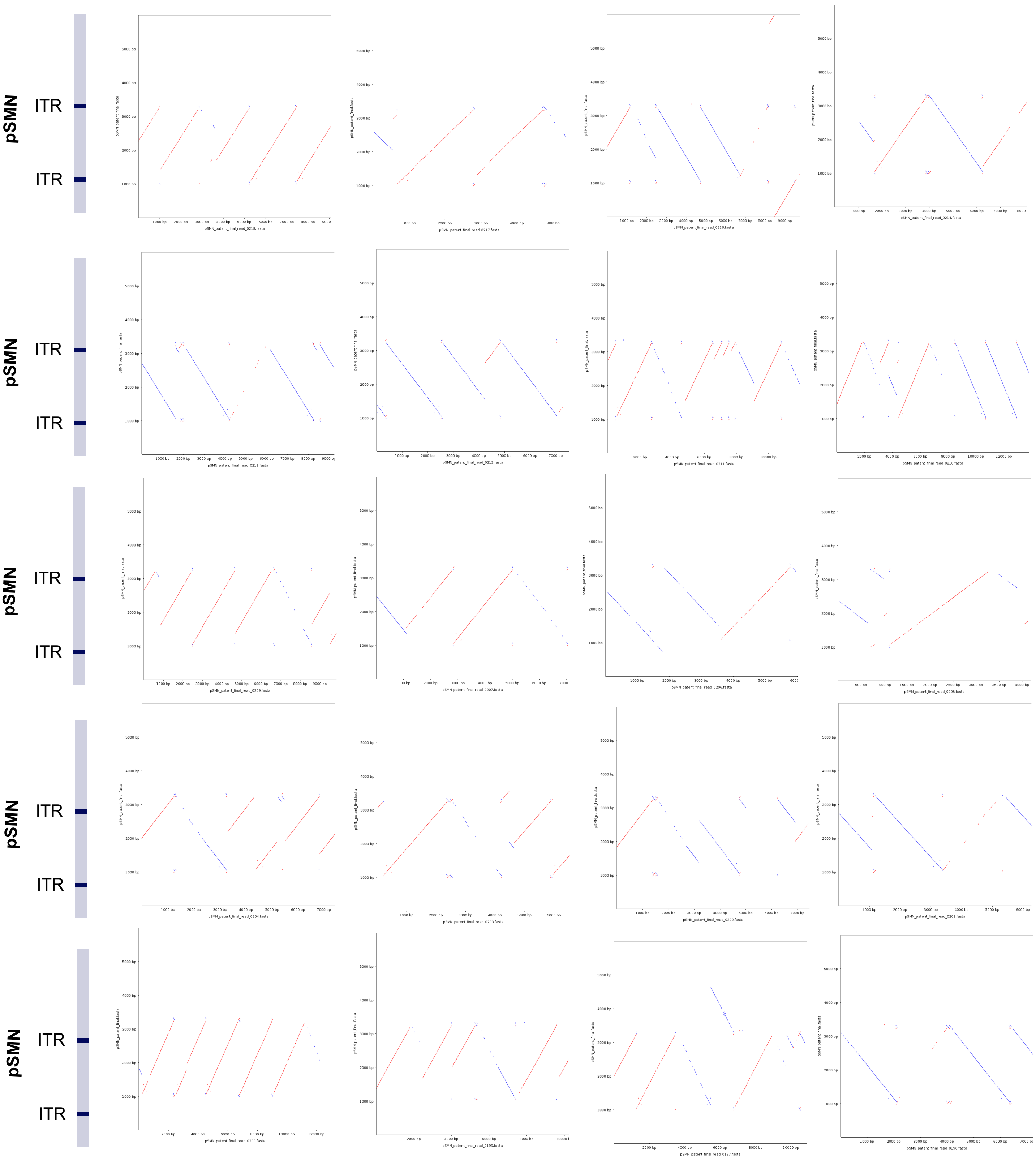

pSMN
ITR
ITR
pSMN
ITR
ITR
pSMN
ITR
ITR
pSMN
ITR
ITR
pSMN
ITR
ITR

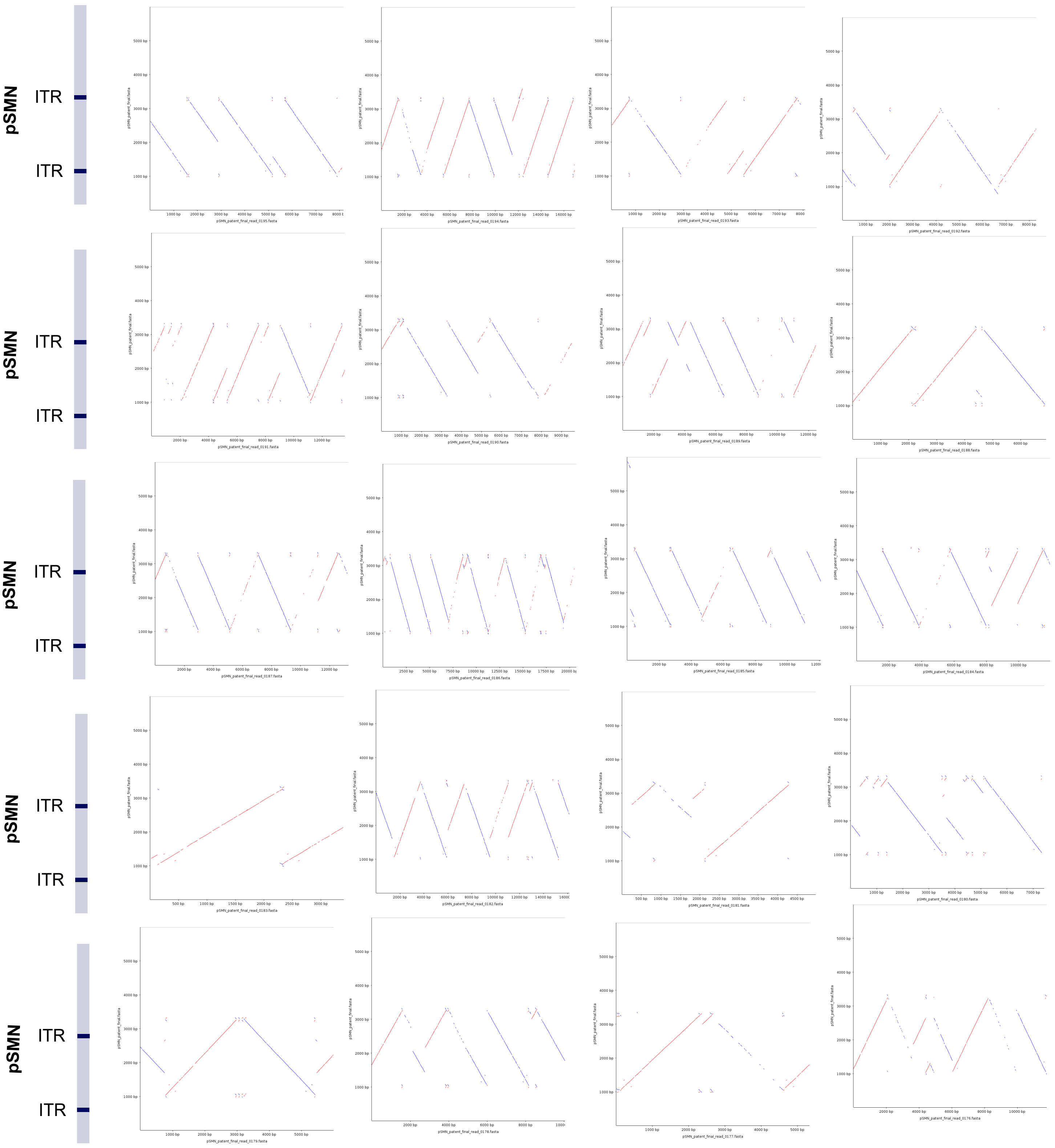
pSMN
ITR
ITR
pSMN
ITR
ITR
pSMN
ITR
ITR
pSMN
ITR
ITR
pSMN
ITR
ITR

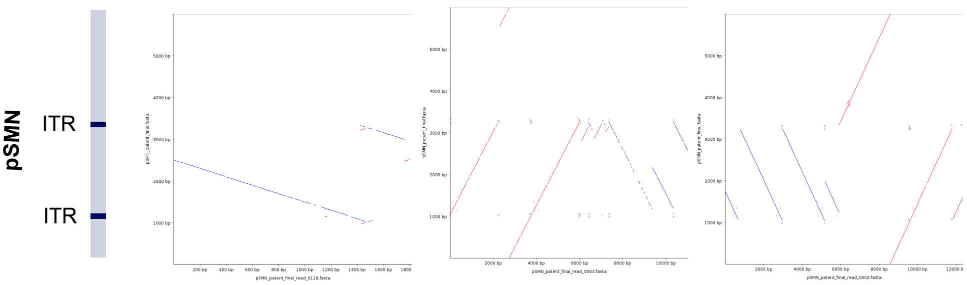
pSMN
ITR
ITR
pSMN_patent_final.fasta
5000 bp
4000 bp
3000 bp
2000 bp
1000 bp
200 bp
400 bp
600 bp
800 bp
1000 bp
1200 bp
1400 bp
1600 bp
1800
pSMN_patent_final_read_0118.fasta
2000 bp
4000 bp
6000 bp
8000 bp
10000 bp
pSMN_patent_final_read_0003.fasta
2000 bp
4000 bp
6000 bp
8000 bp
10000 bp
12000 b
pSMN_patent_final_read_0002.fasta

pSMN plasmid (y axis)
Duplex reads (unfiltered)

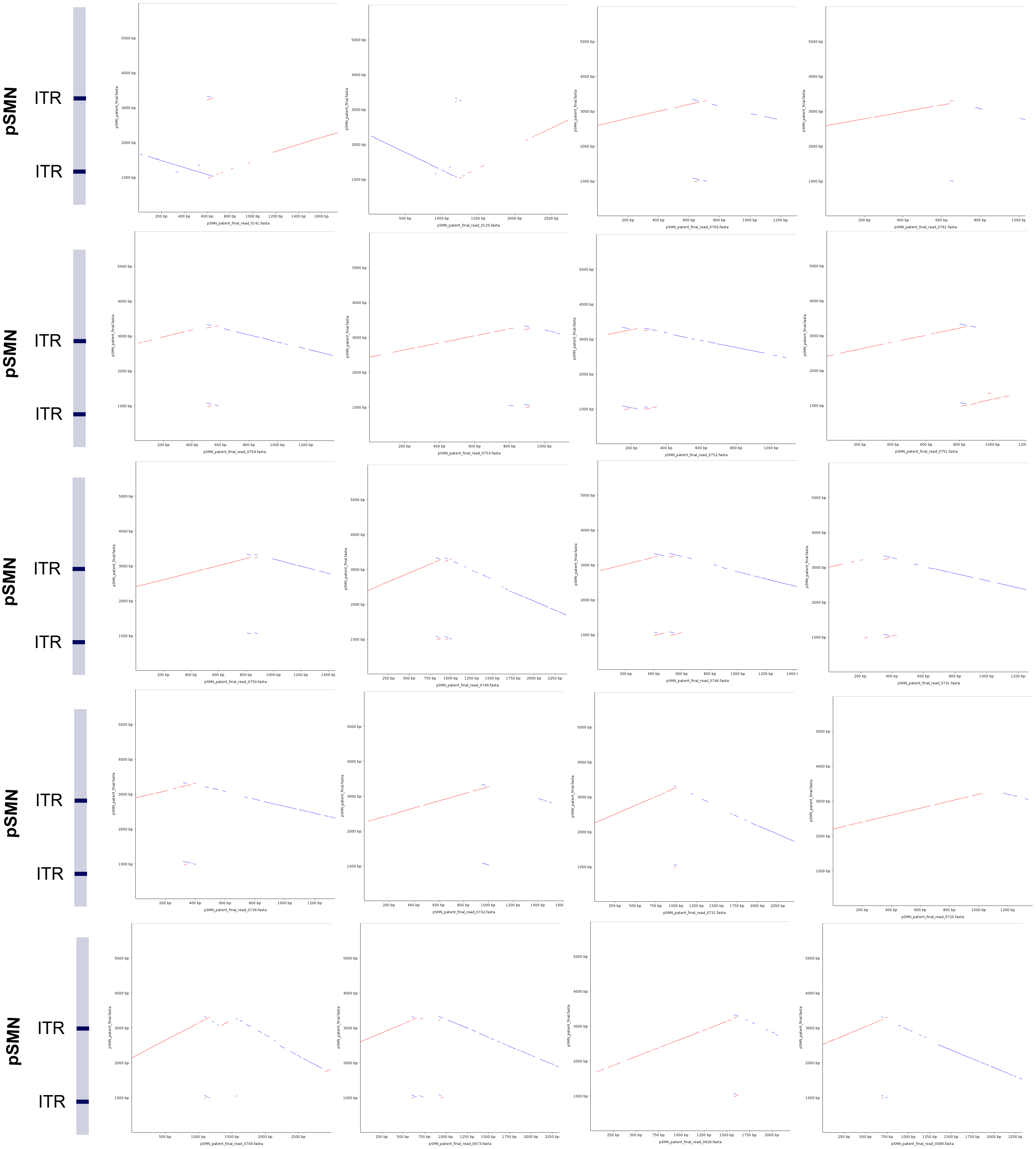

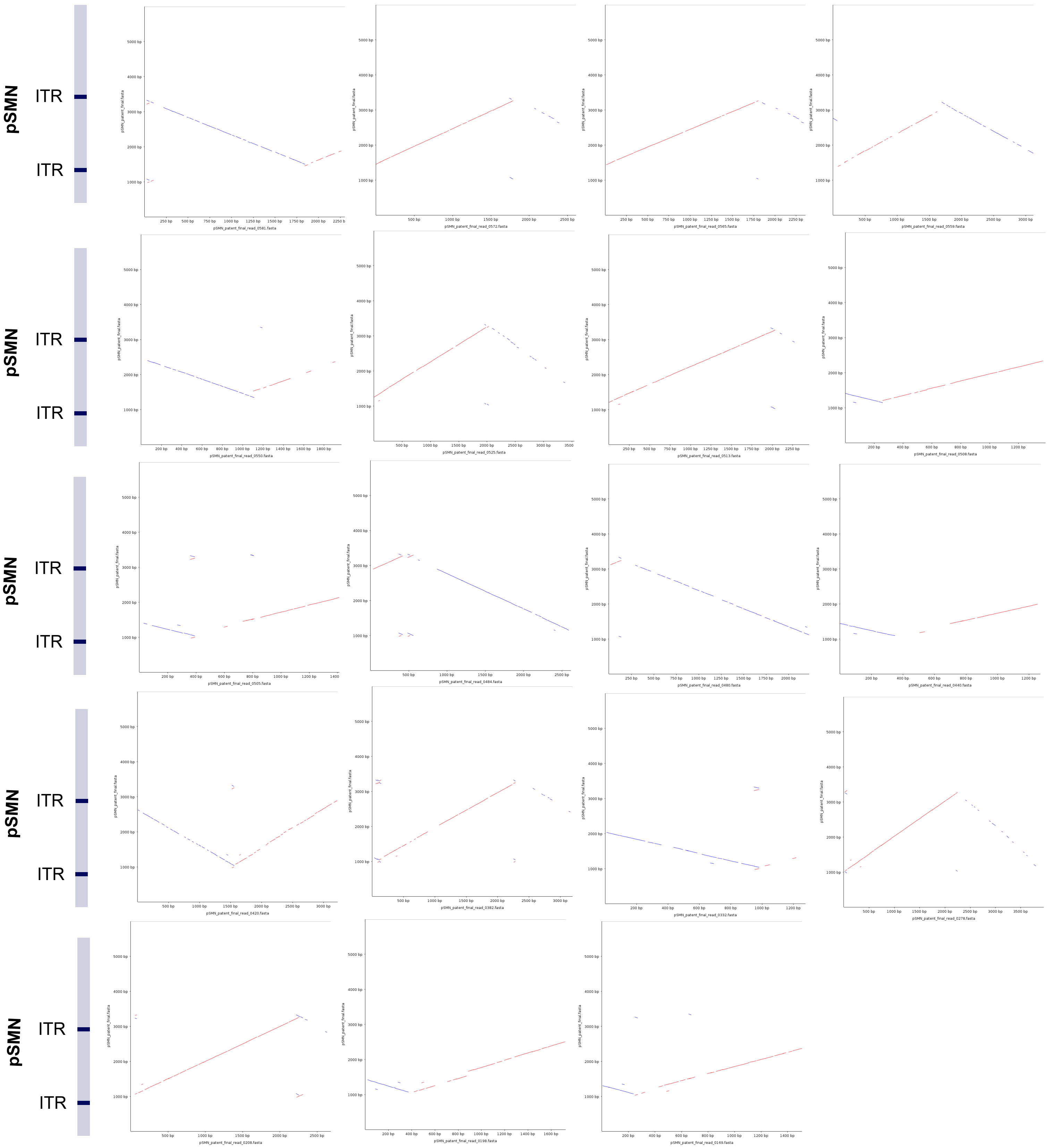
pSMN
ITR
ITR
pSMN
ITR
ITR
pSMN
ITR
ITR
pSMN
ITR
ITR
pSMN
ITR
ITR

pSMN plasmid (y axis)
Other reads (unfiltered)

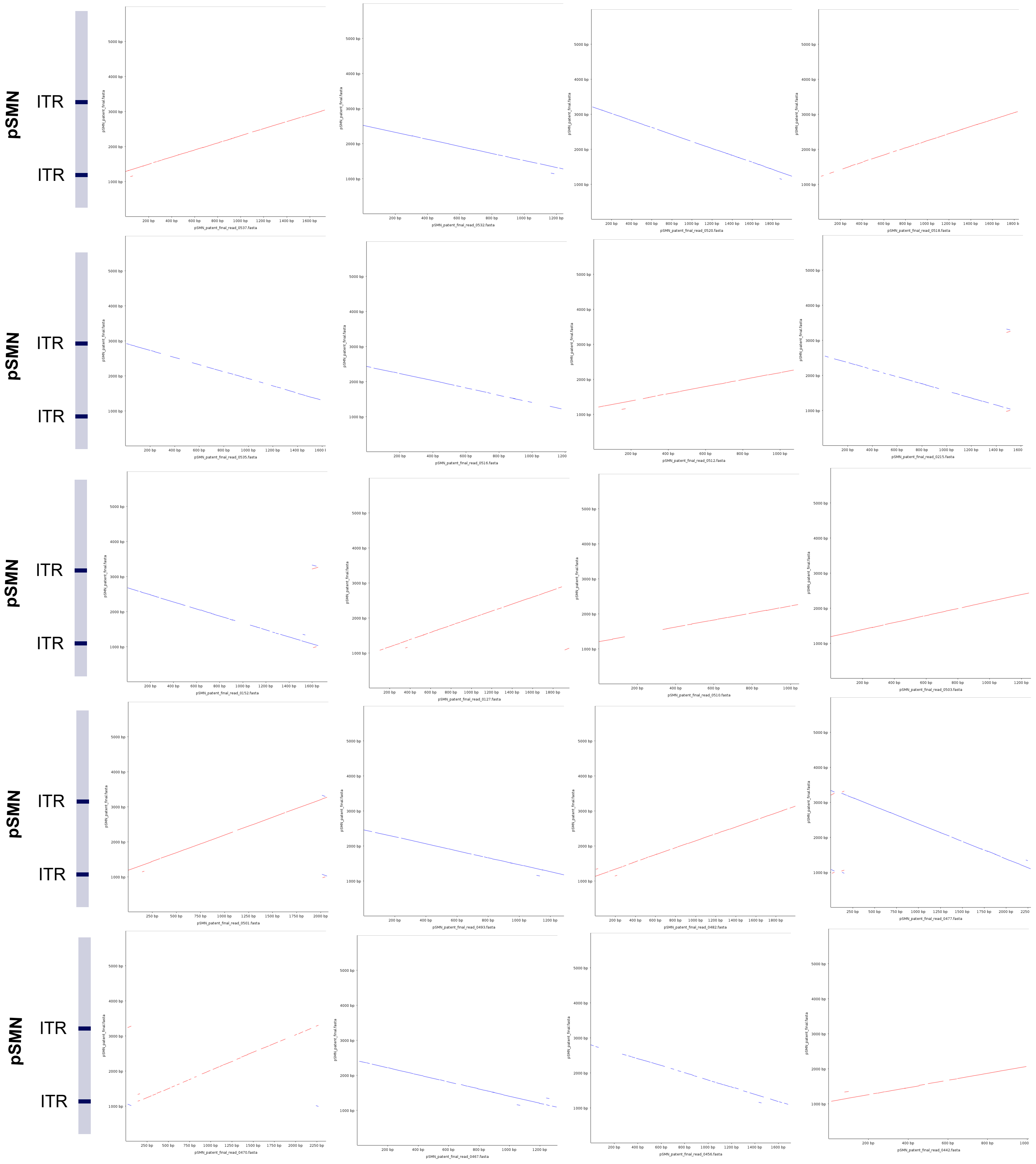

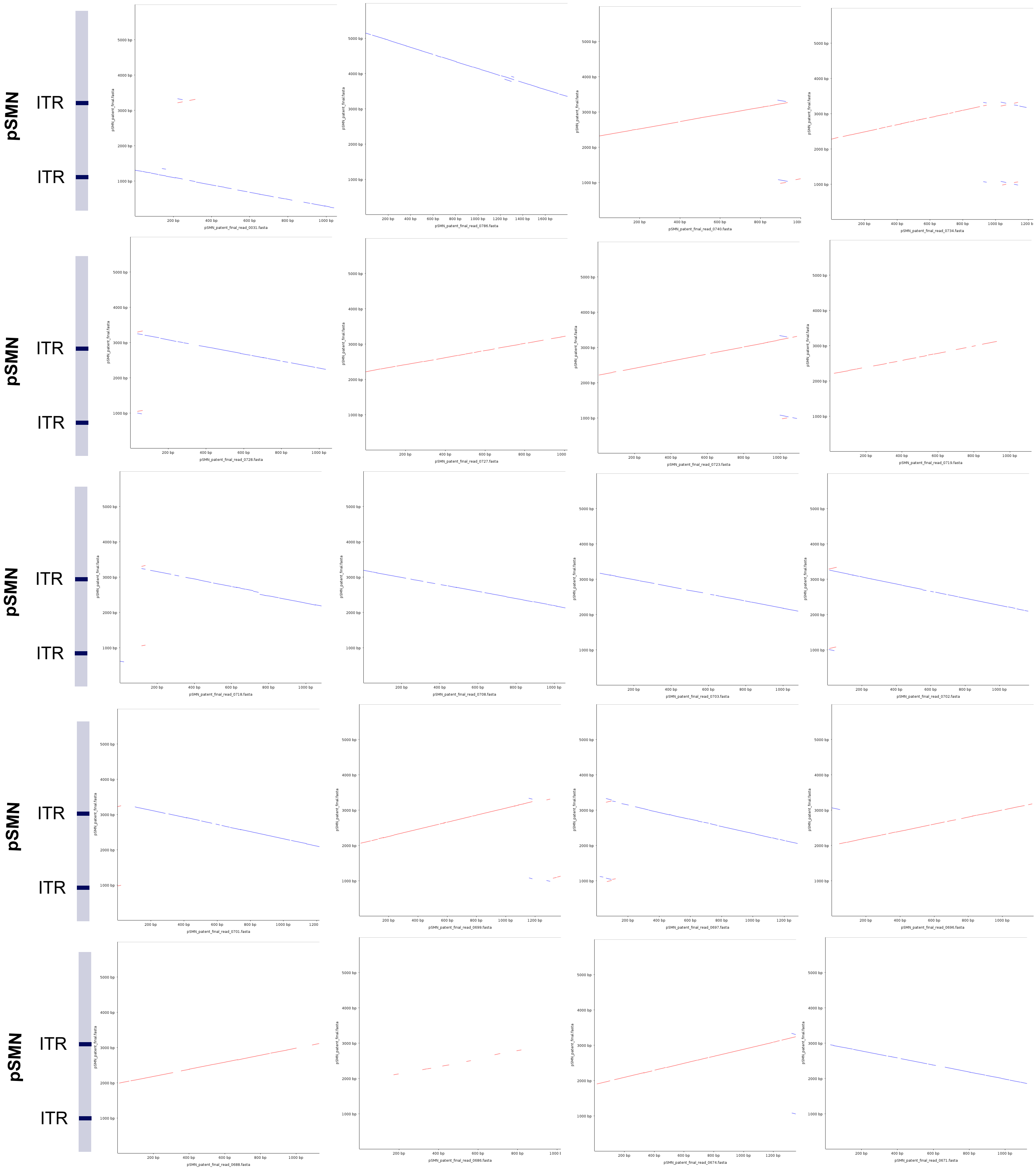
pSMN
ITR
ITR
pSMN
ITR
ITR
pSMN
ITR
ITR
pSMN
ITR
ITR
pSMN
ITR
ITR

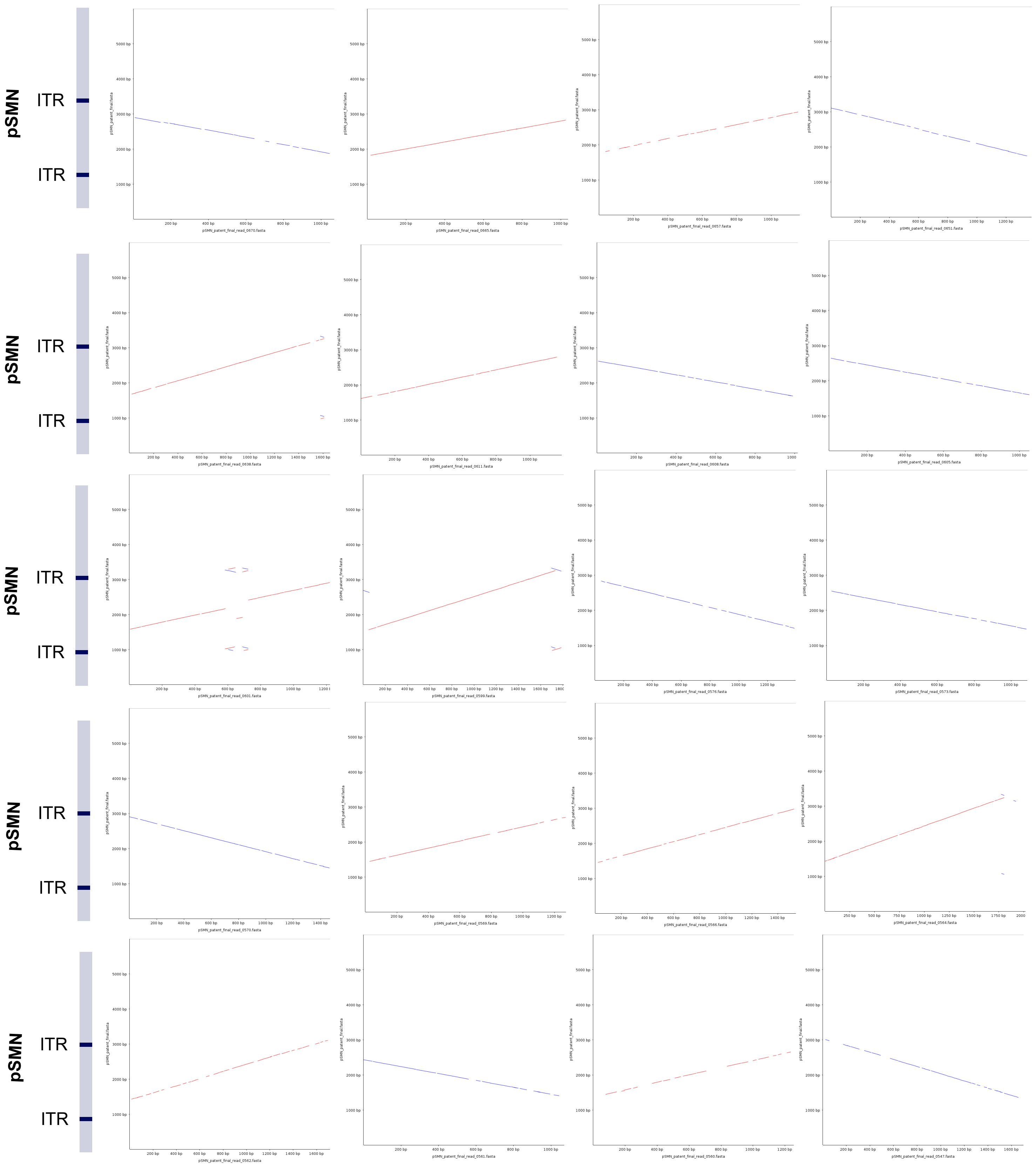

pSMN
ITR
ITR
pSMN_patent_final_read_0670.fasta
pSMN_patent_final_read_0665.fasta
pSMN_patent_final_read_0657.fasta
pSMN_patent_final_read_0651.fasta
pSMN_patent_final_read_0638.fasta
pSMN_patent_final_read_0611.fasta
pSMN_patent_final_read_0608.fasta
pSMN_patent_final_read_0605.fasta
pSMN_patent_final_read_0601.fasta
pSMN_patent_final_read_0599.fasta
pSMN_patent_final_read_0576.fasta
pSMN_patent_final_read_0573.fasta
pSMN_patent_final_read_0570.fasta
pSMN_patent_final_read_0569.fasta
pSMN_patent_final_read_0566.fasta
pSMN_patent_final_read_0564.fasta
pSMN_patent_final_read_0562.fasta
pSMN_patent_final_read_0561.fasta
pSMN_patent_final_read_0560.fasta
pSMN_patent_final_read_0547.fasta

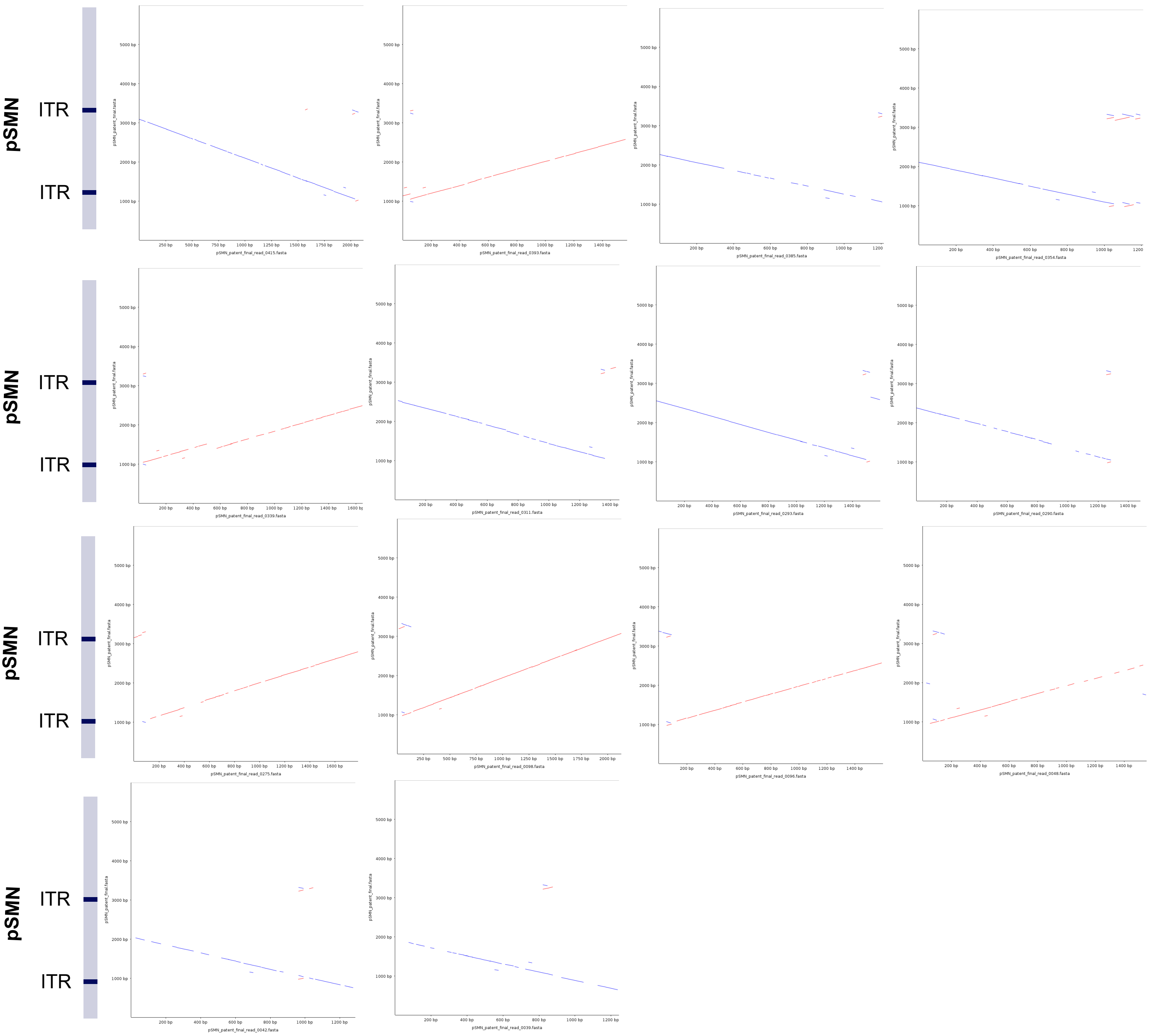
pSMN
ITR
ITR
pSMN_patent_final.fasta
pSMN_patent_final_read_0415.fasta
pSMN_patent_final_read_0393.fasta
pSMN_patent_final_read_0385.fasta
pSMN_patent_final_read_0354.fasta
pSMN
ITR
ITR
pSMN_patent_final_read_0339.fasta
pSMN_patent_final_read_0311.fasta
pSMN_patent_final_read_0293.fasta
pSMN_patent_final_read_0290.fasta
pSMN
ITR
ITR
pSMN_patent_final_read_0275.fasta
pSMN_patent_final_read_0098.fasta
pSMN_patent_final_read_0096.fasta
pSMN_patent_final_read_0048.fasta
pSMN
ITR
ITR
pSMN_patent_final_read_0042.fasta
pSMN_patent_final_read_0039.fasta

pAAV2/9 plasmid (y axis)
All reads

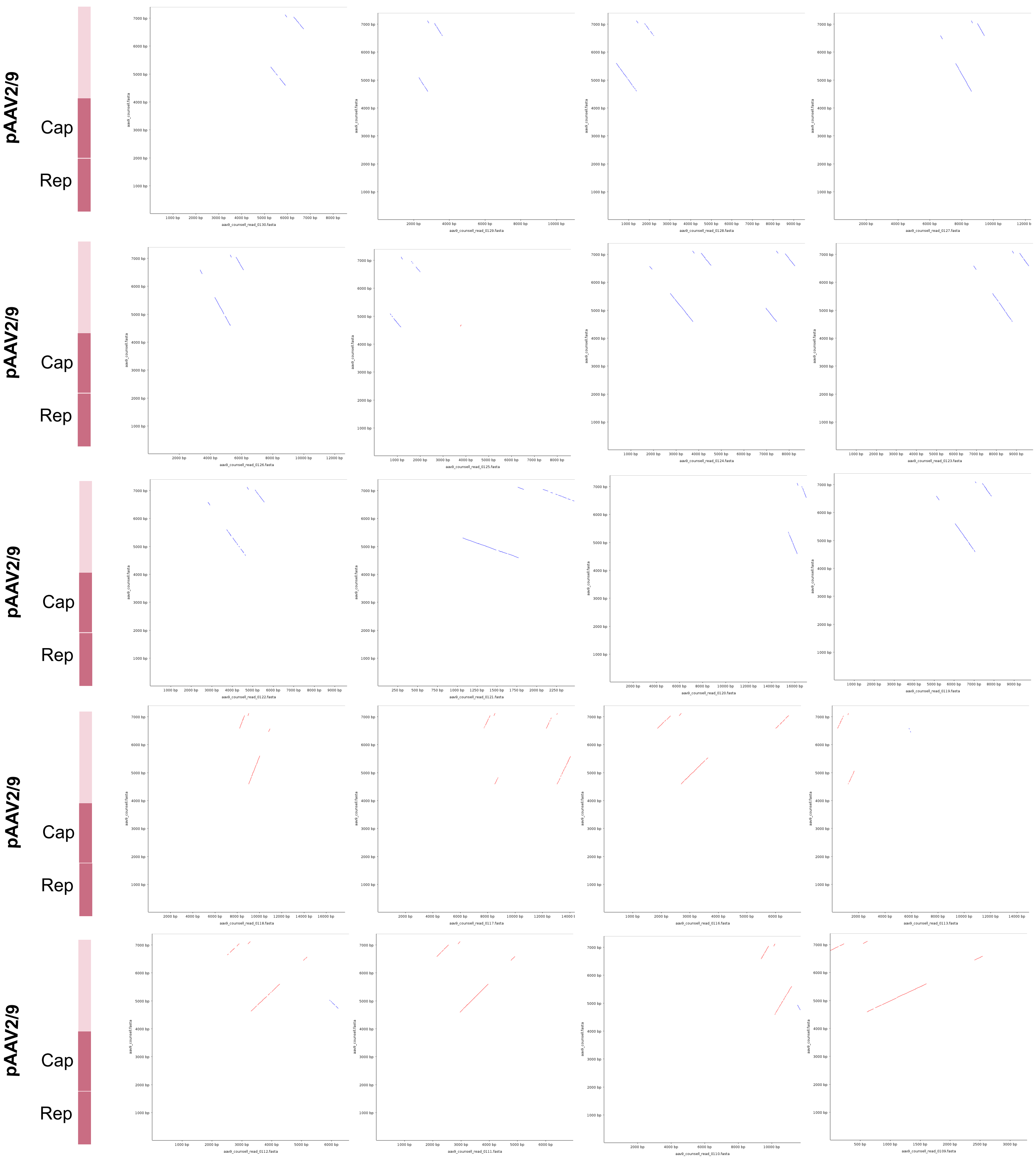

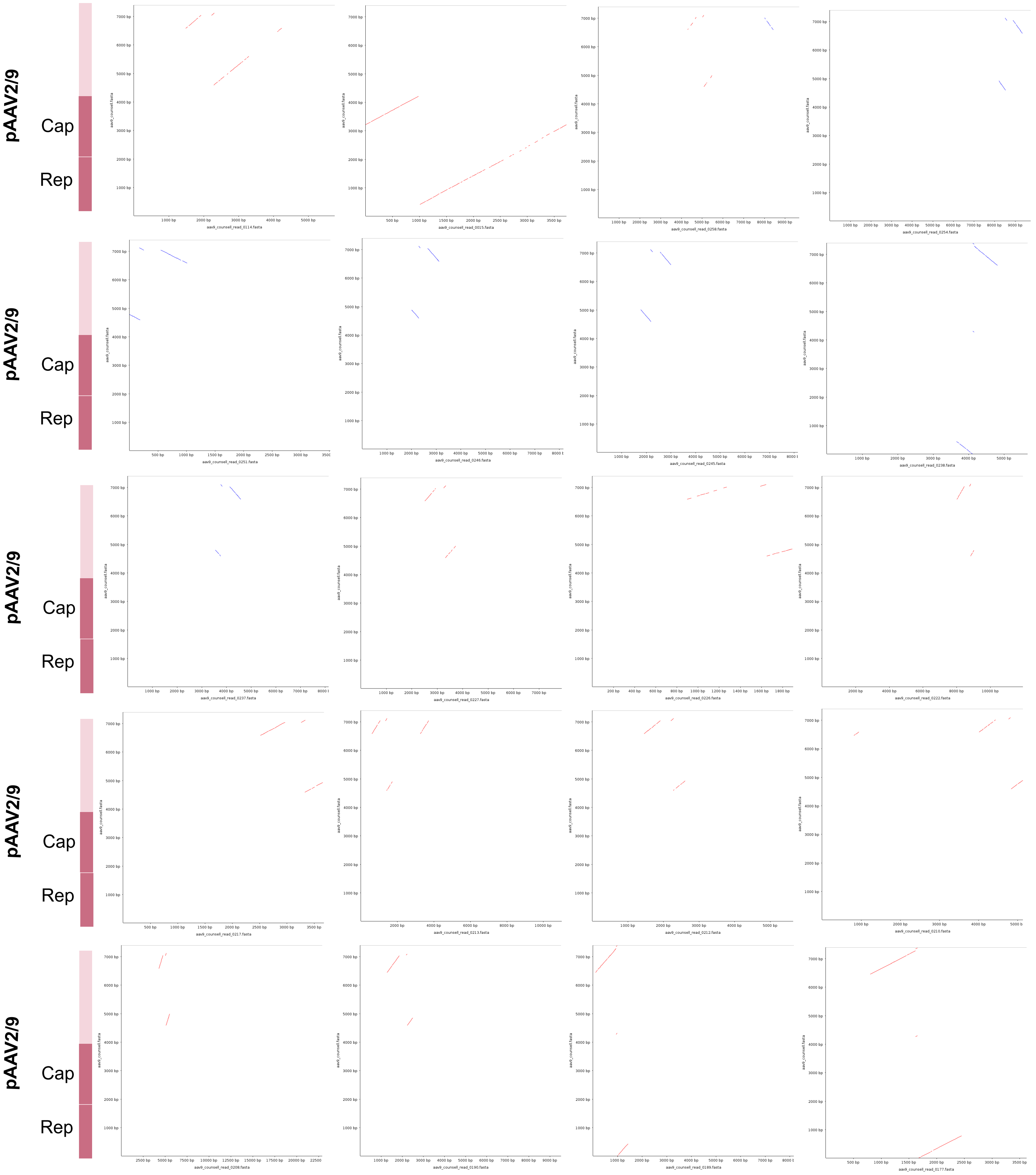
pAAV2/9
Cap
Rep
pAAV2/9
Cap
Rep
pAAV2/9
Cap
Rep
pAAV2/9
Cap
Rep
pAAV2/9
Cap
Rep

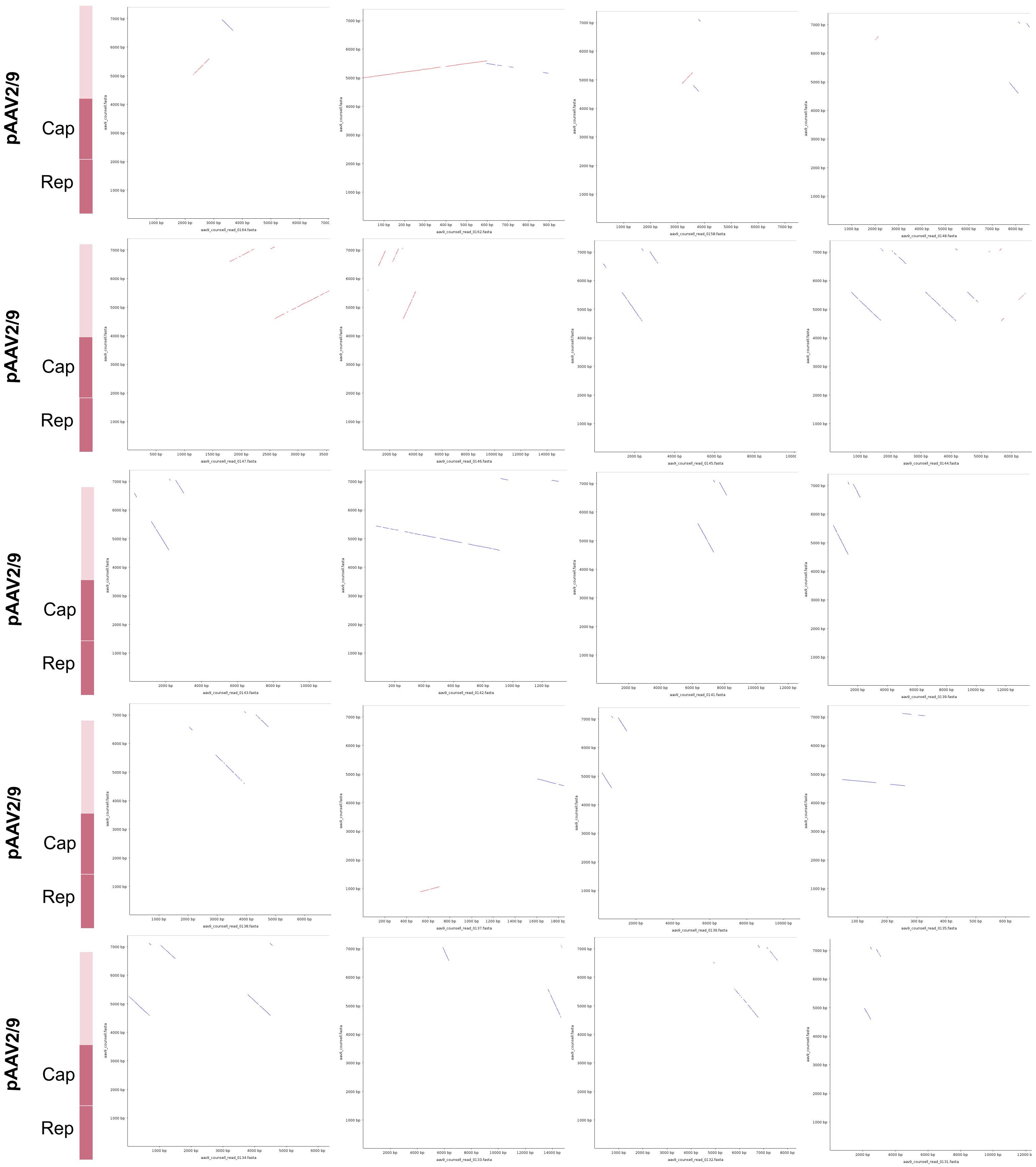
pAAV2/9
Cap
Rep
pAAV2/9
Cap
Rep
pAAV2/9
Cap
Rep
pAAV2/9
Cap
Rep
pAAV2/9
Cap
Rep

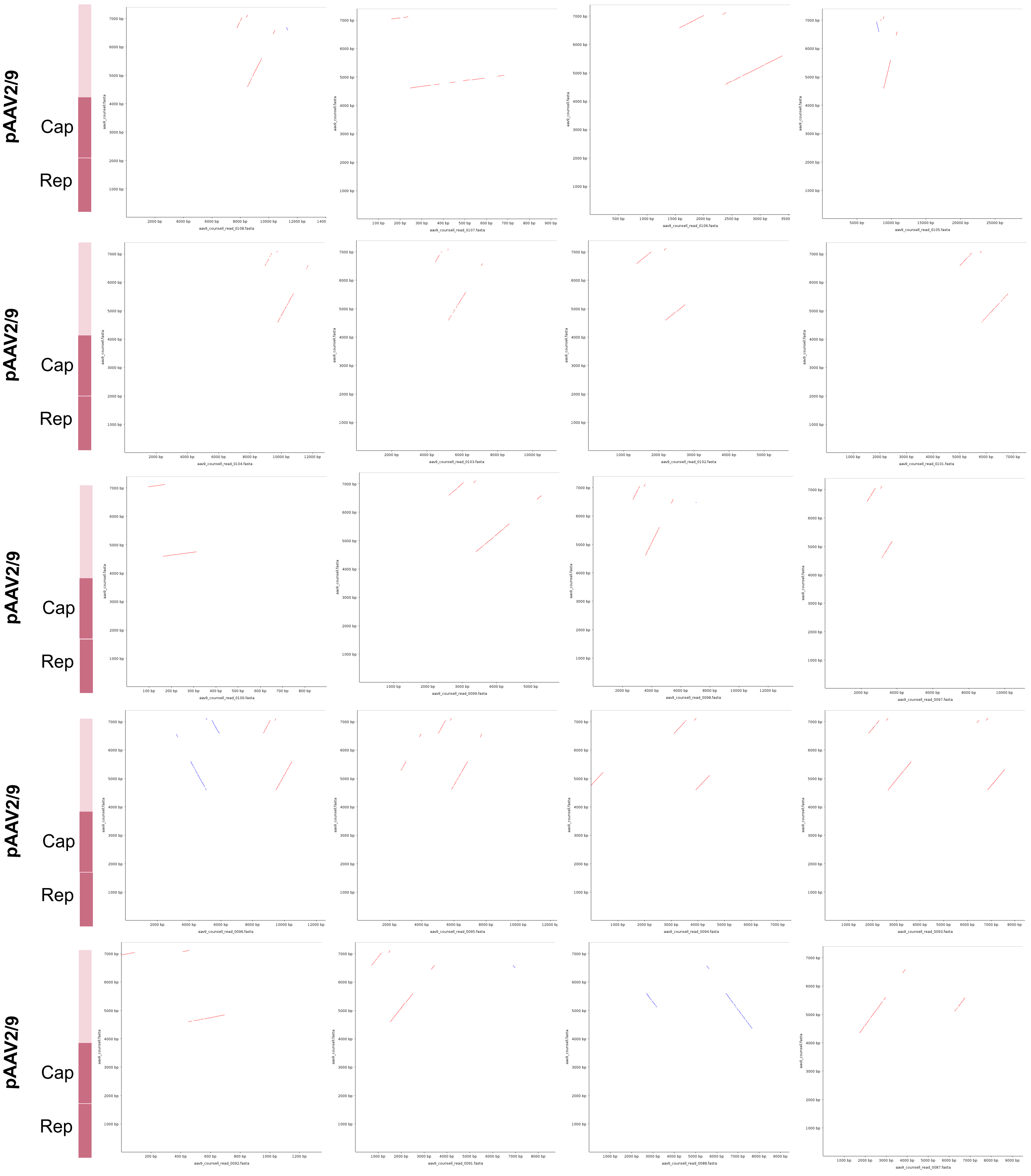
pAAV2/9
Cap
Rep
aav9_counsell.fasta
aav9_counsell_read_0108.fasta
aav9_counsell_read_0107.fasta
aav9_counsell_read_0106.fasta
aav9_counsell_read_0105.fasta
aav9_counsell_read_0104.fasta
aav9_counsell_read_0103.fasta
aav9_counsell_read_0102.fasta
aav9_counsell_read_0101.fasta
aav9_counsell_read_0100.fasta
aav9_counsell_read_0099.fasta
aav9_counsell_read_0098.fasta
aav9_counsell_read_0097.fasta
aav9_counsell_read_0096.fasta
aav9_counsell_read_0095.fasta
aav9_counsell_read_0094.fasta
aav9_counsell_read_0093.fasta
aav9_counsell_read_0092.fasta
aav9_counsell_read_0091.fasta
aav9_counsell_read_0088.fasta
aav9_counsell_read_0087.fasta

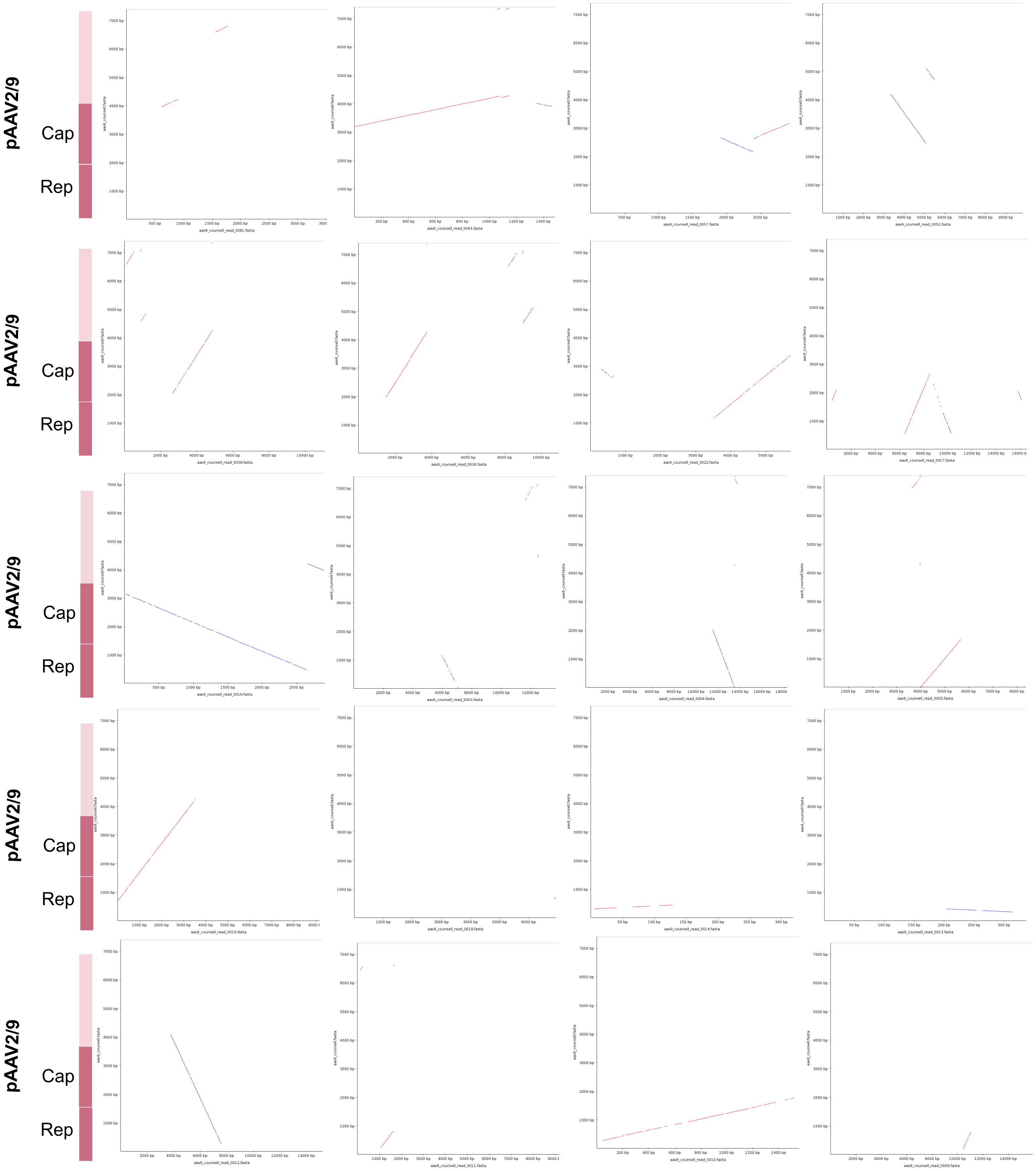
pAAV2/9
Cap
Rep
aav9_counsell_read_0081.fasta
aav9_counsell_read_0064.fasta
aav9_counsell_read_0057.fasta
aav9_counsell_read_0052.fasta
aav9_counsell_read_0038.fasta
aav9_counsell_read_0036.fasta
aav9_counsell_read_0022.fasta
aav9_counsell_read_0017.fasta
aav9_counsell_read_0016.fasta
aav9_counsell_read_0003.fasta
aav9_counsell_read_0004.fasta
aav9_counsell_read_0005.fasta
aav9_counsell_read_0019.fasta
aav9_counsell_read_0018.fasta
aav9_counsell_read_0014.fasta
aav9_counsell_read_0013.fasta
aav9_counsell_read_0012.fasta
aav9_counsell_read_0011.fasta
aav9_counsell_read_0010.fasta
aav9_counsell_read_0009.fasta
aav9_counsell.fasta

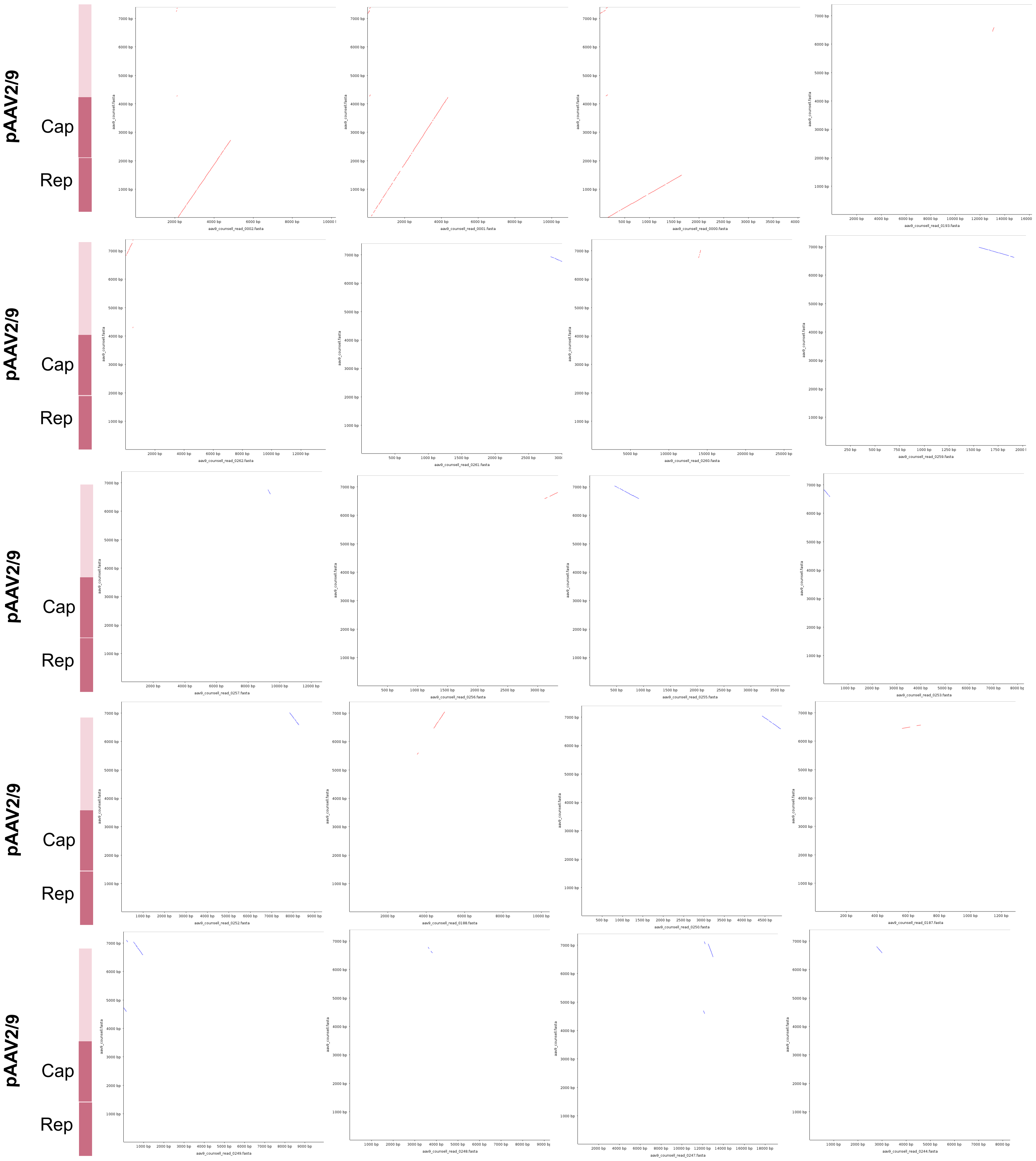
pAAV2/9
Cap
Rep
aav9_counsell.fasta
aav9_counsell_read_0002.fasta
aav9_counsell_read_0001.fasta
aav9_counsell_read_0000.fasta
aav9_counsell_read_0193.fasta
aav9_counsell_read_0262.fasta
aav9_counsell_read_0261.fasta
aav9_counsell_read_0260.fasta
aav9_counsell_read_0259.fasta
aav9_counsell_read_0257.fasta
aav9_counsell_read_0256.fasta
aav9_counsell_read_0255.fasta
aav9_counsell_read_0253.fasta
aav9_counsell_read_0252.fasta
aav9_counsell_read_0188.fasta
aav9_counsell_read_0250.fasta
aav9_counsell_read_0187.fasta
aav9_counsell_read_0249.fasta
aav9_counsell_read_0248.fasta
aav9_counsell_read_0247.fasta
aav9_counsell_read_0244.fasta

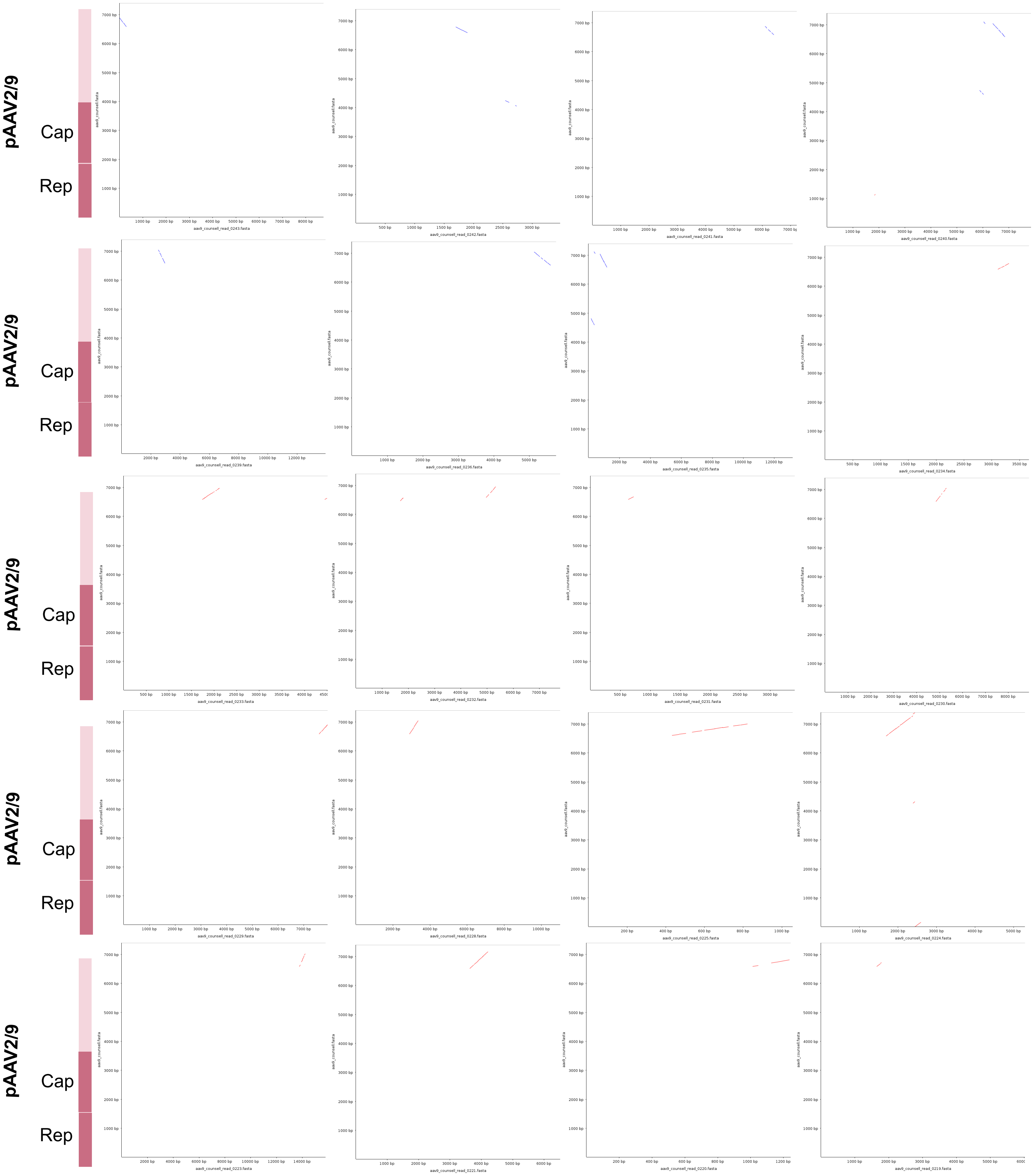
pAAV2/9
Cap
Rep
aav9_counsell.fasta
aav9_counsell_read_0243.fasta
aav9_counsell_read_0242.fasta
aav9_counsell_read_0241.fasta
aav9_counsell_read_0240.fasta
aav9_counsell_read_0239.fasta
aav9_counsell_read_0236.fasta
aav9_counsell_read_0235.fasta
aav9_counsell_read_0234.fasta
aav9_counsell_read_0233.fasta
aav9_counsell_read_0232.fasta
aav9_counsell_read_0231.fasta
aav9_counsell_read_0230.fasta
aav9_counsell_read_0229.fasta
aav9_counsell_read_0228.fasta
aav9_counsell_read_0225.fasta
aav9_counsell_read_0224.fasta
aav9_counsell_read_0223.fasta
aav9_counsell_read_0221.fasta
aav9_counsell_read_0220.fasta
aav9_counsell_read_0219.fasta

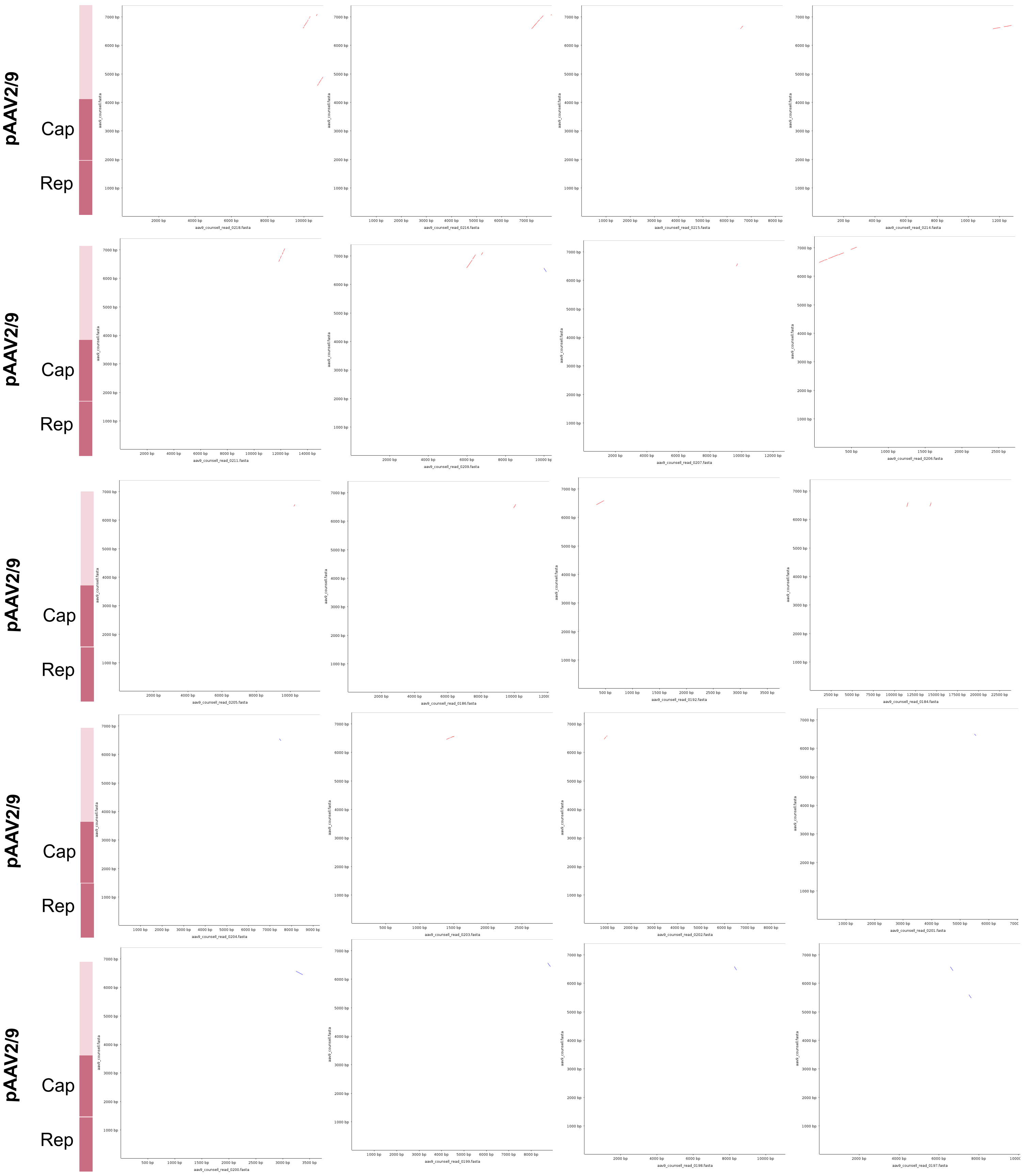

pAAV2/9
Cap
Rep
aav9_counsell.fasta
aav9_counsell_read_0218.fasta
aav9_counsell_read_0216.fasta
aav9_counsell_read_0215.fasta
aav9_counsell_read_0214.fasta
pAAV2/9
Cap
Rep
aav9_counsell_read_0211.fasta
aav9_counsell_read_0209.fasta
aav9_counsell_read_0207.fasta
aav9_counsell_read_0206.fasta
pAAV2/9
Cap
Rep
aav9_counsell_read_0205.fasta
aav9_counsell_read_0186.fasta
aav9_counsell_read_0192.fasta
aav9_counsell_read_0184.fasta
pAAV2/9
Cap
Rep
aav9_counsell_read_0204.fasta
aav9_counsell_read_0203.fasta
aav9_counsell_read_0202.fasta
aav9_counsell_read_0201.fasta
pAAV2/9
Cap
Rep
aav9_counsell_read_0200.fasta
aav9_counsell_read_0199.fasta
aav9_counsell_read_0198.fasta
aav9_counsell_read_0197.fasta

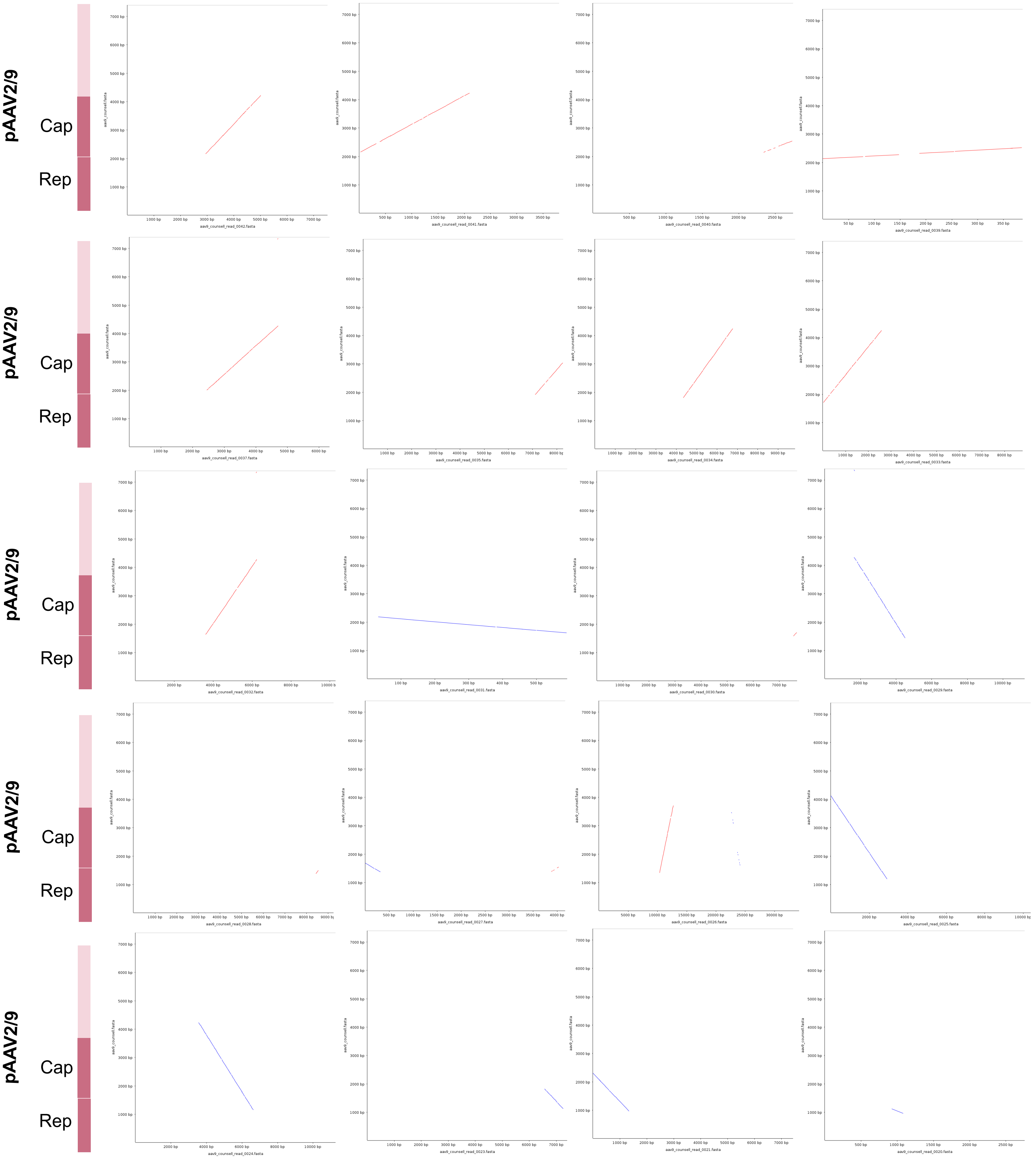
pAAV2/9
Cap
Rep
aav9_counsell_read_0042.fasta
aav9_counsell_read_0041.fasta
aav9_counsell_read_0040.fasta
aav9_counsell_read_0039.fasta
aav9_counsell_read_0037.fasta
aav9_counsell_read_0035.fasta
aav9_counsell_read_0034.fasta
aav9_counsell_read_0033.fasta
aav9_counsell_read_0032.fasta
aav9_counsell_read_0031.fasta
aav9_counsell_read_0030.fasta
aav9_counsell_read_0029.fasta
aav9_counsell_read_0028.fasta
aav9_counsell_read_0027.fasta
aav9_counsell_read_0026.fasta
aav9_counsell_read_0025.fasta
aav9_counsell_read_0024.fasta
aav9_counsell_read_0023.fasta
aav9_counsell_read_0021.fasta
aav9_counsell_read_0020.fasta
aav9_counsell.fasta

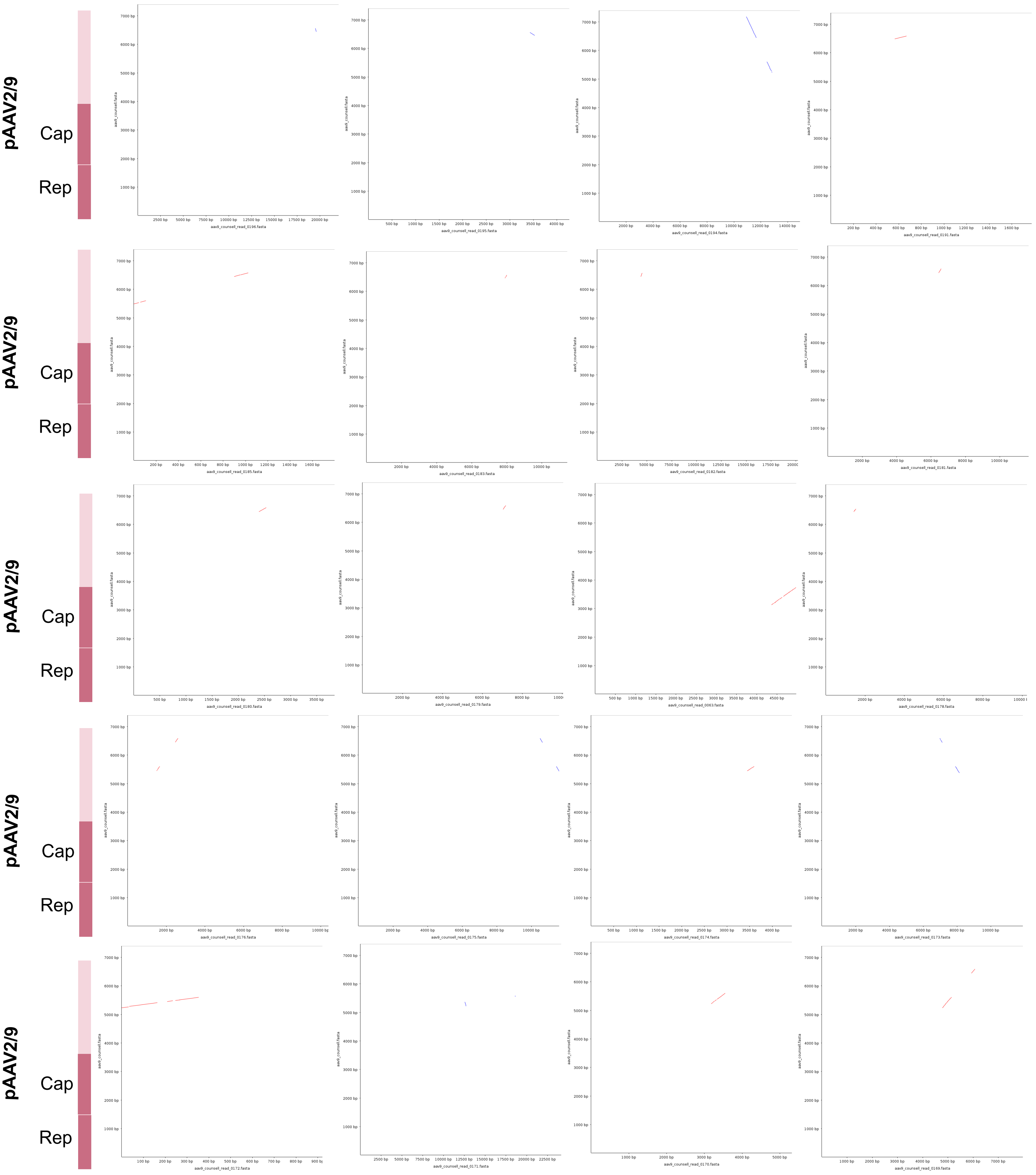
pAAV2/9
Cap
Rep
aav9_counsell.fasta
aav9_counsell_read_0196.fasta
aav9_counsell_read_0195.fasta
aav9_counsell_read_0194.fasta
aav9_counsell_read_0191.fasta
aav9_counsell_read_0185.fasta
aav9_counsell_read_0183.fasta
aav9_counsell_read_0182.fasta
aav9_counsell_read_0181.fasta
aav9_counsell_read_0180.fasta
aav9_counsell_read_0179.fasta
aav9_counsell_read_0063.fasta
aav9_counsell_read_0178.fasta
aav9_counsell_read_0176.fasta
aav9_counsell_read_0175.fasta
aav9_counsell_read_0174.fasta
aav9_counsell_read_0173.fasta
aav9_counsell_read_0172.fasta
aav9_counsell_read_0171.fasta
aav9_counsell_read_0170.fasta
aav9_counsell_read_0169.fasta

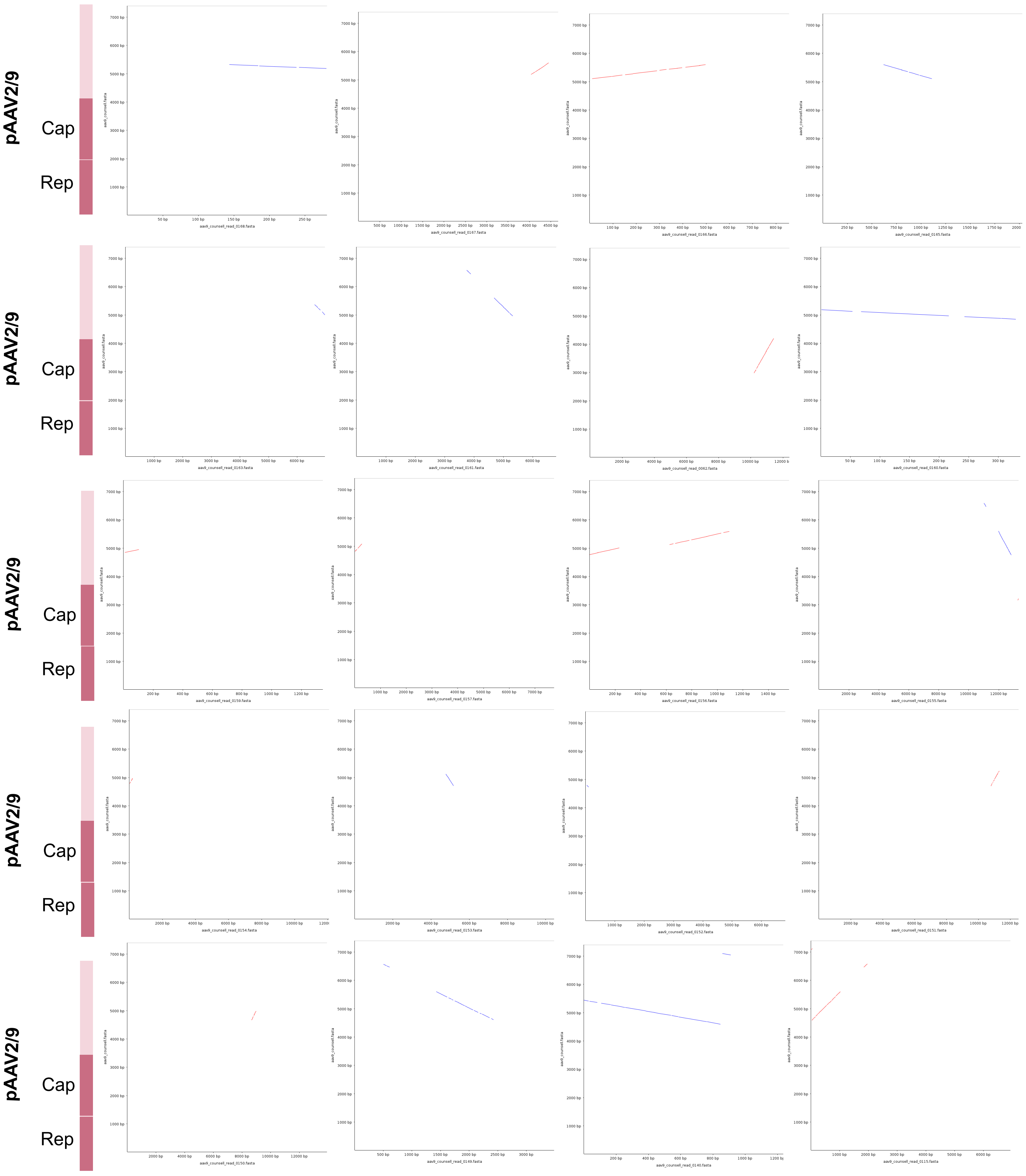
pAAV2/9
Cap
Rep
aav9_counsell_read_0168.fasta
aav9_counsell_read_0167.fasta
aav9_counsell_read_0166.fasta
aav9_counsell_read_0165.fasta
pAAV2/9
Cap
Rep
aav9_counsell_read_0163.fasta
aav9_counsell_read_0161.fasta
aav9_counsell_read_0062.fasta
aav9_counsell_read_0160.fasta
pAAV2/9
Cap
Rep
aav9_counsell_read_0159.fasta
aav9_counsell_read_0157.fasta
aav9_counsell_read_0156.fasta
aav9_counsell_read_0155.fasta
pAAV2/9
Cap
Rep
aav9_counsell_read_0154.fasta
aav9_counsell_read_0153.fasta
aav9_counsell_read_0152.fasta
aav9_counsell_read_0151.fasta
pAAV2/9
Cap
Rep
aav9_counsell_read_0150.fasta
aav9_counsell_read_0149.fasta
aav9_counsell_read_0140.fasta
aav9_counsell_read_0115.fasta

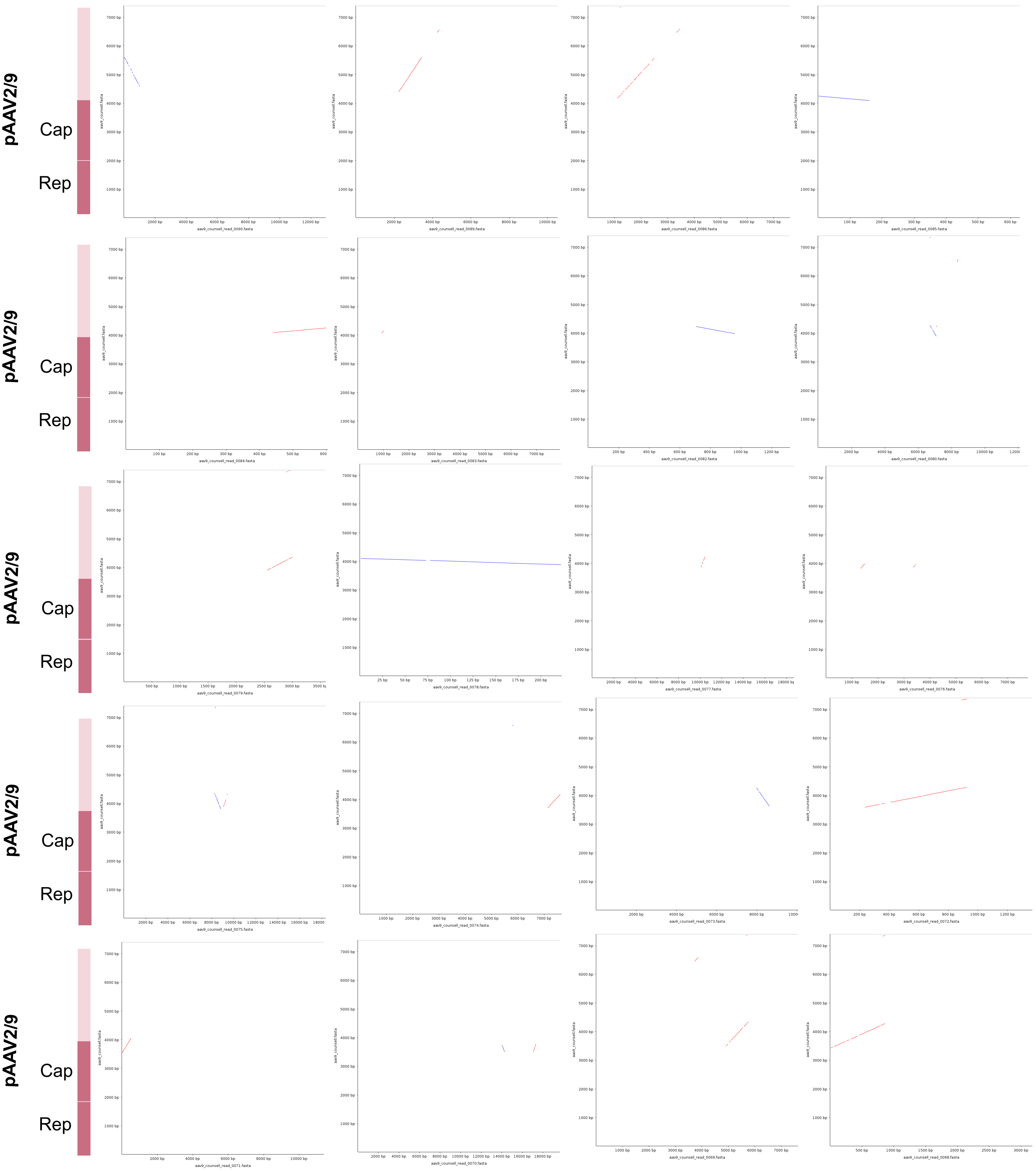
pAAV2/9
Cap
Rep
aav9_counsell_read_0090.fasta
aav9_counsell_read_0089.fasta
aav9_counsell_read_0086.fasta
aav9_counsell_read_0085.fasta
pAAV2/9
Cap
Rep
aav9_counsell_read_0084.fasta
aav9_counsell_read_0083.fasta
aav9_counsell_read_0082.fasta
aav9_counsell_read_0080.fasta
pAAV2/9
Cap
Rep
aav9_counsell_read_0079.fasta
aav9_counsell_read_0078.fasta
aav9_counsell_read_0077.fasta
aav9_counsell_read_0076.fasta
pAAV2/9
Cap
Rep
aav9_counsell_read_0075.fasta
aav9_counsell_read_0074.fasta
aav9_counsell_read_0073.fasta
aav9_counsell_read_0072.fasta
pAAV2/9
Cap
Rep
aav9_counsell_read_0071.fasta
aav9_counsell_read_0070.fasta
aav9_counsell_read_0069.fasta
aav9_counsell_read_0068.fasta

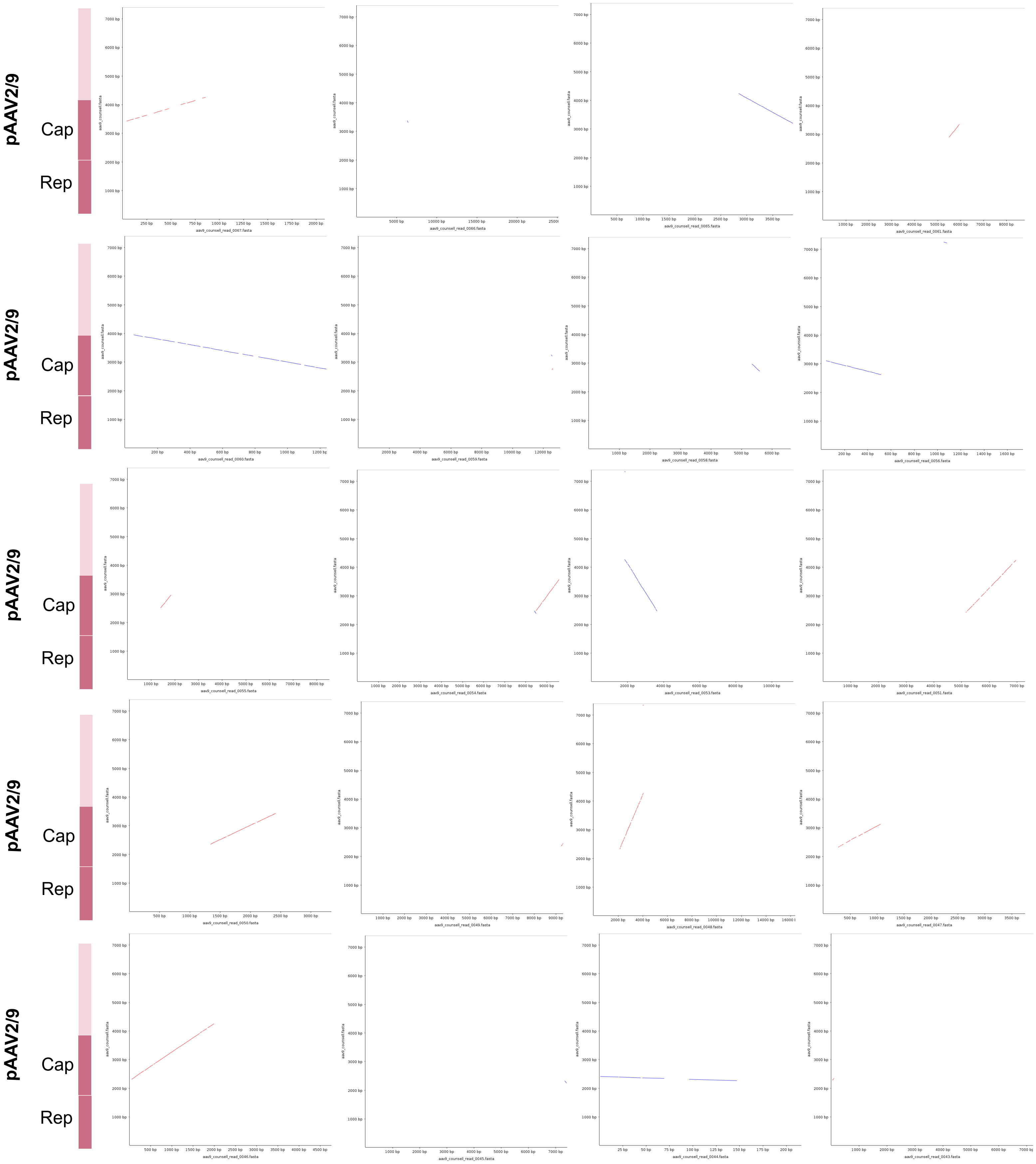
pAAV2/9
Cap
Rep
aav9_counsell.fasta
aav9_counsell_read_0067.fasta
aav9_counsell_read_0066.fasta
aav9_counsell_read_0065.fasta
aav9_counsell_read_0061.fasta
aav9_counsell_read_0060.fasta
aav9_counsell_read_0059.fasta
aav9_counsell_read_0058.fasta
aav9_counsell_read_0056.fasta
aav9_counsell_read_0055.fasta
aav9_counsell_read_0054.fasta
aav9_counsell_read_0053.fasta
aav9_counsell_read_0051.fasta
aav9_counsell_read_0050.fasta
aav9_counsell_read_0049.fasta
aav9_counsell_read_0048.fasta
aav9_counsell_read_0047.fasta
aav9_counsell_read_0046.fasta
aav9_counsell_read_0045.fasta
aav9_counsell_read_0044.fasta
aav9_counsell_read_0043.fasta

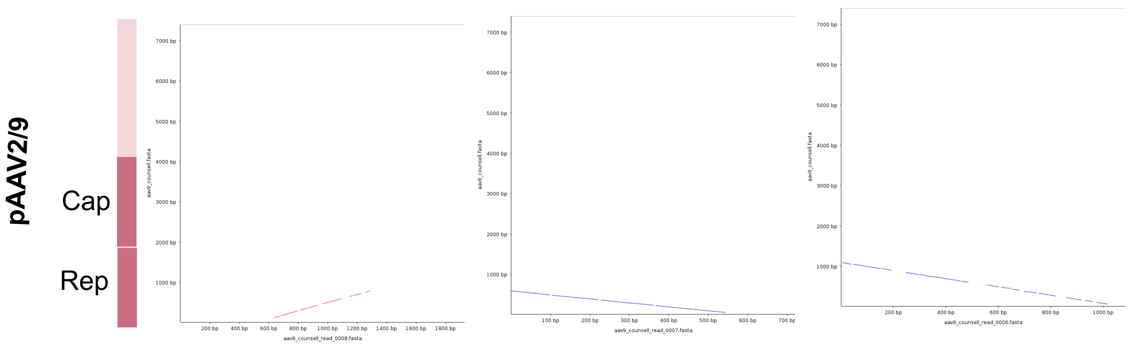
pAAV2/9
Cap
Rep
aav9_counsell_read_0008.fasta
aav9_counsell_read_0007.fasta
aav9_counsell_read_0006.fasta

pHelper plasmid (y axis)
All reads

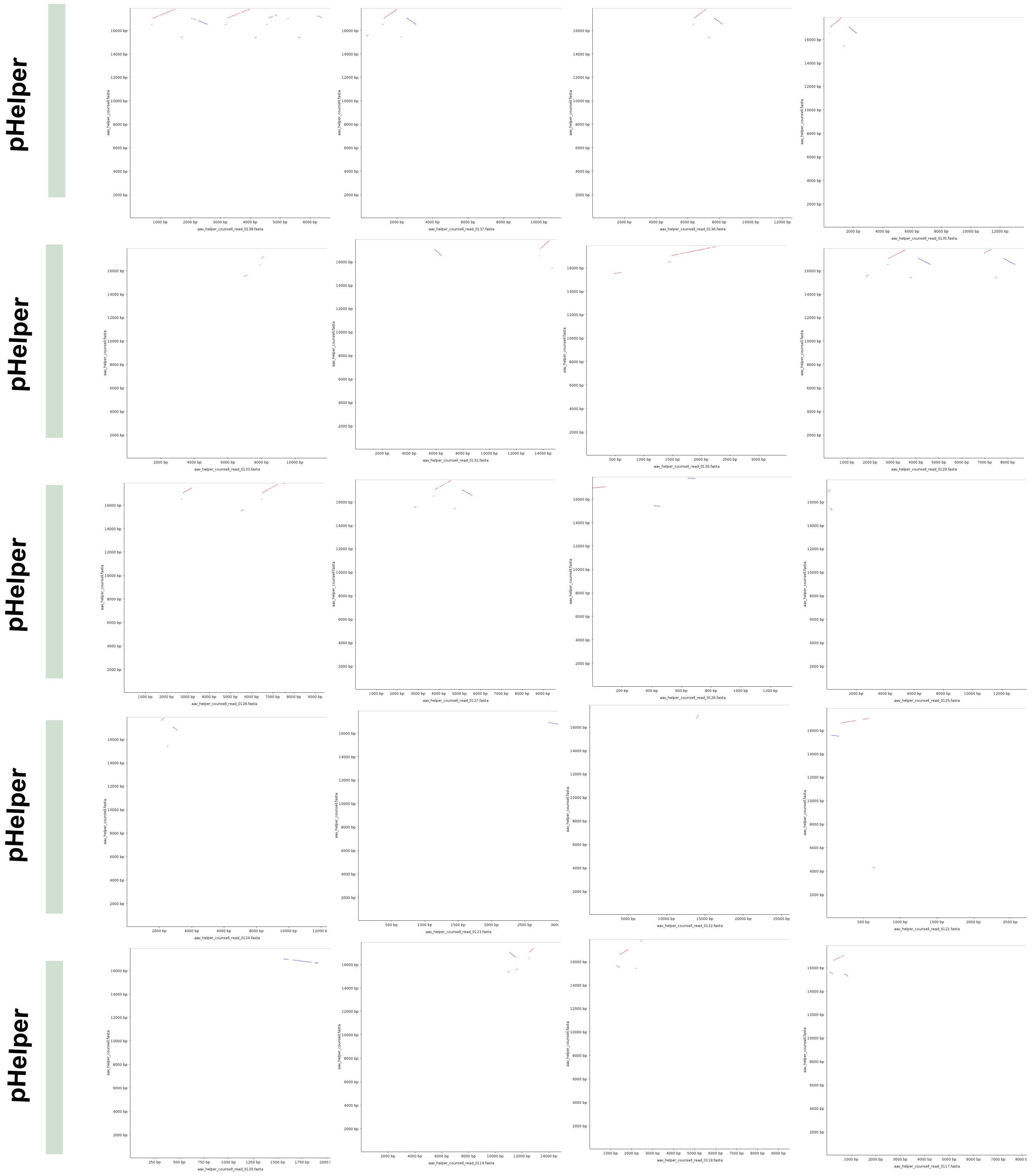

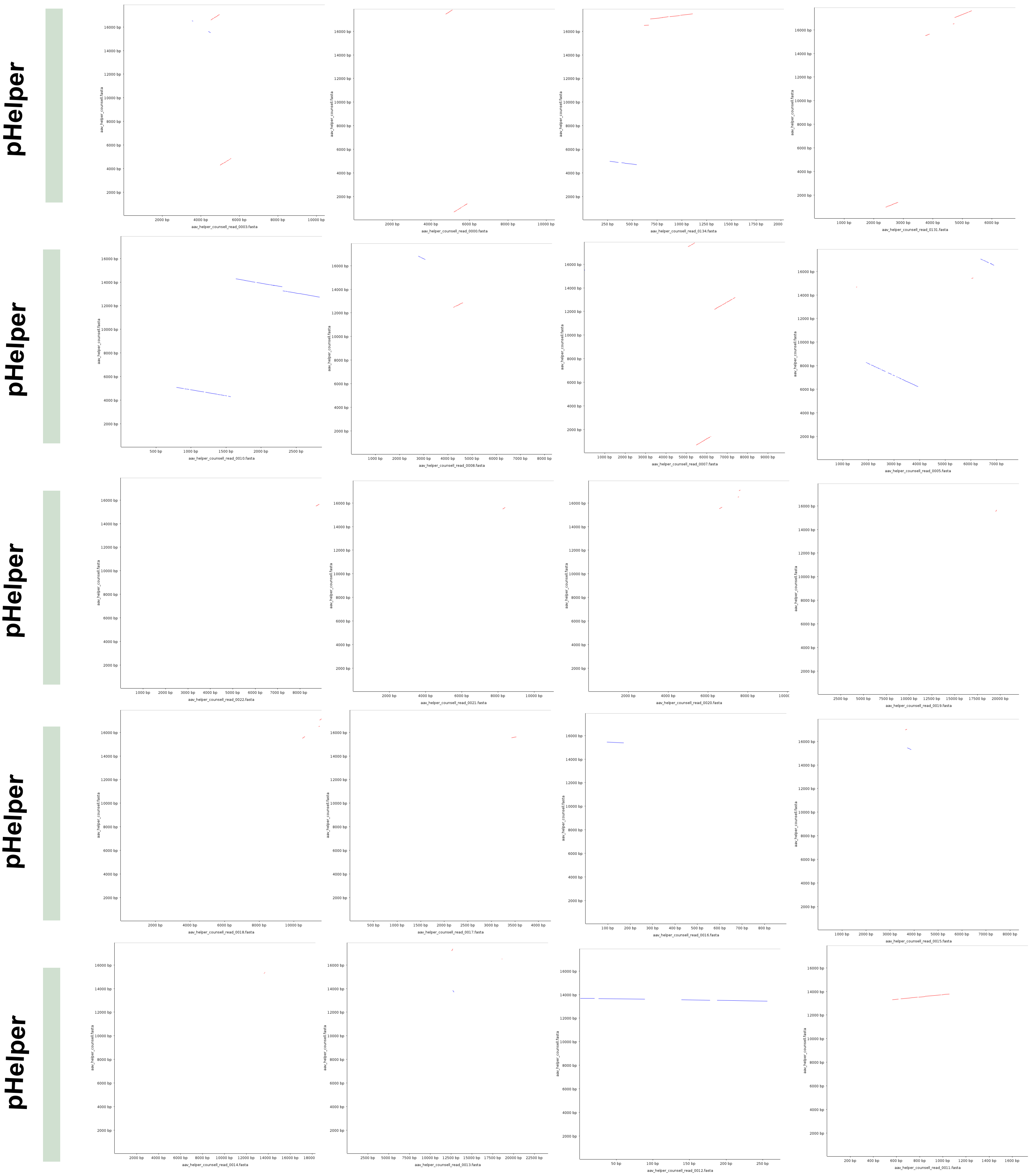
pHelper
pHelper
pHelper
pHelper
pHelper
aav_helper_counsell.fasta
aav_helper_counsell_read_0003.fasta
aav_helper_counsell_read_0000.fasta
aav_helper_counsell_read_0134.fasta
aav_helper_counsell_read_0131.fasta
aav_helper_counsell_read_0010.fasta
aav_helper_counsell_read_0008.fasta
aav_helper_counsell_read_0007.fasta
aav_helper_counsell_read_0005.fasta
aav_helper_counsell_read_0022.fasta
aav_helper_counsell_read_0021.fasta
aav_helper_counsell_read_0020.fasta
aav_helper_counsell_read_0019.fasta
aav_helper_counsell_read_0018.fasta
aav_helper_counsell_read_0017.fasta
aav_helper_counsell_read_0016.fasta
aav_helper_counsell_read_0015.fasta
aav_helper_counsell_read_0014.fasta
aav_helper_counsell_read_0013.fasta
aav_helper_counsell_read_0012.fasta
aav_helper_counsell_read_0011.fasta

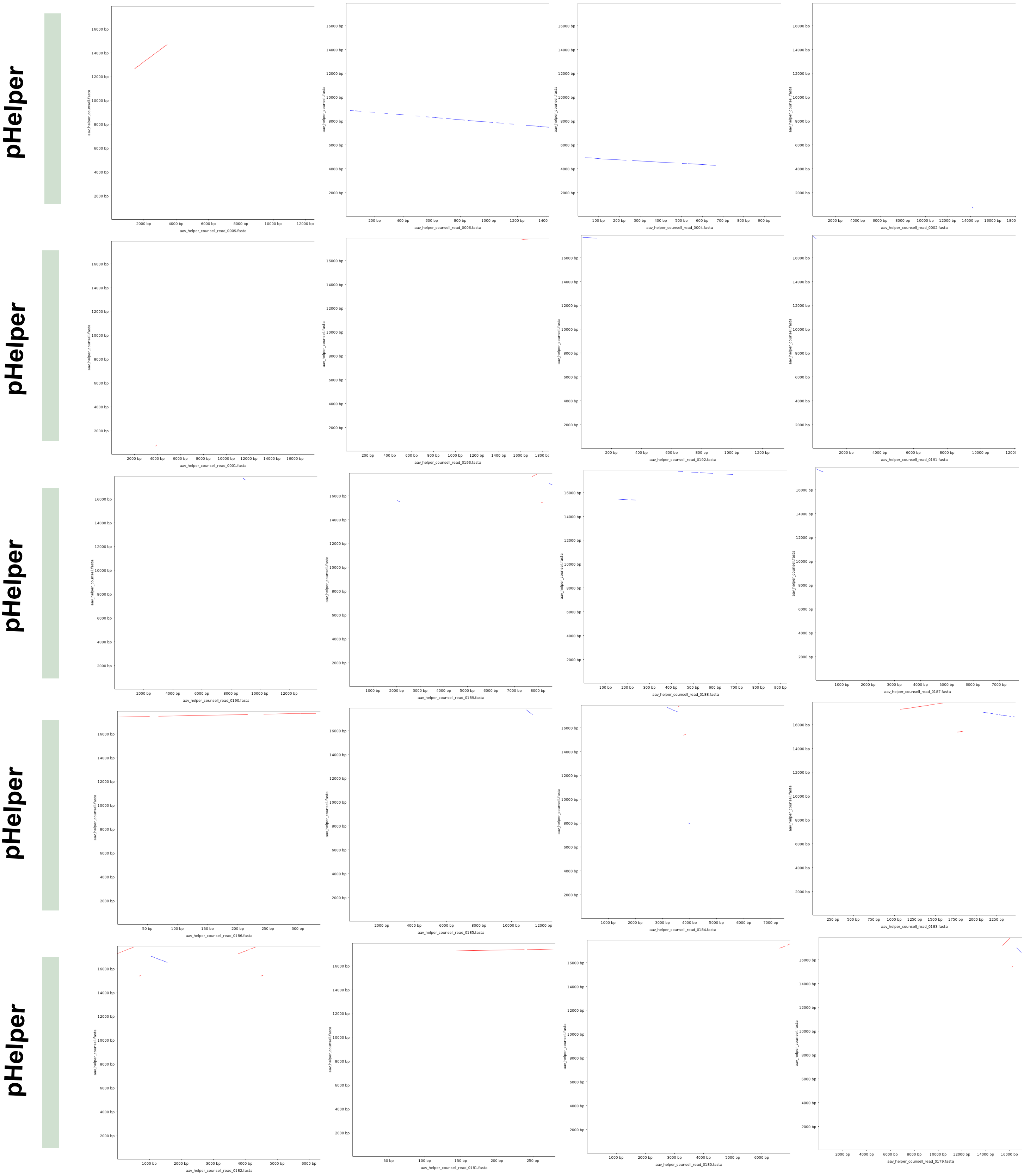
pHelper
pHelper
pHelper
pHelper
pHelper
aav_helper_counsell.fasta
aav_helper_counsell_read_0009.fasta
aav_helper_counsell_read_0006.fasta
aav_helper_counsell_read_0004.fasta
aav_helper_counsell_read_0002.fasta
aav_helper_counsell_read_0001.fasta
aav_helper_counsell_read_0193.fasta
aav_helper_counsell_read_0192.fasta
aav_helper_counsell_read_0191.fasta
aav_helper_counsell_read_0190.fasta
aav_helper_counsell_read_0189.fasta
aav_helper_counsell_read_0188.fasta
aav_helper_counsell_read_0187.fasta
aav_helper_counsell_read_0186.fasta
aav_helper_counsell_read_0185.fasta
aav_helper_counsell_read_0184.fasta
aav_helper_counsell_read_0183.fasta
aav_helper_counsell_read_0182.fasta
aav_helper_counsell_read_0181.fasta
aav_helper_counsell_read_0180.fasta
aav_helper_counsell_read_0179.fasta

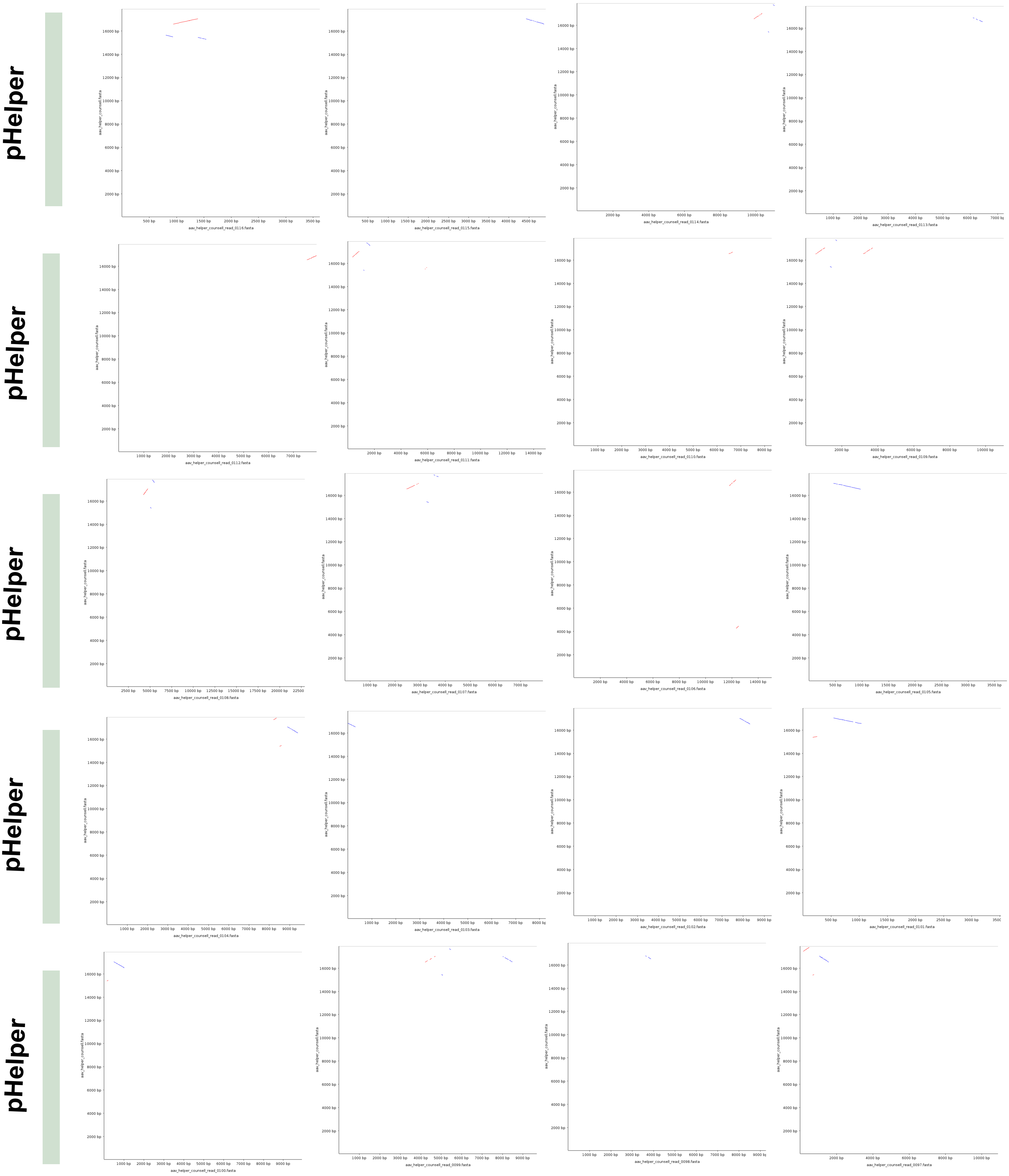
pHelper
pHelper
pHelper
pHelper
pHelper
aav_helper_counsell.fasta
aav_helper_counsell_read_0116.fasta
aav_helper_counsell_read_0115.fasta
aav_helper_counsell_read_0114.fasta
aav_helper_counsell_read_0113.fasta
aav_helper_counsell_read_0112.fasta
aav_helper_counsell_read_0111.fasta
aav_helper_counsell_read_0110.fasta
aav_helper_counsell_read_0109.fasta
aav_helper_counsell_read_0108.fasta
aav_helper_counsell_read_0107.fasta
aav_helper_counsell_read_0106.fasta
aav_helper_counsell_read_0105.fasta
aav_helper_counsell_read_0104.fasta
aav_helper_counsell_read_0103.fasta
aav_helper_counsell_read_0102.fasta
aav_helper_counsell_read_0101.fasta
aav_helper_counsell_read_0100.fasta
aav_helper_counsell_read_0099.fasta
aav_helper_counsell_read_0098.fasta
aav_helper_counsell_read_0097.fasta

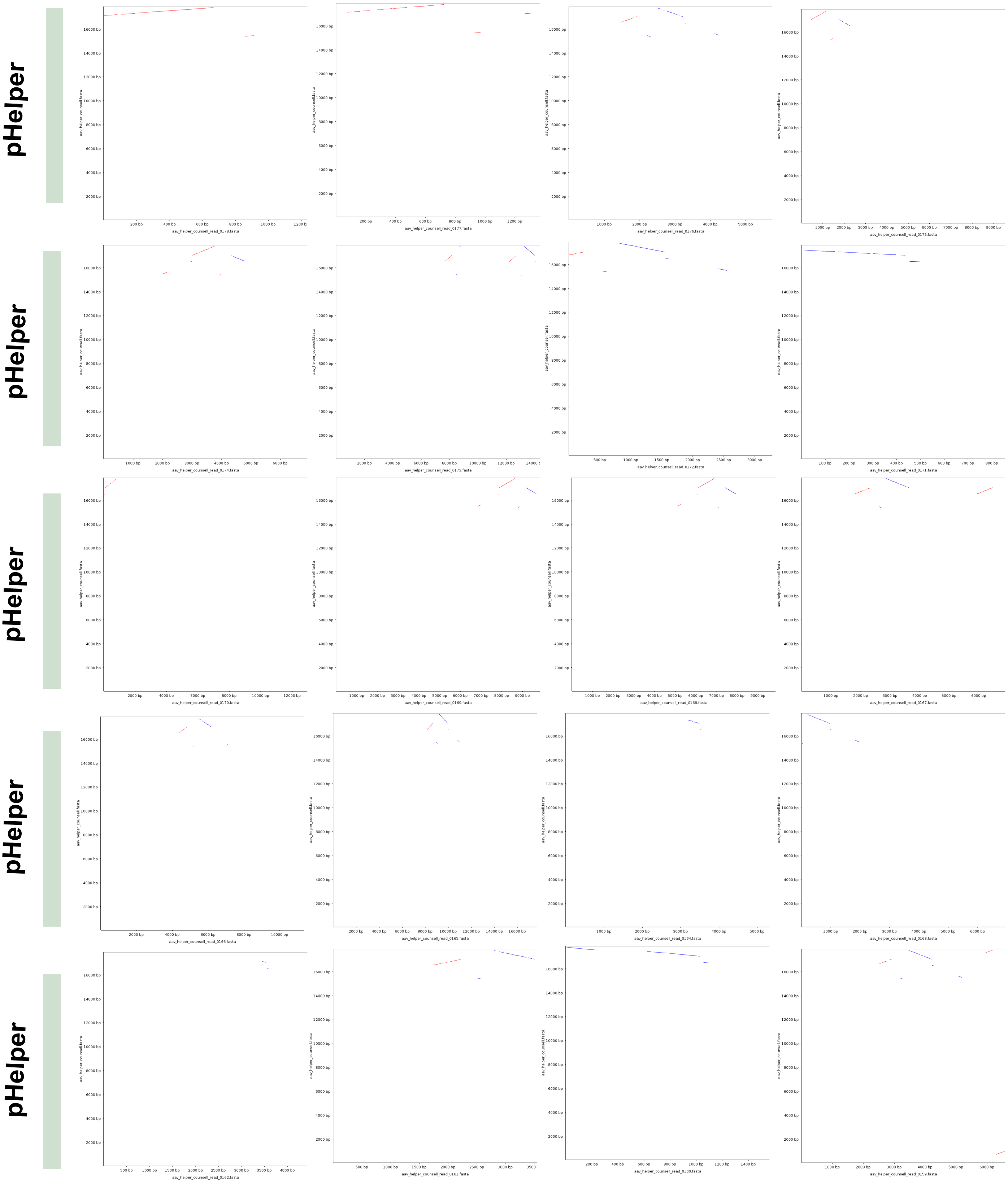
pHelper
aav_helper_counsell.fasta
aav_helper_counsell_read_0178.fasta
aav_helper_counsell_read_0177.fasta
aav_helper_counsell_read_0176.fasta
aav_helper_counsell_read_0175.fasta
pHelper
aav_helper_counsell_read_0174.fasta
aav_helper_counsell_read_0173.fasta
aav_helper_counsell_read_0172.fasta
aav_helper_counsell_read_0171.fasta
pHelper
aav_helper_counsell_read_0170.fasta
aav_helper_counsell_read_0169.fasta
aav_helper_counsell_read_0168.fasta
aav_helper_counsell_read_0167.fasta
pHelper
aav_helper_counsell_read_0166.fasta
aav_helper_counsell_read_0165.fasta
aav_helper_counsell_read_0164.fasta
aav_helper_counsell_read_0163.fasta
pHelper
aav_helper_counsell_read_0162.fasta
aav_helper_counsell_read_0161.fasta
aav_helper_counsell_read_0160.fasta
aav_helper_counsell_read_0159.fasta

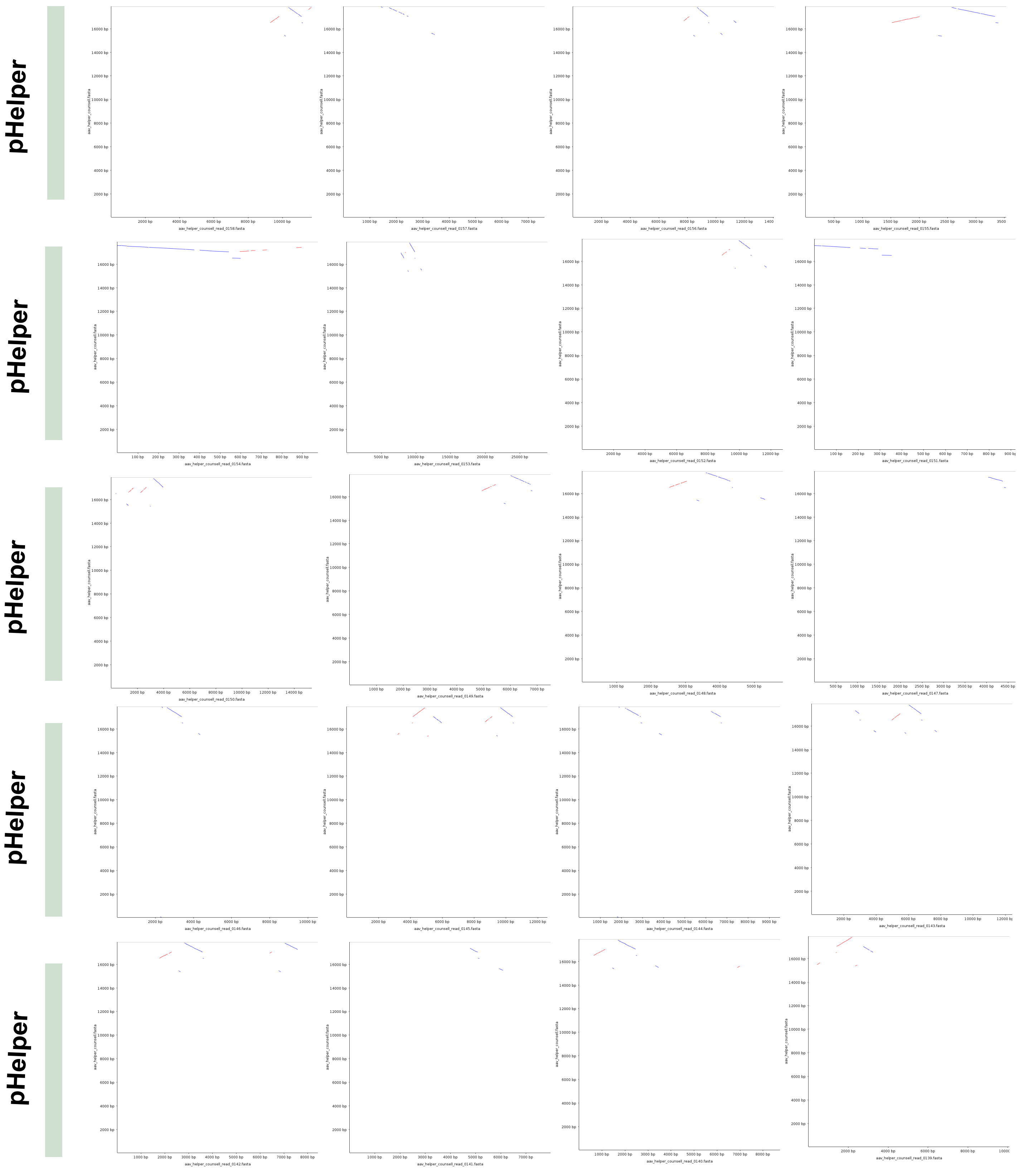
pHelper
pHelper
pHelper
pHelper
pHelper
aav_helper_counsell.fasta
aav_helper_counsell_read_0158.fasta
aav_helper_counsell_read_0157.fasta
aav_helper_counsell_read_0156.fasta
aav_helper_counsell_read_0155.fasta
aav_helper_counsell_read_0154.fasta
aav_helper_counsell_read_0153.fasta
aav_helper_counsell_read_0152.fasta
aav_helper_counsell_read_0151.fasta
aav_helper_counsell_read_0150.fasta
aav_helper_counsell_read_0149.fasta
aav_helper_counsell_read_0148.fasta
aav_helper_counsell_read_0147.fasta
aav_helper_counsell_read_0146.fasta
aav_helper_counsell_read_0145.fasta
aav_helper_counsell_read_0144.fasta
aav_helper_counsell_read_0143.fasta
aav_helper_counsell_read_0142.fasta
aav_helper_counsell_read_0141.fasta
aav_helper_counsell_read_0140.fasta
aav_helper_counsell_read_0139.fasta

pHelper

pHelper

pHelper

pHelper

pHelper

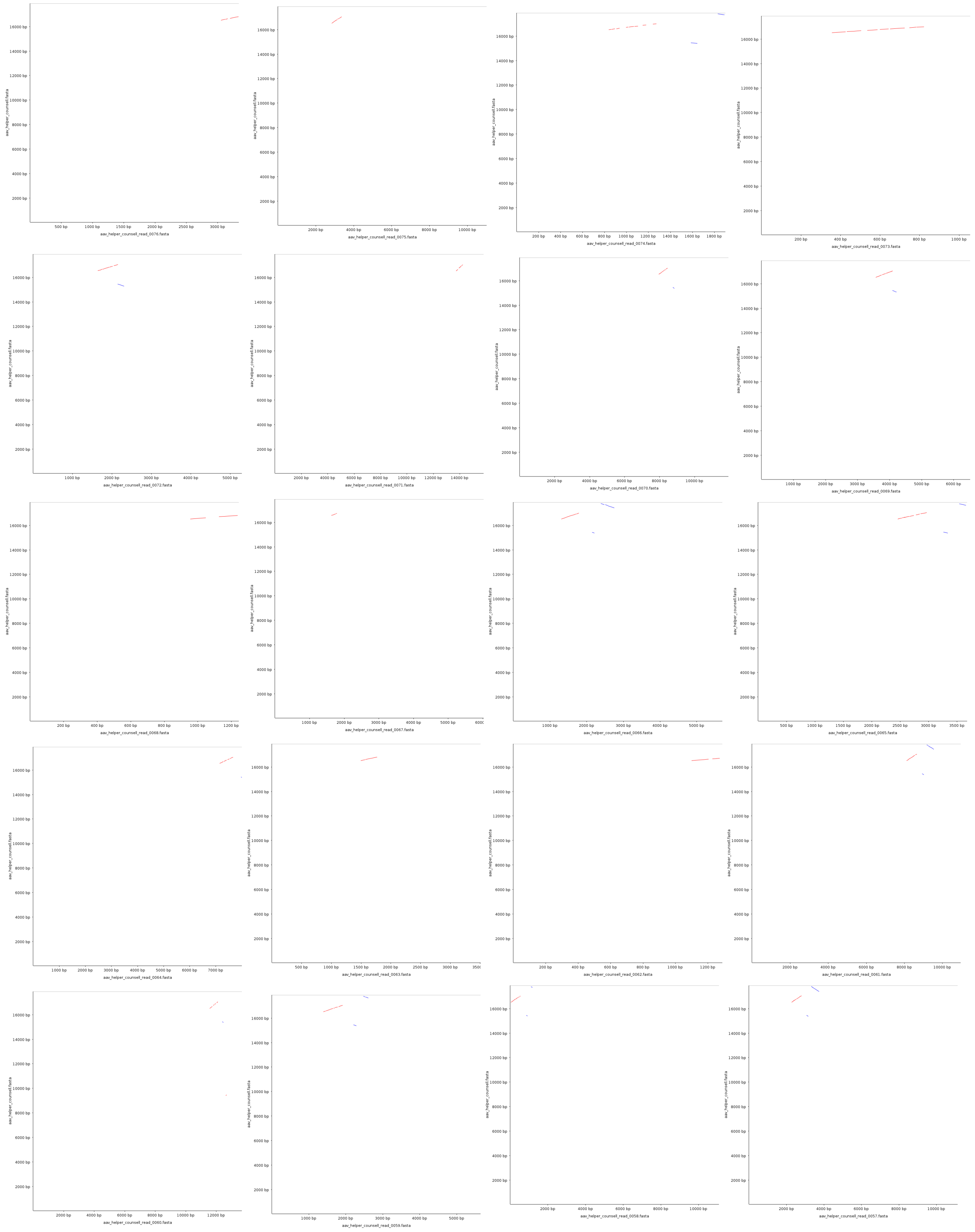

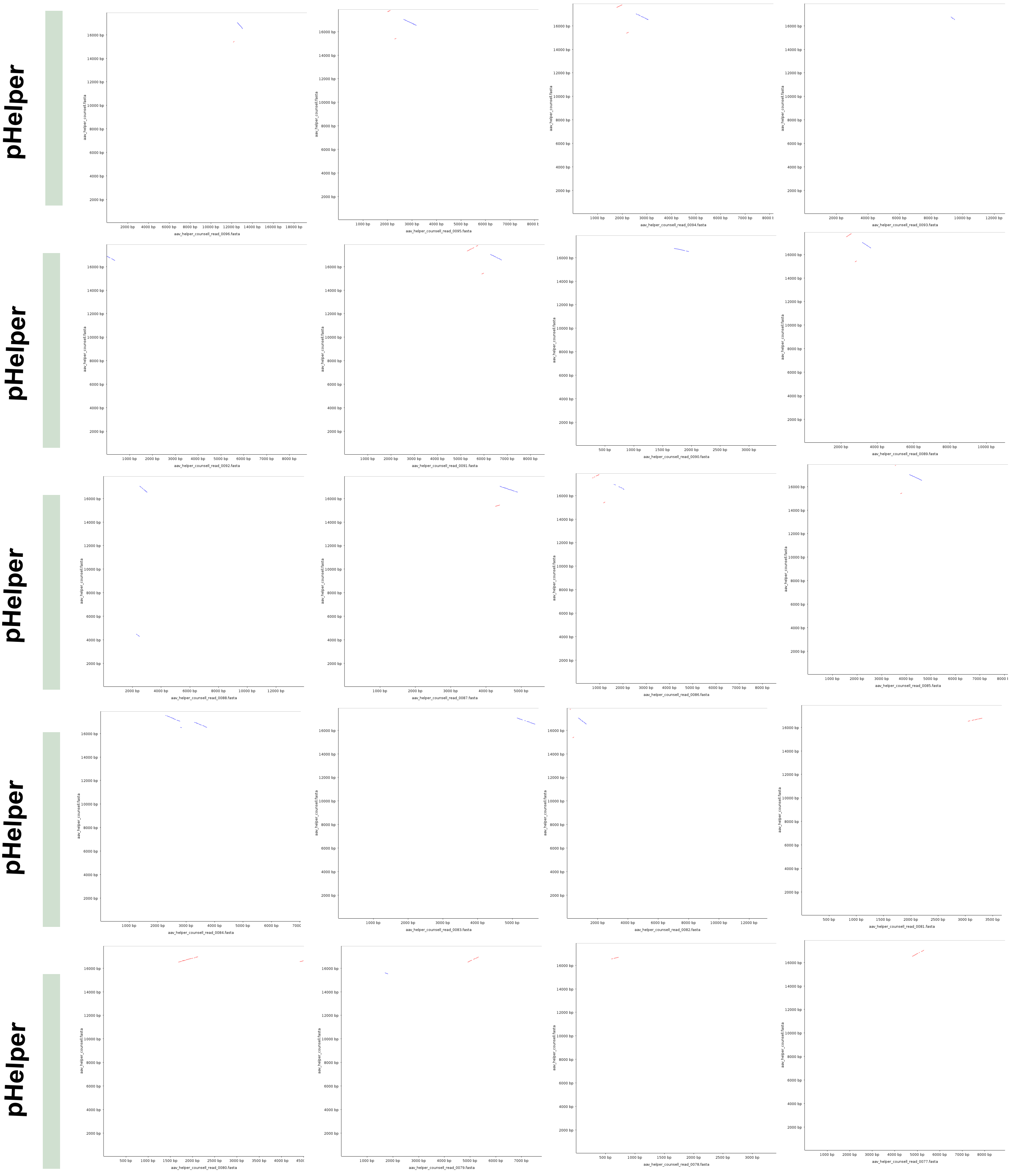
pHelper
pHelper
pHelper
pHelper
pHelper
aav_helper_counsell.fasta
aav_helper_counsell_read_0096.fasta
aav_helper_counsell_read_0095.fasta
aav_helper_counsell_read_0094.fasta
aav_helper_counsell_read_0093.fasta
aav_helper_counsell_read_0092.fasta
aav_helper_counsell_read_0091.fasta
aav_helper_counsell_read_0090.fasta
aav_helper_counsell_read_0089.fasta
aav_helper_counsell_read_0088.fasta
aav_helper_counsell_read_0087.fasta
aav_helper_counsell_read_0086.fasta
aav_helper_counsell_read_0085.fasta
aav_helper_counsell_read_0084.fasta
aav_helper_counsell_read_0083.fasta
aav_helper_counsell_read_0082.fasta
aav_helper_counsell_read_0081.fasta
aav_helper_counsell_read_0080.fasta
aav_helper_counsell_read_0079.fasta
aav_helper_counsell_read_0078.fasta
aav_helper_counsell_read_0077.fasta

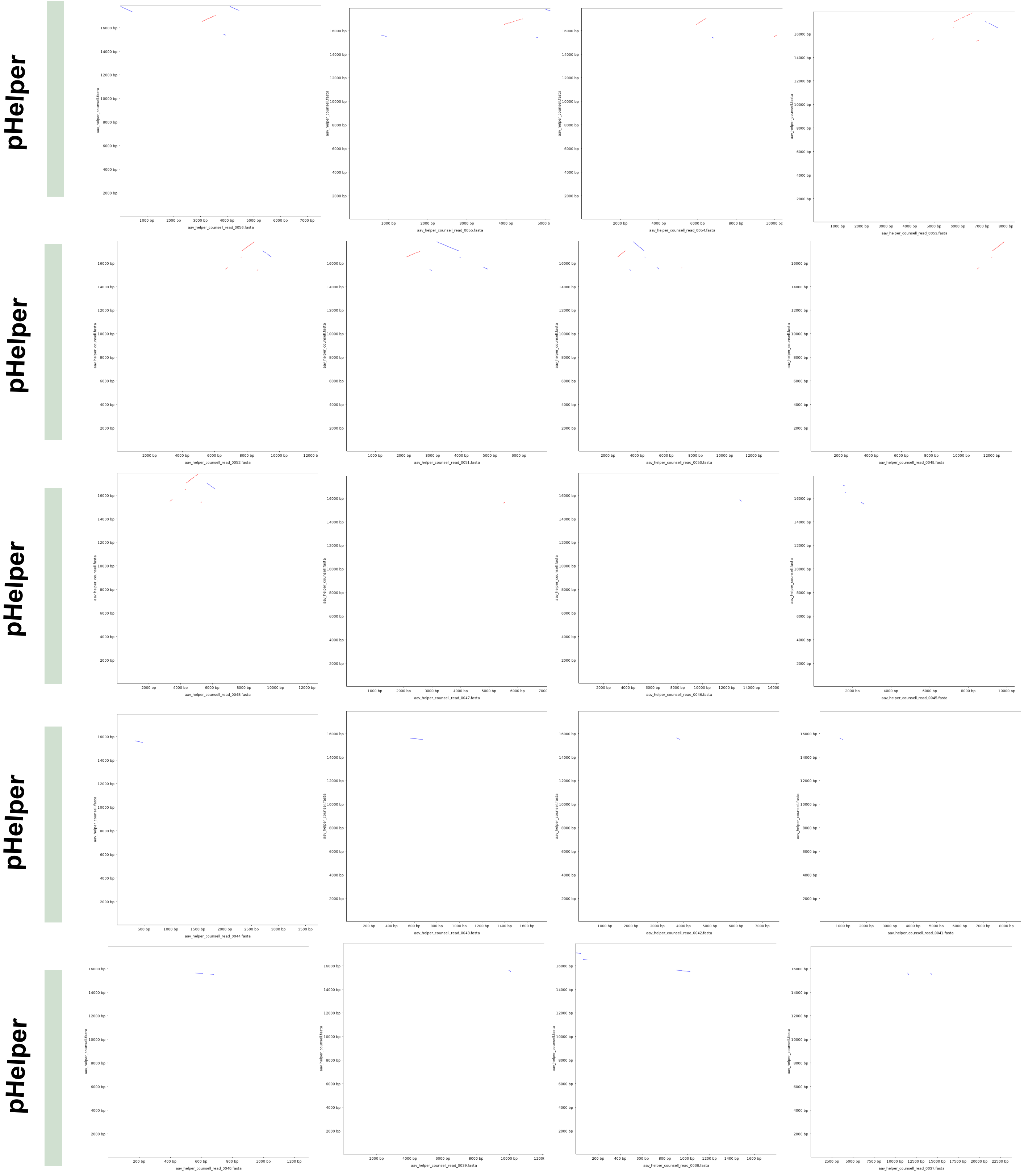
pHelper
pHelper
pHelper
pHelper
pHelper
aav_helper_counsell.fasta
aav_helper_counsell_read_0056.fasta
aav_helper_counsell_read_0055.fasta
aav_helper_counsell_read_0054.fasta
aav_helper_counsell_read_0053.fasta
aav_helper_counsell_read_0052.fasta
aav_helper_counsell_read_0051.fasta
aav_helper_counsell_read_0050.fasta
aav_helper_counsell_read_0049.fasta
aav_helper_counsell_read_0048.fasta
aav_helper_counsell_read_0047.fasta
aav_helper_counsell_read_0046.fasta
aav_helper_counsell_read_0045.fasta
aav_helper_counsell_read_0044.fasta
aav_helper_counsell_read_0043.fasta
aav_helper_counsell_read_0042.fasta
aav_helper_counsell_read_0041.fasta
aav_helper_counsell_read_0040.fasta
aav_helper_counsell_read_0039.fasta
aav_helper_counsell_read_0038.fasta
aav_helper_counsell_read_0037.fasta

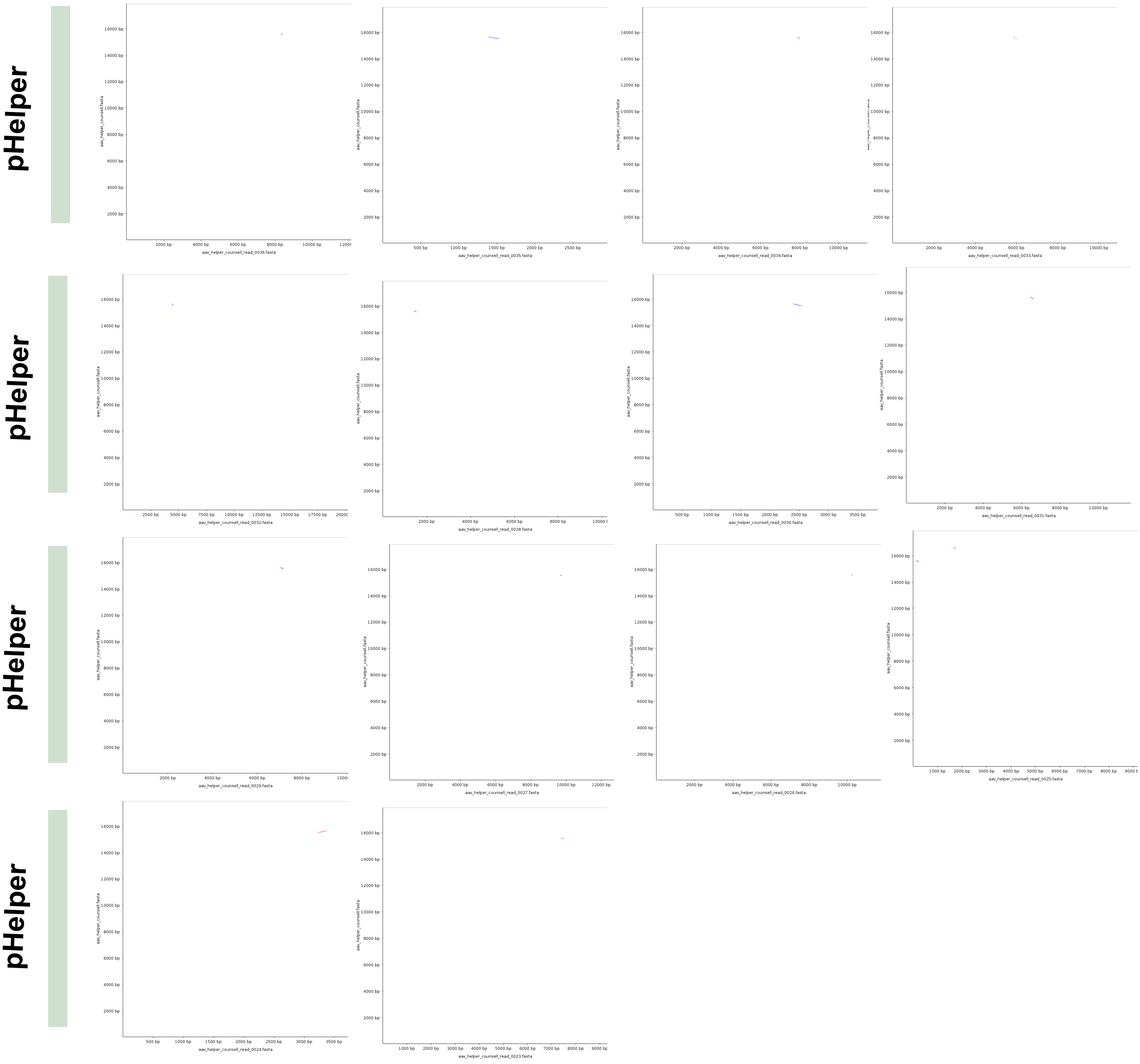
pHelper
aav_helper_counsell.fasta
aav_helper_counsell_read_0036.fasta
aav_helper_counsell_read_0035.fasta
aav_helper_counsell_read_0034.fasta
aav_helper_counsell_read_0033.fasta
pHelper
aav_helper_counsell_read_0032.fasta
aav_helper_counsell_read_0028.fasta
aav_helper_counsell_read_0030.fasta
aav_helper_counsell_read_0031.fasta
pHelper
aav_helper_counsell_read_0029.fasta
aav_helper_counsell_read_0027.fasta
aav_helper_counsell_read_0026.fasta
aav_helper_counsell_read_0025.fasta
pHelper
aav_helper_counsell_read_0024.fasta
aav_helper_counsell_read_0023.fasta

Regions of interest of all plasmids (y axis)
pAAV2/9 reads aligning to another plasmid

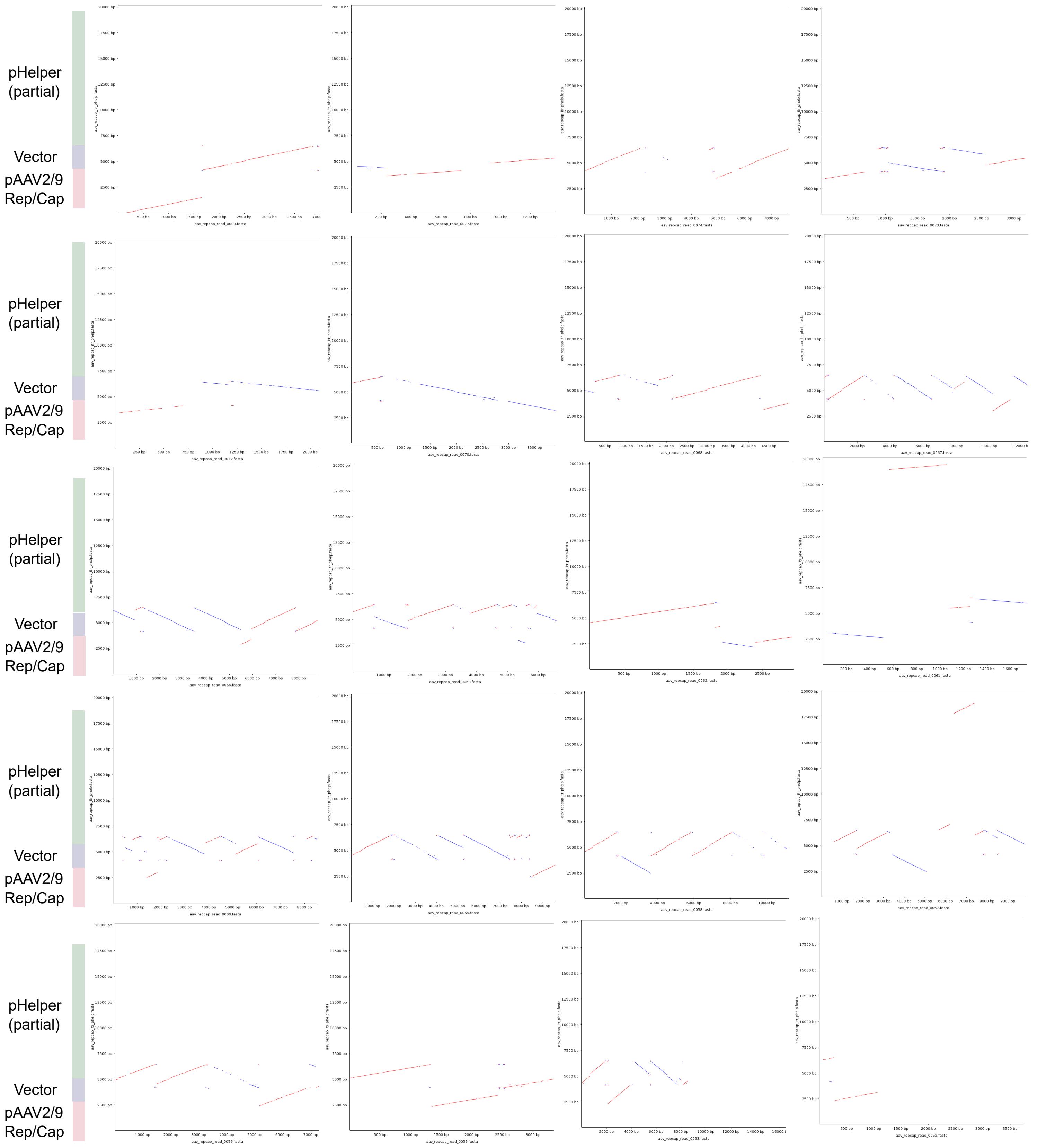

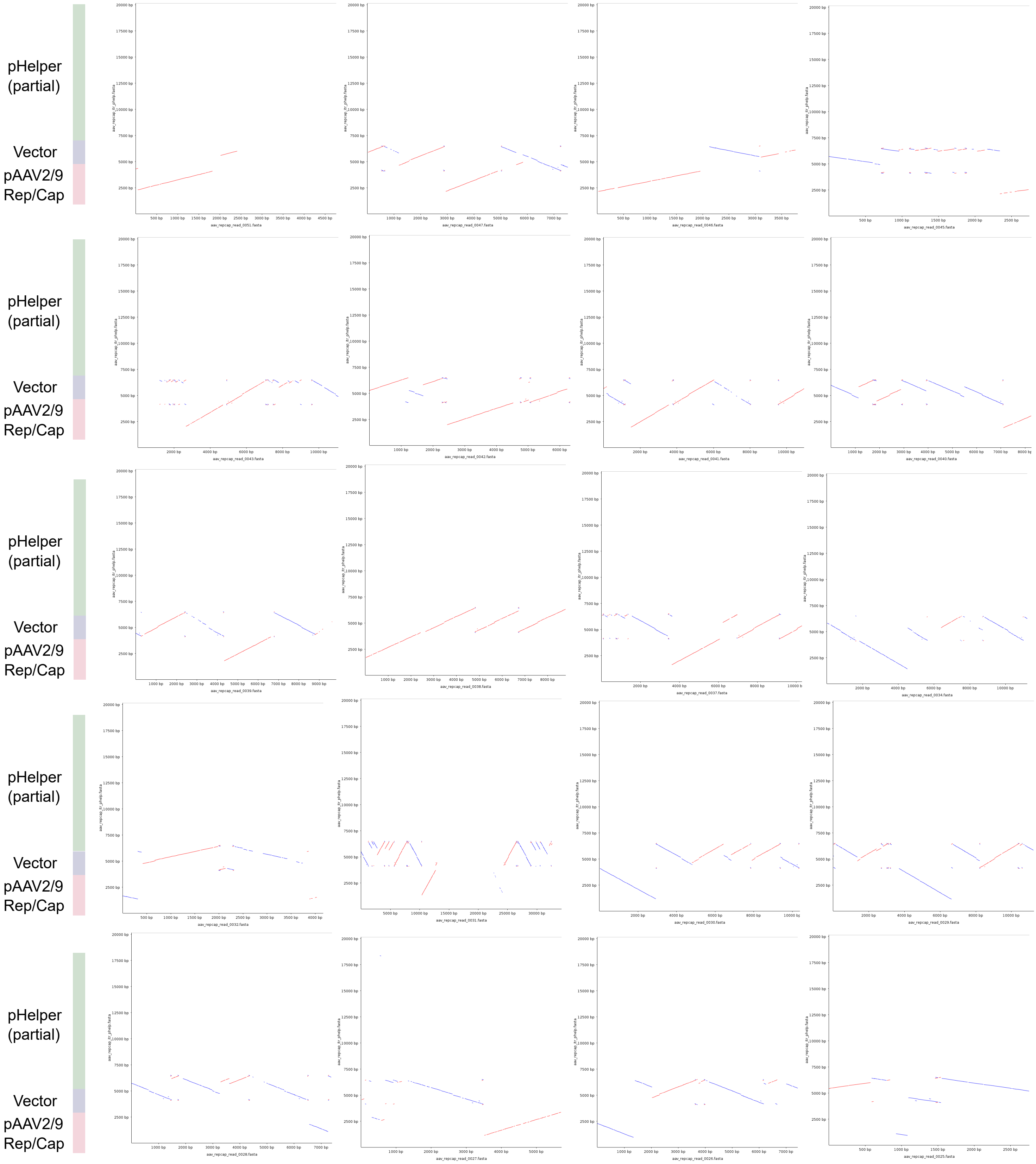
pHelper (partial)
Vector
pAAV2/9 Rep/Cap
aav_repcap_read_0051.fasta
aav_repcap_read_0047.fasta
aav_repcap_read_0046.fasta
aav_repcap_read_0045.fasta
pHelper (partial)
Vector
pAAV2/9 Rep/Cap
aav_repcap_read_0043.fasta
aav_repcap_read_0042.fasta
aav_repcap_read_0041.fasta
aav_repcap_read_0040.fasta
pHelper (partial)
Vector
pAAV2/9 Rep/Cap
aav_repcap_read_0039.fasta
aav_repcap_read_0038.fasta
aav_repcap_read_0037.fasta
aav_repcap_read_0034.fasta
pHelper (partial)
Vector
pAAV2/9 Rep/Cap
aav_repcap_read_0032.fasta
aav_repcap_read_0031.fasta
aav_repcap_read_0030.fasta
aav_repcap_read_0029.fasta
pHelper (partial)
Vector
pAAV2/9 Rep/Cap
aav_repcap_read_0028.fasta
aav_repcap_read_0027.fasta
aav_repcap_read_0026.fasta
aav_repcap_read_0025.fasta

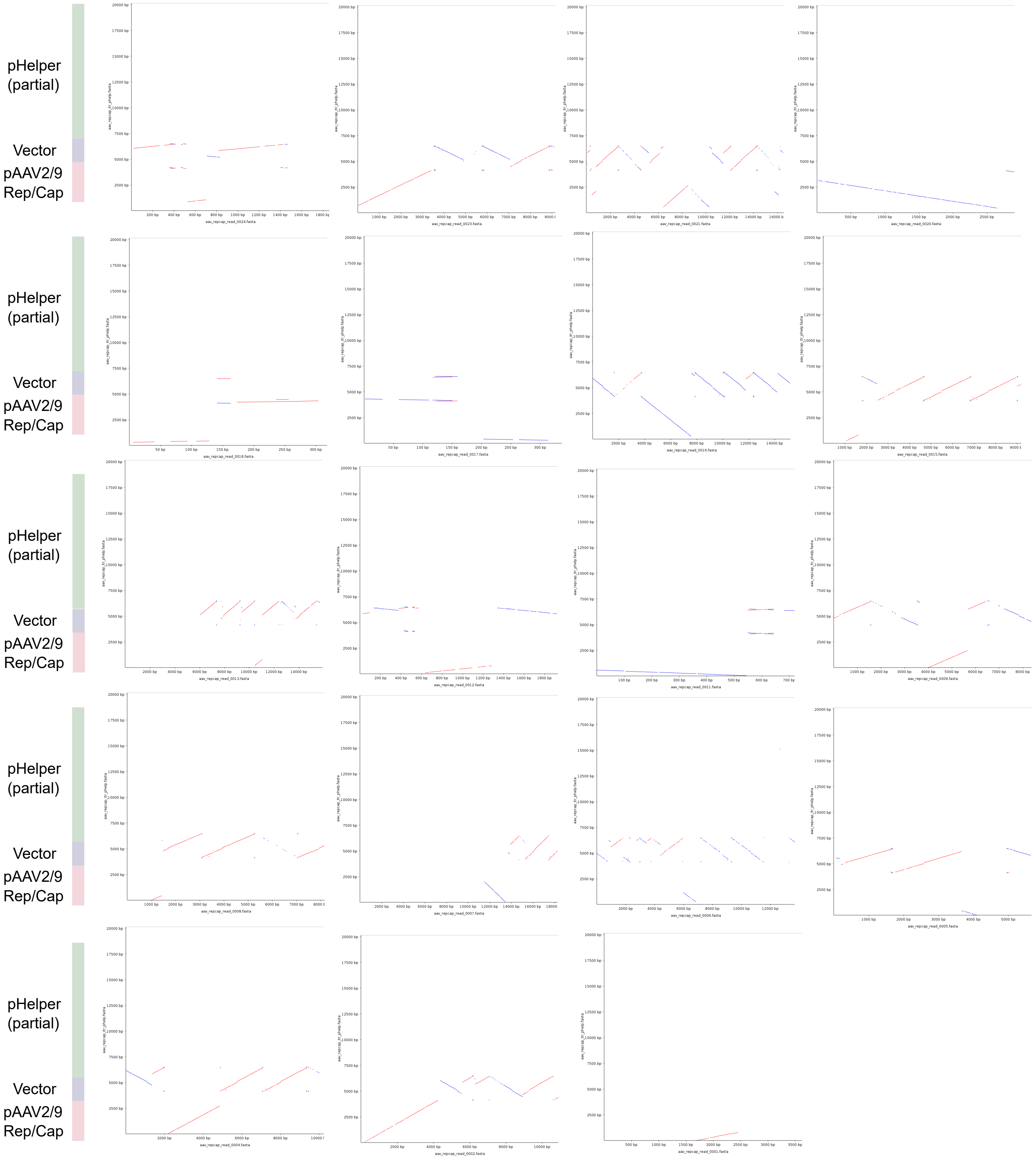
pHelper
(partial)
Vector
pAAV2/9
Rep/Cap
pHelper
(partial)
Vector
pAAV2/9
Rep/Cap
pHelper
(partial)
Vector
pAAV2/9
Rep/Cap
pHelper
(partial)
Vector
pAAV2/9
Rep/Cap
pHelper
(partial)
Vector
pAAV2/9
Rep/Cap

Regions of interest of all plasmids (y axis)
pHelper reads aligning to another plasmid

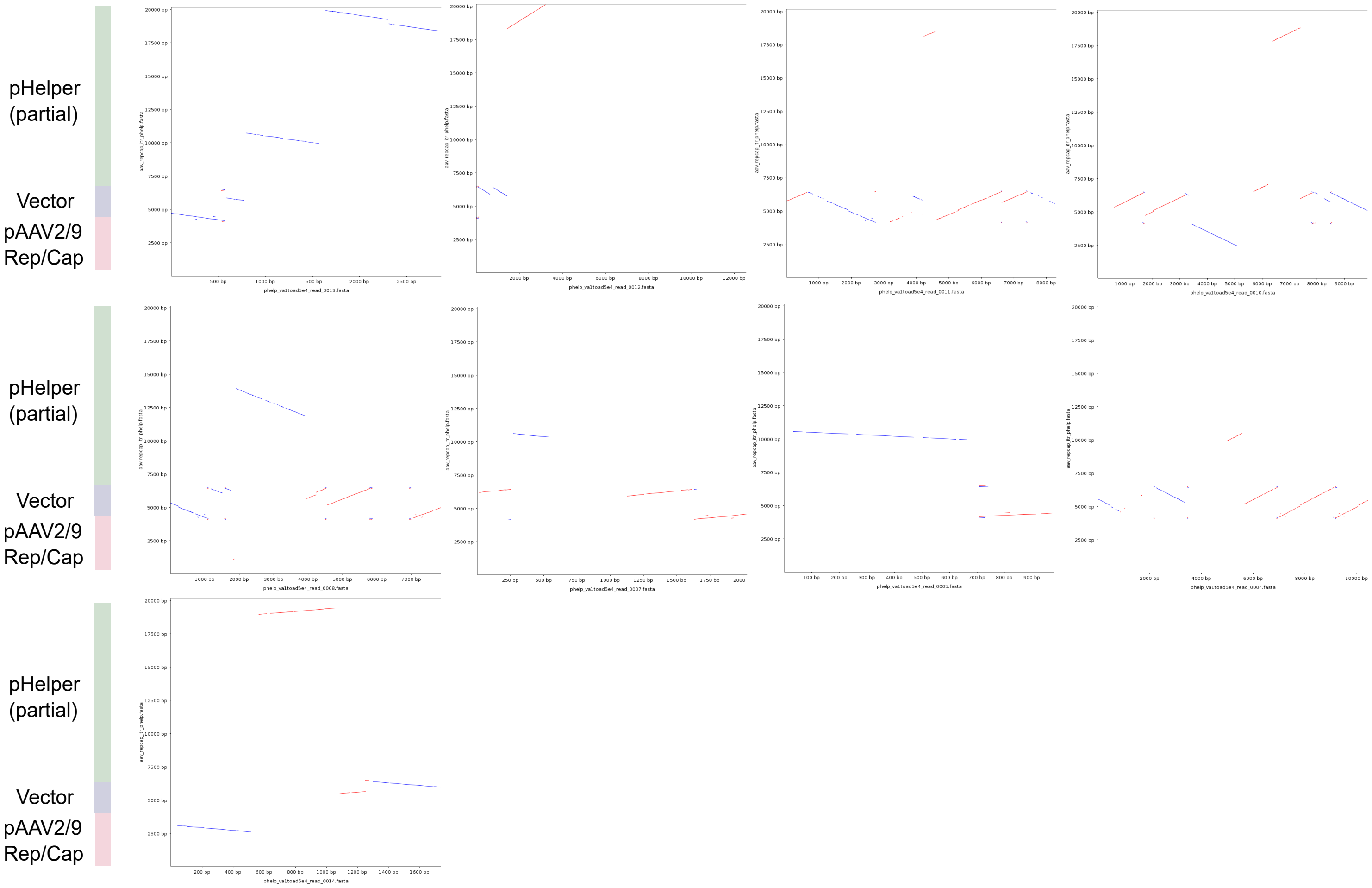

Figure S2

Y axis: ACTB gene and surrounding region in human genome
X axis: Nanopore read

Normal reads

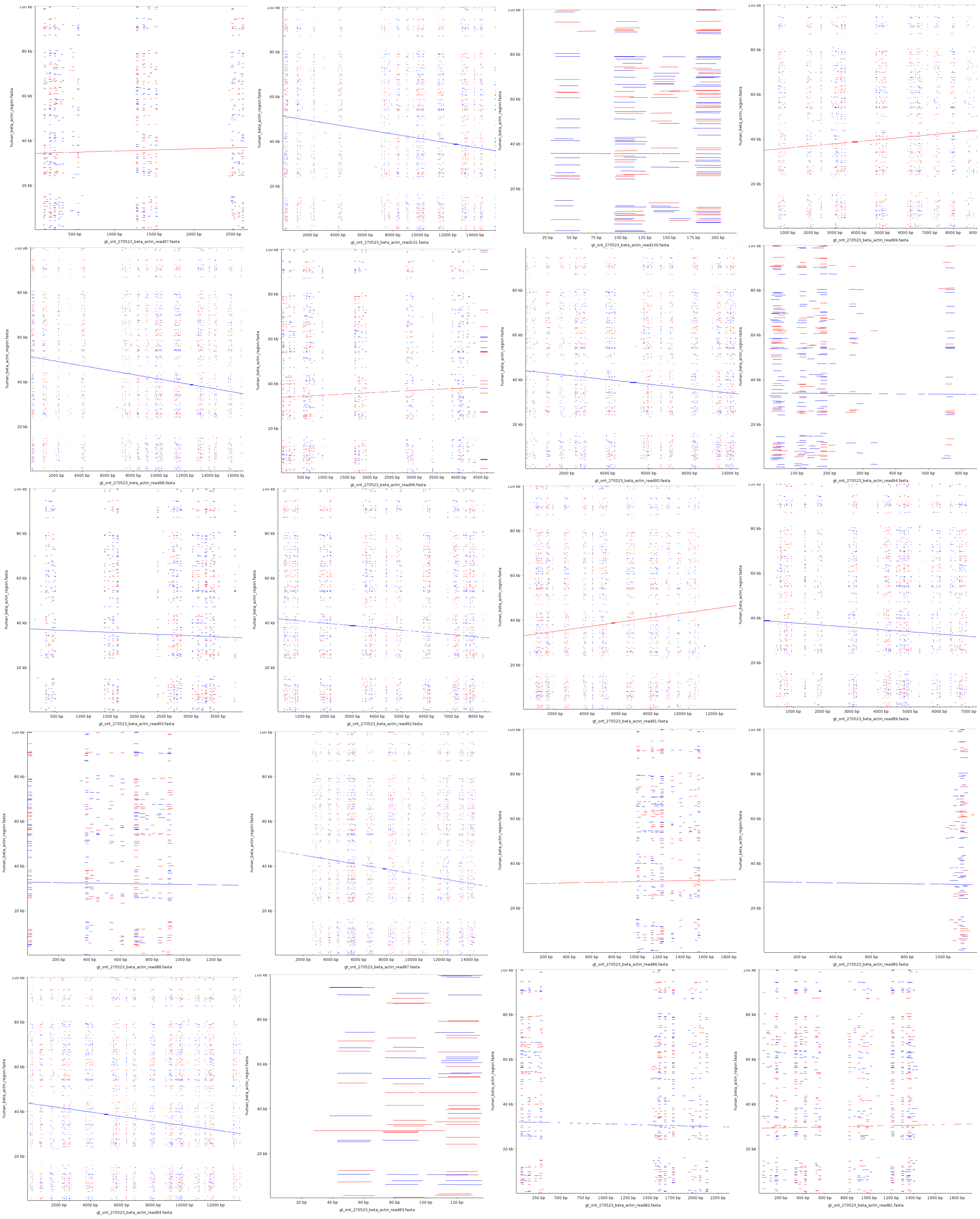

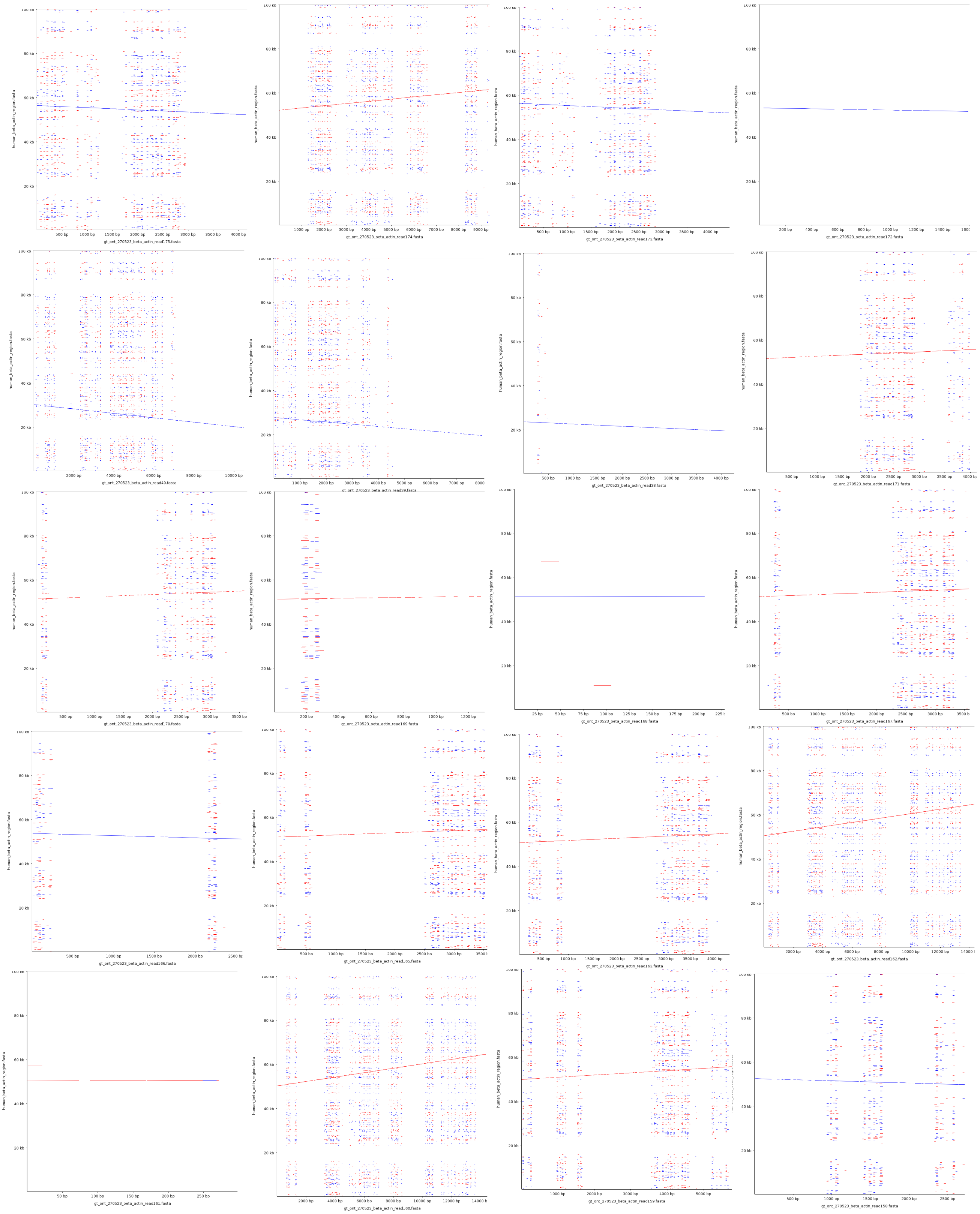
human_beta_actin_region.fasta
gt_ont_270523_beta_actin_read175.fasta
gt_ont_270523_beta_actin_read174.fasta
gt_ont_270523_beta_actin_read173.fasta
gt_ont_270523_beta_actin_read172.fasta
gt_ont_270523_beta_actin_read40.fasta
gt_ont_270523_beta_actin_read39.fasta
gt_ont_270523_beta_actin_read38.fasta
gt_ont_270523_beta_actin_read171.fasta
gt_ont_270523_beta_actin_read170.fasta
gt_ont_270523_beta_actin_read169.fasta
gt_ont_270523_beta_actin_read168.fasta
gt_ont_270523_beta_actin_read167.fasta
gt_ont_270523_beta_actin_read166.fasta
gt_ont_270523_beta_actin_read165.fasta
gt_ont_270523_beta_actin_read163.fasta
gt_ont_270523_beta_actin_read162.fasta
gt_ont_270523_beta_actin_read161.fasta
gt_ont_270523_beta_actin_read160.fasta
gt_ont_270523_beta_actin_read159.fasta
gt_ont_270523_beta_actin_read158.fasta

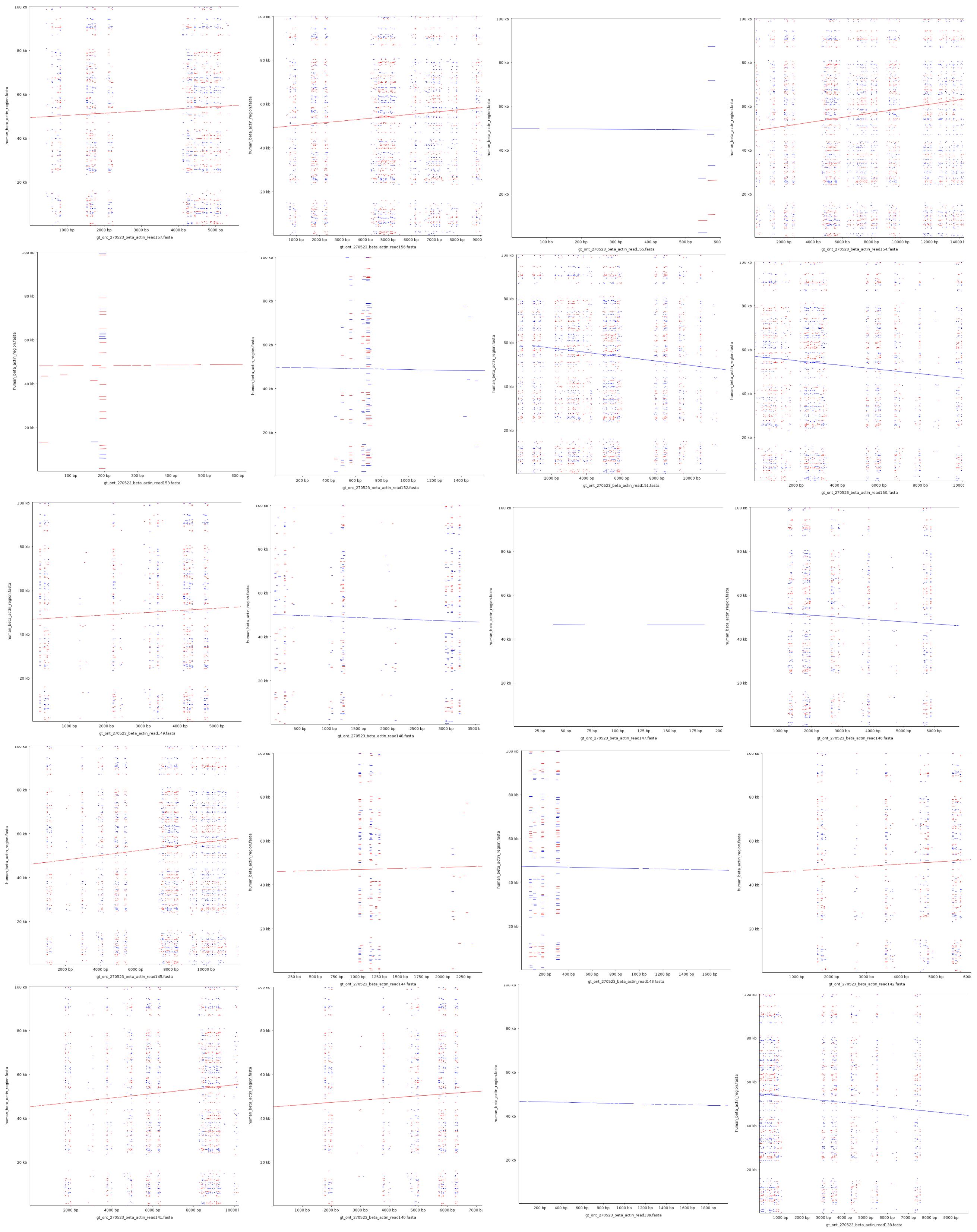
human_beta_actin_region.fasta
gt_ont_270523_beta_actin_read157.fasta
gt_ont_270523_beta_actin_read156.fasta
gt_ont_270523_beta_actin_read155.fasta
gt_ont_270523_beta_actin_read154.fasta
gt_ont_270523_beta_actin_read153.fasta
gt_ont_270523_beta_actin_read152.fasta
gt_ont_270523_beta_actin_read151.fasta
gt_ont_270523_beta_actin_read150.fasta
gt_ont_270523_beta_actin_read149.fasta
gt_ont_270523_beta_actin_read148.fasta
gt_ont_270523_beta_actin_read147.fasta
gt_ont_270523_beta_actin_read146.fasta
gt_ont_270523_beta_actin_read145.fasta
gt_ont_270523_beta_actin_read144.fasta
gt_ont_270523_beta_actin_read143.fasta
gt_ont_270523_beta_actin_read142.fasta
gt_ont_270523_beta_actin_read141.fasta
gt_ont_270523_beta_actin_read140.fasta
gt_ont_270523_beta_actin_read139.fasta
gt_ont_270523_beta_actin_read138.fasta

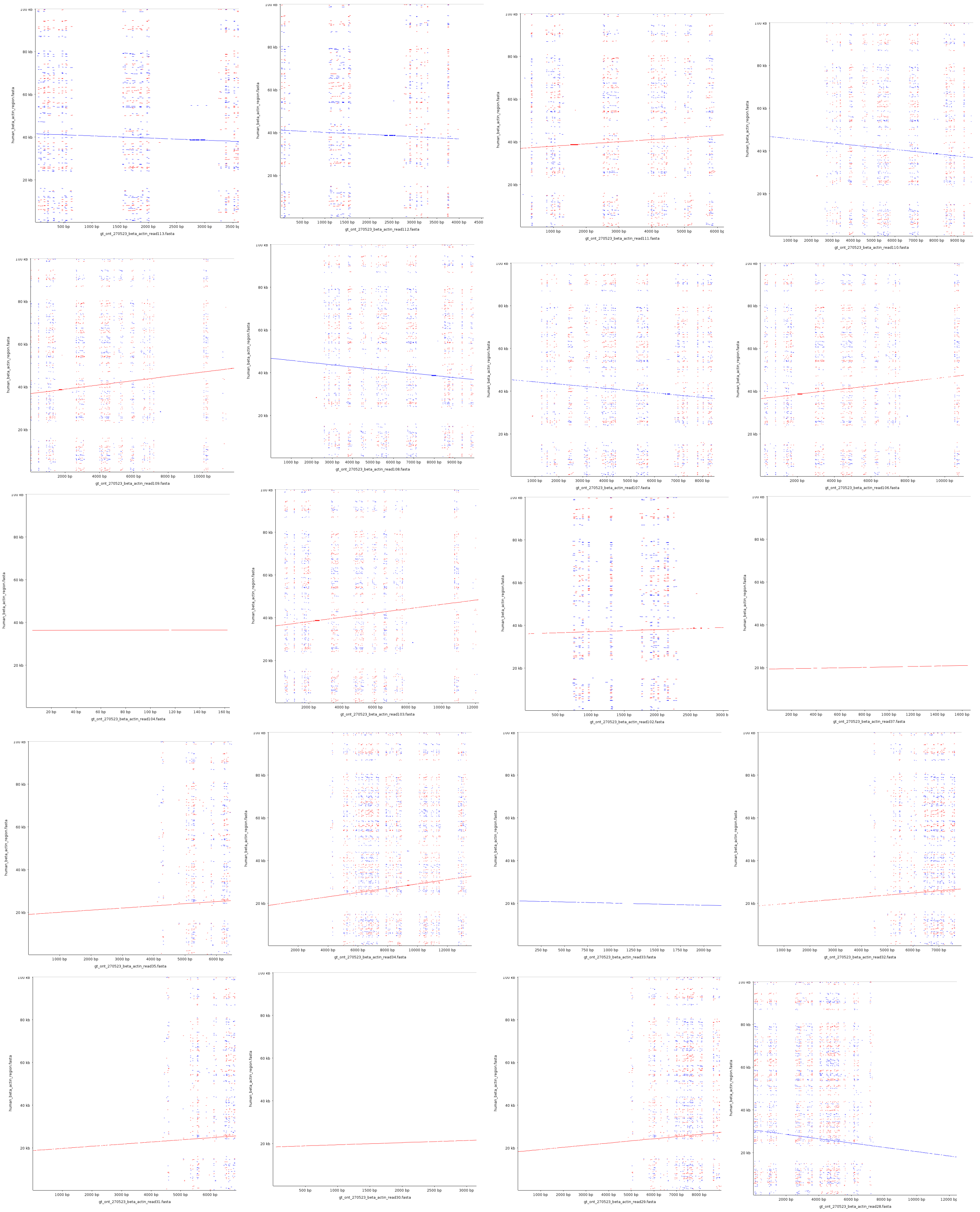
human_beta_actin_region.fasta
gt_ont_270523_beta_actin_read113.fasta
gt_ont_270523_beta_actin_read112.fasta
gt_ont_270523_beta_actin_read111.fasta
gt_ont_270523_beta_actin_read110.fasta
gt_ont_270523_beta_actin_read109.fasta
gt_ont_270523_beta_actin_read108.fasta
gt_ont_270523_beta_actin_read107.fasta
gt_ont_270523_beta_actin_read106.fasta
gt_ont_270523_beta_actin_read104.fasta
gt_ont_270523_beta_actin_read103.fasta
gt_ont_270523_beta_actin_read102.fasta
gt_ont_270523_beta_actin_read37.fasta
gt_ont_270523_beta_actin_read35.fasta
gt_ont_270523_beta_actin_read34.fasta
gt_ont_270523_beta_actin_read33.fasta
gt_ont_270523_beta_actin_read32.fasta
gt_ont_270523_beta_actin_read31.fasta
gt_ont_270523_beta_actin_read30.fasta
gt_ont_270523_beta_actin_read29.fasta
gt_ont_270523_beta_actin_read28.fasta

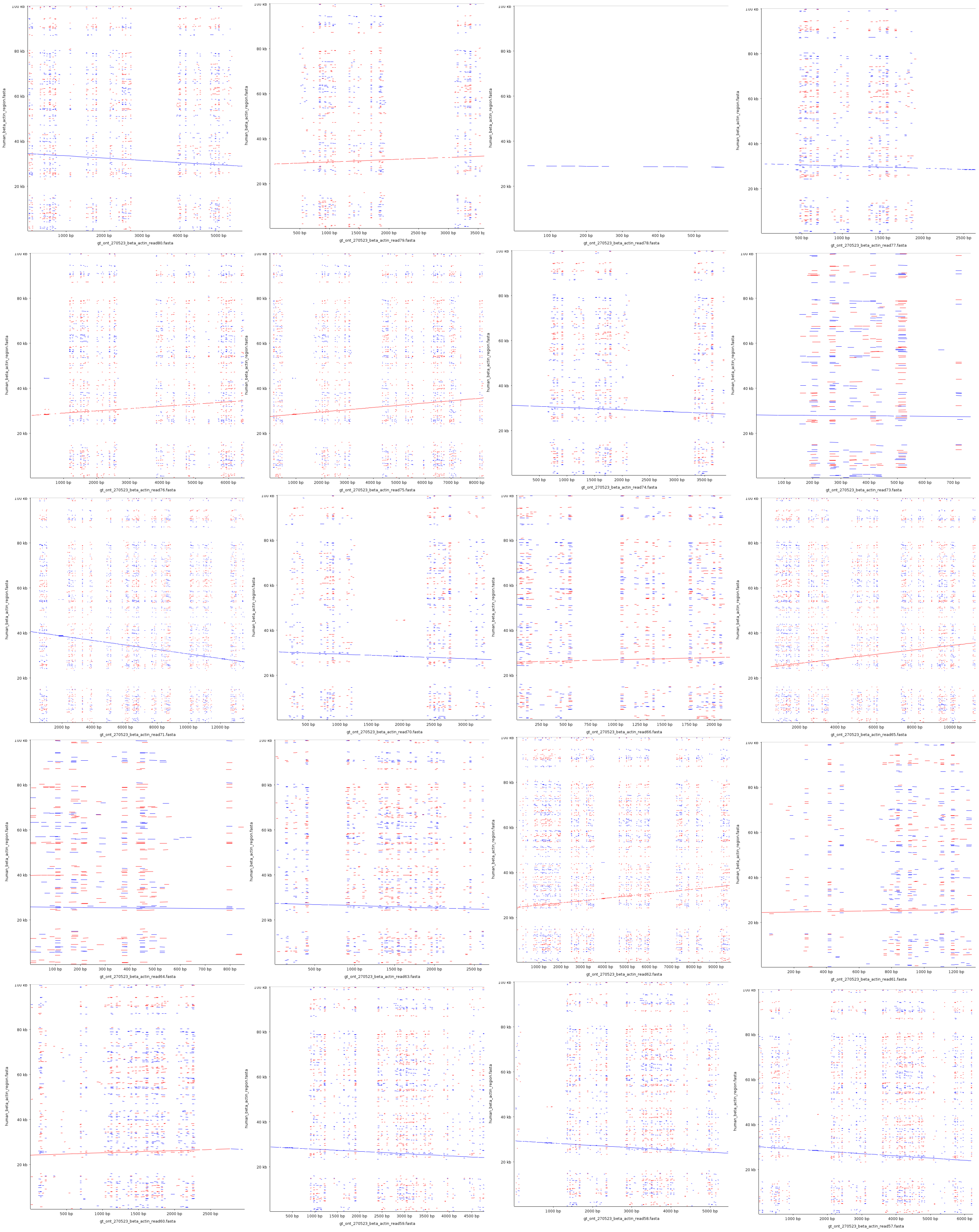
human_beta_actin_region.fasta
gt_ont_270523_beta_actin_read80.fasta
gt_ont_270523_beta_actin_read79.fasta
gt_ont_270523_beta_actin_read78.fasta
gt_ont_270523_beta_actin_read77.fasta
gt_ont_270523_beta_actin_read76.fasta
gt_ont_270523_beta_actin_read75.fasta
gt_ont_270523_beta_actin_read74.fasta
gt_ont_270523_beta_actin_read73.fasta
gt_ont_270523_beta_actin_read71.fasta
gt_ont_270523_beta_actin_read70.fasta
gt_ont_270523_beta_actin_read66.fasta
gt_ont_270523_beta_actin_read65.fasta
gt_ont_270523_beta_actin_read64.fasta
gt_ont_270523_beta_actin_read63.fasta
gt_ont_270523_beta_actin_read62.fasta
gt_ont_270523_beta_actin_read61.fasta
gt_ont_270523_beta_actin_read60.fasta
gt_ont_270523_beta_actin_read59.fasta
gt_ont_270523_beta_actin_read58.fasta
gt_ont_270523_beta_actin_read57.fasta

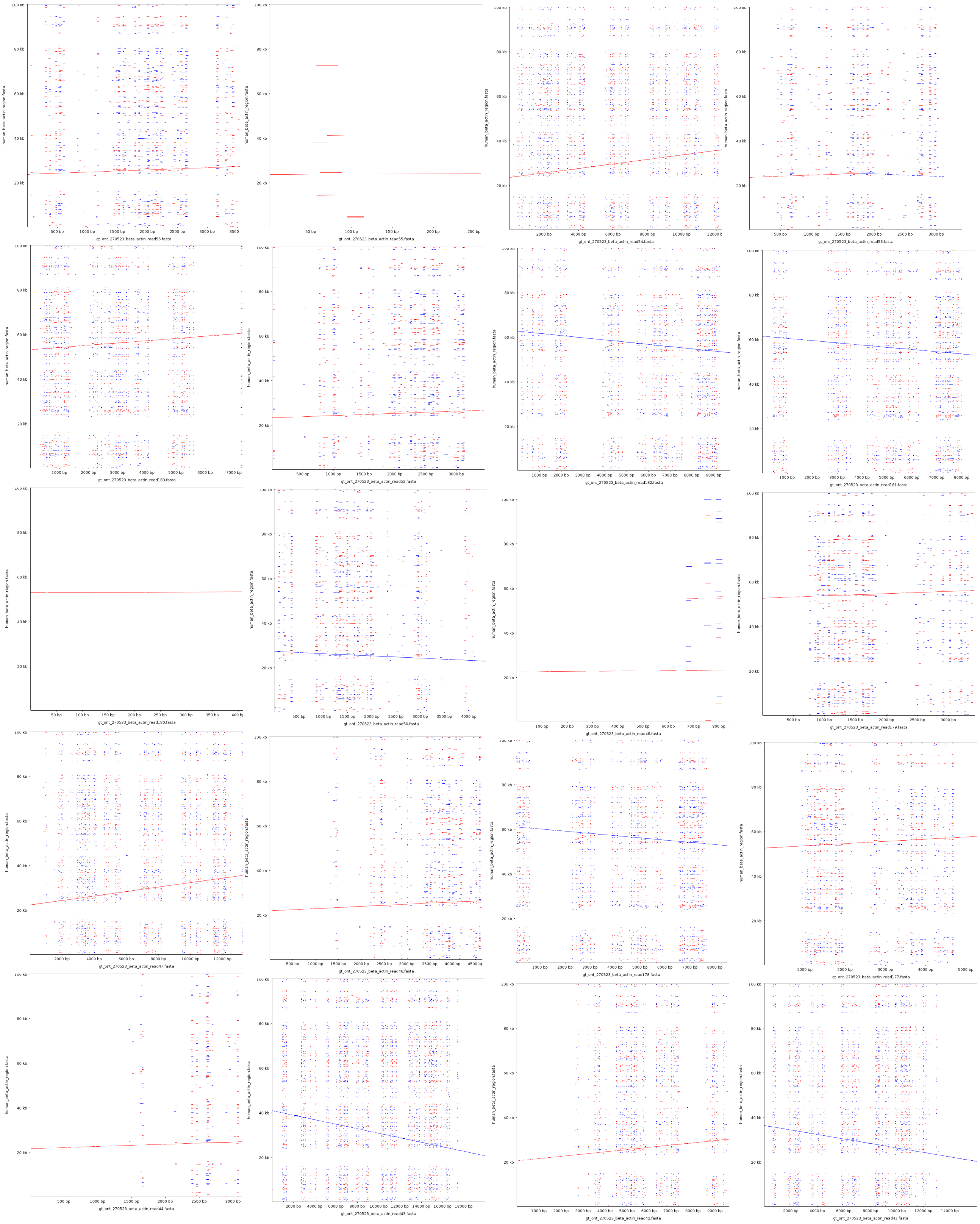
human_beta_actin_region.fasta
gt_ont_270523_beta_actin_read56.fasta
gt_ont_270523_beta_actin_read55.fasta
gt_ont_270523_beta_actin_read54.fasta
gt_ont_270523_beta_actin_read53.fasta
gt_ont_270523_beta_actin_read183.fasta
gt_ont_270523_beta_actin_read52.fasta
gt_ont_270523_beta_actin_read182.fasta
gt_ont_270523_beta_actin_read181.fasta
gt_ont_270523_beta_actin_read180.fasta
gt_ont_270523_beta_actin_read50.fasta
gt_ont_270523_beta_actin_read48.fasta
gt_ont_270523_beta_actin_read179.fasta
gt_ont_270523_beta_actin_read47.fasta
gt_ont_270523_beta_actin_read46.fasta
gt_ont_270523_beta_actin_read178.fasta
gt_ont_270523_beta_actin_read177.fasta
gt_ont_270523_beta_actin_read44.fasta
gt_ont_270523_beta_actin_read43.fasta
gt_ont_270523_beta_actin_read42.fasta
gt_ont_270523_beta_actin_read41.fasta

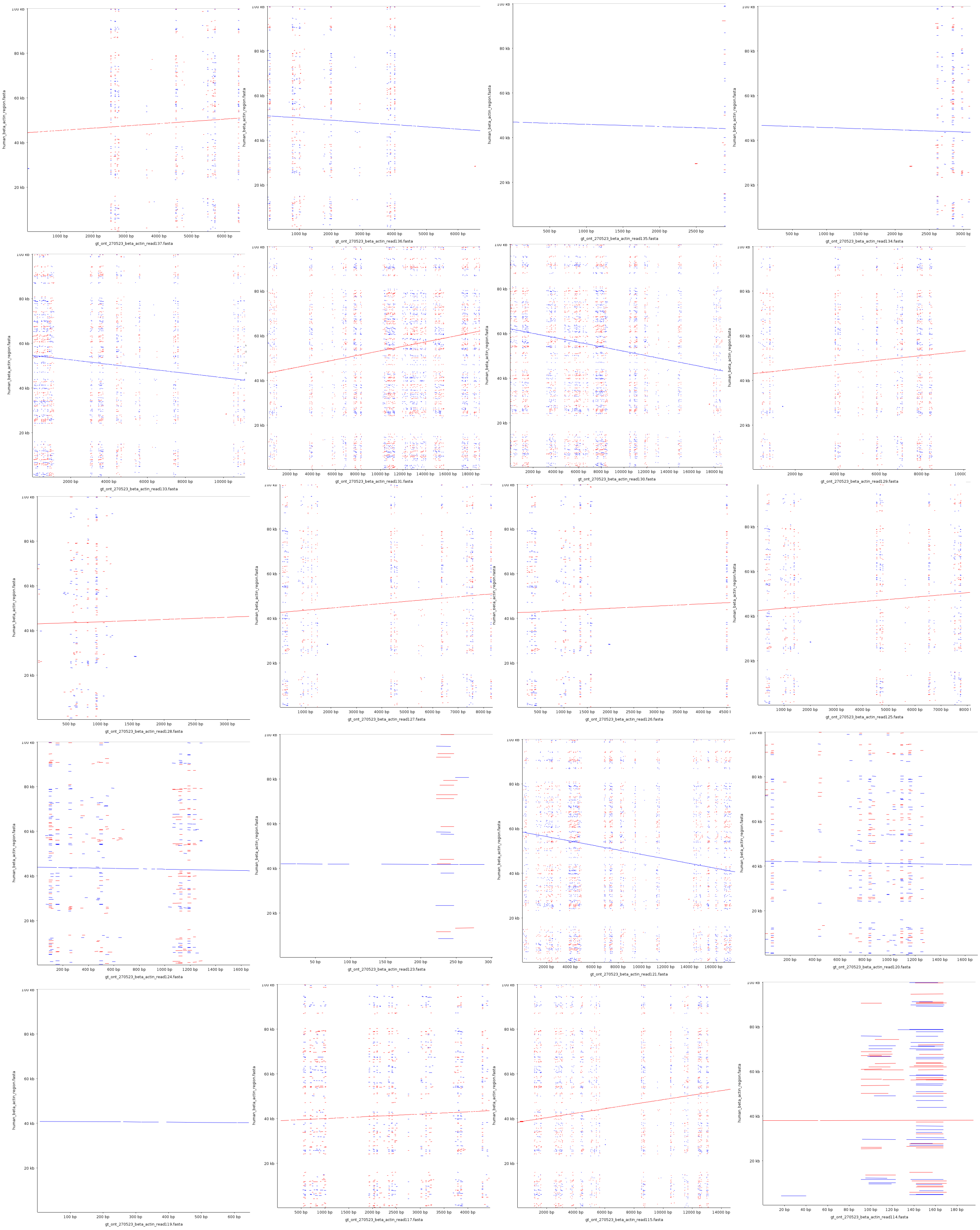
human_beta_actin_region.fasta
gt_ont_270523_beta_actin_read137.fasta
gt_ont_270523_beta_actin_read136.fasta
gt_ont_270523_beta_actin_read135.fasta
gt_ont_270523_beta_actin_read134.fasta
gt_ont_270523_beta_actin_read133.fasta
gt_ont_270523_beta_actin_read131.fasta
gt_ont_270523_beta_actin_read130.fasta
gt_ont_270523_beta_actin_read129.fasta
gt_ont_270523_beta_actin_read128.fasta
gt_ont_270523_beta_actin_read127.fasta
gt_ont_270523_beta_actin_read126.fasta
gt_ont_270523_beta_actin_read125.fasta
gt_ont_270523_beta_actin_read124.fasta
gt_ont_270523_beta_actin_read123.fasta
gt_ont_270523_beta_actin_read121.fasta
gt_ont_270523_beta_actin_read120.fasta
gt_ont_270523_beta_actin_read119.fasta
gt_ont_270523_beta_actin_read117.fasta
gt_ont_270523_beta_actin_read115.fasta
gt_ont_270523_beta_actin_read114.fasta

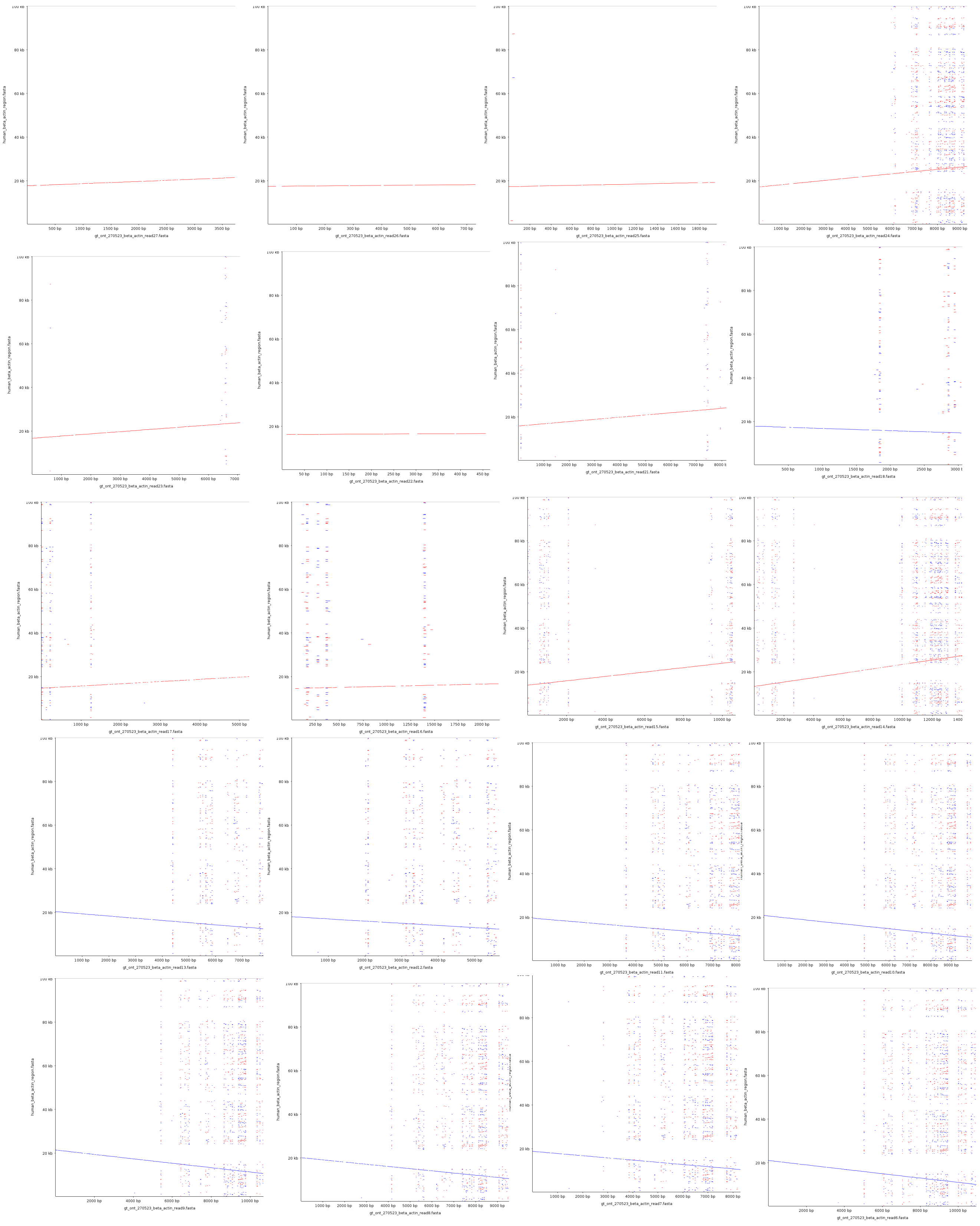
human_beta_actin_region.fasta
gt_ont_270523_beta_actin_read27.fasta
gt_ont_270523_beta_actin_read26.fasta
gt_ont_270523_beta_actin_read25.fasta
gt_ont_270523_beta_actin_read24.fasta
gt_ont_270523_beta_actin_read23.fasta
gt_ont_270523_beta_actin_read22.fasta
gt_ont_270523_beta_actin_read21.fasta
gt_ont_270523_beta_actin_read18.fasta
gt_ont_270523_beta_actin_read17.fasta
gt_ont_270523_beta_actin_read16.fasta
gt_ont_270523_beta_actin_read15.fasta
gt_ont_270523_beta_actin_read14.fasta
gt_ont_270523_beta_actin_read13.fasta
gt_ont_270523_beta_actin_read12.fasta
gt_ont_270523_beta_actin_read11.fasta
gt_ont_270523_beta_actin_read10.fasta
gt_ont_270523_beta_actin_read9.fasta
gt_ont_270523_beta_actin_read8.fasta
gt_ont_270523_beta_actin_read7.fasta
gt_ont_270523_beta_actin_read6.fasta

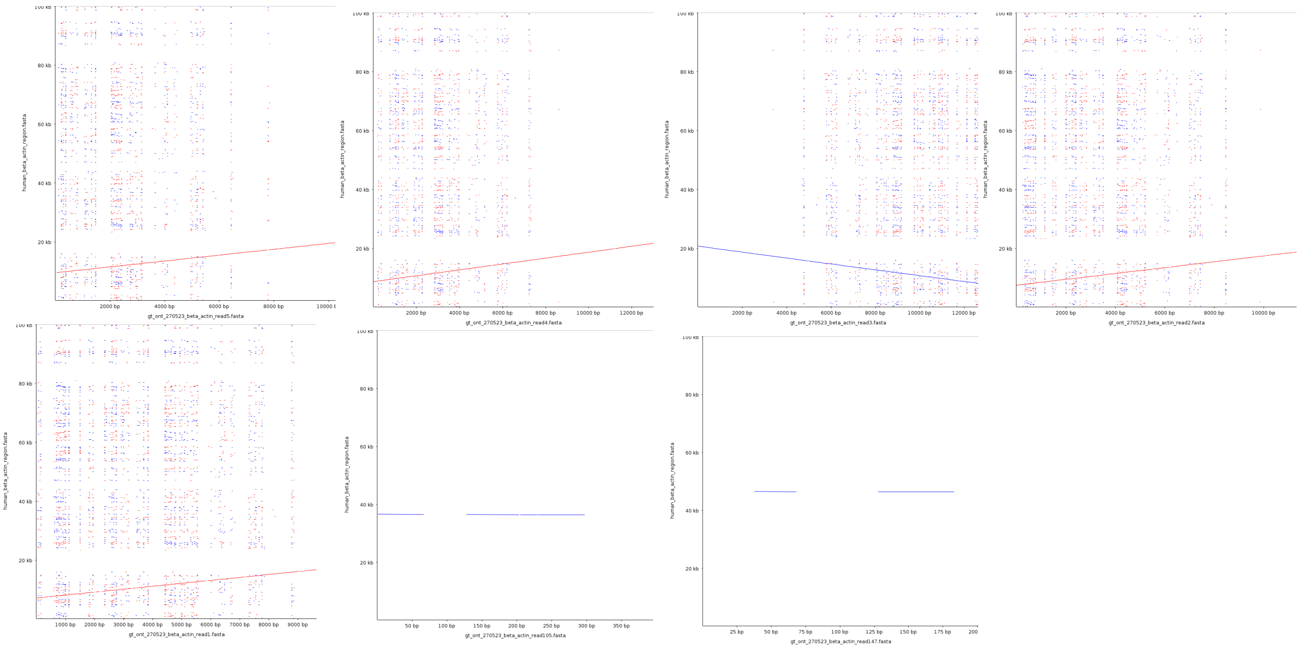

human_beta_actin_region.fasta
gt_ont_270523_beta_actin_read5.fasta
gt_ont_270523_beta_actin_read4.fasta
gt_ont_270523_beta_actin_read3.fasta
gt_ont_270523_beta_actin_read2.fasta
gt_ont_270523_beta_actin_read1.fasta
gt_ont_270523_beta_actin_read105.fasta
gt_ont_270523_beta_actin_read147.fasta

Duplex reads

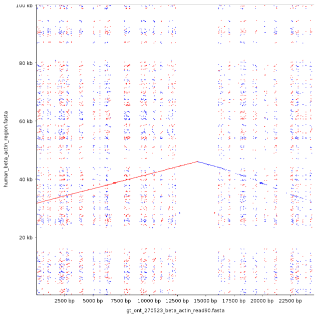

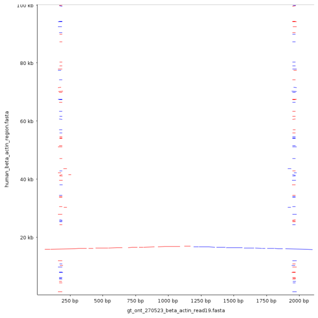

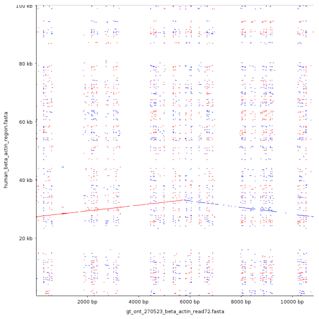

Incomplete alignments

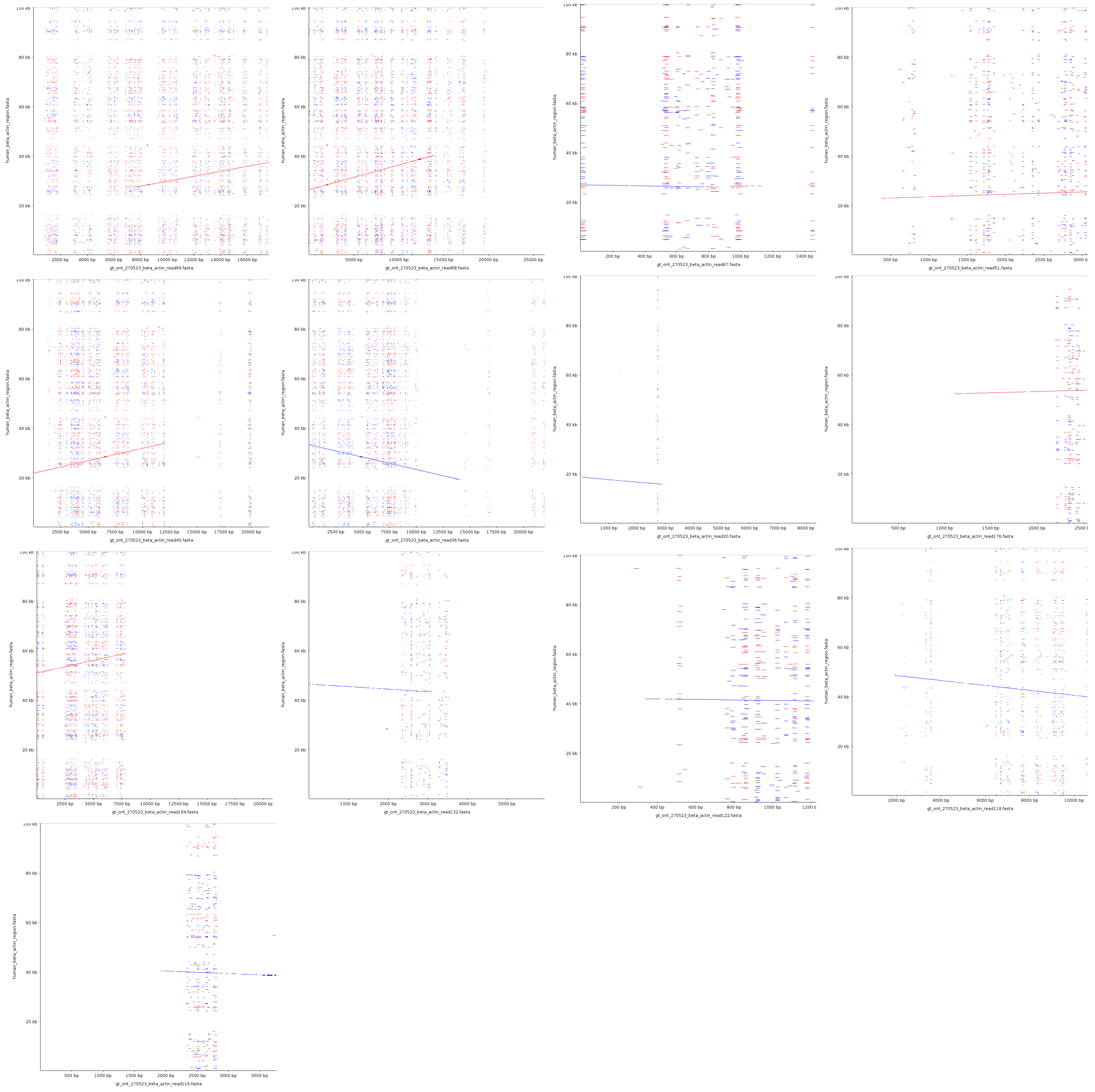

Y axis: ACTB gene and surrounding region in human genome
X axis: Nanopore read

Normal reads

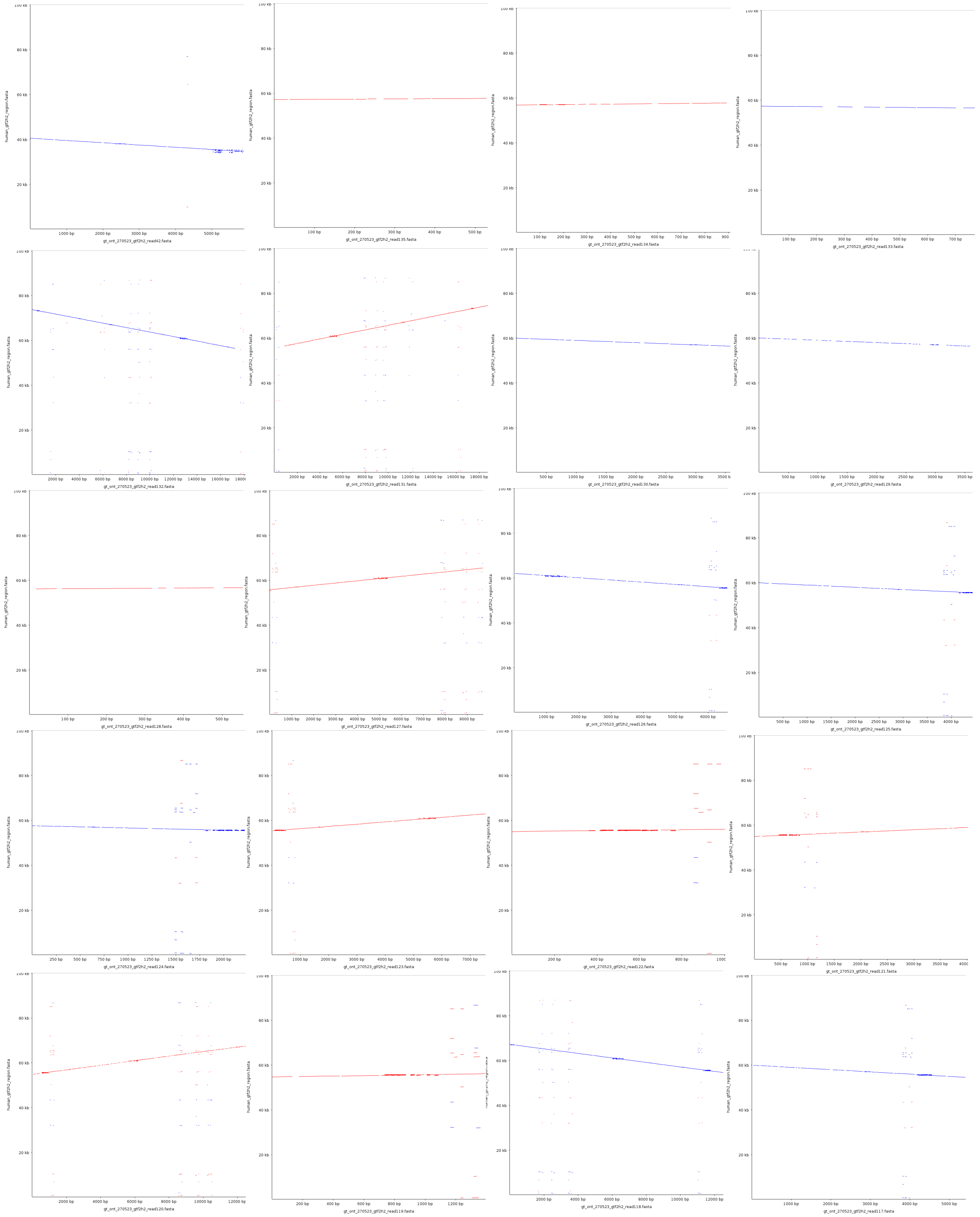

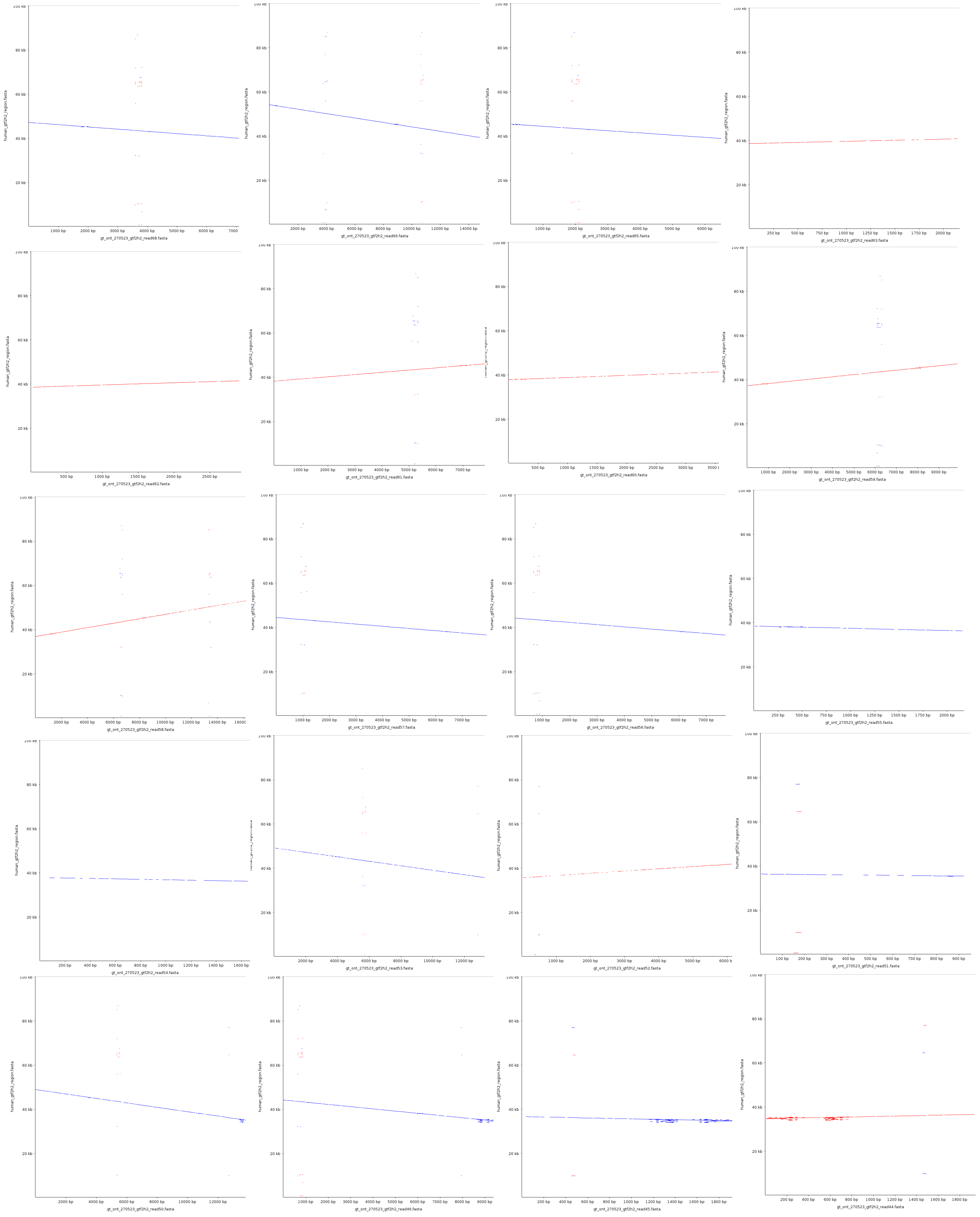
human_gtf2h2_region.fasta
gt_ont_270523_gtf2h2_read68.fasta
gt_ont_270523_gtf2h2_read66.fasta
gt_ont_270523_gtf2h2_read65.fasta
gt_ont_270523_gtf2h2_read63.fasta
gt_ont_270523_gtf2h2_read62.fasta
gt_ont_270523_gtf2h2_read61.fasta
gt_ont_270523_gtf2h2_read60.fasta
gt_ont_270523_gtf2h2_read59.fasta
gt_ont_270523_gtf2h2_read58.fasta
gt_ont_270523_gtf2h2_read57.fasta
gt_ont_270523_gtf2h2_read56.fasta
gt_ont_270523_gtf2h2_read55.fasta
gt_ont_270523_gtf2h2_read54.fasta
gt_ont_270523_gtf2h2_read53.fasta
gt_ont_270523_gtf2h2_read52.fasta
gt_ont_270523_gtf2h2_read51.fasta
gt_ont_270523_gtf2h2_read50.fasta
gt_ont_270523_gtf2h2_read46.fasta
gt_ont_270523_gtf2h2_read45.fasta
gt_ont_270523_gtf2h2_read44.fasta

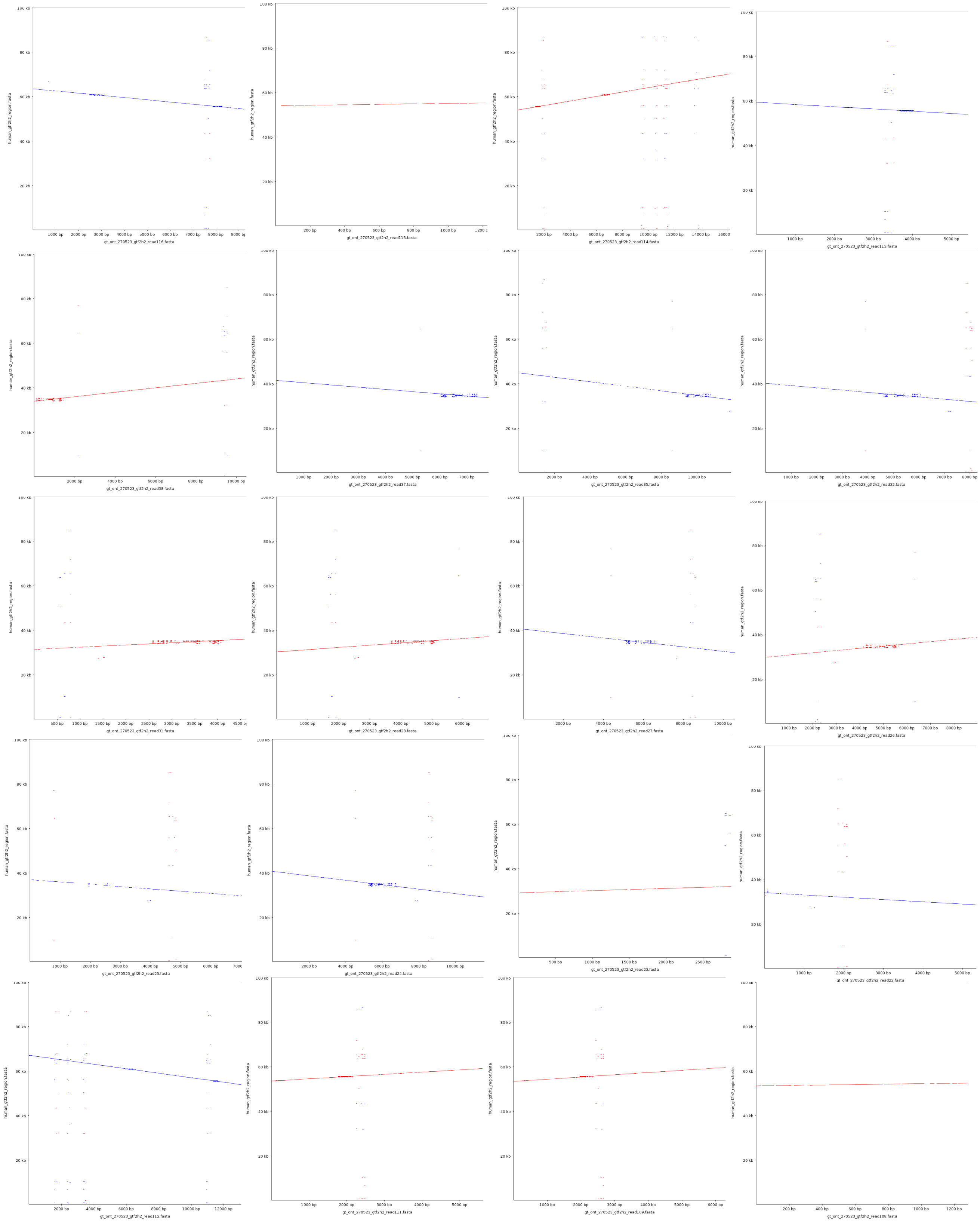
human_gtf2h2_region.fasta
gt_ont_270523_gtf2h2_read116.fasta
gt_ont_270523_gtf2h2_read115.fasta
gt_ont_270523_gtf2h2_read114.fasta
gt_ont_270523_gtf2h2_read113.fasta
gt_ont_270523_gtf2h2_read38.fasta
gt_ont_270523_gtf2h2_read37.fasta
gt_ont_270523_gtf2h2_read35.fasta
gt_ont_270523_gtf2h2_read32.fasta
gt_ont_270523_gtf2h2_read31.fasta
gt_ont_270523_gtf2h2_read28.fasta
gt_ont_270523_gtf2h2_read27.fasta
gt_ont_270523_gtf2h2_read26.fasta
gt_ont_270523_gtf2h2_read25.fasta
gt_ont_270523_gtf2h2_read24.fasta
gt_ont_270523_gtf2h2_read23.fasta
gt_ont_270523_gtf2h2_read22.fasta
gt_ont_270523_gtf2h2_read112.fasta
gt_ont_270523_gtf2h2_read111.fasta
gt_ont_270523_gtf2h2_read109.fasta
gt_ont_270523_gtf2h2_read108.fasta

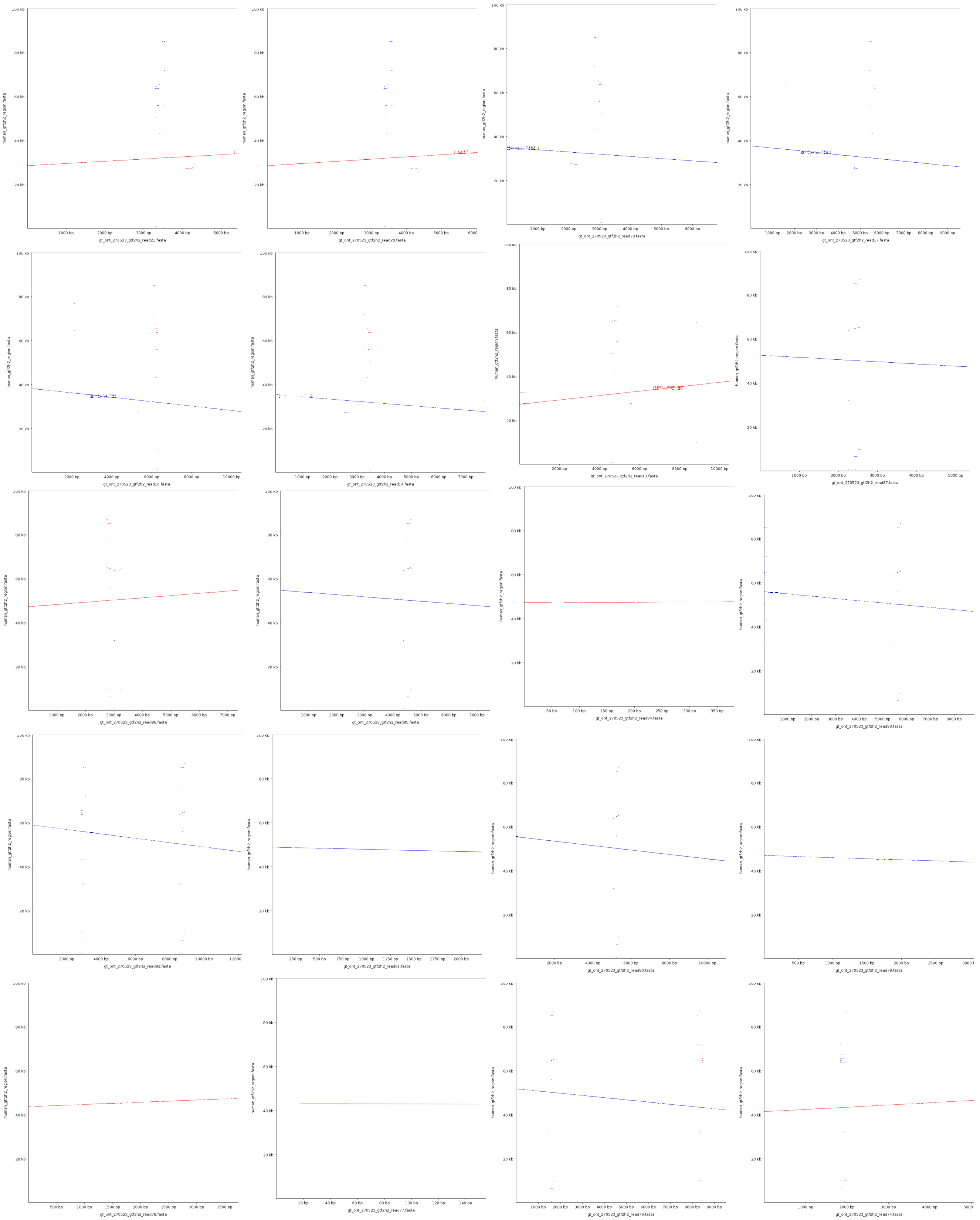
human_gtf2h2_region.fasta
gt_ont_270523_gtf2h2_read21.fasta
gt_ont_270523_gtf2h2_read20.fasta
gt_ont_270523_gtf2h2_read18.fasta
gt_ont_270523_gtf2h2_read17.fasta
gt_ont_270523_gtf2h2_read16.fasta
gt_ont_270523_gtf2h2_read14.fasta
gt_ont_270523_gtf2h2_read13.fasta
gt_ont_270523_gtf2h2_read87.fasta
gt_ont_270523_gtf2h2_read86.fasta
gt_ont_270523_gtf2h2_read85.fasta
gt_ont_270523_gtf2h2_read84.fasta
gt_ont_270523_gtf2h2_read83.fasta
gt_ont_270523_gtf2h2_read82.fasta
gt_ont_270523_gtf2h2_read81.fasta
gt_ont_270523_gtf2h2_read80.fasta
gt_ont_270523_gtf2h2_read79.fasta
gt_ont_270523_gtf2h2_read78.fasta
gt_ont_270523_gtf2h2_read77.fasta
gt_ont_270523_gtf2h2_read76.fasta
gt_ont_270523_gtf2h2_read74.fasta

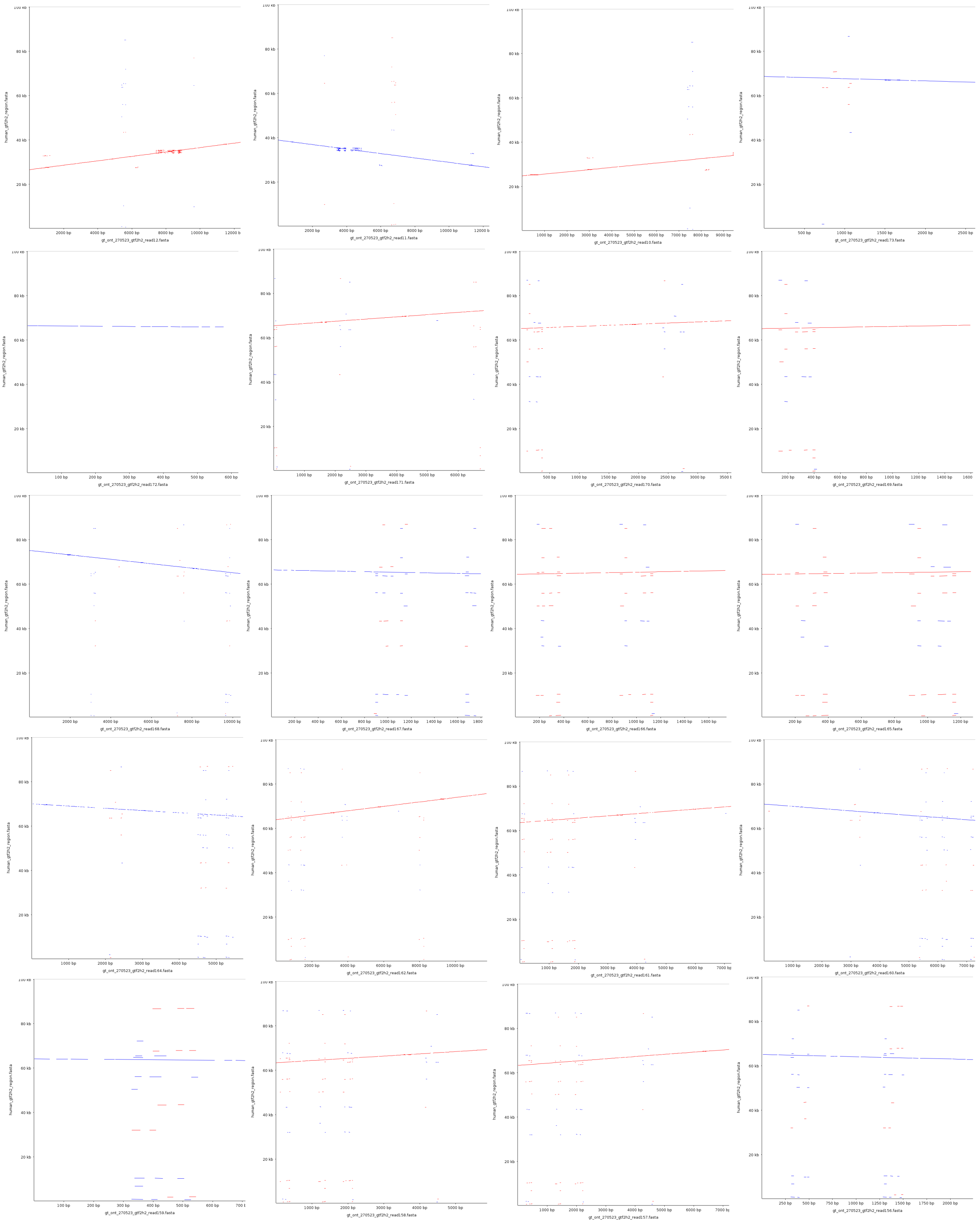
human_gtf2h2_region.fasta
gt_ont_270523_gtf2h2_read12.fasta
gt_ont_270523_gtf2h2_read11.fasta
gt_ont_270523_gtf2h2_read10.fasta
gt_ont_270523_gtf2h2_read173.fasta
gt_ont_270523_gtf2h2_read172.fasta
gt_ont_270523_gtf2h2_read171.fasta
gt_ont_270523_gtf2h2_read170.fasta
gt_ont_270523_gtf2h2_read169.fasta
gt_ont_270523_gtf2h2_read168.fasta
gt_ont_270523_gtf2h2_read167.fasta
gt_ont_270523_gtf2h2_read166.fasta
gt_ont_270523_gtf2h2_read165.fasta
gt_ont_270523_gtf2h2_read164.fasta
gt_ont_270523_gtf2h2_read162.fasta
gt_ont_270523_gtf2h2_read161.fasta
gt_ont_270523_gtf2h2_read160.fasta
gt_ont_270523_gtf2h2_read159.fasta
gt_ont_270523_gtf2h2_read158.fasta
gt_ont_270523_gtf2h2_read157.fasta
gt_ont_270523_gtf2h2_read156.fasta

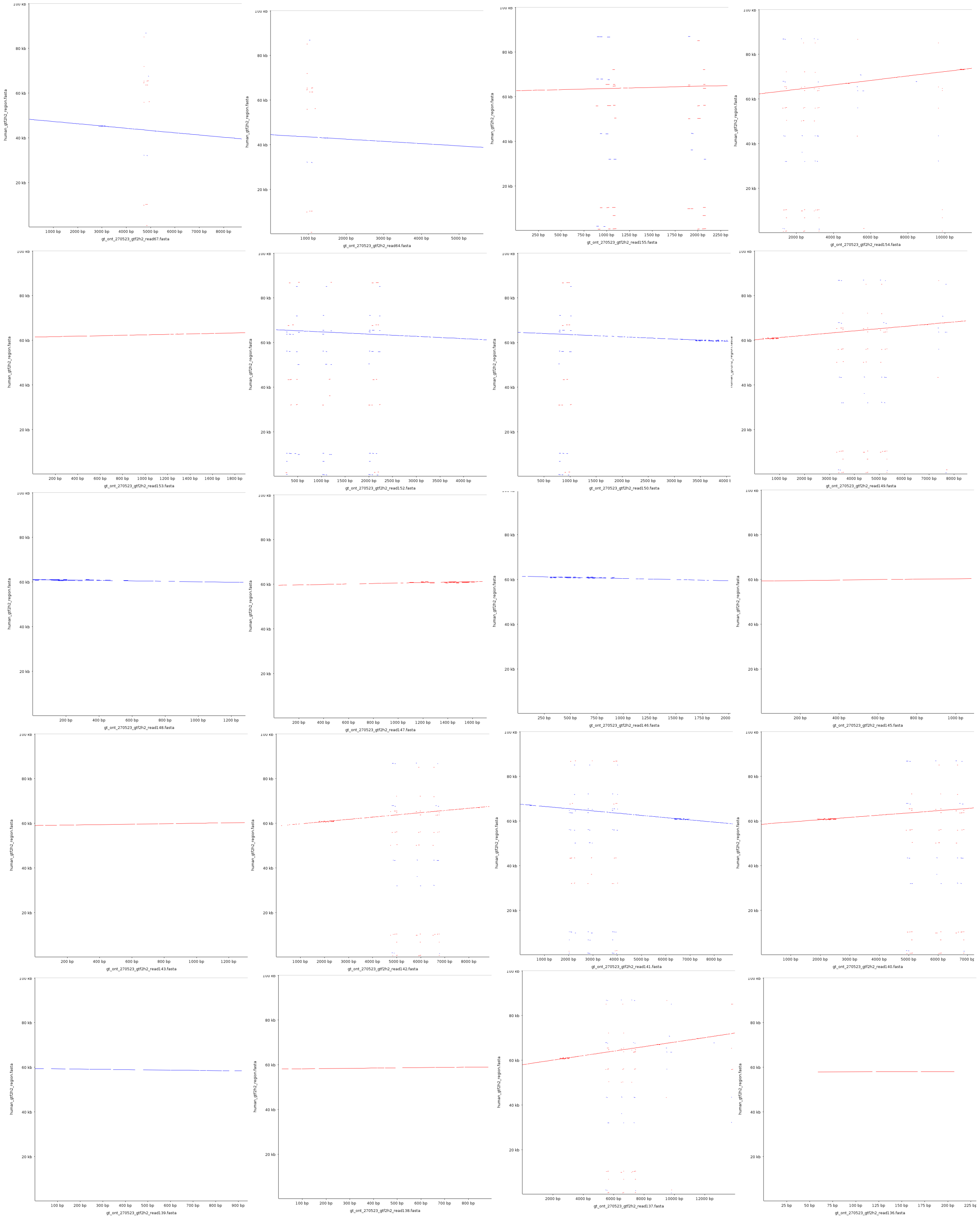
human_gtf2h2_region.fasta
gt_ont_270523_gtf2h2_read67.fasta
gt_ont_270523_gtf2h2_read64.fasta
gt_ont_270523_gtf2h2_read155.fasta
gt_ont_270523_gtf2h2_read154.fasta
gt_ont_270523_gtf2h2_read153.fasta
gt_ont_270523_gtf2h2_read152.fasta
gt_ont_270523_gtf2h2_read150.fasta
gt_ont_270523_gtf2h2_read149.fasta
gt_ont_270523_gtf2h2_read148.fasta
gt_ont_270523_gtf2h2_read147.fasta
gt_ont_270523_gtf2h2_read146.fasta
gt_ont_270523_gtf2h2_read145.fasta
gt_ont_270523_gtf2h2_read143.fasta
gt_ont_270523_gtf2h2_read142.fasta
gt_ont_270523_gtf2h2_read141.fasta
gt_ont_270523_gtf2h2_read140.fasta
gt_ont_270523_gtf2h2_read139.fasta
gt_ont_270523_gtf2h2_read138.fasta
gt_ont_270523_gtf2h2_read137.fasta
gt_ont_270523_gtf2h2_read136.fasta

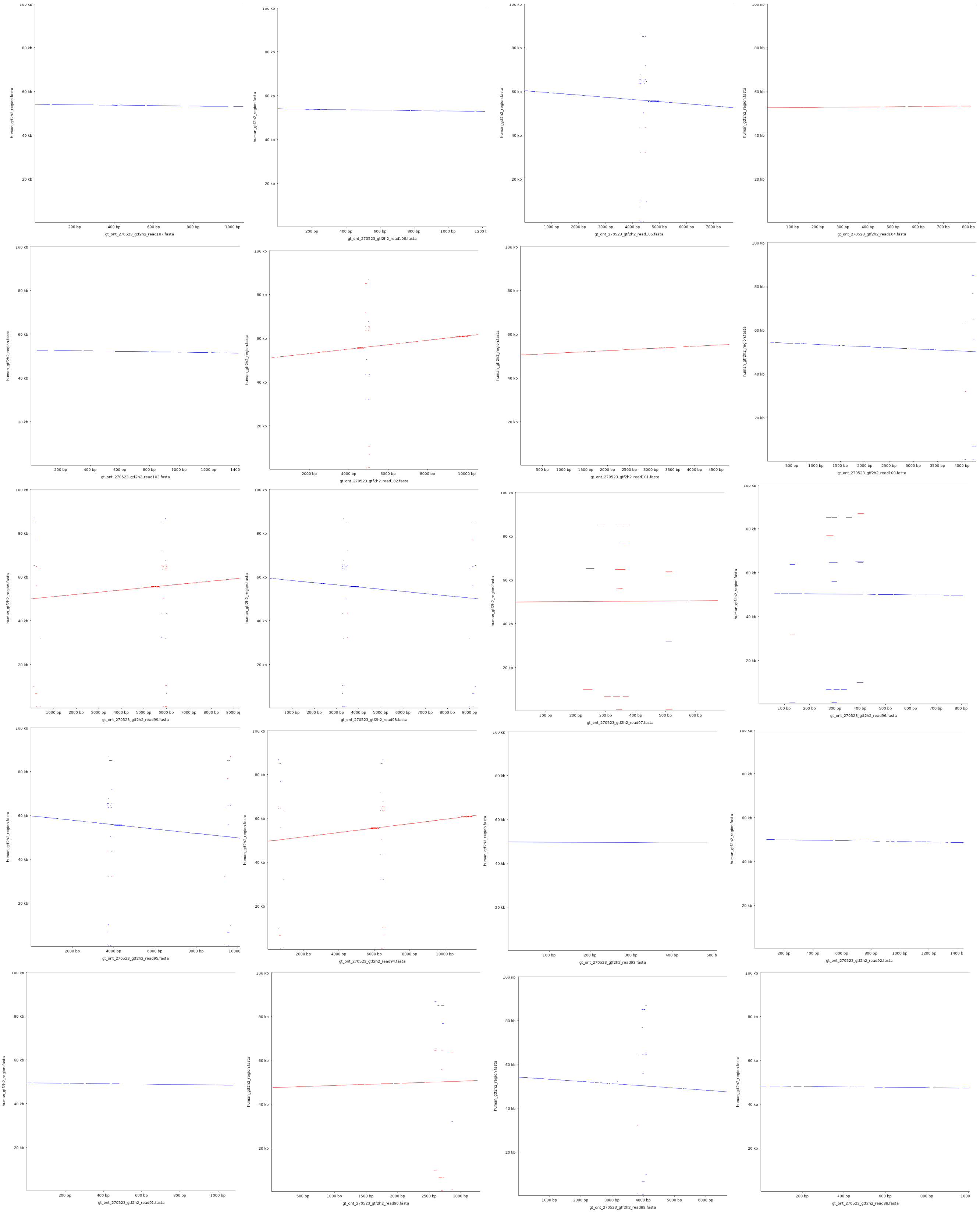
human_gtf2h2_region.fasta
gt_ont_270523_gtf2h2_read107.fasta
gt_ont_270523_gtf2h2_read106.fasta
gt_ont_270523_gtf2h2_read105.fasta
gt_ont_270523_gtf2h2_read104.fasta
gt_ont_270523_gtf2h2_read103.fasta
gt_ont_270523_gtf2h2_read102.fasta
gt_ont_270523_gtf2h2_read101.fasta
gt_ont_270523_gtf2h2_read100.fasta
gt_ont_270523_gtf2h2_read99.fasta
gt_ont_270523_gtf2h2_read98.fasta
gt_ont_270523_gtf2h2_read97.fasta
gt_ont_270523_gtf2h2_read96.fasta
gt_ont_270523_gtf2h2_read95.fasta
gt_ont_270523_gtf2h2_read94.fasta
gt_ont_270523_gtf2h2_read93.fasta
gt_ont_270523_gtf2h2_read92.fasta
gt_ont_270523_gtf2h2_read91.fasta
gt_ont_270523_gtf2h2_read90.fasta
gt_ont_270523_gtf2h2_read89.fasta
gt_ont_270523_gtf2h2_read88.fasta

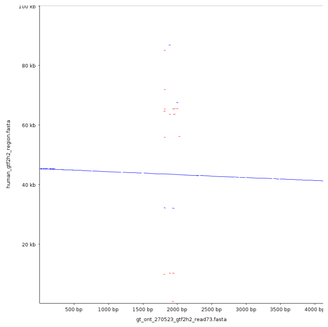
100 kb
80 kb
60 kb
40 kb
20 kb
human_gtf2h2_region.fasta
500 bp
1000 bp
1500 bp
2000 bp
2500 bp
3000 bp
3500 bp
4000 bp
gt_ont_270523_gtf2h2_read73.fasta

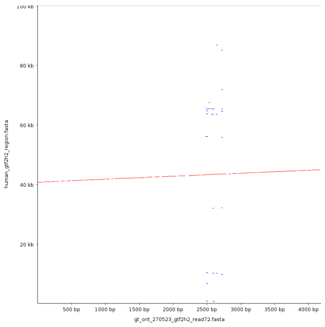
100 kb
80 kb
60 kb
40 kb
20 kb
human_gtf2h2_region.fasta
500 bp
1000 bp
1500 bp
2000 bp
2500 bp
3000 bp
3500 bp
4000 bp
gt_ont_270523_gtf2h2_read72.fasta

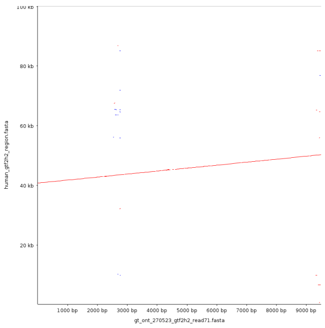
100 kb
80 kb
60 kb
40 kb
20 kb
human_gtf2h2_region.fasta
1000 bp
2000 bp
3000 bp
4000 bp
5000 bp
6000 bp
7000 bp
8000 bp
9000 bp
gt_ont_270523_gtf2h2_read71.fasta

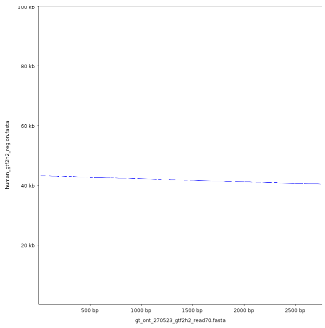
100 kb
80 kb
60 kb
40 kb
20 kb
human_gtf2h2_region.fasta
500 bp
1000 bp
1500 bp
2000 bp
2500 bp
gt_ont_270523_gtf2h2_read70.fasta

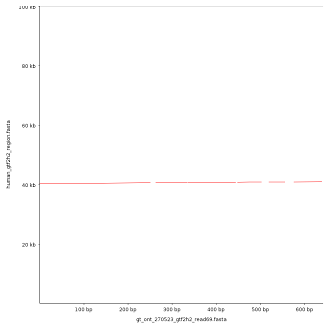
100 kb
80 kb
60 kb
40 kb
20 kb
human_gtf2h2_region.fasta
100 bp
200 bp
300 bp
400 bp
500 bp
600 bp
gt_ont_270523_gtf2h2_read69.fasta

Incomplete alignments

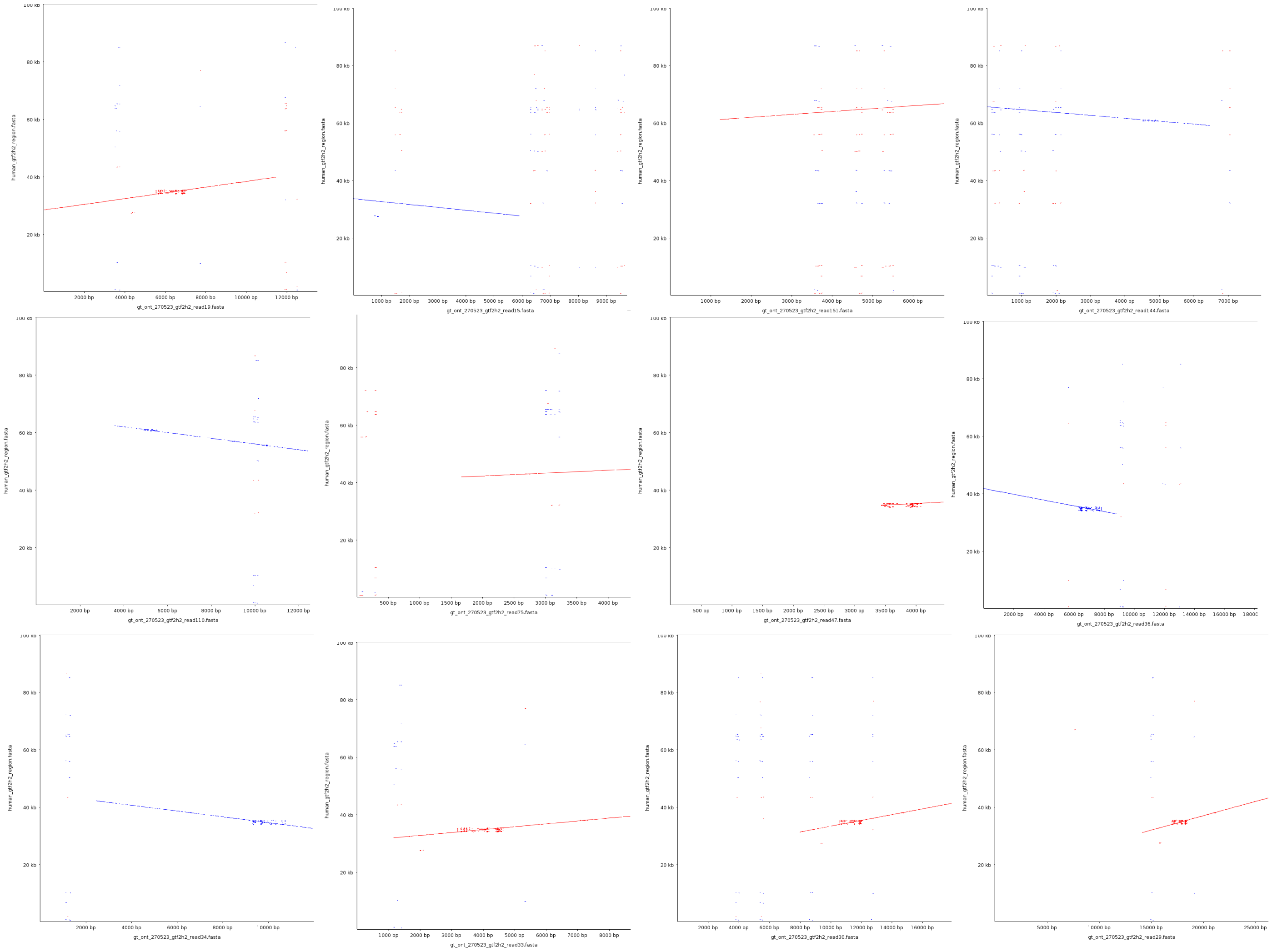

Poor alignments

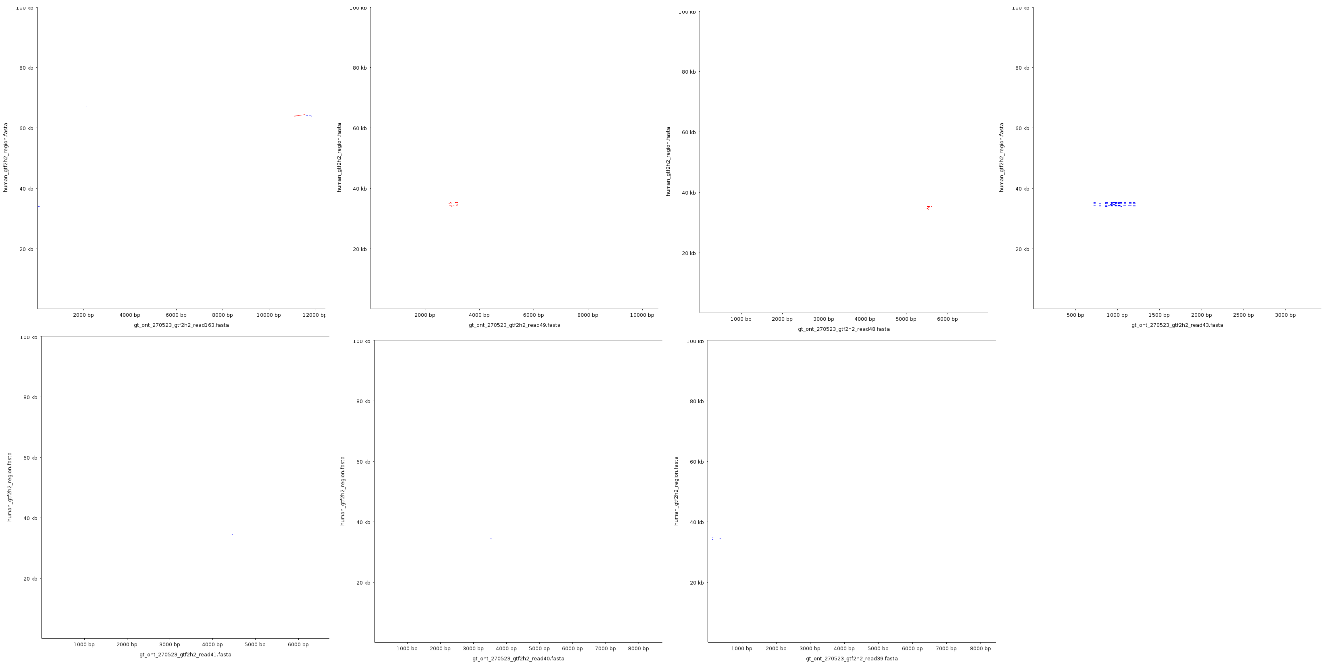

Supplement: Supplementary file 1 — Supplementary Tables 1–4 and Figs. 1 and 2. [file 41591_2025_4073_MOESM1_ESM.pdf]
