# Supplementary material for: Contaminating plasmid sequences and disrupted vector genomes in the liver following adeno-associated virus gene therapy
Source: Nat Med. 2026 Jan 16;32(2):472–80. doi: 10.1038/s41591-025-04073-z (PMC12920116; doi:10.1038/s41591-025-04073-z)
Supplement: Supplementary file 2 — Reporting Summary [file 41591_2025_4073_MOESM2_ESM.pdf]

nature portfolio

Corresponding author(s): Judith Breuer

Last updated by author(s): Aug 14, 2025

Reporting Summary

Nature Portfolio wishes to improve the reproducibility of the work that we publish. This form provides structure for consistency and transparency in reporting. For further information on Nature Portfolio policies, see our Editorial Policies and the Editorial Policy Checklist.

Statistics

For all statistical analyses, confirm that the following items are present in the figure legend, table legend, main text, or Methods section.

n/a	Confirmed	
☐	☒	The exact sample size (*n*) for each experimental group/condition, given as a discrete number and unit of measurement
☒	☐	A statement on whether measurements were taken from distinct samples or whether the same sample was measured repeatedly
☒	☐	The statistical test(s) used AND whether they are one- or two-sided<br>*Only common tests should be described solely by name; describe more complex techniques in the Methods section.*
☒	☐	A description of all covariates tested
☒	☐	A description of any assumptions or corrections, such as tests of normality and adjustment for multiple comparisons
☒	☐	A full description of the statistical parameters including central tendency (e.g. means) or other basic estimates (e.g. regression coefficient) AND variation (e.g. standard deviation) or associated estimates of uncertainty (e.g. confidence intervals)
☒	☐	For null hypothesis testing, the test statistic (e.g. *F*, *t*, *r*) with confidence intervals, effect sizes, degrees of freedom and *P* value noted<br>*Give P values as exact values whenever suitable.*
☒	☐	For Bayesian analysis, information on the choice of priors and Markov chain Monte Carlo settings
☒	☐	For hierarchical and complex designs, identification of the appropriate level for tests and full reporting of outcomes
☒	☐	Estimates of effect sizes (e.g. Cohen's *d*, Pearson's *r*), indicating how they were calculated

Our web collection on statistics for biologists contains articles on many of the points above.

Software and code

Policy information about availability of computer code

Data collection: No specific software was used for data collection.

Data analysis:
bowtie2 v2.4.1
samtools v1.14/v1.15
minimap2 v2.17
redotable v1.1
porechop 0.2.4
nextflow 23.04.3
taxprofiler v1.1.0
bracken v2.8
R v4.2.2
tidyverse v2.0.0
GenomicAlignments v1.32.1
metaMix v0.4
fastp v0.23.2
PEAR v0.9.11
STAR v2.7.10b
pyRanges v0.1.4
pyCirclize v1.9.1
HALO v3.6

GraphPad prism v10
Code used for analysis in this study is available at https://github.com/sarah-buddle/aav-gene-therapy

For manuscripts utilizing custom algorithms or software that are central to the research but not yet described in published literature, software must be made available to editors and reviewers. We strongly encourage code deposition in a community repository (e.g. GitHub). See the Nature Portfolio guidelines for submitting code & software for further information.

Data

Policy information about availability of data

All manuscripts must include a data availability statement. This statement should provide the following information, where applicable:

- Accession codes, unique identifiers, or web links for publicly available datasets
- A description of any restrictions on data availability
- For clinical datasets or third party data, please ensure that the statement adheres to our policy

Full sequencing datasets are not available due to the presence of human genetic data. Human filtered datasets are available to reviewers and on reasonable request to the authors.

Research involving human participants, their data, or biological material

Policy information about studies with human participants or human data. See also policy information about sex, gender (identity/presentation), and sexual orientation and race, ethnicity and racism.

Reporting on sex and gender	No sex or gender based analyses were performed since our study focuses on a single case.
Reporting on race, ethnicity, or other socially relevant groupings	Race and ethnicity were not reported or analysed since our study focuses on a single case.
Population characteristics	This study focused on a single case, a 7 year-old female patient with spinal muscular atrophy.
Recruitment	Single case, recruited due to clinical presentation
Ethics oversight	The liver biopsy procedure was performed for diagnostic purposes. Residual material was analysed in this study with written informed consent for additional research given by a parent, under the International Severe Acute Respiratory and Emerging Infection Consortium (ISARIC) WHO Clinical Characterisation Protocol UK (CCP-UK) (ISRCTN 66726260). Ethical approval for the ISARIC CCP-UK study was given by the South Central–Oxford Research Ethics Committee in England (13/SC/0149), the Scotland A Research Ethics Committee (20/SS/0028) and the WHO Ethics Review Committee (RPC571 and RPC572).

Note that full information on the approval of the study protocol must also be provided in the manuscript.

Field-specific reporting

Please select the one below that is the best fit for your research. If you are not sure, read the appropriate sections before making your selection.

☒ Life sciences ☐ Behavioural & social sciences ☐ Ecological, evolutionary & environmental sciences

For a reference copy of the document with all sections, see nature.com/documents/nr-reporting-summary-flat.pdf

Life sciences study design

All studies must disclose on these points even when the disclosure is negative.

Sample size	This is a report on a single case. Sample size is one.
Data exclusions	No data were excluded.
Replication	No replicates were performed since this was a case report and sample volumes were very limited.
Randomization	Not relevant - single case
Blinding	Not relevant - single case

Reporting for specific materials, systems and methods

We require information from authors about some types of materials, experimental systems and methods used in many studies. Here, indicate whether each material, system or method listed is relevant to your study. If you are not sure if a list item applies to your research, read the appropriate section before selecting a response.

Materials & experimental systems

n/a	Involved in the study
☐	☒ Antibodies
☒	☐ Eukaryotic cell lines
☒	☐ Palaeontology and archaeology
☒	☐ Animals and other organisms
☒	☐ Clinical data
☒	☐ Dual use research of concern
☒	☐ Plants

Methods

n/a	Involved in the study
☒	☐ ChIP-seq
☒	☐ Flow cytometry
☒	☐ MRI-based neuroimaging

Antibodies

Antibodies used

anti-CD4 (clone SP35, Roche, 790-4423), anti-CD8 (clone SP239, Roche, 790-7176) and anti-CD20 (clone L26, Dako (Agilent), M0755), anti-adenovirus (clone 2/6 and 20/11, Roche, 760-4870)

Validation

All immunohistochemistry was performed with a known positive control tissue in a clinical diagnostic laboratory and interpreted by a qualified pathologist in conjunction with histological examination and relevant clinical information.

Manufacturer validation statements:

1. Anti-CD4 (clone SP35, Roche, 790-4423) Species: rabbit. Pre-diluted.
The detection of CD4 by immunohistochemistry (IHC) with the CONFIRM anti-CD4 (SP35) Rabbit Monoclonal Primary Antibody (CONFIRM anti-CD4 (SP35) antibody), when evaluated with other markers, may be used to aid in the identification of normal helper T-cells and sub-classification of T-cell lymphoma. The cellular staining pattern for CONFIRM anti-CD4 (SP35) antibody is membranous.

1. Dabbs DJ. Diagnostic Immunohistochemistry Theranostic and Genomic Applications, 5th edition. Vol 5. Amsterdam, Netherlands: Elsevier; 2019.
2. Naeim F. Principles of Immunophenotyping. In: Naeim F, Rao PN, Grody WW, eds. Hematopathology: Morphology, Immunophenotype, Cytogenetics, and Molecular Approaches. Cambridge, MA: Academic Press; 2009.
3. Swerdlow SH, Campo E, Harris NL, et al. WHO Classification of Tumours of Haematopoietic and Lymphoid Tissues, 4th edition. Vol 4. Lyon, France: International Agency for Research on Cancer; 2008.
4. Higgins RA, Blankenship JE, Kinney MC. Application of immunohistochemistry in the diagnosis of non-Hodgkin and Hodgkin lymphoma. Arch Pathol Lab Med. 2008;132(3):441-461.

2. Anti-CD8 (clone SP239, Roche, 790-7176) Species: rabbit. Pre-diluted.
The detection of CD8 by immunohistochemistry (IHC) with the anti-CD8 (SP239) Rabbit Monoclonal Primary Antibody (anti-CD8 (SP239) antibody) maybe used to aid in the identification of a subset of normal T-cells and sub-classification of T-cell lymphoma.

1. Rich R, Fleisher T, Shearer W, Frew A, Weyand C. Clinical Immunology Principles and Practice, 5th edition. Vol 5. Amsterdam, Netherlands: Elsevier; 2018.
2. Dabbs DJ. Diagnostic Immunohistochemistry Theranostic and Genomic Applications, 5th edition. Vol 5. Amsterdam, Netherlands: Elsevier; 2019.
3. Higgins RA, Blankenship JE, Kinney MC. Application of immunohistochemistry in the diagnosis of non-Hodgkin and Hodgkin lymphoma. Arch Pathol Lab Med. 2008;132(3):441-461.
4. Naeim F. Principles of Immunophenotyping. In: Naeim F, Rao PN, Grody WW, eds. Hematopathology: Morphology, Immunophenotype, Cytogenetics, and Molecular Approaches. Cambridge, MA: Academic Press; 2009.
5. Swerdlow SH, Campo E, Harris NL, et al. WHO Classification of Tumours of Haematopoietic and Lymphoid Tissues, 4th edition. Vol 4. Lyon, France: International Agency for Research on Cancer; 2008.

3. Anti-CD20 (clone L26, Dako (Agilent), M0755) Species: mouse. Used at 1:100 dilution
Monoclonal Mouse Anti-Human CD20cy, Clone L26, is intended for use in immunohistochemistry (IHC). The antibody labels cells of the B-cell lineage and is a useful aid for the classification of neoplasms of B-cell derivation (1). CD20 is a transmembrane, non-glycosylated protein expressed on B-cell precursors and mature B cells, but is lost following differentiation into plasma cells (3). In resting B cells, CD20 appears in a 33 kDa non-phosphorylated form. After mitogen stimulation, CD20 becomes heavily phosphorylated (35-37 kDa isoforms), and it is a dominant phosphoprotein in activated B cells, B-cell lines, and hairy cell leukemias (2). The long N- and C-terminal ends of the protein are located on the cytoplasmic side of the membrane and only a minor portion of the protein is exposed on the cell surface (3). Antibodies reacting with CD20 cytoplasmic epitopes are designated CD20cy (2). It is suggested that CD20 plays a direct role in regulating the transmembrane conductive Ca2+ flux of B cells which indicates a possible function for CD20 as a regulator of proliferation and differentiation (3).

References
1. Takami T, Qi C-F, Yamada T, Yamashina M, Kon S-I, Ishii Y, et al. B20.3. Reactivity and specificity of L26 (pan-B-cell mAb) on 322 cases of fresh and paraffin-embedded
lymphoproliferative diseases. In: Knapp W, Dörken B, Gilks WR, Rieber EP, Schmidt RE, Stein H, et al., editors. Leukocyte typing IV. White cell differentiation antigens.
Proceedings of the 4th International Workshop and Conference; 1989 Feb 21-25; Vienna, Austria. Oxford, New York, Tokyo: Oxford University Press; 1989. p. 134-6.
2. Zhou L-J, Tedder TF. CD20 workshop panel report. In: Schlossman SF, Boumsell L, Gilks W, Harlan JM, Kishimoto T, Morimoto C, et al., editors. Leukocyte typing V.
White cell differentiation antigens. Proceedings of the 5th International Workshop and Conference; 1993 Nov 3-7; Boston, USA.

Oxford, New York, Tokyo: Oxford
University Press; 1995. p. 511-4.
3. Tedder TF, Engel P. CD20: a regulator of cell-cycle progression of B lymphocytes. Immunology Today 1994;15:450-4.
4. Ishii Y, Takami T, Yuasa H, Takei T, Kikuchi K. Two distinct antigen systems in human B lymphocytes: identification of cell surface and intracellular antigens using
monoclonal antibodies. Clin Exp Immunol 1984;58:183-92.
5. Mason DY, Comans-Bitter WM, Cordell JL, Verhoeven MAJ, van Dongen JJM. Antibody L26 recognizes an intracellular epitope on the B-cell-associated CD20 antigen.
Am J Pathol 1990;136:1215-22.
6. Cartun RW, Coles FB, Pastuszak WT. Utilization of monoclonal antibody L26 in the identification and confirmation of B-cell lymphomas. A sensitive and specific marker
applicable to formalin- and B5-fixed, paraffin-embedded tissues. Am J Pathol 1987;129:415-21.
7. Norton AJ, Isaacson PG. Monoclonal antibody L26: an antibody that is reactive with normal and neoplastic B lymphocytes in routinely fixed and paraffin wax embedded
tissue. J Clin Pathol 1987;40:1405-12.
8. Blakolmer K, Vesely M, Kummer JA, Jurecka W, Mannhalter C, Chott A. Immunoreactivity of B-cell markers (CD79a, L26) in rare cases of extranodal cytotoxic peripheral
T- (NK/T-) cell lymphomas. Mod Pathol 2000;13:766-72.

4. Anti-adenovirus (clone 2/6 and 20/11, Roche, 760-4870) Species: Mouse. Pre-diluted.
Adenovirus (20/11 & 2/6) Mouse Monoclonal Primary Antibody is intended for laboratory use in the detection of Adenovirus in formalin-fixed, paraffin-embedded tissue stained on VENTANA BenchMark IHC/ISH instruments. This product should be interpreted by a qualified pathologist in conjunction with histological examination, relevant clinical information, and proper controls. This antibody is intended for in vitro diagnostic (IVD) use.

References
1. Ison, MG. Adenovirus Infections in Transplant Recipients. Clin Infect Dis. 2006; 43:331–9.
2. Shayan K, et al. Adenovirus enterocolitis in pediatric patients following bone marrow transplantation: report of 2 cases and review of the literature. Arch Pathol Lab Med. 2003; 127:1615-8.

Plants

Seed stocks

Report on the source of all seed stocks or other plant material used. If applicable, state the seed stock centre and catalogue number. If plant specimens were collected from the field, describe the collection location, date and sampling procedures.

Novel plant genotypes

Describe the methods by which all novel plant genotypes were produced. This includes those generated by transgenic approaches, gene editing, chemical/radiation-based mutagenesis and hybridization. For transgenic lines, describe the transformation method, the number of independent lines analyzed and the generation upon which experiments were performed. For gene-edited lines, describe the editor used, the endogenous sequence targeted for editing, the targeting guide RNA sequence (if applicable) and how the editor was applied.

Authentication

Describe any authentication procedures for each seed stock used or novel genotype generated. Describe any experiments used to assess the effect of a mutation and, where applicable, how potential secondary effects (e.g. second site T-DNA insertions, mosiacism, off-target gene editing) were examined.
